# Palladium-Catalyzed Aminocyclization–Coupling Cascades: Preparation of Dehydrotryptophan Derivatives and Computational Study

**DOI:** 10.1021/acs.joc.1c00636

**Published:** 2021-06-14

**Authors:** Belén Vaz, Claudio Martínez, Francisco Cruz, J. Gabriel Denis, Ángel R. de Lera, José M. Aurrecoechea, Rosana Álvarez

**Affiliations:** † Departamento de Química Orgánica, Facultad de Química (CINBIO) and Instituto de Investigación Biomédica de Vigo (IBIV), Universidade de Vigo, Lagoas-Marcosende, 36310 Vigo, Spain; ‡ Departamento de Química Orgánica e Inorgánica, Facultad de Ciencia y Tecnología, Universidad del País Vasco UPV/EHU, Apartado 644, 48080 Bilbao, Spain

## Abstract

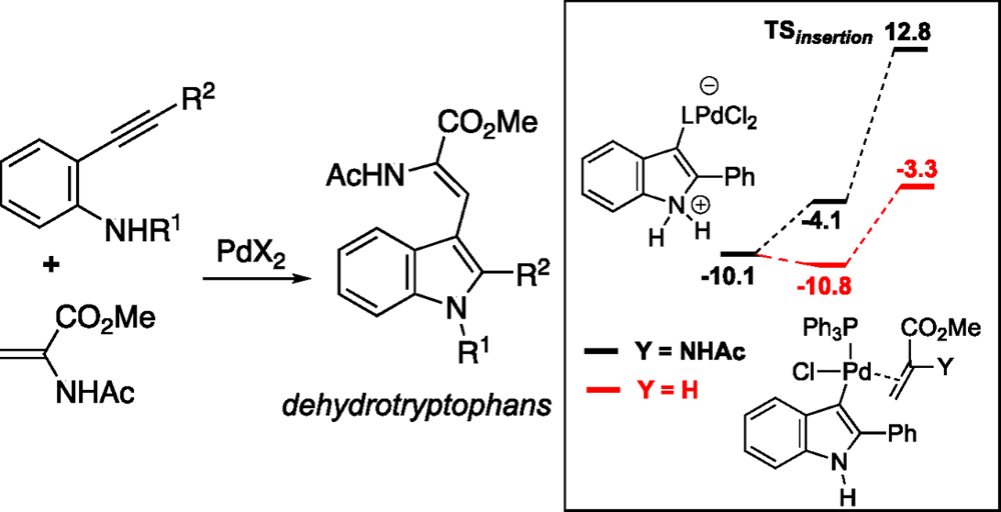

Dehydrotryptophan derivatives have been prepared by palladium-catalyzed aminocyclization-Heck-type coupling cascades starting from *o*-alkynylaniline derivatives and methyl α-aminoacrylate. Aryl, alkyl (primary, secondary, and tertiary), and alkenyl substituents have been introduced at the indole C-2 position. Further variations at the indole benzene ring, as well as the C-2-unsubstituted case, have all been demonstrated. In the case of C-2 aryl substitution, the preparation of the *o*-alkynylaniline substrate by Sonogashira coupling and the subsequent cyclization–coupling cascade have been performed in a one-pot protocol with a single catalyst. DFT calculations have revealed significant differences in the reaction profiles of these reactions relative to those involving methyl acrylate or methacrylate, and between the reactions of the free anilines and their corresponding carbamates. Those calculations suggest that the nature of the alkene and of the acid HX released in the HX/alkene exchange step that precedes C–C bond formation could be responsible for the experimentally observed differences in reaction efficiencies.

## Introduction

**Scheme 1:**
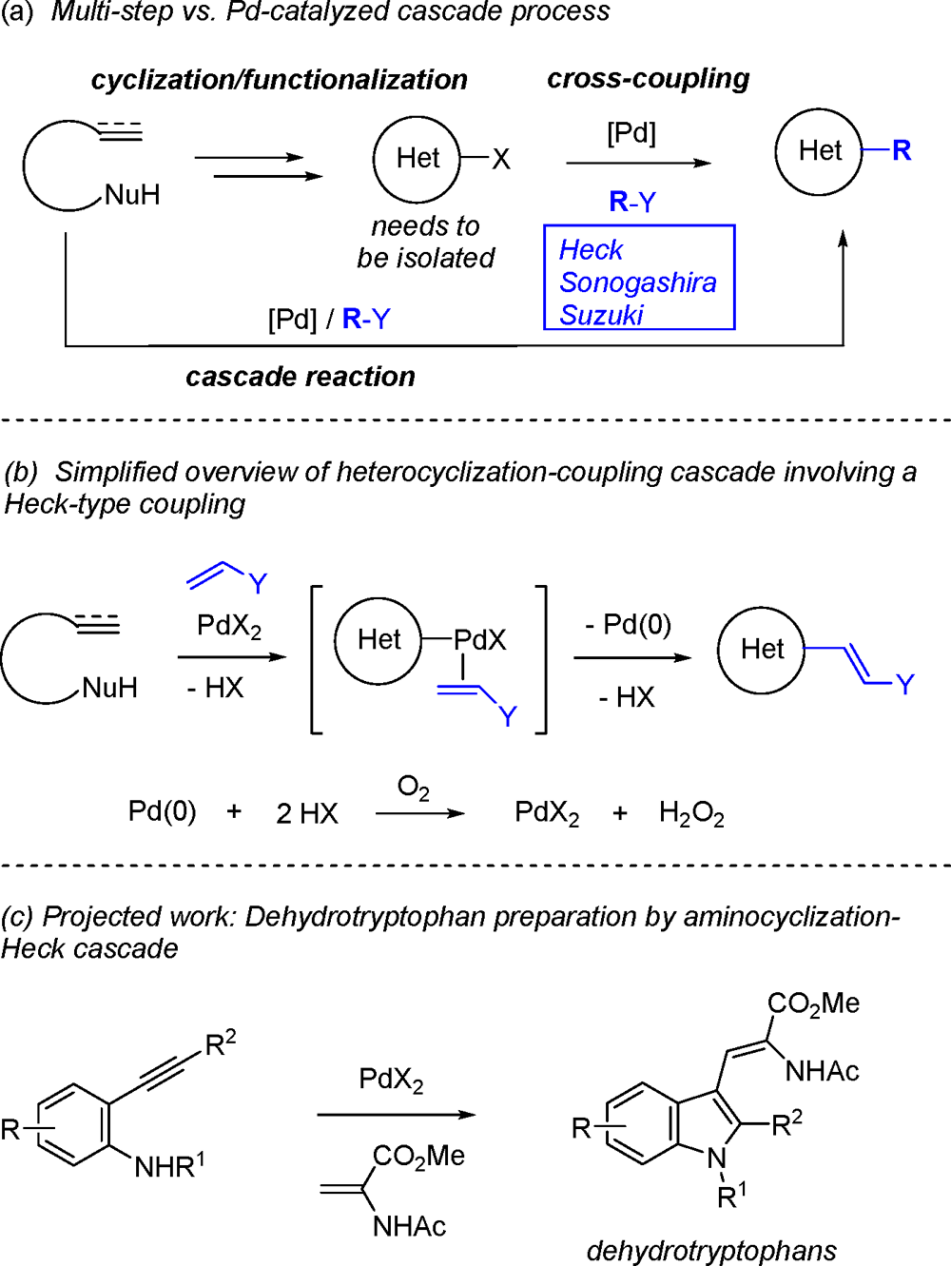
Palladium-catalyzed Oxidative Heterocyclization–Coupling Cascades and Application to the Preparation of Dehydrotrytophans

Palladium complexes are extensively utilized as catalysts in nucleophilic additions to unsaturated systems^1^ as well as in cross-coupling reactions.^2^ These two abilities have been combined in heterocyclization–coupling cascades involving nucleophile-tethered unsaturated systems and suitable coupling agents (Scheme 1a). In this manner, the cascade strategy has provided a practical access to heterocyclic systems with an appended alkene, alkyne, or aryl group, depending on whether heterocyclization is strategically designed to be followed by Heck-,^3−30^ Sonogashira-^31−41^ or Suzuki-type^7,42−46^ couplings, respectively.^47^ Compared with an alternative two-step protocol where a precursor is functionalized (typically as halide) before performing a palladium-catalyzed coupling,^2^ the use of those reaction cascades provides a more direct, expeditious and convergent method, as they use simpler acyclic substrates as starting materials and do not require the isolation of a cyclic functionalized intermediate.^47^ Taking the case of an alkene coupling agent as example, in mechanistic terms the cascade reaction can be thought of as proceeding via Pd(II)-promoted nucleopalladation and alkene complexation steps leading to a typical Heck reaction intermediate Pd(II) complex (Scheme 1b). However, relative to the conventional coupling reaction from a prefunctionalized precursor, the cascade reaction features two important distinctions. Thus, oxidative conditions are needed to regenerate the Pd(II) species that promote the heterocyclization from the Pd(0) generated during the coupling and, after intramolecular nucleopalladation, an acid molecule HX has to be released and exchanged for the coupling partner. As a result, in addition to the ligand ability of the alkene coupling partner, the acidity of HX becomes also an important consideration. Perhaps not surprisingly, cycloisomerization of the acyclic starting material, a well-established Pd-catalyzed transformation,^48^ has often been reported as a side-reaction, particularly in the case of alkyne-tethered substrates.^5,6,11,17,27,42^ Within the context of Scheme 1b, one particular field of application of cascade reactions has been the preparation of 3-alkenyl indoles by Pd-catalyzed aminocyclization-Heck-type coupling between 2-alkynylanilines and alkenes.^14,21,27^ It was envisaged that the application of this methodology to the particular use of an α-acetamidoacrylate as the alkene partner would be advantageous in the preparation of the expected dehydrotryptophan products (Scheme 1c). These compounds have attracted interest as precursors of the important family of tryptophan derivatives,^49−63^ and also because of their presence in natural and non-natural substances with potential use in therapeutic applications.^64−71^ Access to dehydrotryptophans has been gained, among other methodologies,^65^ via Heck reaction of preformed 3-haloindoles^72,73^ and α-acetamidoacrylate derivatives.^74−78^ In this contribution, we report a more direct access to dehydrotryptophans from acyclic 2-alkynylaniline and α-acetamidoacrylate substrates using a Pd-catalyzed aminocyclization–Heck-type-coupling cascade. Additionally, with a combination of experimental and computational data, we have inquired into the effects of the alkene, phosphine ligand, and the aniline precursor on the overall efficiency of the cascade coupling reaction, and on the competition between coupling and cycloisomerization.

## Results and Discussion

Initially, the reaction conditions previously developed for related reactions with acrylate esters^14^ were tested on methyl α-acetamidoacrylate (**3**) and representative *o*-alkynylaniline substrates **1** variable at the terminal alkynyl position (aryl or alkyl). The corresponding carbamates **2** were also tried since on occasion their use had proven more advantageous.^27^ Results are shown in Table 1. Thus, heating *o*-alkynylaniline **1a** derived from *p-*tolylacetylene with an excess (6 equiv) of alkene **3** in DMF in the presence of a catalytic amount of PdCl_2_ and KI (0.5 equiv) under an air atmosphere provided the expected dehydrotryptophan product **4a** in moderate yield (entry 1). Cycloisomerization of the starting *N*-unsubstituted alkynylaniline **1a** was found to be an important side-reaction leading to the formation of the 3-unsubstituted indole **6a**. The use of a phosphine ligand had been found beneficial in cases where cycloisomerization was a problem,^14^ and this was also the case here, as the use of PdCl_2_(PPh_3_)_2_ (entry 2) resulted in a considerable increase in the yield of **4a** (63%) and a much more favorable **4a**/**6a** ratio (4:1). Other phosphines were also tested but gave inferior overall results (entries 3–4). For example, with (*p-*MeOC_6_H_4_)_3_P a further increase of the **4a/6a** ratio was observed (perhaps pointing to a possible effect of the phosphine electron-donating ability) but at the expense of an overall lower yield of **4a** (entry 3). The use of Pd(OAc)_2_ in place of PdCl_2_ was also less effective (entry 5). Next, the reaction conditions of entry 2 were applied to alkynylanilines **1b** and **1c** with alkyl groups at the terminal alkynyl position, but the results were much less successful (entries 6 and 7). Finally, the reactions of carbamates **2** were tested (entries 8–10). Relative to the *N*-unsubstituted anilines **1**, carbamates **2** were less reactive, as indicated by longer reaction times (entries 8 and 9) and complete lack of reactivity in the case of the *t*-Bu-substituted substrate **2c** (entry 10). Nevertheless, when reactive enough, carbamates led to the best results in terms of both yield and selectivity (72% for **5a**, entry 8), as the formation of a 3-unsubstituted indole analogous to **6** was not observed in those cases. In the case of the alkyl-substituted substrate **2b** partial carbamate cleavage took place, resulting in the formation of the *N*-unsubstituted indole **4b**, in addition to the corresponding carbamate **5b**.^79^ In any case, the overall yield of cyclization–coupling (58%) was a substantial improvement over the result of entry 6.

**Table 1:**
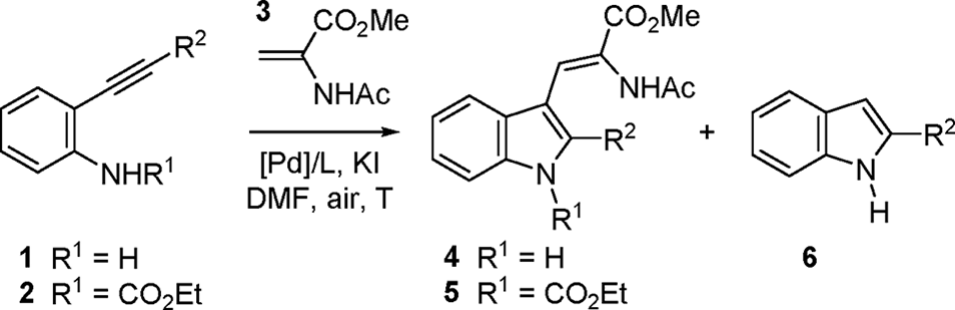
Survey of Reaction Conditions for Aminocyclization–Heck Coupling Between *o*-Alkynylaniline Derivatives **1** and **2** and Methyl α-Acetamidoacrylate (**3**)^a^

Entry	**1**	R^1^, R^2^	[Pd], L	*t* (h)	**4a**/**5a**	Yield of **4**/**5**^b^	**4**–**5**/**6** Ratio^c^
1	**1a**	H, *p*-tolyl	PdCl_2_	18	**4a**	39	1:1
2	**1a**	H, *p*-tolyl	PdCl_2_(PPh_3_)_2_	7	**4a**	63	4:1
3	**1a**	H, *p*-tolyl	PdCl_2_, (*p*-MeOC_6_H_4_)_3_P	18	**4a**	54	5:1
4	**1a**	H, *p*-tolyl	PdCl_2_, (*o*-furyl)_3_P	18	**4a**	33	2:1
5	**1a**	H, *p*-tolyl	Pd(OAc)_2_, PPh_3_	18	**4a**	36	2:1
6	**1b**	H, *n*-hexyl	PdCl_2_(PPh_3_)_2_	4	**4b**	20	d
7	**1c**	H, *tert*-butyl	PdCl_2_(PPh_3_)_2_	5	**4c**	43	d
8	**2a**	CO_2_Et, *p*-tolyl	PdCl_2_(PPh_3_)_2_	31	**5a**	72	*only***5a**
9	**2b**	CO_2_Et, *n*-hexyl	PdCl_2_(PPh_3_)_2_	31	**4b**, **5b**	18, 40	only **4b**, **5b**
10	**2c**	CO_2_Et, *tert*-butyl	PdCl_2_(PPh_3_)_2_	43	**–**	–	e

a Taken (in part) from Cruz, F. *Development of cascade reactions catalyzed by Palladium and their application to the synthesis of heterocycles*, Ph.D. Thesis, Universidade de Vigo, 2019. Reaction conditions: Alkynylaniline derivative **1** or **2**, alkene **3** (6 equiv), a Pd complex (5 mol %), a phosphine ligand (where appropriate, 10 mol %), and KI (0.5 equiv) were heated in DMF (10 mL/mmol) at 100 °C under air for the indicated time.

b Isolated yield (%).

c Measured in the crude reaction mixture.

d Not determined due to signal overlap.

e No reaction.

### Preparation of *N*-Unsubstituted Dehydrotryptophans

The extension of the cascade reaction to other carbamate substrates **2** was then studied. Additionally, the possibility of incorporating the preparation of the alkynyl carbamate substrate **2** into a one-pot protocol to perform a Sonogashira-cyclization–coupling sequence was sought. In this manner, the dehydrotryptophan derivatives would be prepared from 2-iodoarylcarbamates **7**, terminal alkynes **8**, and alkene **3** in a one-pot Pd-catalyzed sequence,^21^ using a single catalyst, without isolation of the Sonogashira intermediate **2** (Table 2). In practice, it was found that only alkynes with terminal aryl substituents took part effectively. The resulting aryl-substituted dehydrotryptophan products are endowed with particular interest, as the 2-phenylindole moiety is considered a privileged structure in medicinal chemistry.^80^ In the event, iodides **7**, arylalkynes **8**, and alkene **3** were reacted under typical Sonogashira conditions under argon, until complete consumption of the starting aryl iodide, whereupon air was allowed into the system and the mixture was heated to 120 °C (Table 2). Under these conditions, the expected dehydrotryptophan carbamate products **5** were formed, but it was noticed that, in line with previous observations (Table 1, entry 9), partial carbamate cleavage took place to yield also variable amounts of the *N*-unsubstituted dehydrotryptophans **4**.^79^ As a result, the experimental procedure was modified to include a carbamate-cleaving step. Accordingly, after a simple workup, the crude dehydrotryptophan product mixture (**5** and **4**) was treated with *tert-*butylamine^81^ to complete the conversion of **5** into **4**. This three-step procedure afforded dehydrotryptophan products **4** in good overall yields without the need for purification of intermediates. It was also found advantageous to use a polymer-bound PPh_3_.^82^ In this manner, a simple filtration facilitated the removal of catalyst residues that otherwise made chromatographic purification of some of the products difficult. As shown in Table 2, the procedure is effective for a variety of substituted arylalkynes **8** and 2-iodoarylcarbamates **7**. Electron-donating and electron-withdrawing substituents were tolerated in both sets of reactants, and the presence of bromine substituents at alternative positions of the aryl group of **7** did not interfere with the desired reaction sequence (entries 4 and 5).

**Table 2:**
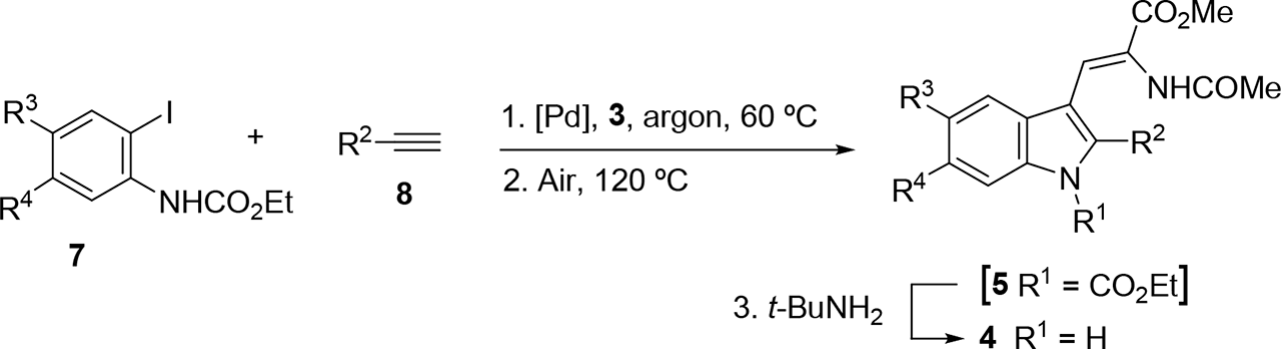
Preparation of 2-Aryldehydrotryptophans **4a**, **4d**–**n** (R^2^ = Ar) from 2-Iodoarylcarbamates **7**^a^

Entry	**7**	R^3^	R^4^	R^2^	*t* (h)^b^	**4**	Yield of **4**^c^
1	**7a**	H	H	*p-*tolyl	6, 31, 4	**4a**	62
2	**7a**	H	H	(*p-*MeO)C_6_H_4_	6, 30, 4	**4d**	73
3	**7a**	H	H	(*p-*CO_2_Me)C_6_H_4_	6, 40, 4	**4e**	59
4	**7b**	Br	H	*p-*tolyl	5, 41, 3	**4f**	68
5	**7c**	H	Br	*p-*tolyl	4, 40, 3	**4g**	62
6	**7d**	CO_2_Me	H	*p-*tolyl	4, 40, 3	**4h**	49
7	**7e**	OMe	H	*p-*tolyl	4, 40, 3	**4i**	55
8	**7a**	H	H	(*m-*Cl)C_6_H_4_	21, 48, 6	**4j**	58
9	**7a**	H	H	(*m-*F)C_6_H_4_	21, 48, 5	**4k**	50
10	**7a**	H	H	(*m-*OMe)C_6_H_4_	21, 48, 4	**4l**	59
11	**7a**	H	H	(3,4-diF)C_6_H_3_	21, 48, 5	**4m**	58
12	**7a**	H	H	3-thienyl	21, 48, 5	**4n**	51

a Taken (in part) from Cruz, F. *Development of cascade reactions catalyzed by Palladium and their application to the synthesis of heterocycles*, Ph. D. Thesis, Universidade de Vigo, 2019. Reaction conditions. Step 1: Iodide **7**, alkene **3** (6 equiv), alkyne **8** (2 equiv), PdCl_2_ (5 mol %), polymer-bound PPh_3_ (10 mol % of PPh_3_ relative to **7**), CuI (20 mol %), Et_3_N (4.5 equiv) in DMF (10 mL/mmol) at 60 °C under Ar. Step 2:120 °C under air. Step 3: *t-*BuNH_2_ (30 equiv), MeOH (21 mL/mmol), reflux.

b Reaction times for steps 1–3.

c Isolated yield (%).

**Scheme 2:**
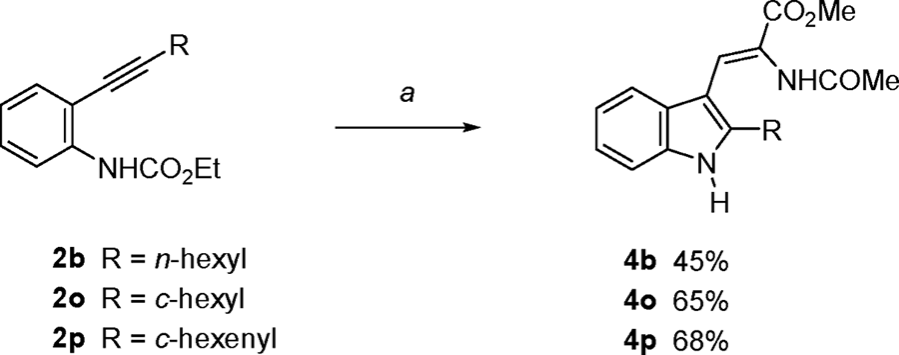
Preparation of 2-Alkyldehydrotryptophans from 2-Alkynylaniline Carbamates Reaction conditions: (*i*) alkene **3** (6 equiv), PdCl_2_(PPh_3_) _2_ (10 mol %), KI (0.5 equiv), DMF, air, 100 °C (**2b** and **2p**) or 120 °C (**2o**). (*ii*) *t*-BuNH_2_ (30 equiv), MeOH, 90 °C.

As indicated above, the one-pot Sonogashira-cyclization–coupling sequence did not provide satisfactory results when applied to alkyl-substituted terminal alkynes. Alternatively, a cyclization–coupling–carbamate-cleavage protocol was efficient with such alkynes (Scheme 2). Still, these alkynyl carbamates had a rather sluggish reactivity in the Pd-catalyzed process, resulting in long reaction times and recovery of some starting material. This prompted the use of a higher Pd load (10 mol %), and in the case of the *c*-hexyl-substituted substrate **2o**, a higher temperature (120 °C) was also needed for practical results. As shown in Scheme 2, both primary- and secondary-alkyl groups, as well as an alkenyl substituent, were successfully used to afford the corresponding *N*-unsubstituted dehydrotryptophans (**4b**, **4o**, and **4p**) after carbamate deprotection.

### Preparation of *N*-(PMB)dehydrotryptophans

To explore also the possibility of using C_sp3_-substituents at the aniline *N* atom, *p-*methoxybenzyl (PMB) dehydrotryptophans were targeted as representative of the interesting subclass of benzyl-substituted indoles^83−86^ (Table 3). In line with the results of Scheme 2, modifications of the reaction conditions were introduced in order to improve the performance of these aniline substrates. This implied increasing again the catalyst loading to 10 mol % and, for consistent results, also incorporating triphenylphosphine oxide (TPPO, 10 mol %) as an additive.^87^

**Table 3:**
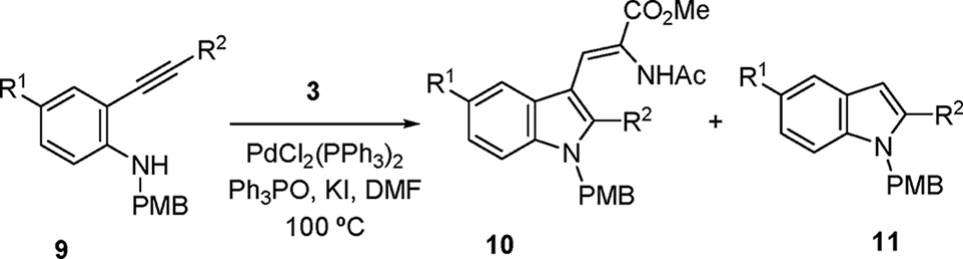
Preparation of 2-Alkyl- and 2-Alkenyldehydrotryptophans **10** from *N*-(PMB)-2-Alkynylanilines **9**^a^

Entry	**9**	R^1^	R^2^	*t* (h)	**10**	Yield^b^ of **10**
1	**9a**	H	*n-*hexyl	15	**10a**	40
2	**9b**	H	CH_2_OAc	5	**10b**	41
3	**9c**	H	*c-*hexyl	16	**10c**	50
4	**9d**	H	*tert-*butyl	24	**10d**^c^	82^c^
5	**9e**	H	C(Me)_2_CH_2_OTBDPS	20	**10e**	72
6	**9f**	H	C(Me)_2_OH	16	**10f**	41
7	**9g**	H	cyclohexenyl	23	**10g**	67
8	**9h**	Me	*tert*-butyl	18	**10h**	53
9	**9i**	CO_2_Me	*tert*-butyl	17	**10i**	48
10	**9j**	Me	cyclohexenyl	19	**10j**	50

a Taken (in part) from Cruz, F. *Development of cascade reactions catalyzed by Palladium and their application to the synthesis of heterocycles*, Ph.D. Thesis, Universidade de Vigo, 2019. Reaction conditions: *N*-PMB-2-alkynylaniline **9**, alkene **3** (6 equiv), PdCl_2_(PPh_3_)_2_ (10 mol %), TPPO (10 mol %), and KI (0.5 equiv) in DMF (10 mL/mmol) under air.

b Isolated yield (%).

c A 3-unsubstituted indole **11d** (R^1^ = H; R^2^ = *t-*Bu) was also obtained in 17% yield.

**Scheme 3:**
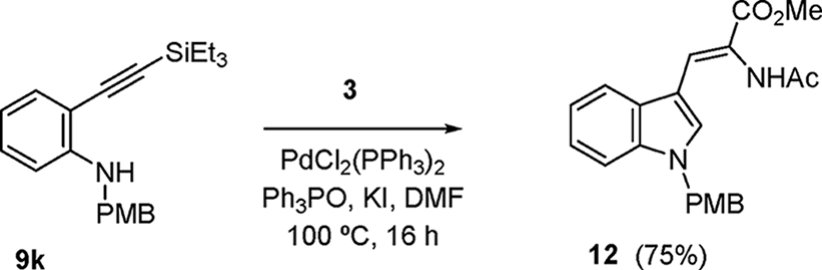
Preparation of a 2-Unsubstituted Dehydrotryptophan Derivative

As displayed in Table 3, the reaction has been applied to substrates **9** with primary-, secondary-, and tertiary-alkyl groups at the alkynyl terminal position. Additionally, both protected and unprotected carbinol-type substituents were tolerated at that position, leading to the expected 2-indolylmethanols^88^ in moderate yields (entries 2, 5–6), and the incorporation of a cyclohexenyl substituent was also successful (entries 7 and 10). The particular examples of entries 4–6 and 8–9 provide a precedent for the introduction of tertiary alkyl groups, prevalent in natural and otherwise interesting dehydrotryptophan derivarives.^64−67,69^ Furthermore, the reaction could also be applied to the triethylsilyl (TES)-substituted substrate **9k** (Scheme 3) to yield a C-2-unsubstituted dehydrotryptophan **12** as a result of an aminocyclization–alkenylation cascade and concomitant desilylation. The use of a silyl substituent as a H surrogate was prompted by unsuccessful attempts to use directly simple *o*-ethynylaniline featuring a terminal alkyne. In general, some cycloisomerization of the starting alkynylaniline, with formation of the uncoupled 3-unsubstituted indoles (**11**), was observed as a side reaction in the cases shown in Table 3. For example, the 2-(*tert-*butyl) derivative **11d** (R^1^ = H; R^2^ = *t-*Bu) was isolated in 17% yield (entry 4, Table 3), and the formation of analogous products could also be inferred in the remaining entries of Table 3 (and Scheme 3) from inspection of the ^1^H NMR of the crude products (singlet at δ 6.3–6.8), although these products were not further characterized.

In all the cases under study (Tables 1–3 and Schemes 2–3) the exocyclic trisubstituted double bond of dehydrotryptophans **4**, **10**, and **12** was generated with high stereoselectivity, as only one geometric isomer was isolated. The configuration was determined to be *Z* in product **10c** by X-ray analysis (see Figure S1 in Supporting Information), and the same geometry was assigned by analogy to the remaining products. This result is in line with previous literature examples where the formation of the (*Z*)-isomers of dehydrotryptophans was also reported in Heck-type reactions between indole and α-aminoacrylate derivatives.^58,74,76,78^

### Formation of Cycloisomerization Products

Relative to the related heterocyclization–coupling reactions of 2-alkynylanilines with *n*-butyl acrylate and methyl methacrylate (α-unsubstituted- and α-Me-substituted analogs, respectively, of acrylate **3**),^14^ the α-acetamidoacrylate reactions appear to be less effective, as indicated by their often lower isolated yields and higher incidence of cycloisomerization products (the main observed side reaction for *N*-unsubstituted and *N*-PMB-substituted anilines; see also Table 4 below), although that type of product was not observed in the carbamate series (Tables 1–2 and Scheme 2). The relative importance of that competing cycloisomerization reaction is also strongly dependent on the presence or absence of PPh_3_, as seen in Table 1. In order to have a more precise picture of the alkene- and phosphine-dependence of the coupling/cycloisomerization ratio, we have run some control experiments that have enabled comparisons, under the same reaction conditions, between α-acetamidoacrylate (**3**), *n*-butyl acrylate, and methyl methacrylate. These results are displayed in Table 4, where entries 1, 2, and 7 have been taken from Table 1.

**Table 4:**
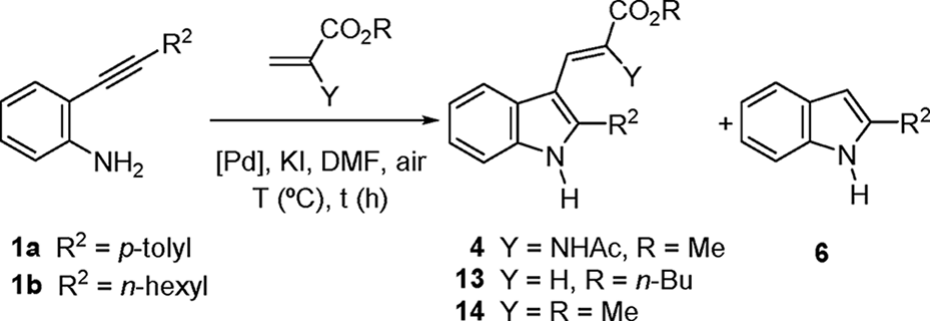
Effect of the Alkene and PPh_3_ on Yields and Coupling/Cycloisomerization Ratios^a^

entry	Y	R^2^	[Pd]	*T* (° C)	*t* (h)	**4**, **13**–**14**	Yield of **4**, **13**–**14**^b^	**4** or **13**–**14**/**6** Ratio^c^
1	NHAc	*p*-tolyl	PdCl_2_	100	18	**4a**	39	1:1
2	NHAc	*p*-tolyl	PdCl_2_(PPh_3_)_2_	100	7	**4a**	63	4:1
3	H	*p*-tolyl	PdCl_2_	80	20	**13a**^e^	56	1.4:1^d^^,^^e^
4	H	*p*-tolyl	PdCl_2_	100	20	**13a**^e^	85	only **13a**^e^
5	H	*p*-tolyl	PdCl_2_(PPh_3_)_2_	80	19	**13a**^e^	90	only **13a**^e^
6	H	*p*-tolyl	PdCl_2_(PPh_3_)_2_	100	18	**13a**^e^	91	only **13a**^e^
7	NHAc	*n*-hexyl	PdCl_2_(PPh_3_)_2_	100	4	**4b**	20	f
8	H	*n*-hexyl	PdCl_2_(PPh_3_)_2_	100	18	**13b**^e^	52	only **13b**^e^
9	Me	*p*-tolyl	PdCl_2_(PPh_3_)_2_	60	20	**14**^e^	67	only **14**^e^
10^g^	NHAc	*p*-tolyl	PdCl_2_(PPh_3_)_2_	100	20	g	g	
11^g^	NHAc	*p*-tolyl	PdCl_2_	100	20	g	g	
12^h^	NHAc	*p*-tolyl		100	4	h	h	

a Reaction conditions: Unless otherwise indicated, **1a** or **1b**, a Pd complex (5 mol %), KI (0.5 equiv) and alkene (6 equiv) in DMF under air atmosphere.

b Isolated yield (%).

c Measured in the crude reaction mixture.

d Ratio of isolated yields.

e Reference (14).

f Not determined due to signal overlap.

g Reaction run in the absence of alkene. The cycloisomerization product **6a** was obtained in 28% yield (entry 10) or 15% yield (entry 11).

h Experiment run in the absence of alkene and Pd catalyst: No reaction.

The results in Table 4 confirm that, under comparable conditions, cycloisomerization is a more important competing side reaction in the α-acetamidoacrylate case relative to the other acrylates. Notably, the disubstituted alkene of entry 9 reacts without observable formation of cycloisomerization products. It is also apparent that the effect of the phosphine ligand PPh_3_ on cycloisomerization is not exclusive of α-acetamidoacrylates. Thus, while cycloisomerization is subdued in the α-acetamidoacrylate reaction in the presence of PPh_3_ (entries 1 and 2), it appears to be completely suppressed with the simpler acrylates under similar reaction conditions (see entry 3 vs entries 5 and 9). Control reactions *in the absence of alkene* showed that the 3-unsubstituted indole **6a** was formed (albeit in a low 15–28% yield)^89^ (Table 4, entries 10–11) but was not detected when the Pd catalyst was also omitted (Table 4, entry 12). This indicated that, at least in part, cycloisomerization is a Pd-catalyzed reaction.

### Computational Studies

**Scheme 4:**
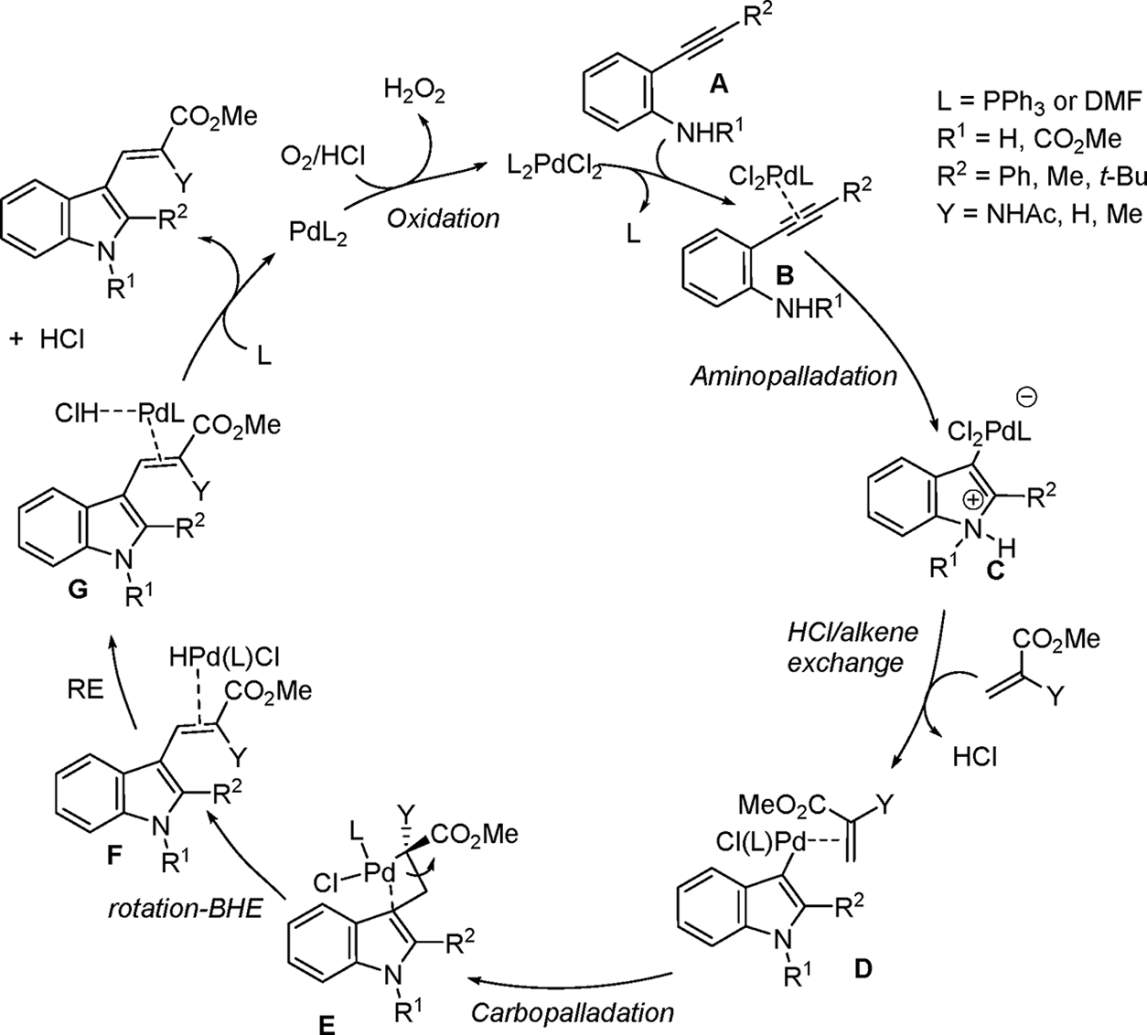
Expected Catalytic Cycle

In order to gain further insights into the above-mentioned differences in reactivity, we have studied computationally the aminocyclization–coupling reactions using DFT methods. We have evaluated the effect of the alkene and PPh_3_ ligand on the energetics of the reaction pathway, as well as the differences between *N*-unsubstituted 2-alkynylanilines and their corresponding carbamates. The expected catalytic cycle is shown in Scheme 4, where product formation is the result of four major steps, namely cyclization (intramolecular aminopalladation), HCl/alkene exchange, carbopalladation (insertion), and β-hydride elimination (BHE). To complete the catalytic cycle, oxidation of the Pd(0) released by reductive elimination (RE) of HCl after BHE regenerates the Pd(II) species needed to activate the C–C triple bond and reinitiate the cycle.

**Figure 1:**
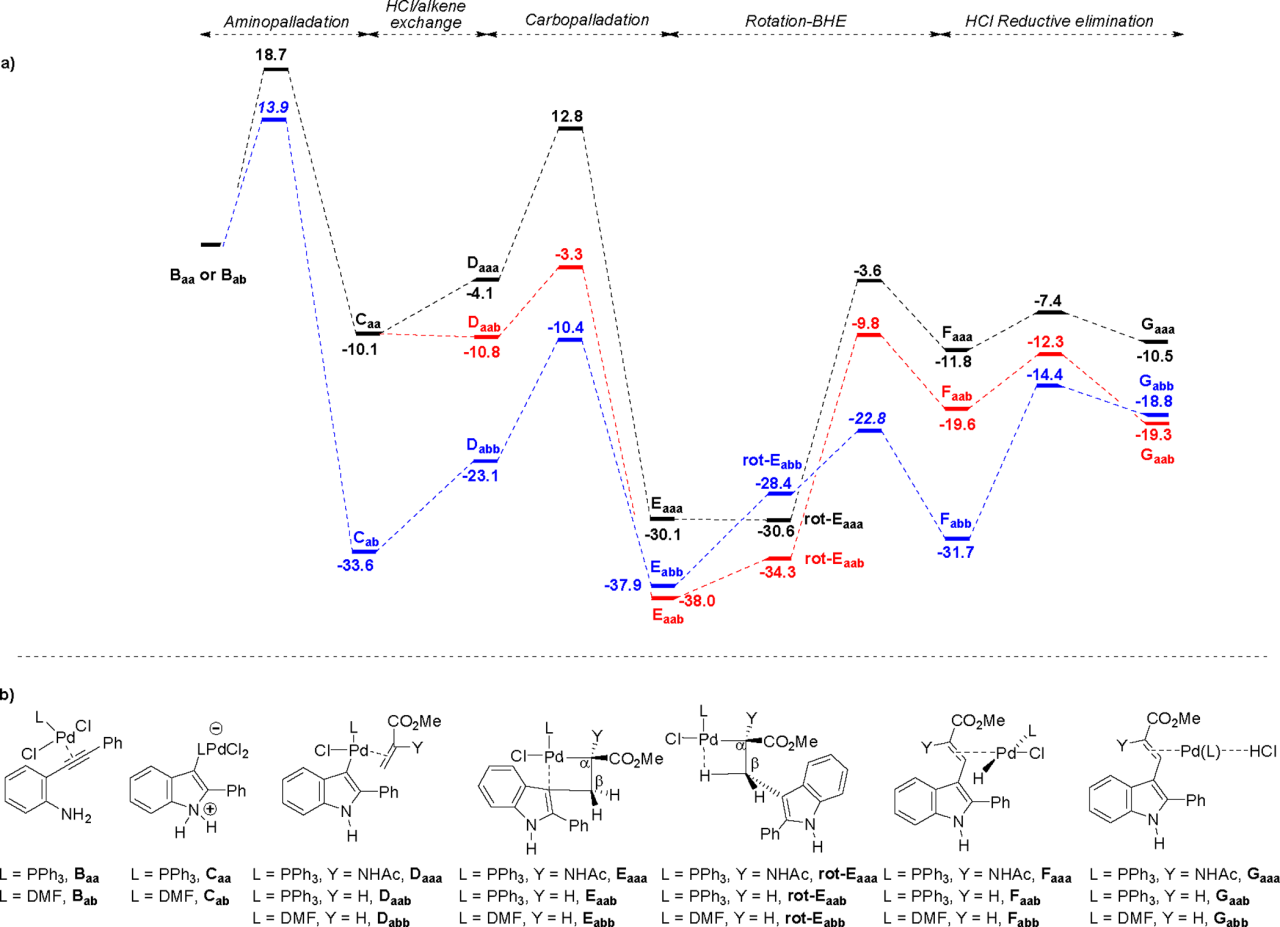
(a) Reaction profile starting from complexes **B** derived from **A**_**a**_ (R^1^ = H, R^2^ = Ph in Scheme 4) and methyl α-acetamidoacrylate (Y = NHAc) or methyl acrylate (Y = H) in a reaction promoted by PdCl_2_/L (L = PPh_3_ or DMF) [energy values in kcal/mol; WB97XD/def2SVPP_LANL2DZ(SMD, DMF)//B97XD/def2TZVP (SMD, DMF)]. (b) Ground state structures of intermediates involved in the reaction profile.

**Figure 2:**
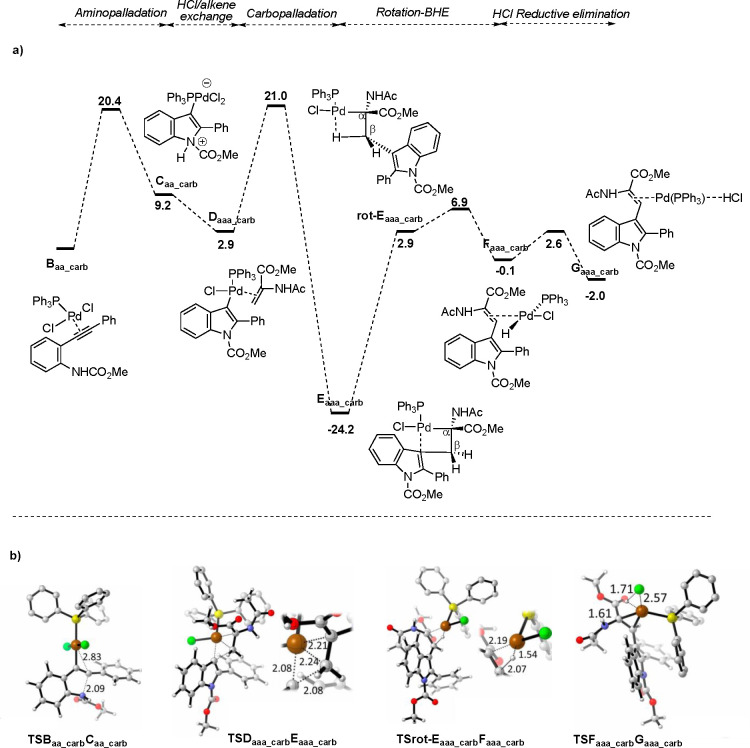
(a) Reaction profile starting from complexes **B** derived from **A**_**a-carb**_ (R^1^ = CO_2_Me, R^2^ = Ph, L = PPh_3_ in Scheme 4) and methyl α-acetamidoacrylate in a reaction promoted by PdCl_2_(PPh_3_)_2_ [energy values in kcal/mol; WB97XD/def2SVPP_LANL2DZ(SMD, DMF)//B97XD/def2TZVP (SMD, DMF)]. (b) 3-D structures of TSs, as representative of those involved in the different series.

According to this general scheme, we have compared the reaction profiles of cyclization–coupling cascades involving palladium complexes **B** originating from *o*-alkynylaniline derivatives **A**, variable at the nitrogen and alkynyl substituents (R^1^ and R^2^, respectively), and acrylate esters where variations were introduced at C_α_ (substituent Y). For reactions run in the absence of PPh_3_, the vacant position at Pd has been filled with a molecule of solvent (DMF).^90^ A summary of results is displayed in Table 5, where the energy differences (Δ*G*, kcal/mol) between species (intermediates and transition states) have been collected for cyclization, HCl/alkene exchange, insertion, BHE, and RE steps starting from complex **B**. Full energy profiles are displayed in Figures 1–2 and S3 (see Supporting Information).

**Table 5:**
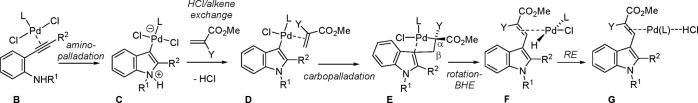
Energy Differences [in kcal/mol; WB97XD/def2SVPP_LANL2DZ(SMD, DMF)//B97XD/def2TZVP (SMD, DMF)] for the Stepwise Transformation of **B** to **G**

				*Aminopalladation*		*HCl/alkene exchange*	*Carbopalladation*			*Rotation-BHE*		*HCl RE*	
**entry**	**R**^**1**^, **R**^**2**^	**L**	**Y**	**Δ*****G***^**#**^_**B–C**_	**Δ*****G*****°**_**B–C**_	**Δ*****G*****°**_**C–D**_	**Δ*****G***^**#**^_**D–E**_	**Δ*****G*****°**_**D–E**_	**Δ*****G***^**#**^_**C–E**_	**Δ*****G***^**#**^_**E–F**_	**Δ*****G*****°**_**E–F**_	**Δ*****G***^**#**^_**F–G**_	**Δ*****G*****°**_**F–G**_
1	H, Ph	PPh_3_	H	18.7	–10.1	–0.7	7.5	–27.2	6.8	28.2	18.4	7.3	0.3
2	H, Ph	DMF	H	13.9	–33.6	10.5	12.7	–14.8	23.2	15.2	6.2	17.3	12.9
3	H, Ph	PPh_3_	Me	18.7	–10.1	2.2	6.7	–23.0	8.9				
4	H, Ph	PPh_3_	NHAc	18.7	–10.1	6.0	16.9	–26.0	22.9	26.5^a^	18.3	4.4	1.3
5	H, Me	PPh_3_	NHAc	19.1	–9.0	7.7	16.6	–32.8	24.3	29.9	24.2	4.9	–2.9
6	H, *t*-Bu	PPh_3_	NHAc	17.8	–8.2	10.0	14.9	–32.6	24.9	29.1	24.5	3.5	–1.8
7	CO_2_Me, Ph	PPh_3_	NHAc	20.4	9.2	–6.3	18.1	–27.1	10.8^b^	31.1	24.1	2.7	–1.9
8	CO_2_Me, *t*-Bu	PPh_3_	NHAc	21.8	12.5	2.9	16.3	–28.6	19.2	30.2	26.2	2.1	–4.9

a 27.0 kcal/mol from the lowest energy intermediate **E**_**rot**_ (see Figure 1).

b 18.1 kcal/mol from **D** (see Figure 2).

Formation of palladium complexes **B** from *o*-alkynylaniline derivatives **A** and PdCl_2_(PPh_3_)_2_ is an endergonic process in all cases (15.6–16.8 kcal/mol; see also Tables S2–S4 in Supporting Information), and almost insensitive to substituent effects. However, it is noticed that this estimate is probably realistic only in the early stages of the reaction, when PPh_3_ has to be displaced from Pd by the *o*-alkynylaniline **A**. Indeed, under the experimental oxidative conditions, some PPh_3_ is oxidized to the corresponding triphenylphosphine oxide (TPPO),^91,92^ and this should make complexation of the *o*-alkynylaniline **A** more favorable, as TPPO is a weaker Pd-ligand than PPh_3_. In any event, the activated triple bond and internal amino group of **B** then engage in a 5-*endo-dig* cyclization leading to zwitterions **C** (where the palladium *trans* geometry is maintained) with moderate activation energies, which are in the range 17.8–21.8 kcal/mol, with the exception of the phosphine-free cyclization, which has a significantly lower cyclization barrier (13.9 kcal/mol). It is interesting that this step appears to be insensitive to the steric bulk of the *t*-Bu group in the *N*-unsubstituted series. On the other hand, carbamates have somewhat higher barriers (by 1.7 and 4 kcal/mol, for R^2^ = Ph and *t*-Bu, respectively) than the corresponding amines. However, a much more substantial difference is observed in the reaction energies, as the cyclization of the carbamate substrates (**B**, R^1^ = CO_2_Me) proceeds uphill (by 9.2–12.5 kcal/mol) whereas for the *N*-unsubstituted substrates (**B**, R^1^ = H) the cyclizations are all exergonic (by 8.2–33.6 kcal/mol). Again, differences are noticed between the PPh_3_-ligated complexes (**B**, L = PPh_3_), which maintain cyclization exergonicity within a narrow range (8.2–10.1 kcal/mol), and the much more favorable phosphine-free case (**B**, L = DMF; Δ*G*°_B–C_ = −33.6 kcal/mol). Those calculated differences between *N*-unsubstituted anilines and their carbamates are understood as a consequence of the effect of the electron-withdrawing group on *N* that, relative to the unsubstituted aniline (**B**, R^1^ = H), renders the carbamate (**B**, R^1^ = CO_2_Me) a less reactive nucleophile and reduces the stability of the zwitterionic cyclization product **C** (R^1^ = CO_2_Me). In any case, from zwitterions **C** the loss of HCl is accompanied by the incorporation of an alkene molecule to arrive at intermediates **D**, where the alkene ligand occupies the position of the released chloride anion. Complexes **D** are the starting point for the C–C-bond forming carbopalladation step eventually leading to the coupling product. Some interesting information emerges from the HCl/alkene exchange data. Thus, for reactions catalyzed by PdCl_2_(PPh_3_)_2_, methyl acrylate (Y = H, cf. entry 1, Table 5) and methyl methacrylate (Y = Me, cf. entry 3) have a more favorable HCl/alkene exchange energy than the corresponding methyl α-acetamidoacrylate (Y = NHAc, cf. entries 4–6), indicating that the former have a higher affinity for that particular palladium moiety. This exchange is also more favorable for carbamates, relative to the *N*-unsubstituted anilines; for example, for the carbamate of entry 7 (R^2^ = Ph, Y = NHAc) HCl/alkene exchange is exergonic (by 6.3 kcal/mol), whereas in the corresponding aniline it is endergonic by approximately the same amount (6.0 kcal/mol, cf. entry 4). It is reasonable to expect that, in this case, the same substituent effects discussed above would make the carbamate zwitterion **C** (R^1^ = CO_2_Me) more acidic, leading to a more favorable HCl/alkene exchange. A final note on this step has to do with the phosphine-free complex (**C**, L = DMF, cf. entry 2), where the exchange is found to be much less favorable (by 11.2 kcal/mol) than in the corresponding PPh_3_-ligated case (**C**, L = PPh_3_, cf. entry 1); however, these latter data would be difficult to interpret in the foregoing context of substituent effects because both the acidity of **C** and the affinity of the HCl-free Pd complex for the alkene are affected by the change at L in this case.

Next, from complexes **D** carbopalladation leads to intermediates **E**, which display a *trans*-relationship between the Cl–Pd and newly formed σ–Pd-C_α_ bonds, while the 5-membered indole π-system occupies the remaining vacant position at Pd. This step is predicted to be very exergonic in all cases and irreversible, as a result. However, differences are clearly observed in the activation energies. Thus, both methyl acrylate and methyl methacrylate are predicted to have substantially lower insertion barriers when compared with methyl α-acetamidoacrylate (cf. entries 1 and 3 vs 4–8). From the carbopalladation product **E**, an energy sink, the reaction proceeds to the BHE TS by detachment of the indole π-ligand from palladium and C_α_–C_β_ rotation to first reach **rot-E** (Figures 1–2 and S3) and allow the interaction between palladium and one of the C–H bonds at the original alkene’s β-position. The overall activation energy for BHE leading to the experimentally obtained *Z*-isomer is relatively high (26.5–31.1 kcal/mol), again with the exception of the PPh_3_-free complex (L = DMF, Δ*G*^#^_E-F_ = 15.2 kcal/mol, entry 2), whereas the barrier for the unobserved *E*-isomer is 7 kcal/mol higher in the calculated case (R^1^ = H; R^2^ = Ph; L = PPh_3_; Y = NHAc) (see Table S5 in Supporting Information). The BHE step is reversible in all cases, and in this scenario, product release and completion of the catalytic cycle require a relatively facile and irreversible regeneration of the starting PdX_2_ catalyst.^93^ According to the literature,^93,94^ this could take place from HPdX by initial rate-determining H atom abstraction by O_2_ (HAA pathway) or an alternative reductive elimination of HX, followed by facile oxidation by O_2_ of the resulting Pd(0) species (HXRE pathway), leading in both cases to the formation of a Pd(II) hydroperoxide intermediate. While the actual oxidation mechanism has not been subject to study in our particular system, in the context of the more generally considered HXRE mechanism^93^ we have determined that reductive elimination of HCl from complexes **F** (L = PPh_3_), where the H and Cl atoms already have the required *cis* arrangement, has indeed a relatively low activation energy (2.7–7.3 kcal/mol, Table 5, Figures 1–2 and S3). Furthermore, the subsequent Pd(0) oxidation is expected to be facilitated by the participation of molecular iodine formed from the KI additive under the oxidizing (air) reaction conditions.^14,95^

The analysis of the data in Table 5, Figures 1–2 and S3 (Supporting Information) reveals that the carbamate reactions transit through a higher energy pathway than the *N*-unsubstituted cases, and this would be in line with the experimental observation of a longer reaction time required for substrate **2a** relative to **1a** (Table 1). Nevertheless, the carbamate reactions are higher yielding than those of their corresponding amines, possibly because the former benefit from an exergonic HCl/alkene exchange (with the exception of the *t*-Bu-substituted case; see below) between the zwitterion **C** and the key insertion precursor **D**. As a result, intermediate **D** (that leads irreversibly to **E**) is much more populated than **C**, while the insertion barrier is kept lower than that of the *N*-unsubstituted aniline (18.1 kcal/mol from **D** vs 22.9 kcal/mol from **C**, entries 7 and 4, respectively; Table 5). On the other hand, the lack of reactivity of the *t*-Bu-substituted carbamate **2c** (entry 10, Table 1) could be ascribed to the endergonicity of both cyclization and HCl/alkene exchange steps, resulting in a total insertion barrier of 31.7 kcal/mol starting from **B**. This, together with a similarly high barrier for BHE (30.2 kcal/mol, entry 8, Table 1) would lead to a slow reaction.

Further examination of Tables 4–5 also indicates that cycloisomerization of the starting alkynylaniline derivative, leading to products **6**, is absent in cases with low insertion energy and/or favorable HCl/alkene exchange energy. As suggested in the literature,^96^ protodepalladation is a possible cycloisomerization mechanism and, in the context of the catalytic cycle of Scheme 4, this could be taking place from an intermediate of an undetermined structure, originating from **C** or **D**.^97^ In this scenario, reactions of *n*-butyl acrylate or methyl methacrylate, with low insertion activation energies, would be expected to compete more efficiently with that cycloisomerization pathway than those of α-acetamidoacrylates, where insertion has a much higher barrier.^98^ As for the effect of PPh_3_, it is noticed that protodepalladation would be dependent on the availability of HCl (released upon formation of **D** and also after BHE), which is in turn limited by its consumption during the final Pd(0) oxidation (Scheme 4), and possibly by interaction with TPPO produced as a result of PPh_3_ air oxidation.^91,92^ In fact, the formation of the hydrochloride HClTPPO^99^ from TPPO and HCl is a very favorable process, calculated to release 13.7 kcal/mol. As a result, TPPO could have a regulatory effect on the available amount of HCl. Interestingly, upon following the reaction of **1a** and **3** by HPLC-MS, it was found that the ratio **4a**/**6a** increased as the reaction progressed (and presumably more HClTPPO was formed).^92^ This possible regulatory role of TPPO could explain its beneficial effect in the reactions of *N*-PMB derivatives (Table 3), as indicated above, and perhaps also the higher incidence of cycloisomerization in the PPh_3_-free reactions (entries 1 and 3 in Tables 1 and 4, respectively), where that regulatory effect is absent.

## Conclusions

The preparation of structurally diverse dehydrotryptophans has been developed using palladium-catalyzed oxidative aminocyclization–coupling cascade reactions involving 2-alkynylaniline derivatives and methyl α-acetamidoacrylate. In this direct manner, moderate-to-high yields of products are realized and, relative to alternative strategies, the isolation and purification of intermediates is minimized. The method is effective for the preparation of the indolyl *N*-unsubstituted dehydrotryptophans (through the corresponding carbamates), as well as for *N*-PMB derivatives. Aryl, alkenyl, and alkyl (primary, secondary, and tertiary) substituents have all been incorporated at the indole C-2 position, and the presence of both electron-donating and electron-withdrawing groups at the aniline benzene ring has been shown to be well tolerated. DFT calculations have been performed on these and related reactions using model methyl acrylates. The computed data indicate that the presence of the amino substituent on the alkene tends to disfavor both HCl/alkene exchange and alkene insertion steps, whereas the presence of PPh_3_ and the use of a carbamate of the alkynylaniline have the opposite effect. These findings are in line with the experimentally observed higher incidence of competing cycloisomerization in those cases where HCl/alkene exchange and alkene insertion are disfavored (use of α-acetamidoacrylates, absence of PPh_3_, and use of *N*-unsubstituted alkynylanilines).

### Computational Details

All calculations were carried out using the Gaussian 09 program package^100^ and ωB97XD functional developed by Chai and Head-Gordon.^101^ The def2SVPP basis set developed by Ahlrichs and co-workers was used for nonmetals and LANL2DZ for Pd.^102^ Single-point energy calculations were carried out with a triple ζ basis (def2TZVPP) for all atoms.^103^ The SMD model^104^ was used to include the solvent (DMF) in both optimizations and single-point calculations. The nature of the different saddle points were determined by the number of imaginary frequencies, and these structures were connected via IRC. All 3D representations were created using the Chemcraft software.^105^

## Experimental Section

### General Information

THF and MeOH were dried using a Puresolv solvent purification system. Commercial DMF (≥99.8%) was kept over 4 Å MS. A polymer-bound PPh_3_ (100–200 mesh; 1.6 mmol/g PPh_3_ loading; diphenylphosphino polystyrene cross-linked with divinylbenzene) was purchased from Aldrich. All other reagents were commercial compounds of the highest purity available. For reactions that require heating we used the Heat-On block system of radleys. New compounds were fully characterized by their ^1^H and ^13^C NMR, IR, and HRMS spectral properties. Unless otherwise indicated, routine NMR spectra were obtained at 25 °C on a Bruker ARX-400 spectrometer (400.16 MHz for ^1^H and 100.62 MHz for ^13^C) using CDCl_3_, acetone-d_6_, CD_3_OD, and DMSO-*d*_6_ as solvents and internal reference (CDCl_3_ δ 7.26 for ^1^H and δ 77.0 for ^13^C, acetone-d_6_ δ 2.05 for ^1^H and δ 29.84 for ^13^C, CD_3_OD δ 3.31 for ^1^H and δ 49.0 for ^13^C, DMSO-*d*_6_ δ 2.50 for ^1^H and δ 39.5 for ^13^C). Chemical shifts (δ) are reported in ppm, and coupling constants (*J*) are given in hertz (Hz). The proton spectra are reported as follows: multiplicity, coupling constant *J*, number of protons. The DEPT sequence was routinely used for ^13^C multiplicity assignment. Additionally, a combination of COSY, HSQC, and HMBC NMR experiments were used for structural assignments. Infrared spectra (IR) data were obtained from a thin film deposited onto a NaCl glass and were measured on a Jasco FT/IR 4100 in the interval between 4000 and 600 cm^–1^ with a 4 cm^–1^ resolution; data include only characteristic absorptions. Electrospray ionization (ESI) mass spectra were obtained on a micrOTOF focus mass spectrometer (Bruker Daltonics) using an ApolloII (ESI) source with a voltage of 4500 V applied to the capillary. Electron impact (EI) mass spectra were obtained on a Hewlett-Packard HP59970 instrument operating at 70 eV. For UPLC-QTOF, chromatographic separation was done with an Acquity UPLC BEH C18 1.7 μm, 50 mm × 2.1 mm column, and H_2_O/HCO_2_H (99.9:0.1, v/v) or MeOH/HCO_2_H (99.9:0.1, v/v) as eluent mixture; the ionization source was electrospray in positive mode (ESI^+^) with a voltage of 15 V; the range of masses in acquisition was 50–1200 u in SCAN mode. Flash-column chromatography was carried out in an automated system, using silica gel (230–400 mesh), cyano-functionalized silica gel (CN-silica gel, 20 to 40 μm particle size, spherical) or C18-derivatized silica gel (C18-silica, 40 to 63 μm, particle size). Analytical thin layer chromatography (TLC) was performed on aluminum plates with Merck Kieselgel 60F_254_ and visualized by UV irradiation (254 nm). Melting points were measured in a Büchi B-540 apparatus in open capillary tubes.

### X-ray Crystallographic Analysis of **10c**

The crystals were grown in hexane/EtOAc by warming for 2 days at 5–7 °C. Crystallographic data were collected on a Bruker Smart 1000 CCD diffractometer at CACTI (Universidade de Vigo) at 20 °C using graphite monochromated Mo Kα radiation (λ = 0.71073 Å), and were corrected for Lorentz and polarization effects. The software SMART1 was used for collecting frames of data, indexing reflections, and the determination of lattice parameters and SAINT2 for integration of intensity of reflections and scaling and SADABS3 for empirical absorption correction. The structure (Figure 1) was solved by direct methods using the program SHELXS97.4. Non-hydrogen atoms were refined with anisotropic thermal parameters by full-matrix least-squares calculations on F2 using the program SHELXL97.5. Hydrogen atoms were inserted at calculated positions and constrained with isotropic thermal parameters. The crystallographic data of **10c** were deposited in the Cambridge Crystallographic Data Centre with the deposition number CCDC 1939395.

### Carbamate Preparation from *o*-Iodoanilines. General Procedure

To a suspension of K_2_CO_3_ (0.36 g, 2.61 mmol) and the appropriate 2-iodoaniline (2.01 mmol) in THF (10 mL) at 0 °C was added dropwise ethyl chloroformate (0.23 mL, 2.41 mmol), and the mixture was stirred either at room temperature or at 80 °C for 12–22 h. The reaction mixture was poured over H_2_O (10 mL), and the mixture was extracted with CH_2_Cl_2_ (3×). The combined organic layers were washed with a saturated aqueous solution of NaHCO_3_ and dried (Na_2_SO_4_). The solvent was evaporated, and the residue was purified by flash-column chromatography (silica gel; solvent A, hexane; solvent B, EtOAc; gradient from 100:0 to 80:20 A/B) to afford the product.

#### Ethyl (4-Bromo-2-iodophenyl)carbamate (**7b**)^27,106^

Following the general procedure for carbamate formation described above, the reaction of 4-bromo-2-iodoaniline (0.50 g, 1.68 mmol), ethyl chloroformate (0.19 mL, 2.01 mmol), and K_2_CO_3_ (0.30 g, 2.18 mmol) in THF (8.4 mL) at 80 °C for 19 h provided 0.55 g (88%) **7b** as a white solid. Mp: 105–106 °C (hexane/EtOAc).^106^

#### Ethyl (5-Bromo-2-iodophenyl)carbamate (**7c**).^106^

To a cooled (0 °C) solution of 5-bromo-2-iodoaniline (1.5 g, 5.03 mmol) in pyridine (6.6 mL) was added ethyl chloroformate (0.67 mL, 7.05 mmol). The mixture was warmed up to 25 °C and stirred for 20 h. The reaction was poured into a mixture of EtOAc and brine (100 mL, 1:1 v/v). The layers were separated, the aqueous layer was extracted with EtOAc (3×), and the combined organic layers were washed successively with a saturated aqueous solution of CuSO_4_ (2×) and brine (2×) and dried (Na_2_SO_4_). The solvent was evaporated, and the residue was purified by flash-column chromatography (silica gel, 95:5 hexane/EtOAc) to afford 1.80 g (97%) of **7c** as a white solid. Mp 108–109 °C (hexane/EtOAc). ^1^H NMR (400.16 MHz, CDCl_3_): δ 8.30 (d, *J* = 2.4 Hz, 1H), 7.58 (d, *J* = 8.4 Hz, 1H), 6.93 (dd, *J* = 8.3, 2.5 Hz, 2H), 4.26 (q, *J* = 7.1 Hz, 2H), 1.34 (t, *J* = 7.1 Hz, 3H) ppm.^106^^13^C{^1^H} NMR (100.62 MHz, CDCl_3_): δ 153.2, 139.8, 139.7, 128.0, 123.5, 122.9, 86.3, 62.0, 14.6 ppm. FTIR (NaCl): ν 3291 (s, N–H), 1690 (s, C=O), 1565 (s), 1518 (s), 1075 (s, C–Br) cm^–1^.

#### Methyl 4-[(Ethoxycarbonyl)amino]-3-iodobenzoate (**7d**).^107^

Following the general procedure for carbamate formation described above the reaction of methyl 4-amino-3-iodobenzoate (0.78 g, 2.82 mmol), ethyl chloroformate (0.32 mL, 3.38 mmol), and K_2_CO_3_ (0.51 g, 3.66 mmol) in THF (14.1 mL) at 80 °C for 12 h provided 0.98 g (69%) of **7d** as a white solid. Mp: 113–114 °C (hexane/EtOAc). ^1^H NMR (400.16 MHz, CDCl_3_): δ 8.40 (d, *J* = 1.9 Hz, 1H), 8.18 (d, *J* = 8.7 Hz, 1H), 7.96 (dd, *J* = 8.7, 2.0 Hz, 1H), 7.16 (s, 1H), 4.25 (q, *J* = 7.1 Hz, 2H), 3.87 (s, 3H), 1.33 (t, *J* = 7.1 Hz, 3H) ppm. ^13^C{^1^H} NMR (100.62 MHz, CDCl_3_): δ 165.3, 153.0, 142.5, 140.4, 130.9, 126.2, 118.4, 87.1, 62.0, 52.3, 14.5 ppm. FTIR (NaCl): ν 3380 (s, N–H), 2976 (m, C–H), 1748 (s, C=O), 1709 (s, C=O), 1520 (s), 1242 (s), 759 (s, C–Br) cm^–1^. HRMS (ESI) *m*/*z*: [M + H]^+^ calcd for C_11_H_13_INO_4_ 349.9884; found 349.9889.

#### Ethyl (2-Iodo-4-methoxyphenyl)carbamate (**7e**)

Following the general procedure for carbamate formation described above the reaction of 2-iodo-4-methoxyaniline (0.20 g, 0.80 mmol), ethyl chloroformate (0.09 mL, 0.96 mmol), and K_2_CO_3_ (0.14 g, 1.04 mmol) in THF (4 mL) at 25 °C for 22 h afforded 0.21 g (81%) of **7e** as a white solid. Mp: 79–80 °C (hexane/EtOAc). ^1^H NMR (400.16 MHz, CDCl_3_): δ 7.81 (d, *J* = 9.0 Hz, 1H), 7.30 (d, *J* = 2.9 Hz, 1H), 6.90 (dd, *J* = 9.0, 2.9 1H), 6.65 (s, 1H), 4.23 (q, *J* = 7.1 Hz, 2H), 3.76 (s, 3H), 1.32 (t, *J* = 7.1 Hz, 3H) ppm. ^13^C{^1^H} NMR (100.62 MHz, CDCl_3_): δ 156.4, 154.0), 132.1, 123.9, 122.2, 115.1, 90.9, 61.6, 55.8, 14.7) ppm. FTIR (NaCl): ν 3279 (s, N–H), 2978 (w), 2938 (w), 2904 (w), 2838 (w), 1697 (s, C=O), 1528 (s, N–C=O), 1401 (w), 1283 (s, C–O–C), 1240 (s, C–O), 1216 (s, C–O), 1034 (w), 1024 (w), 854 (w) cm^–1^. HRMS (ESI) *m*/*z*: [M + H]^+^ calcd for C_10_H_13_INO_3_ 321.9935; found 321.9931.

#### *tert*-Butyl[(2,2-dimethylbut-3-yn-1-yl)oxy]diphenylsilane (**8n**)

To a cooled (0 °C) solution of 4-hydroxy-3,3-dimethylbutan-2-one^108^ (5.5 g, 47 mmol) in DMF (95 mL), TBDPSCl (18.5 mL, 71 mmol) and imidazole (8.1 g, 118 mmol) were added. The resulting mixture was stirred at 25 °C for 20 h under argon. Then, the mixture was poured over a 1:1 NaHCO_3_ (sat)/water solution, and the mixture was extracted with CH_2_Cl_2_ (3×). The combined organic layers were washed with water (3×), dried (Na_2_SO_4_), and concentrated. The residue was purified by flash-column chromatography (silica gel, 95: hexane/EtOAc) to afford 8.0 g (68%) of the silyl ether 4-[(*tert*-butyldiphenylsilyl)oxy]-3,3-dimethylbutan-2-one. ^1^H NMR (400.16 MHz, CDCl_3_): δ 7.68–7.62 (m, 4H), 7.48–7.36 (m, 6H), 3.65 (s, 2H), 2.18 (s, 3H), 1.13 (s, 6H), 1.06 (s, 9H) ppm. ^13^C{^1^H} NMR (100.62 MHz, CDCl_3_): δ 213.0, 135.8 (4×), 133.3 (2×), 129.9 (2×), 127.8 (4×), 70.9, 50.0, 27.0 (3×), 26.2, 21.7 (2×), 19.4 ppm. FTIR (NaCl): ν 2961 (m, C–H), 2932 (m, C–H), 2859 (m, C–H), 1710 (s), 1472 (m), 1427 (m), 1392 (m), 1360 (m), 1109 (s), 703 (s) cm^–1^. HRMS (ESI) *m*/*z*: [M + H]^+^ calcd for C_22_H_31_O_2_Si 355.2088; found 355.2082. This material was processed as follows: *n*-BuLi (1.77 M in hexanes, 1.2 mL, 2.07 mmol) was added to a cooled (0 °C) solution of diisopropylamine (0.307 mL, 2.17 mmol) in THF (3.9 mL), and the resulting mixture was stirred at 0 °C for 30 min. Then, this solution was cooled down to −78 °C, and a solution of 4-[(*tert*-butyldiphenylsilyl)oxy]-3,3-dimethylbutan-2-one (1.00 g, 1.97 mmol) in THF (2.0 mL) was added. After stirring for 1 h at the same temperature, diethyl chlorophosphate (0.314 mL, 2.17 mmol) was added, and the reaction mixture was allowed to warm up to 25 °C over a period of 90 min. Then, a second LDA solution was prepared by the addition of *n*-BuLi (1.77 M in hexanes, 2.5 mL, 4.44 mmol) to a solution of diisopropylamine (0.642 mL, 4.54 mmol) in THF (3.9 mL) at 0 °C and further stirring for 30 min at the same temperature. Then, the previous reaction mixture was added to this freshly prepared LDA solution at −78 °C, and stirring was continued at this temperature for 1 h. After additional stirring for 1 h at 25 °C, the reaction mixture was quenched at 0 °C by the addition of a saturated aqueous solution of NH_4_Cl. Then Et_2_O was added, the layers were separated, and the aqueous layer was extracted with Et_2_O (3×). The combined organic layers were washed with a saturated aqueous solution of NaHCO_3_ and dried (Na_2_SO_4_), and the solvent was evaporated. The residue was purified by flash-column chromatography (silica gel, gradient from hexane to 95:5 hexane/EtOAc) to afford 0.34 g (52%) of **8n** as a yellow oil. ^1^H NMR (400.16 MHz, CDCl_3_): δ 7.72–7.66 (m, 4H), 7.46–7.35 (m, 6H), 3.52 (s, 2H), 2.08 (s, 1H), 1.26 (s, 6H), 1.08 (s, 9H) ppm. ^13^C{^1^H} NMR (100.62 MHz, CDCl_3_): δ 135.9 (4×), 133.8 (2×), 129.8 (2×), 127.8 (4×), 90.6, 71.8, 68.4, 33.7, 27.0 (3×), 25.7 (2×), 19.6. FTIR (NaCl): ν 3066 (m, C–H), 2948 (m, C–H), 2929 (m, C–H), 1690 (s), 1632 (m), 1466 (m), 1387 (m), 1247 (m), 1107 (s), 746 (s) cm^–1^. HRMS (ESI) *m*/*z*: [M + H]^+^ calcd for C_22_H_29_OSi 337.1982; found 337.1984.

### Preparation of 2-Alkynylaniline Derivatives **2** and **9** by Sonogashira Reaction

*Procedure A*: In a typical experiment, to a solution of an appropriate *o*-iodoaniline derivative (0.29 mmol) and alkyne **8** (1.18 mmol) in Et_3_N (0.2 mL) and DMF (2.9 mL) were added PdCl_2_(PPh_3_)_2_ (4.2 mg, 0.006 mmol) and CuI (2.3 mg, 0.012 mmol), and the mixture was stirred at 25 °C under argon. After reaction completion, a saturated aqueous solution of NH_4_Cl was added, and the mixture was extracted with CH_2_Cl_2_ (3×). The combined organic layers were dried (Na_2_SO_4_), and the solvent was evaporated. The residue was purified by flash-column chromatography to afford product **2** or **9**. *Procedure B*: In a typical experiment, to a solution of an appropriate *o*-iodoaniline derivative (1.18 mmol) and alkyne **8** (4.72 mmol) in Et_3_N (2.6 mL) were added PdCl_2_(PPh_3_)_2_ (16.8 mg, 0.024 mmol) and CuI (4.6 mg, 0.024 mmol), and the mixture was stirred at 25 °C under argon. It then proceeded as in *procedure A* to afford product **2** or **9**. *Procedure C*: In a typical experiment, to a solution of an appropriate *o*-iodoaniline derivative (0.29 mmol) and alkyne **8** (0.35 mmol) in Et_3_N (0.6 mL) and DMF (0.3 mL) were added PdCl_2_(PPh_3_)_2_ (4.2 mg, 0.006 mmol) and CuI (0.6 mg, 0.003 mmol), and the mixture was stirred at 25 °C under argon. It then proceeded as in *procedure A* to afford product **2** or **9**. *Procedure D*: In a typical experiment, to a solution an appropriate *o*-iodoaniline derivative (0.34 mmol) and alkyne **8** (0.69 mmol) in a mixture of THF/Et_3_N (6.4 mL, 4:1 v/v) were added PdCl_2_(PPh_3_)_2_ (4.8 mg, 0.007 mmol) and CuI (2.6 mg, 0.014 mmol), and the mixture was stirred at 25 °C under argon. It then proceeded as in *procedure A* to afford product **2** or **9**.

#### Ethyl [2-(Oct-1-yn-1-yl)phenyl]carbamate (**2b**)

The Sonogashira Procedure A was followed from ethyl (2-iodophenyl)carbamate^27,107^ (**7a**) (500 mg, 1.72 mmol) and oct-1-yne (**8b**) (1.0 mL, 6.87 mmol) (reaction time 20 h) to afford, after purification by flash-column chromatography (silica gel; solvent A, *n*-hexane; solvent B, EtOAc; gradient from 100:0 to 80:20 A/B), 413 mg (88%) of **2b** as a yellow oil. ^1^H NMR (400.16 MHz, CDCl_3_): δ 8.13 (d, *J* = 8.4 Hz, 1H), 7.42 (s, 1H, NH), 7.34 (dd, *J* = 7.7, 1.5 Hz, 1H), 7.32–7.22 (m, 1H), 6.95 (td, *J* = 7.6, 1.2 Hz, 1H), 4.24 (q, *J* = 7.1 Hz, 2H), 2.49 (t, *J* = 7.0 Hz, 2H), 1.70–1.57 (m, 2H), 1.54–1.43 (m, 2H), 1.37–1.31 (m, 7H), 0.92 (t, *J* = 7.1 Hz, 3H) ppm. ^13^C{^1^H} NMR (100.62 MHz, CDCl_3_): δ 153.4, 139.1, 131.7, 128.9, 122.3, 117.4, 112.2, 97.8, 75.9, 61.4, 31.5, 28.7 (2×), 22.7, 19.7, 14.7, 14.2 ppm. FTIR (NaCl): ν 3398 (s, N–H), 2956 (w), 2931 (w), 2858 (w), 1741 (s, C=O), 1580 (s), 1521 (s), 1452 (s), 1230 (w), 1210 (w) cm^–1^. HRMS (ESI) *m*/*z*: [M + H]^+^ calcd for C_17_H_24_NO_2_ 274.1803; found 274.1802.

#### Ethyl [2-(3,3-Dimethylbut-1-yn-1-yl)phenyl]carbamate (**2c**)

The Sonogashira Procedure B was followed from **7a**(27,107) (200 mg, 0.69 mmol) and *tert*-butylacetylene (**8c**) (0.10 mL, 0.83 mmol) (reaction time 3 h) to afford, after purification by flash-column chromatography (silica gel; solvent A, *n*-hexane; solvent B, EtOAc; gradient from 100:0 to 90:10 A/B), 165 mg (98%) of **2c** as a colorless oil. ^1^H NMR (400.16 MHz, CDCl_3_): δ 8.12 (d, *J* = 8.4 Hz, 1H), 7.39 (s, 1H), 7.32 (ddd, *J* = 7.7, 1.6, 0.5 Hz, 1H), 7.26 (dddd, *J* = 8.2, 7.4, 1.6, 0.5 Hz, 1H), 6.94 (td, *J* = 7.6, 1.2 Hz, 1H), 4.24 (q, *J* = 7.1 Hz, 2H), 1.37 (s, 9H), 1.32 (t, *J* = 7.1 Hz, 3H) ppm. ^13^C{^1^H} NMR (100.62 MHz, CDCl_3_): δ 153.4, 139.0, 131.3, 128.9, 122.2, 117.4, 112.1, 106.0, 74.5, 61.3, 31.1 (3×), 28.4, 14.6 ppm. FTIR (NaCl): ν 3399 (m, N–H), 2970 (m, C–H), 1742 (s, C=O), 1582 (m), 1522 (s), 1454 (m), 1216 (s) cm^–1^. HRMS (ESI) *m*/*z*: [M + H]^+^ calcd for C_15_H_20_NO_2_ 246.1488; found 246.1486.

#### Ethyl [2-Cyclohexyl)ethynyl)phenyl]carbamate (**2o**)

The Sonogashira Procedure A was followed from **7a**(27,107) (500 mg, 1.72 mmol) and 1-ethynylcyclohexane (**8k**) (0.90 mL, 6.87 mmol) (reaction time 20 h) to afford, after purification by flash-column chromatography (silica gel; solvent A, *n*-hexane; solvent B, EtOAc; gradient from 100:0 to 80:20 A/B), 357 mg (77%) of **2o** as a yellow oil. ^1^H NMR (400.16 MHz, CDCl_3_): δ 8.11 (d, *J* = 8.4 Hz, 1H), 7.44 (s, 1H, NH), 7.34 (dd, *J* = 7.9, 1.4 Hz, 1H), 7.29–7.24 (m, 1H), 6.95 (td, *J* = 7.6, 1.2 Hz, 1H), 4.24 (q, *J* = 7.1 Hz, 2H), 2.70 (tt, *J* = 8.4, 3.7 Hz, 1H), 1.98–1.84 (m, 2H), 1.80–1.74 (m, 2H), 1.64–1.52 (m, 3H), 1.47–1.36 (m, 3H), 1.33 (t, *J* = 7.1 Hz, 3H) ppm. ^13^C{^1^H} NMR (100.62 MHz, CDCl_3_): δ 153.5, 139.1, 131.5, 128.9, 122.3, 117.4, 112.2, 101.9, 75.9, 61.4, 32.7 (2×), 29.8, 25.9 (2×), 24.8, 14.7 ppm. FTIR (NaCl): ν 3395 (s, N–H), 2979 (w), 2930 (s), 2854 (s), 1741 (s, C=O), 1581 (s), 1521 (s), 1451(s), 1307 (w), 1227 (s), 1207 (s) cm^–1^. HRMS (ESI) *m*/*z*: [M + H]^+^ calcd for C_17_H_22_NO_2_ 272.1646; found 272.1645.

#### Ethyl [2-Cyclohex-1-en-1-ylethynyl)phenyl]carbamate (**2p**)

The Sonogashira Procedure A was followed from **7a**(27,107) (500 mg, 1.72 mmol) and 1-ethynylcyclohex-1-ene (**8l**) (0.81 mL, 6.87 mmol) (reaction time 20 h) to afford, after purification by flash-column chromatography (silica gel; solvent A, *n*-hexane; solvent B, EtOAc; gradient from 100:0 to 80:20 A/B), 460 mg (99%) of **2p** as a yellow oil. ^1^H NMR (400.16 MHz, CDCl_3_): δ 8.15 (d, *J* = 8.4 Hz, 1H), 7.38 (dd, *J* = 7.7, 1.7 Hz, 2H), 7.33–7.27 (m, 1H), 6.99 (tt, *J* = 7.6, 1.0 Hz, 1H), 6.32–6.25 (m, 1H), 4.27 (q, *J* = 7.1 Hz, 2H), 2.27 (ddt, *J* = 5.9, 4.4, 2.4 Hz, 2H), 2.23–2.15 (m, 2H), 1.77–1.69 (m, 2H), 1.69–1.62 (m, 2H), 1.36 (t, *J* = 7.1 Hz, 3H) ppm. ^13^C{^1^H} NMR (100.62 MHz, CDCl_3_): δ 153.4, 138.9, 136.2, 131.6, 129.3, 122.4, 120.3, 117.6, 111.9, 98.3, 81.7, 61.4, 29.3, 25.9, 22.4, 21.5, 14.7 ppm. FTIR (NaCl): ν 3398 (s, N–H), 2979 (w), 2932 (m), 2859 (w), 2837 (w), 1740 (s, C=O), 1579 (m), 1520 (s), 1452 (m), 1208 (s), 1060 (w) cm^–1^. HRMS (ESI) *m*/*z*: [M + H]^+^ calcd for C_17_H_20_NO_2_ 270.1488; found 270.1489.

#### N-(4-Methoxybenzyl)-2-(oct-1-yn-1-yl)aniline (**9a**)

The Sonogashira Procedure A was followed from 2-iodo-*N*-(4-methoxybenzyl)aniline^109^ (100 mg, 0.29 mmol) and **8b** (0.174 mL, 1.18 mmol) at 60 °C (reaction time 16 h) to afford, after purification by flash-column chromatography (silica gel; solvent A, hexane; solvent B, EtOAc; gradient from 100:0 to 80:20 A/B), 52 mg (55%) of **9a** as an oil. ^1^H NMR (400.16 MHz, CDCl_3_): δ 7.30 (d, *J* = 8.7 Hz, 2H), 7.27 (dd, *J* = 7.5, 1.5 Hz, 1H), 7.12 (ddd, *J* = 7.7, 7.0, 1.5 Hz, 1H), 6.89 (d, *J* = 8.7 Hz, 2H), 6.62 (td, *J* = 7.5, 1.0 Hz, 1H), 6.57 (d, *J* = 8.1 Hz, 1H), 4.94 (t, *J* = 5.6 Hz, 1H), 4.34 (d, *J* = 5.6 Hz, 2H), 3.81 (s, 3H), 2.45 (t, *J* = 7.0 Hz, 2H), 1.63–1.53 (m, 2H), 1.47–1.37 (m, 2H), 1.33–1.23 (m, 4H), 0.90 (t, *J* = 7.0 Hz 3H) ppm. ^13^C{^1^H} NMR (100.62 MHz, CDCl_3_): δ 158.9, 148.8, 132.0, 131.4, 129.2, 128.6 (2×), 116.5, 114.1 (2×), 109.8, 108.7, 96.4, 77.2, 55.4, 47.4, 31.5, 29.0, 28.7, 22.7, 19.8, 14.2 ppm. FTIR (NaCl): ν 3396 (m, N–H), 2924 (s, C–H), 2858 (s, C–H), 1605 (m), 1508 (s), 1246 (s), 741 (m) cm^–1^. HRMS (ESI) *m*/*z*: [M + H]^+^ calcd for C_22_H_28_NO 322.2165; found 322.2161.

#### 3-[2-(4-Methoxybenzylamino)phenyl]prop-2-yn-1-yl Acetate (**9b**)

The Sonogashira Procedure B was followed from 2-iodo-*N*-(4-methoxybenzyl)aniline^109^ (400 mg, 1.18 mmol) and prop-2-yn-1-yl acetate (**8m**) (0.468 mL, 4.72 mmol) in Et_3_N (2.6 mL) (reaction time 24 h) to afford, after purification by flash-column chromatography (silica gel; solvent A, hexane; solvent B, EtOAc; gradient from 100:0 to 80:20 A/B), 170 mg (47%) of **9b** as a yellow solid. Mp: 59–60 °C (hexane/EtOAc). ^1^H NMR (400.16 MHz, CDCl_3_): δ 7.31 (dd, *J* = 7.5, 1.5 Hz, 1H), 7.29 (d, *J* = 8.7 Hz, 2H), 7.16 (ddd, *J* = 8.3, 7.4, 1.6 Hz, 1H), 6.89 (d, *J* = 8.7 Hz, 2H), 6.62 (td, *J* = 7.5, 1.1 Hz, 1H), 6.57 (d, *J* = 8.3 Hz, 1H), 5.05 (br s, 1H), 4.92 (s, 2H), 4.36 (s, 2H), 3.80 (s, 3H), 2.09 (s, 3H) ppm. ^13^C{^1^H} NMR (100.62 MHz, CDCl_3_): δ 170.4, 158.9, 149.4, 132.6, 131.1, 130.6, 128.4 (2×), 116.4, 114.1 (2×), 110.0, 106.3, 89.0, 83.6, 55.3, 53.1, 47.1, 20.9 ppm. FTIR (NaCl): ν 3398 (m, N–H), 3000 (w, C–H), 2936 (w), 2836 (w), 2223 (w, C≡C), 1741 (s, C=O), 1507 (s), 1229 (s), 1028 (s), 743 (m) cm^–1^. HRMS (ESI) *m*/*z*: [M + H]^+^ calcd for C_19_H_20_NO_3_ 310.1438; found 310.1429.

#### 2-(Cyclohexylethynyl)-N-(4-methoxybenzyl)aniline (**9c**)

The Sonogashira Procedure C was followed from 2-iodo-*N*-(4-methoxybenzyl)aniline^109^ (100 mg, 0.29 mmol) and **8k** (0.046 mL, 0.35 mmol) (reaction time 24h) to afford, after purification by flash-column chromatography (silica gel; solvent A, hexane; solvent B, EtOAc; gradient from 100:0 to 80:20 A/B), 58 mg (62%) of **9c** as an oil. ^1^H NMR (400.16 MHz, CDCl_3_): δ 7.31 (d, *J* = 8.7 Hz, 2H), 7.28 (dd, *J* = 7.6, 1.6 Hz, 1H), 7.13 (ddd, *J* = 8.2, 7.4, 1.6 Hz, 1H), 6.89 (d, *J* = 8.7 Hz, 2H), 6.62 (td, *J* = 7.5, 1.1 Hz, 1H), 6.58 (d, *J* = 8.2 Hz, 1H), 4.95 (br s, 1H), 4.34 (s, 2H), 3.81 (s, 3H), 2.66 (tt, *J* = 8.6, 3.8 Hz, 1H), 1.88–1.79 (m, 2H), 1.75–1.64 (m, 2H), 1.58–1.45 (m, 3H), 1.40–1.26 (m, 3H) ppm. ^13^C{^1^H} NMR (100.62 MHz, CDCl_3_): δ 159.0, 148.8, 131.8, 131.4, 129.1, 128.6 (2×), 116.5, 114.1 (2×), 109.7, 108.7, 100.6, 77.2), 55.4, 47.5, 32.9 (2×), 29.9, 26.0 (2×), 24.8 ppm. FTIR (NaCl): ν 3400 (m, N–H), 3006 (m, C–H), 2928 (s, C–H), 2851 (s, C–H), 1603 (s), 1508 (s), 1454 (s), 1246 (s), 744 (s) cm^–1^. HRMS (ESI) *m*/*z*: [M + H]^+^ calcd for C_22_H_26_NO 320.2009; found 320.2004.

#### 2-(3,3-Dimethylbut-1-yn-1-yl)-N-(4-methoxybenzyl)aniline (**9d**)

The Sonogashira Procedure B was followed from 2-iodo-*N*-(4-methoxybenzyl)aniline^109^ (100 mg, 0.29 mmol) and **8c** (0.044 mL, 0.35 mmol) (reaction time 4 h) to afford, after purification by flash-column chromatography (silica gel, solvent A: hexane; solvent B: EtOAc; gradient from 100:0 to 80:20 A/B) 84 mg (98%) of **9d** as a yellow solid. Mp: 30–31 °C (hexane/EtOAc). ^1^H NMR (400.16 MHz, CDCl_3_): δ 7.32 (d, *J* = 8.4 Hz, 2H), 7.28 (dd, *J* = 7.5, 1.5 Hz, 1H), 7.14 (ddd, *J* = 8.2, 7.4, 1.6 Hz, 1H), 6.91 (d, *J* = 8.4 Hz, 2H), 6.63 (td, *J* = 7.5, 1.1 Hz, 1H), 6.59 (dd, *J* = 8.2, 1.0 Hz, 1H), 4.92 (s, 1H), 4.35 (s, 2H), 3.82 (s, 3H), 1.32 (s, 9H) ppm. ^13^C{^1^H} NMR (100.62 MHz, CDCl_3_): δ 158.9, 148.7, 131.7, 131.4, 129.2, 128.5 (2×), 116.5, 114.1 (2×), 109.8, 108.6, 104.9, 75.7, 55.4, 47.5, 31.4 (3×) 28.4 ppm. FTIR (NaCl): ν 3401 (m, N–H), 3064 (m, C–H), 2967 (s, C–H), 2903 (m, C–H), 2841 (m, C–H), 1607 (s), 1579 (s), 1506 (s), 1457 (s), 1247 (s), 741 (s) cm^–1^. HRMS (ESI) *m*/*z*: [M + H]^+^ calcd for C_20_H_24_NO 294.1852; found 294.1850.

#### 2-{4-[(*tert*-Butyldiphenylsilyl)oxy]-3,3-dimethylbut-1-yn-1-yl}-N-(4-methoxybenzyl)aniline (**9e**)

The Sonogashira Procedure C was followed from 2-iodo-*N*-(4-methoxybenzyl)aniline^109^ (500 mg, 1.47 mmol) and **8n** (595 mg, 1.77 mmol) (reaction time 48 h) to afford, after purification by flash-column chromatography (C18-silica gel; solvent A, acetonitrile; solvent B, water; gradient from 70:30 to 100:0 A/B), 580 mg (72%) of **9e** as a yellow oil. ^1^H NMR (400.16 MHz, CDCl_3_): δ 7.72–7.66 (m, 4H), 7.44–7.37 (m, 2H), 7.38–7.30 (m, 4H), 7.25–7.19 (m, 3H), 7.10 (ddd, *J* = 8.3, 7.4, 1.6 Hz, 1H), 6.83 (d, *J* = 8.0 Hz, 2H), 6.59 (td, *J* = 7.5, 1.1 Hz, 1H), 6.52 (d, *J* = 8.2 Hz, 1H), 4.94 (br s, 1H), 4.26 (d, *J* = 3.9 Hz, 2H), 3.79 (s, 3H), 3.58 (s, 2H), 1.30 (s, 6H), 1.07 (s, 9H) ppm. ^13^C{^1^H} NMR (100.62 MHz, CDCl_3_): δ 158.8, 148.8, 135.8 (4×), 133.7, 131.9, 131.4 (2×), 129.8 (2×), 129.3, 128.3 (2×), 127.8 (4×), 116.4, 114.1 (2×), 109.8, 108.4, 102.2, 77.4, 72.1, 55.4, 47.3, 34.8, 27.0 (3×), 26.1 (2×), 19.6 ppm. FTIR (NaCl): ν 3398 (w, N–H), 3067 (w, C–H), 2960 (s, C–H), 2927 (s, C–H), 2863 (s, C–H), 1606 (m), 1509 (s), 1464 (m), 1246 (m), 1106 (s) cm^–1^. HRMS (ESI) *m*/*z*: [M + H]^+^ calcd for C_36_H_42_NO_2_Si 548.2979; found 548.2995.

#### 4-[2-(4-Methoxybenzylamino)phenyl]-2-methylbut-3-yn-2-ol (**9f**)

The Sonogashira Procedure D was followed from 2-iodo-*N*-(4-methoxybenzyl)aniline^109^ (400 mg, 1.18 mmol) and 2-methylbut-3-yn-2-ol (**8o**) (0.23 mL, 2.36 mmol) (reaction time 12 h) to afford, after purification by flash-column chromatography (silica gel, solvent A: hexane; solvent B: EtOAc; gradient from 90:10 to 80:20 A/B), 340 mg (98%) of **9f** as a brown oil. ^1^H NMR (400.16 MHz, CDCl_3_): δ 7.31–7.25 (m, 3H), 7.18–7.12 (m, 1H), 6.88 (d, *J* = 8.6 Hz, 2H), 6.62 (td, *J* = 7.5, 1.0 Hz, 1H), 6.57 (d, *J* = 8.2 Hz, 1H), 4.87 (br s, 1H, NH), 4.34 (s, 2H), 3.81 (s, 3H), 1.60 (s, 6H), 1.53 (br s, 1H, OH) ppm. ^13^C{^1^H} NMR (100.62 MHz, CDCl_3_): δ 158.8, 148.8, 132.1, 131.1, 129.9, 128.3 (2×), 116.5, 114.1 (2×), 110.0, 107.1, 100.1, 78.9, 65.8, 55.3, 47.2, 31.7, 31.1 ppm. FTIR (NaCl): ν 3600–3000 (br, O–H), 2979 (m, C–H), 2932 (m, C–H), 2835 (m, C–H), 1509 (s), 1248 (s), 1173 (s), 772 (s) cm^–1^. HRMS (ESI) *m*/*z*: [M + H]^+^ calcd for C_19_H_22_NO_2_ 296.1645; found 296.1639.

#### 2-(Cyclohex-1-en-1-ylethynyl)-N-(4-methoxybenzyl)aniline (**9g**)

The Sonogashira Procedure B was followed from 2-iodo-*N*-(4-methoxybenzyl)aniline^109^ (100 mg, 0.29 mmol) and **8l** (0.042 mL, 0.35 mmol) (reaction time 6 h) to afford, after purification by flash-column chromatography (silica gel, solvent A: hexane; solvent B: EtOAc; gradient from 100:0 to 80:20 A/B), 91 mg (98%) of **9g** as a yellow solid. Mp: 38–39 °C (hexane/EtOAc). ^1^H NMR (400.16 MHz, CDCl_3_): δ 7.29 (d, *J* = 8.7 Hz, 2H), 7.28 (dd, *J* = 7.6, 1.6 Hz, 1H), 7.12 (ddd, *J* = 8.3, 7.4, 1.6 Hz, 1H), 6.88 (d, *J* = 8.7 Hz, 2H), 6.62 (td, *J* = 7.5, 1.1 Hz, 1H), 6.56 (d, *J* = 8.4 Hz, 1H), 6.15 (tt, *J* = 3.9, 1.8 Hz, 1H), 5.00 (br s, 1H), 4.35 (s, 2H), 3.81 (s, 3H), 2.23–2.17 (m, 2H), 2.17–2.10 (m, 2H), 1.72–1.57 (m, 4H) ppm. ^13^C{^1^H} NMR (100.62 MHz, CDCl_3_): δ 158.9, 148.6, 134.9, 132.0, 131.3, 129.6, 128.5 (2×), 120.9, 116.7, 114.2 (2×), 110.0, 108.3, 97.4, 83.3, 55.4, 47.4, 29.6, 25.9, 22.5, 21.7 ppm. FTIR (NaCl): ν 3400 (m, N–H), 2926 (s, C–H), 2853 (s, C–H), 2190 (w, C≡C), 1601 (m), 1507 (s), 1454 (m), 1244 (s), 1034 (m), 747 (m) cm^–1^. HRMS (ESI) *m*/*z*: [M + H]^+^ calcd for C_22_H_24_NO [(M+H)^+^] 318.1852; found 318.1857.

#### 2-(3,3-Dimethylbut-1-yn-1-yl)-N-(4-methoxybenzyl)-4-methylaniline (**9h**)

The Sonogashira Procedure D was followed from 2-iodo-*N*-(4-methoxybenzyl)-4-methylaniline^110^ (710 mg, 2.04 mmol) and **8c** (0.50 mL, 4.07 mmol) (reaction time 16 h) to afford, after purification by flash-column chromatography (silica gel; solvent A, acetonitrile; solvent B, water; gradient from 50:50 to 100:0 A/B), 593 mg (95%) of **9h** as a yellow oil. ^1^H NMR (400.16 MHz, CDCl_3_): δ 7.29 (d, *J* = 8.8 Hz, 2H), 7.08 (d, *J* = 2.2 Hz, 1H), 6.94–6.89 (m, 1H), 6.87 (d, *J* = 8.7 Hz, 2H), 6.47 (d, *J* = 8.3 Hz, 1H), 4.72 (t, *J* = 5.5 Hz, 1H, NH), 4.30 (d, *J* = 5.6 Hz, 2H), 3.80 (s, 3H), 2.18 (s, 3H), 1.28 (s, 9H) ppm. ^13^C{^1^H} NMR (100.62 MHz, CDCl_3_): δ 158.8, 146.6, 139.3, 132.1, 131.6, 129.8, 128.5 (2×), 125.6, 114.0 (2×), 109.9, 108.5, 104.5, 75.7, 55.4, 47.7, 31.4 (3×), 20.3 ppm. FTIR (NaCl): ν 3397 (w, N–H), 2965 (w), 2925 (w), 2864 (w), 2834 (w), 1511 (s), 1248 (w) cm^–1^. HRMS (ESI) *m*/*z*: [M + H]^+^ calcd for C_21_H_26_NO 308.2009; found 308.2011.

#### Methyl 3-(3,3-Dimethylbut-1-yn-1-yl)-4-[(4-methoxybenzyl)amino]benzoate (**9i**)

The Sonogashira Procedure D was followed from methyl 3-iodo-4-[(4-methoxybenzyl)amino]benzoate^109^ (500 mg, 1.26 mmol) and **8c** (0.31 mL, 2.52 mmol) (reaction time 16 h) to afford, after purification by flash-column chromatography (silica gel; solvent A, *n*-hexane; solvent B, EtOAc; gradient from 100:0 to 90:10 A/B), 428 mg (95%) of **9i** as a brown oil. ^1^H NMR (400.16 MHz, CDCl_3_): δ 7.95 (d, *J* = 2.1 Hz, 1H), 7.80 (dd, *J* = 8.5, 1.8 Hz, 1H), 7.27 (d, *J* = 8.5 Hz, 2H), 6.89 (d, *J* = 8.6 Hz, 2H), 6.54 (d, *J* = 8.7 Hz, 1H), 5.29 (t, *J* = 5.3 Hz, 1H, NH), 4.37 (d, *J* = 5.3 Hz, 2H), 3.84 (s, 3H), 3.81 (s, 3H), 1.28 (s 9H) ppm. ^13^C{^1^H} NMR (100.62 MHz, CDCl_3_): δ 167.0, 159.1, 151.7, 133.7, 131.3, 130.2, 128.5 (2×), 117.8, 114.4, 114.2 (2×), 108.6, 108.0, 105.4, 74.6, 55.4, 51.7, 47.0, 31.2 (3×) ppm. FTIR (NaCl): ν 3397 (m, N–H), 2967 (w), 2900 (w), 2866 (w), 2837 (w), 1709 (s, C=O), 1604 (s), 1514 (s), 1302 (s), 1249 (s), 1176 (w), 1131 (w) cm^–1^. HRMS (ESI) *m*/*z*: [M + H]^+^ calcd for C_22_H_26_NO_3_ 352.1910; found 352.1907.

#### 2-(Cyclohex-1-en-1-ylethynyl)-N-(4-methoxybenzyl)-4-methylaniline (**9j**)

The Sonogashira Procedure D was followed from 2-iodo-*N*-(4-methoxybenzyl)-4-methylaniline^110^ (600 mg, 1.70 mmol) and **8l** (0.40 mL, 3.40 mmol) (reaction time 16 h) to afford, after purification by flash-column chromatography (silica gel; solvent A, acetonitrile; solvent B, water; gradient from 50:50 to 100:0 A/B), 231 mg (41%) **9j** as a yellow oil. ^1^H NMR (400.16 MHz, CDCl_3_): δ 7.29 (d, *J* = 8.8 Hz, 2H), 7.16–7.09 (m, 1H), 6.93 (ddd, *J* = 8.3, 2.2, 0.8 Hz, 1H), 6.88 (d, *J* = 8.7 Hz, 2H), 6.47 (d, *J* = 8.3 Hz, 1H), 6.15 (tt, *J* = 3.9, 1.8 Hz, 1H), 4.82 (br s, 1H, NH), 4.33 (s, 2H), 3.81 (s, 3H), 2.24–2.17 (m, 5H), 2.14 (tdd, *J* = 6.1, 4.0, 2.1 Hz, 2H), 1.72–1.59 (m, 4H) ppm. ^13^C{^1^H} NMR (100.62 MHz, CDCl_3_): δ 158.8, 146.5, 134.7, 132.2, 131.6, 130.2, 128.4 (2×), 125.6, 120.8, 114.1 (2×), 110.1, 108.2, 97.1, 83.5, 55.4, 47.5, 29.6, 25.9, 22.5, 21.6, 20.3 ppm. FTIR (NaCl): ν 3398 (w, N–H), 3021 (w), 2928 (w), 2857 (w), 2834 (w), 1611 (w), 1509 (s), 1437 (w), 1246 (m), 1034 (w) cm^–1^. HRMS (ESI) *m*/*z*: [M + H]^+^ calcd for C_23_H_26_NO 332.2004; found 332.2008.

#### N-(4-Methoxybenzyl)-2-[(triethylsilyl)ethynyl]aniline (**9k**)

The Sonogashira Procedure D was followed from 2-iodo-*N*-(4-methoxybenzyl)aniline^109^ (400 mg, 1.18 mmol) and triethyl(ethynyl)silane (**8p**) (0.42 mL, 2.36 mmol) (reaction time 12 h) to afford, after purification by flash-column chromatography (silica gel, solvent A: hexane; solvent B: EtOAc; gradient from 95:5 to 90:10 A/B), 214 mg (52%) of **9k** as a yellow oil. ^1^H NMR (400.16 MHz, CDCl_3_): δ 7.32 (dd, *J* = 7.6, 1.6 Hz, 1H), 7.29 (d, *J* = 8.8 Hz, 2H), 7.21–7.13 (m, 1H), 6.88 (d, *J* = 8.6 Hz, 2H), 6.65–6.56 (m, 2H), 4.97 (br s, 1H, NH), 4.31 (s, 2H), 3.81 (s, 3H), 0.97 (t, *J* = 7.9 Hz, 9H), 0.62 (q, *J* = 7.9 Hz, 6H) ppm. ^13^C{^1^H} NMR (100.62 MHz, CDCl_3_): δ 159.1, 149.4, 132.2, 131.0, 130.2, 128.8 (2×), 116.4, 114.2 2×), 109.7, 107.8, 103.3, 97.9, 55.4, 47.5, 7.6 (3×), 4.6 (3×) ppm. FTIR (NaCl): ν 3398 (s, N–H), 2953 (s, C–H), 2933 (m, C–H), 2909 (m, C–H), 2873 (m, C–H), 2834 (w, C–H), 2141 (w, C≡C), 1602 (s), 1510 (s), 1458 (s), 1249 (s), 1038 (s) cm^–1^. HRMS (ESI) *m*/*z*: [M + H]^+^ calcd for C_22_H_30_NOSi 352.2091; found 352.2085.

### Preparation of Dehydrotryptophans **4a**–**c** or **5a**–**b** from **1a**–**c** or **2a**–**b** and **3** (Table 1)

In a typical experiment, to a solution of the appropriate alkynylaniline derivative **1** or **2** (0.24 mmol) and methyl α-acetamidoacrylate (**3**) (207.2 mg, 1.45 mmol) in DMF (2.4 mL) were added PdCl_2_(PPh_3_)_2_ (8.5 mg, 0.012 mmol) and KI (20.0 mg, 0.121 mmol). The resulting mixture was stirred at 100 °C with the flask open to air. After reaction completion, a saturated aqueous solution of NaHCO_3_ was added, and the mixture was extracted with EtOAc (3×). The combined organic layers were dried (Na_2_SO_4_), and the solvent was evaporated. The residue was purified by flash-column chromatography to afford products **4** and/or **5**.

#### Methyl (Z)-2-Acetamido-3-[2-(p-tolyl)-1H-indol-3-yl]acrylate (**4a**)

The reaction of 2-(*p*-tolylethynyl)aniline (**1a**)^14,111^ (50.0 mg, 0.24 mmol) and methyl 2-acetamidoacrylate (**3**) (207.2 mg, 1.45 mmol) in 7 h afforded, after purification by flash-column chromatography (CN-silica gel, solvent A: hexane; solvent B: EtOAc; gradient from 100:0 to 50:50 A/B), 50.4 mg (63%) of **4a** and 9.8 mg (20%) of **6a**.^111,112^ Data for **4a**: Yellow solid. Mp: 158–159 °C (hexane/EtOAc). ^1^H NMR (400.16 MHz, Acetone-*d*_6_): δ 10.88 (s, 1H), 8.40 (s, 1H), 7.57 (d, *J* = 8.1 Hz, 1H), 7.56 (d, *J* = 8.1 Hz, 2H), 7.44 (dt, *J* = 8.0, 1.0 Hz, 1H), 7.39 (s, 1H), 7.30 (d, *J* = 8.1 Hz, 2H), 7.18 (ddd, *J* = 8.1, 7.0, 1.2 Hz, 1H), 7.10 (ddd, *J* = 8.1, 7.1, 1.1 Hz, 1H), 3.74 (s, 3H), 2.39 (s, 3H), 1.82 (s, 3H) ppm. ^13^C{^1^H} NMR (100.62 MHz, Acetone-*d*_6_): δ 169.0, 166.4, 140.3, 139.2, 137.6, 130.4, 130.2 (2×), 129.4 (2×), 127.8, 126.0, 125.7, 123.1, 121.4, 120.9, 112.4, 108.2, 52.2, 22.8, 21.3 ppm. FTIR (NaCl): ν 3284 (br, m, N–H), 3013 (m, C=C), 2950 (m, C–H), 2866 (m, C–H), 1680 (s, C=O), 1671 (s, C=O), 1507 (s), 1246 (s), 754 (s) cm^–1^. HRMS (ESI) *m*/*z*: [M + H]^+^ calcd for C_21_H_21_N_2_O_3_ 349.1547; found 349.1546.

#### Methyl (Z)-2-Acetamido-3-(2-hexyl-1H-indol-3-yl)acrylate (**4b**)

The reaction of 2-(oct-1-yn-1-yl)aniline^113^ (**1b**) (40.3 mg, 0.20 mmol) and **3** (171.8 mg, 1.20 mmol) in DMF (2.0 mL) in 4 h afforded, after purification by flash-column chromatography (silica gel, solvent A: hexane; solvent B: EtOAc; gradient from 90:10 to 20:80 A/B) 14.0 mg (20%) of **4b** as a brown solid. Mp: 122–123 °C (hexane/EtOAc). ^1^H NMR (400.16 MHz, CDCl_3_): δ 8.65 (s, 1H), 7.53 (s, 1H), 7.46 (d, *J* = 7.7 Hz, 1H), 7.29–7.26 (m, 1H), 7.17–7.06 (m, 2H), 6.95 (s, 1H), 3.87 (s, 3H), 2.61 (t, *J* = 7.8 Hz, 2H), 2.04 (s, 3H), 1.64–1.51 (m, 2H), 1.38–1.20 (m, 6H), 0.87 (t, *J* = 7.0 Hz, 3H) ppm. ^13^C{^1^H} NMR (100.62 MHz, CDCl_3_): δ 168.4, 166.2, 143.6, 135.9, 125.9, 125.9, 122.0, 120.8, 120.6, 120.4, 111.4, 107.4, 52.5, 31.7, 29.4, 29.1, 26.8, 23.5, 22.7, 14.2 ppm. FTIR (NaCl): ν 3271 (s, N–H), 2924 (s, C–H), 2858 (m), 1674 (s, C=O), 1625 (s, C=O), 1460 (s), 1239 (s), 749 (s) cm^–1^. HRMS (ESI) *m*/*z*: [M + H]^+^ calcd for C_20_H_27_N_2_O_3_ 343.2016; found 343.2027.

#### Methyl (Z)-2-Acetamido-3-[2-(*tert*-butyl)-1H-indol-3-yl]acrylate (**4c**)

The reaction of 2-(3,3-dimethylbut-1-yn-1-yl)aniline^113^ (**1c**) (34.7 mg, 0.20 mmol) and **3** (171.8 mg, 1.20 mmol) in 5 h afforded, after purification by flash-column chromatography (silica gel, solvent A: hexane; solvent B: EtOAc; gradient from 90:10 to 20:80 A/B), 27.0 mg (20%) of **4c** as a white solid. Mp: 107–108 °C (hexane/EtOAc). ^1^H NMR (400.16 MHz, CDCl_3_): δ 8.65 (s, 1H), 7.59 (s, 1H), 7.32 (d, *J* = 8.1 Hz, 1H), 7.26 (d, *J* = 7.7 Hz, 2H), 7.14 (ddd, *J* = 8.1, 7.0, 1.3 Hz, 1H), 7.08 (t, *J* = 7.6 Hz, 1H), 6.73 (s, 1H), 3.87 (s, 3H), 1.88 (s, 3H), 1.47 (s, 9H) ppm. ^13^C{^1^H} NMR (100.62 MHz, CDCl_3_): δ 168.8, 165.7, 147.6, 134.3, 126.3, 125.4, 124.3, 121.9, 120.6, 119.8, 111.2, 104.8, 52.6, 33.4, 30.3 (3×), 23.3 ppm. FTIR (NaCl): ν 3321 (s, N–H), 2967 (s, C–H), 1709 (s, C=O), 1674 (s, C=O), 1432 (s), 1257 (s), 752 (s) cm^–1^. HRMS (ESI) *m*/*z*: [M + H]^+^ calcd for C_18_H_23_N_2_O_3_ 315.1703; found 315.1701.

#### Ethyl (Z)-3-(2-Acetamido-3-methoxy-3-oxoprop-1-en-1-yl)-2-(p-tolyl)-1H-indole-1-carboxylate (**5a**)

The reaction of ethyl [2-(*p*-tolylethynyl)phenyl]carbamate^114^ (**2a**) (50.0 mg, 0.18 mmol) and **3** (153.7 mg, 1.07 mmol) for 31 h afforded, after purification by flash-column chromatography (CN-silica gel, solvent A: hexane; solvent B: EtOAc; gradient from 100:0 to 50:50 A/B), 54.2 mg (72%) of **5a** as a yellow solid. Mp: 85–86 °C (hexane/EtOAc). ^1^H NMR (400.16 MHz, Acetone-*d*_6_): δ 8.62 (s, 1H), 8.22 (dt, *J* = 8.4, 0.9 Hz, 1H), 7.52 (d, *J* = 7.9 Hz, 1H), 7.42–7.33 (m, 3H), 7.33–7.23 (m, 3H), 6.89 (s, 1H), 4.21 (q, *J* = 7.1 Hz, 2H), 3.71 (s, 3H), 2.41 (s, 3H), 1.84 (s, 3H), 1.03 (t, *J* = 7.1 Hz, 3H) ppm. ^13^C{^1^H} NMR (100.62 MHz, Acetone-*d*_6_): δ 168.7, 165.8, 151.9, 140.4, 139.0, 137.4, 130.8 (2×), 130.8, 129.2 (2×), 128.8, 127.8, 125.4, 123.9, 123.3, 121.4, 116.7, 116.0, 63.9, 52.4, 22.8, 21.4, 13.8 ppm. FTIR (NaCl): ν 3400–3200 (m, N–H), 2991 (m, C–H), 2955 (m, C–H), 1727 (s, C=O), 1681 (s, C=O), 1508 (m), 1221 (s), 751 (s) cm^–1^. HRMS (ESI) *m*/*z*: [M + H]^+^ calcd for C_24_H_25_N_2_O_5_ 421.1758; found 421.1749.

#### Methyl (Z)-2-Acetamido-3-(2-hexyl-1H-indol-3-yl)acrylate (**4b**) and Ethyl (Z)-3-(2-Acetamido-3-methoxy-3-oxoprop-1-en-1-yl)-2-hexyl-1H-indole-1-carboxylate (**5b**)

The reaction of **2b** (48.9 mg, 0.18 mmol) and **3** (153.7 mg, 1.07 mmol) in 31 h afforded, after purification by flash-column chromatography (CN-silica gel, solvent A: hexane; solvent B: EtOAc; gradient from 95:5 to 0:100 A/B), 29.8 mg (40%) of **5b** and 11.2 mg (18%) of **4b**.

Data for **5b**: Pale yellow solid. Mp: 136–137 °C (hexane/EtOAc). ^1^H NMR (400.16 MHz, CDCl_3_): δ 8.11 (d, *J* = 8.3, 1H), 7.33–7.28 (m, 2H), 7.26 (dd, *J* = 8.3, 1.5 Hz, 1H), 7.20 (td, *J* = 7.4, 1.1 Hz, 1H), 6.87 (s, 1H), 4.51 (q, *J* = 7.1 Hz, 2H), 3.89 (s, 3H), 3.02 (app t, *J* = 7.7 Hz, 2H), 1.91 (s, 3H), 1.66–1.56 (m, 2H), 1.50 (t, *J* = 7.1 Hz, 3H), 1.42–1.26 (m, 6H), 0.88 (t, *J* = 7.0 Hz, 3H) ppm. ^13^C{^1^H} NMR (100.62 MHz, CDCl_3_): δ 168.3, 165.3, 151.6, 142.7, 136.1, 126.9, 126.8, 124.3, 123.3, 122.0, 119.8, 116.0, 113.9, 63.6, 52.8, 31.7, 29.9, 29.5, 28.0, 23.3, 22.8, 14.4, 14.2 ppm. FTIR (NaCl): ν 3263 (m, N–H), 2954 (w, C–H), 2928(w, C–H), 2856 (w, C–H), 1735 (s, C=O), 1457 (m), 1325 (m), 1223 (m), 1122 (w), 756 (w) cm^–1^. HRMS (ESI) *m*/*z*: [M + H]^+^ calcd for C_23_H_31_N_2_O_5_ 415.2227; found 415.2238.

### Preparation of 2-Aryldehydrotryptophans **4a** and **4d**–**n** from *o*-Iodoaryl Carbamates **7**, Alkynes **8**, and Alkene **3** (Table 2)

In a typical experiment, to a solution of an *o*-iodoaryl carbamate **7** (0.21 mmol), an alkyne **8** (0.41 mmol), and **3** (177 mg, 1.24 mmol) in DMF (2.1 mL, previously degassed with freeze–thaw cycles under argon) were added PdCl_2_ (1.8 mg, 0.010 mmol), polymer-bound PPh_3_ (13.1 mg, 1.6 mmol/g PPh_3_ loading, 0.021 mmol of PPh_3_), CuI (7.9 mg, 0.041 mmol), and Et_3_N (0.129 mL, 0.93 mmol). The resulting mixture was stirred at 60 °C under argon. After reaction completion, air was allowed into the system, and the mixture was stirred at 120 °C. Then, a saturated aqueous solution of NaHCO_3_ was added, and the mixture was extracted with EtOAc (3×). The combined organic layers were dried (Na_2_SO_4_) and filtered over silica gel (230–400 mesh). After removal of the solvent by evaporation, the residue was dissolved in methanol (4.3 mL) in the presence of *tert*-butylamine (0.64 mL, 6.1 mmol), and the mixture was heated at reflux. The solvent was removed under reduced pressure, and the crude product was purified by flash-column chromatography (silica gel, solvent A: hexane; solvent B: EtOAc; gradient from 20:80 to 0:100 A/B) to afford products **4a** and **4d**–**n**. Reaction times are those given in Table 2.

#### Methyl (Z)-2-Acetamido-3-[2-(p-tolyl)-1H-indol-3-yl]acrylate (**4a**)

Starting from ethyl (2-iodophenyl)carbamate (**7a**)^27,107^ (60 mg, 0.21 mmol) and *p*-tolylacetylene (**8a**) (0.052 mL, 0.41 mmol) afforded 44 mg (62%) of **4a**.

#### Methyl (Z)-2-Acetamido-3-[2-(4-methoxyphenyl)-1H-indol-3-yl]acrylate (**4d**)

Starting from **7a**(27,107) (60 mg, 0.21 mmol) and 1-ethynyl-4-methoxybenzene (**8d**) (55 mg, 0.41 mmol) afforded 55 mg (73%) of **4d** as a solid. Mp: 165–166 °C (hexane/EtOAc). ^1^H NMR (400.16 MHz, CDCl_3_): δ 8.84 (s, 1H), 7.53–7.48 (m, 2H), 7.44 (d, *J* = 8.5 Hz, 2H), 7.22 (d, *J* = 7.6 Hz, 1H), 7.18–7.09 (m, 2H), 6.93 (d, *J* = 8.5 Hz, 2H), 6.89 (s, 1H), 3.84 (s, 3H), 3.83 (s, 3H), 1.85 (s, 3H) ppm. ^13^C{^1^H} NMR (100.62 MHz, CDCl_3_): δ 168.0, 166.0, 160.1, 139.3, 136.3, 129.9 (2×), 126.9, 125.7, 124.6, 122.8, 122.7, 120.8, 120.2, 114.6 (2×), 111.5, 107.5, 55.5, 52.6, 23.3 ppm. FTIR (NaCl): ν 3500–3100 (br, s, N–H), 3012 (m, C–H), 2950 (m, C–H), 2837 (m, C–H), 1706 (s, C=O), 1670 (s, C=O), 1611 (m), 1502 (s), 1250 (s), 752 (s) cm^–1^. HRMS (ESI) *m*/*z*: [M + H]^+^ calcd for C_21_H_21_N_2_O_4_ 365.1496; found 365.1489.

#### Methyl (Z)-4-[3-(2-Acetamido-3-methoxy-3-oxoprop-1-en-1-yl)-1H-indol-2-yl]benzoate (**4e**)

Starting from **7a**(27,107) (60.0 mg, 0.21 mmol) and methyl 4-ethynylbenzoate (**8e**) (66.0 mg, 0.41 mmol) afforded 48.0 mg (59%) of **4e** as a yellow solid. Mp: 111–112 °C (hexane/EtOAc). ^1^H NMR (400.16 MHz, DMSO-*d*_6_): δ 12.05 (s, 1H), 9.38 (s, 1H), 8.10 (d, *J* = 8.5 Hz, 2H), 7.74 (d, *J* = 8.5 Hz, 2H), 7.47 (d, *J* = 8.2 Hz, 1H), 7.44 (d, *J* = 8.1 Hz, 1H), 7.24 (s, 1H), 7.22 (ddd, *J* = 8.1, 7.1, 1.1 Hz, 1H), 7.09 (ddd, *J* = 8.1, 7.1, 1.1 Hz, 1H), 3.89 (s, 3H), 3.71 (s, 3H), 1.70 (s, 3H). ^13^C{^1^H} NMR (100.62 MHz, DMSO-*d*_6_): δ 168.5, 165.9, 165.4, 137.3, 136.8, 136.4, 129.5 (2×), 128.8, 128.5 (2×), 126.2, 125.4, 124.4, 122.8, 120.5, 120.1, 111.9, 108.2, 52.3, 51.9, 22.3 ppm. FTIR (NaCl): ν 3500–3100 (br, m, N–H), 3009 (m, C–H), 2949 (m, C–H), 1713 (s, C=O), 1670 (s, C=O), 1435 (m), 1276 (s), 751 (s) cm^–1^. HRMS (ESI) [M + H]^+^: calcd for C_22_H_21_N_2_O_5_ 393.1445; found 393.1433.

#### Methyl (Z)-2-Acetamido-3-[5-bromo-2-(p-tolyl)-1H-indol-3-yl]acrylate (**4f**)

Starting from **7b**(27,106) (200 mg, 0.54 mmol) and **8a** (0.137 mL, 1.08 mmol) afforded 157 mg (68%) of **4f** as a yellow solid. Mp: 163–164 °C (hexane/EtOAc). ^1^H NMR (400.16 MHz, CD_3_OD): δ 7.62 (d, *J* = 1.9 Hz, 1H), 7.49 (d, *J* = 8.0 Hz, 2H), 7.49 (s, 1H), 7.33 (d, *J* = 8.6 Hz, 1H), 7.30 (d, *J* = 8.0 Hz, 2H), 7.26 (dd, *J* = 8.6, 1.8 Hz, 1H), 3.80 (s, 3H), 2.40 (s, 3H), 1.91 (s, 3H) ppm. ^13^C{^1^H} NMR (100.62 MHz, CD_3_OD): δ 172.4, 167.4, 143.4, 140.3, 136.8, 130.5 (2×), 130.1, 129.9 (2×), 129.4, 129.1, 126.0, 124.2, 123.9, 114.4, 114.2, 107.7, 52.8, 22.5, 21.3 ppm. FTIR (NaCl): ν 3400–3100 (br, m, N–H), 3013 (w, C=C), 2949 (w, C–H), 1684 (s, C=O), 1672 (s, C=O), 1433 (s), 1245 (s), 753 (s) cm^–1^. HRMS (ESI) *m*/*z*: [M + H]^+^ calcd for C_21_H_20_^79^BrN_2_O_3_ 427.0652; found 427.0637.

#### Methyl (Z)-2-Acetamido-3-[6-bromo-2-(p-tolyl)-1H-indol-3-yl]acrylate (**4g**)

Starting from **7c**(106) (200 mg, 0.54 mmol) and **8a** (0.137 mL, 1.08 mmol) afforded 144 mg (62%) of **4g** as a yellow solid. Mp: 157–158 °C (hexane/EtOAc). ^1^H NMR (400.16 MHz, CD_3_OD): δ 7.50 (s, 1H), 7.46 (s, 1H), 7.40 (d, *J* = 7.9 Hz, 2H), 7.31 (d, *J* = 8.6 Hz, 1H), 7.16 (d, *J* = 7.9 Hz, 2H), 7.12 (d, *J* = 8.6 Hz, 1H), 3.72 (s, 3H), 2.28 (s, 3H), 1.76 (s, 3H) ppm. ^13^C{^1^H} NMR (100.62 MHz, CD_3_OD): δ 172.1, 167.4, 142.3, 140.0, 138.8, 130.4 (2×), 130.1, 129.6 (2×), 129.1, 126.8, 124.3, 124.0), 122.6, 116.6, 115.4, 108.0, 52.8, 22.5, 21.4 ppm. FTIR (NaCl): ν 3500–3100 (br, m, N–H), 3017 (m, C=C), 2953 (m, C–H), 1685 (s, C=O), 1671 (s, C=O), 1434 (m), 1246 (s), 757 (s) cm^–1^. HRMS (ESI) *m*/*z*: [M + H]^+^ calcd for C_21_H_20_^79^BrN_2_O_3_ 427.0652; found 427.0644.

#### Methyl (Z)-3-(2-Acetamido-3-methoxy-3-oxoprop-1-en-1-yl)-2-(p-tolyl)-1H-indole-5-carboxylate (**4h**)

Starting from **7d**(107) (200 mg, 0.57 mmol) and **8a** (0.145 mL, 1.15 mmol) afforded 115 mg (49%) of **4h** as a yellow solid. Mp: 244–245 °C (hexane/EtOAc). ^1^H NMR (400.16 MHz, DMSO-*d*_6_): δ 12.27 (s, 1H), 9.56 (s, 1H), 8.22 (d, *J* = 1.7 Hz, 1H), 7.82 (dd, *J* = 8.5, 1.7 Hz, 1H), 7.53 (d, *J* = 8.5 Hz, 1H), 7.52 (d, *J* = 7.9 Hz, 2H), 7.39 (d, *J* = 7.9 Hz, 2H), 7.18 (s, 1H), 3.85 (s, 3H), 3.71 (s, 3H), 2.39 (s, 3H), 1.85 (s, 3H) ppm. ^13^C{^1^H} NMR (100.62 MHz, DMSO-*d*_6_): δ 168.8, 167.0, 165.6, 141.5, 139.1, 138.6, 129.5 (2×), 128.7 (2×), 128.3, 125.6, 124.8, 124.0, 123.5), 123.0, 121.3, 111.6), 107.6, 51.9, 51.7, 22.2, 20.9 ppm. FTIR (NaCl): ν 3600–2900 (s, br, N–H), 3040 (w, C–H), 2963 (w, C–H), 2257 (w), 2126 (w), 1708 (s, C=O), 1664 (s, C=O), 1243 (m), 1025 (s) cm^–1^. HRMS (ESI) *m*/*z*: [M + H]^+^ calcd for C_23_H_23_N_2_O_5_ 407.1602; found 407.1601.

#### Methyl (Z)-2-Acetamido-3-[5-methoxy-2-(p-tolyl)-1H-indol-3-yl]acrylate (**4i**)

Starting from **7e** (200 mg, 0.62 mmol) and **8a** (0.158 mL, 1.25 mmol) afforded 130 mg (55%) of **4i** as a solid. Mp: 179–180 °C (hexane/EtOAc). ^1^H NMR (400.16 MHz, CD_3_OD): δ 7.54 (s, 1H), 7.46 (d, *J* = 8.0 Hz, 2H), 7.29 (d, *J* = 8.8 Hz, 1H), 7.24 (d, *J* = 8.0 Hz, 2H), 6.96 (d, *J* = 2.4 Hz, 1H), 6.81 (dd, *J* = 8.8, 2.4 Hz, 1H), 3.79 (s, 3H), 3.78 (s, 3H), 2.35 (s, 3H), 1.87 (s, 3H) ppm. ^13^C{^1^H} NMR (100.62 MHz, CD_3_OD): δ 172.4, 167.5, 155.9, 142.3, 139.6, 133.1, 130.7, 130.4 (2×), 130.2, 129.6 (2×), 128.5, 123.4, 113.6, 113.3, 107.9, 103.5, 56.2, 52.7, 22.7, 21.3 ppm. FTIR (NaCl): ν 3400–3100 (br, s, N–H), 3010 (m, C–H), 2950 (m, C–H), 2840 (s, C–H), 1701 (s, C=O), 1668 (s, C=O), 1624 (s), 1488 (s), 1256 (s), 754 (s) cm^–1^. HRMS (ESI) *m*/*z*: [M + H]^+^ calcd for C_22_H_23_N_2_O_4_ 379.1652; found 379.1660.

#### Methyl (Z)-2-Acetamido-3-[2-(3-chlorophenyl)-1H-indol-3-yl]acrylate (**4j**)

Starting from **7a**(27,107) (75.0 mg, 0.26 mmol) and **8d** (0.063 mL, 0.52 mmol) afforded 55 mg (58%) of **4j** as a foam. ^1^H NMR (400.16 MHz, CD_3_OD, 323 K): δ 7.90 (s, 1H), 7.55–7.53 (m, 2H), 7.50 (d, *J* = 8.1 Hz, 1H), 7.45–7.41 (m, 3H), 7.20 (t, *J* = 7.6 Hz, 1H), 7.11 (t, *J* = 7.5 Hz, 1H), 3.82 (s, 3H), 1.80 (s, 3H) ppm. ^13^C{^1^H} NMR (100.62 MHz, CD_3_OD, 323 K): δ 172.1, 167.4, 139.3, 138.3, 135.7, 135.6, 131.4, 129.3 (2×), 128.6, 128.3, 127.8, 124.7, 123.9, 121.5 (2×), 112.8, 109.1, 52.8, 22.4 ppm. FTIR (NaCl): ν 3263 (s, N–H), 3058 (w), 3001 (w), 2950 (w), 1704 (s, C=O), 1669 (s, C=O), 1435 (s), 1243 (s) cm^–1^. HRMS (ESI) *m*/*z*: [M + H]^+^ calcd for C_20_H_18_ClN_2_O_3_ [(M+H)^+^] 369.1000; found 369.1000.

#### Methyl (Z)-2-Acetamido-3-[2-(3-fluorophenyl)-1H-indol-3yl]acrylate (**4k**)

Starting from **7a**(27,107) (75.0 mg, 0.26 mmol) and **8e** (0.060 mL, 0.52 mmol) afforded 45.0 mg (50%) of **4k** as a foam. ^1^H NMR (400.16 MHz, CD_3_OD, 323 K): δ 7.90 (s, 1H), 7.53–7.41 (m, 4H), 7.38 (ddd, *J* = 10.0, 2.6, 1.5 Hz, 1H), 7.23–7.08 (m, 3H), 3.82 (s, 3H), 1.80 (s, 3H) ppm. ^13^C{^1^H} NMR (100.62 MHz, CD_3_OD, 323 K): δ 172.2, 167.4, 164.2 (^1^*J*_*CF*_ = 245.1 Hz), 139.5, 138.3, 135.9 (^3^*J*_*CF*_ = 8.3 Hz), 131.7 (^3^*J*_*CF*_ = 8.6 Hz), 128.8, 127.8, 125.8 (^4^*J*_*CF*_ = 3.0 Hz), 124.7, 123.9, 121.5 (2×), 116.3 (^2^*J*_*CF*_ = 18.3 Hz), 116.1 (^2^*J*_*CF*_ = 16.8 Hz), 112.7, 109.0, 52.8, 22.4 ppm. FTIR (NaCl): ν 3260 (s, N–H), 3050 (w), 3029 (w), 2950 (w), 1698 (s, C=O), 1669 (s, C=O), 1436 (s), 1270 (s), 1243 (s) cm^–1^. HRMS (ESI) *m*/*z*: [M + H]^+^ calcd for C_20_H_18_FN_2_O_3_ 353.1298; found 353.1296.

#### Methyl (Z)-2-Acetamido-3-[2-(3-methoxyphenyl)-1H-indol-3-yl]acrylate (**4l**)

Starting from **7a**(27,107) (75.0 mg, 0.26 mmol) and 1-ethynyl-3-methoxybenzene (**8f**) (0.065 mL, 0.52 mmol) afforded 55.0 mg (59%) of **4l** as a foam. ^1^H NMR (400.16 MHz, DMSO-*d*_6_, 333 K): δ 9.23 (s, 1H), 7.50–7.42 (m, 3H), 7.32 (s, 1H), 7.23–7.16 (m, 3H), 7.09 (d, *J* = 8.1 Hz, 1H), 7.08–6.99 (m, 1H), 3.84 (s, 3H), 3.72 (s, 3H), 1.77 (s, 3H) ppm. ^13^C{^1^H} NMR (100.62 MHz, DMSO-*d*_6_, 333 K): δ 168.3, 165.4, 159.2, 138.9, 136.3, 132.9, 129.6, 126.0, 125.6, 124.2, 122.0, 120.8, 120.3, 119.7, 114.0, 113.8, 111.5, 107.0, 55.0, 51.6, 22.1 ppm. FTIR (NaCl): ν 3274 (s, N–H), 3058 (w), 3006 (w), 2951 (w), 2836 (w), 1708 (s, C=O), 1670 (s, C=O), 1491 (s), 1435 (s), 1240.0 (s), 1042 (w) cm^–1^. HRMS (ESI) *m*/*z*: [M + H]^+^ calcd for C_21_H_21_N_2_O_4_ 365.1480; found 365.1496.

#### Methyl (Z)-2-Acetamido-3-[2-(3,4-difluorophenyl)-1H-indol-3-yl]acrylate (**4m**)

Starting from **7a**(27,107) (75.0 mg, 0.26 mmol) and 4-ethynyl-1,2-difluorobenzene (**8g**) (0.062 mL, 0.52 mmol) afforded 55.0 mg (58%) of **4m** as a foam. ^1^H NMR (400.16 MHz, DMSO-*d*_6_, 333 K): δ 9.22 (s, 1H), 7.69–7.54 (m, 2H), 7.45 (dd, *J* = 7.6, 7.0 Hz, 3H), 7.26 (s, 1H), 7.20 (t, *J* = 7.6 Hz, 1H), 7.09 (t, *J* = 8.1 Hz, 1H), 3.73 (s, 3H), 1.72 (s, 3H) ppm. ^13^C{^1^H} NMR (100.62 MHz, DMSO-*d*_6_, 333 K): δ 168.1, 165.2, 146.3 (^1^*J*_*CF*_ = 245.4 Hz), 149.2 (^1^*J*_*CF*_ = 246.0 Hz), 136.3 (^3^*J*_*CF*_ = 8.5 Hz), 129.4, 129.3, 126.1, 125.3 (2×), 124.3, 122.4, 120.2, 119.9, 117.8 (^2^*J*_*CF*_ = 17.4 Hz), 116.9 (^2^*J*_*CF*_ = 18.1 Hz), 111.6, 107.5, 51.6, 22.0 ppm. FTIR (NaCl): ν 3264 (s, N–H), 3058 (w), 3019 (w), 2952 (w), 1707 (s, C=O), 1669 (s, C=O), 1513 (s), 1456 (s), 1436 (s), 1277 (s), 1242 (s), 1202 (w), 1118 (w) cm^–1^. HRMS (ESI) *m*/*z*: [M + H]^+^ calcd for C_20_H_17_F_2_N_2_O_3_ 371.1184; found 371.1201.

#### Methyl (Z)-2-Acetamido-3-[2-(3,4-thiophen-2-yl)-1H-indol-3-yl]acrylate (**4n**)

Starting from **7a**(27,107) (75.0 mg, 0.26 mmol) and 3-ethynylthiophene (**8h**) (0.051 mL, 0.52 mmol) afforded 45.0 mg (51%) of **4n** as a foam. ^1^H NMR (400.16 MHz, DMSO-*d*_6_, 333 K): δ 9.22 (s, 1H), 7.73 (s, 2H), 7.53–7.33 (m, 4H), 7.17 (q, *J* = 7.3 Hz, 1H), 7.06 (q, *J* = 7.2 Hz, 1H), 3.74 (s, 3H), 1.82 (s, 3H) ppm. ^13^C{^1^H} NMR (100.62 MHz, DMSO-*d*_6_, 333 K): δ 168.5, 165.4, 136.1, 134.8), 132.4, 127.0, 126., 125.8, 125.1, 124.4, 124.2, 121.2, 120.3, 119.7, 111.3, 106.7, 51.6, 22.2 ppm. FTIR (NaCl): ν 3275 (s, N–H), 3105 (w), 3055 (w), 3008 (w), 2950 (w), 1704 (s, C=O), 1669 (s, C=O), 1514 (w), 1434 (s), 1243 (s), 1131 (w) cm^–1^. HRMS (ESI) *m*/*z*: [M + H]^+^ calcd for C_18_H_17_N_2_O_3_S 341.0938; found 341.0954.

### Preparation of 2-Alkyldehydrotryptophans **4b**, **4o**, and **4p** from *o*-Alkynylaryl Carbamates **2** and Alkene **3** (Scheme 2)

The following procedure is representative: To a solution of **2** (0.20 mmol) and **3** (170.9 mg, 1.19 mmol) in DMF (2.0 mL) were added PdCl_2_(PPh_3_)_2_ (14.0 mg, 0.020 mmol) and KI (16.5 mg, 0.099 mmol). The resulting mixture was stirred at 100 °C (**2b**, 45 h; **2p**, 45 h) or 120 °C (**2o**, 44 h) with the flask opened to air. After reaction completion, water was added, and the mixture was extracted with EtOAc (3×). The combined organic layers were dried (Na_2_SO_4_) and filtered through a pad of Celite, and the solvent was evaporated. The residue was dissolved in MeOH (4.2 mL) in the presence of *tert*-butylamine (0.63 mL, 6.0 mmol), and the mixture was heated to 90 °C (**2b**, 16 h; **2o**, 18 h; **2p**, 45 h). The solvent was removed under reduced pressure, and the crude product was purified by flash-column chromatography (silica gel, hexane/EtOAc gradient) to afford product **4b**, **4o**, or **4p**.

#### Methyl (Z)-2-Acetamido-3-(2-hexyl-1H-indol-3-yl)acrylate (**4b**)

Starting from **2b** (60.0 mg, 0.22 mmol), purification by flash-column chromatography (silica gel, solvent A: hexane; solvent B: EtOAc; gradient from 90:10 to 20:80 A/B) afforded 33.7 mg (45%) of **4b**.

#### Methyl (Z)-2-Acetamido-3-(2-cyclohexyl-1H-indol-3-yl)acrylate (**4o**)

Starting from **2o** (54.0 mg, 0.20 mmol), purification by flash-column chromatography (silica gel, solvent A: hexane; solvent B: EtOAc; gradient from 90:10 to 0:100 A/B) afforded 43.8 mg (65%) of **4o** as a yellow foam. ^1^H NMR (400.16 MHz, CD_3_OD): δ 7.67 (s, 1H), 7.39 (d, *J* = 8.0 Hz, 1H), 7.32 (d, *J* = 8.0 Hz, 1H), 7.09 (ddd, *J* = 8.1, 7.1, 1.2 Hz, 1H), 7.02 (ddd, *J* = 8.1, 7.1, 1.2 Hz, 1H), 3.82 (s, 3H), 2.94 (tt, *J* = 12.2, 3.3 Hz, 1H), 1.97 (s, 3H), 1.93–1.85 (m, 4H), 1.83–1.77 (m, 1H), 1.65 (qd, *J* = 12.8, 3.6 Hz, 2H), 1.48 (qt, *J* = 12.8, 3.2 Hz, 2H), 1.43–1.29 (m, 1H) ppm. ^13^C{^1^H} NMR (100.62 MHz, CD_3_OD): δ 172.8, 167.8, 149.4, 137.8, 129.9, 127.1, 122.5, 121.9, 121.3, 121.0, 112.2, 106.5, 52.7, 37.9, 33.8 (2×), 27.6 (2×), 27.1, 22.6 ppm. FTIR (NaCl): ν 3281 (s, N–H), 2930 (s, C–H), 2853 (s), 1670 (s), 1625 (s) 1455 (s), 1434 (s), 1247 (s), 1223 (s), 751 (s) cm^–1^. HRMS (ESI) *m*/*z*: [M + H]^+^ calcd for C_20_H_25_N_2_O_3_ 341.1860; found 341.1859.

#### Methyl (Z)-2-Acetamido-3-[2-(cyclohex-1-en-yl)-1H-indol-3-yl]acrylate (**4p**)

Starting from **2p** (60.0 mg, 0.22 mmol), purification by flash-column chromatography (silica gel, solvent A: hexane; solvent B: EtOAc; gradient from 90:10 to 0:100 A/B) afforded 51.7 mg (68%) of **4p** as a yellow foam. ^1^H NMR (400.16 MHz, CDCl_3_, 323 K): δ 8.44 (s, 1H), 7.48 (s, 1H), 7.41 (d, *J* = 8.1 Hz, 1H), 7.29 (d, *J* = 7.1 Hz, 1H), 7.16 (t, *J* = 7.8 Hz, 1H), 7.10 (t, *J* = 7.4 Hz, 1H), 6.92 (s, 1H), 6.14–6.10 (m, 1H), 3.87 (s, 3H), 2.40–2.34 (m, 2H), 2.32–2.24 (m, 2H), 1.97 (s, 3H), 1.83–1.74 (m, 2H), 1.73–1.67 (m, 2H) ppm. ^13^C{^1^H} NMR (100.62 MHz, CDCl_3_, 323 K): δ 168.3, 165.9, 141.6, 135.4, 132.8, 129.3, 128.7, 126.3, 122.7, 122.5, 120.6, 120.2, 111.4, 106.8, 52.6, 27.4, 26.0, 23.5, 22.6, 21.9 ppm. FTIR (NaCl): ν 3400–3200 (s, N–H), 3011 (w), 2936 (w), 2865 (w), 2829 (w), 1683 (s, C=O), 1666 (s, C=O), 1495 (w), 1435 (w), 1247 (s) cm^–1^. HRMS (ESI) *m*/*z*: [M + H]^+^ calcd for C_20_H_23_N_2_O_3_ 339.1703; found 339.1705.

### Preparation of Dehydrotryptophans **10** from *N*-PMB-*o*-Alkynylanilines **9** (Table 3 and Scheme 3)

In a typical procedure, to a solution of an *o*-alkynylaniline **9** (0.22 mmol) and **3** (188 mg, 1.32 mmol) in DMF (2.1 mL) were added PdCl_2_(PPh_3_)_2_ (15.4 mg, 0.022 mmol), TPPO (6.1 mg, 0.022 mmol), and KI (18.2 mg, 0.11 mmol), and the mixture was stirred at 100 °C for 5–24 h (see Table 3 for details) with the flask opened to air. After reaction completion, a saturated aqueous solution of NaHCO_3_ was added, and the mixture was extracted with EtOAc (3×). The combined organic layers were dried (Na_2_SO_4_), and the solvent was evaporated. The residue was purified by flash-column chromatography (silica gel, solvent A: hexane; solvent B: EtOAc; gradient from 20:80 to 80:20 A/B) to afford products **10**.

#### Methyl (Z)-2-Acetamido-3-(2-hexyl-1-(4-methoxybenzyl)-1H-indol-3-yl)acrylate (**10a**)

Following the procedure for the preparation of *N*-PMB-dehydrotryptophans **10**, starting from **9a** (70 mg, 0.22 mmol) afforded 40 mg (40%) of **10a** as a pale brown solid. Mp: 128–129 °C (hexane/EtOAc). ^1^H NMR (400.16 MHz, CD_3_OD, 323 K): δ 7.64 (s, 1H), 7.49 (d, *J* = 7.5 Hz, 1H), 7.27 (d, *J* = 7.8 Hz, 1H), 7.13–7.05 (m, 2H), 6.93 (d, *J* = 8.3 Hz, 2H), 6.81 (d, *J* = 8.6 Hz, 2H), 5.35 (s, 2H), 3.82 (s, 3H), 3.73 (s, 3H), 2.83 (t, *J* = 7.8 Hz, 2H), 1.97 (s, 3H), 1.54–1.43 (m, 2H), 1.39–1.30 (m, 2H), 1.30–1.20 (m, 4H), 0.86 (t, *J* = 7.1 Hz, 3H) ppm. ^13^C{^1^H} NMR (100.62 MHz, CD_3_OD, 323 K): δ 172.5, 167.8, 160.6, 146.1, 138.8, 130.9, 129.4, 128.4 (2×), 126.8, 122.9, 122.4, 121.8, 121.5, 115.3 (2×), 111.3, 109.0, 55.8, 52.7, 47.2, 32.5, 30.9, 30.0, 26.1, 23.4, 22.7, 14.3 ppm. FTIR (NaCl): ν 3400–3200 (br, m, N–H), 2933 (s, C–H), 1711 (s, C=O), 1678 (s), 1514 (s), 1250 (s) cm^–1^. HRMS (ESI) *m*/*z*: [M + H]^+^ calcd for C_28_H_35_N_2_O_4_ 463.2591; found 463.2595.

#### Methyl (Z)-2-Acetamido-3-[2-(acetoxymethyl)-1-(4-methoxybenzyl)-1H-indol-3-yl]acrylate (**10b**)

Following the procedure for the preparation of *N*-PMB-dehydrotryptophans **10**, starting from **9b** (50 mg, 0.16 mmol) afforded 30 mg (41%) of **10b** as a solid. Mp: 139–140 °C (hexane/EtOAc). ^1^H NMR (400.16 MHz, CD_3_OD, 323 K): δ 7.83 (s, 1H), 7.72 (s, 1H), 7.52 (d, *J* = 8.1 Hz, 1H), 7.34 (d, *J* = 8.3 Hz, 1H), 7.20 (ddd, *J* = 8.3, 7.6, 1.2 Hz, 1H), 7.12 (app t, *J* = 7.6 Hz, 1H), 6.94 (d, *J* = 8.6 Hz, 2H), 6.82 (d, *J* = 8.6 Hz, 2H), 5.43 (s, 2H), 5.30 (s, 2H), 3.83 (s, 3H), 3.73 (s, 3H), 1.93 (s, 3H), 1.83 (s, 3H) ppm. ^13^C{^1^H} NMR (100.62 MHz, CD_3_OD, 323 K): δ 172.5, 172.0, 167.3, 160.6, 139.1, 136.2, 130.8, 128.4 (2×), 127.4, 126.4, 125.7, 124.3, 122.2, 121.8, 115.2 (2×), 112.1, 111.7, 56.7, 55.8, 52.8, 47.6, 22.6, 20.4 ppm. FTIR (NaCl): ν 3500–3200 (br, m, N–H), 3004 (w, C–H), 2947 (w, C–H), 1731 (s, C=O), 1681 (s, C=O), 1514 (s), 1246 (s) cm^–1^. HRMS (ESI) *m*/*z*: [M + H]^+^ calcd for C_25_H_27_N_2_O_6_ 451.1864, found 451.1870.

#### (Z)-2-Acetamido-3-[2-cyclohexyl-1-(4-methoxybenzyl)-1H-indol-3-yl]acrylate (**10c**)

Following the procedure for the preparation of *N*-PMB-dehydrotryptophans **10**, starting from **9c** (70 mg, 0.22 mmol) afforded 50 mg (50%) of **10c** as a white solid. Mp: 176–177 °C (hexane/EtOAc). ^1^H NMR (400.16 MHz, CDCl_3_): δ 7.60 (s, 1H), 7.33 (d, *J* = 7.5 Hz, 1H), 7.24–7.19 (m, 1H), 7.16–7.07 (m, 2H), 6.93 (d, *J* = 8.3 Hz, 2H), 6.81 (d, *J* = 8.4 Hz, 2H), 6.76 (br s, 1H), 5.34 (s, 2H), 3.89 (s, 3H), 3.76 (s, 3H), 2.93–2.80 (m, 1H), 1.93 (s, 3H), 1.84–1.78 (m, 2H), 1.77–1.68 (m, 5H), 1.30–1.21 (m, 3H) ppm. ^13^C{^1^H} NMR (100.62 MHz, CDCl_3_): δ 168.6, 165.8, 159.1, 146.2, 136.7, 129.5, 127.3 (2×), 125.5, 125.1, 123.6, 121.9, 120.6, 120.2, 114.4 (2×), 110.2, 106.2, 55.4, 52.6, 46.8, 37.7, 32.2 (2×), 27.1 (2×), 25.9, 23.4 ppm. FTIR (NaCl): ν 3400–3100 (w, N–H), 3009 (m, C–H), 2931 (s, C–H), 2852 (m, C–H), 1716 (s, C=O), 1679 (s, C=O), 1511 (s), 1249 (s), 750 (s) cm^–1^. HRMS (ESI) *m*/*z*: [M + H]^+^ calcd for C_28_H_33_N_2_O_4_ 461.2435; found 461.2453.

#### Methyl (Z)-2-Acetamido-3-[2-(*tert*-butyl)-1-(4-methoxybenzyl)-1H-indol-3-yl]acrylate (**10d**) and 2-(*tert*-Butyl)-1-(4-methoxybenzyl)-1H-indole (**11d**)

Following the procedure for the preparation of *N*-PMB-dehydrotryptophans **10**, starting from **9d** (50 mg, 0.17 mmol) afforded 61 mg (82%) of **10d** and 17 mg (17%) of **11d**. Data for **10d**: Yellow solid. Mp: 82–83 °C (hexane/EtOAc). ^1^H NMR (400.16 MHz, CDCl_3_): δ 7.63 (s, 1H), 7.25–7.21 (m, 1H), 7.10–7.02 (m, 2H), 7.02–6.96 (m, 1H), 6.80 (app. s, 4H), 6.65 (s, 1H), 5.59 (s, 2H), 3.89 (s, 3H), 3.75 (s, 3H), 1.82 (s, 3H), 1.50 (s, 9H) ppm. ^13^C{^1^H} NMR (100.62 MHz, CDCl_3_): δ 168.5, 165.6, 158.8, 146.2, 137.7, 129.6, 127.3, 126.8 (2×), 125.6, 125.3, 122.2, 120.5, 119.5, 114.2 (2×), 110.6, 105.7, 55.3, 52.6, 48.9, 34.7, 32.0 (3×), 23.3 ppm. FTIR (NaCl): ν 3400–3100 (m, N–H), 3000 (m, C–H), 2962 (m, C–H), 1716 (s, C=O), 1678 (s, C=O), 1510 (s), 1470 (s), 1249 (s), 753 (s) cm^–1^. HRMS (ESI) *m*/*z*: [M + H]^+^ calcd for C_26_H_31_N_2_O_4_ 435.2278; found 435.2279. Data for **11d:** White solid. Mp: 135–136 °C (hexane/EtOAc). ^1^H NMR (400.16 MHz, CDCl_3_): δ 7.62–7.54 (m, 1H), 7.10–6.98 (m, 3H), 6.85–6.75 (m, 4H), 6.41 (d, *J* = 0.8 Hz, 1H), 5.56 (s, 2H), 3.76 (s, 3H), 1.44 (s, 9H) ppm. ^13^C{^1^H} NMR (100.62 MHz, CDCl_3_): δ 158.7, 149.3, 138.4, 130.3, 127.6, 126.9 (2×), 121.2, 120.1, 119.7, 114.1 (2×), 110.1, 98.7, 55.3, 48.2, 32.6, 30.8 (3×) ppm. FTIR (NaCl): ν 3041 (w, C–H), 2964 (s, C–H), 2871 (w, C–H), 1607 (w), 1512 (s), 1466 (s), 1244 (s), 1175 (m), 738 (m) cm^–1^. HRMS (ESI) *m*/*z*: [M + H]^+^ calcd for C_20_H_24_NO 294.1852; found 294.1856.

#### Methyl (Z)-2-Acetamido-3-{2-[1-(*tert*-butyldiphenylsilyloxyl)-2-methylpropan-2-yl]-1-(4-methoxybenzyl)-1H-indol-3-yl}acrylate (**10e**)

Following the procedure for the preparation of *N*-PMB-dehydrotryptophans **10**, starting from **9e** (50 mg, 0.091 mmol) afforded 45 mg (72%) of **10e** as a yellow oil. ^1^H NMR (400.16 MHz, CD_3_OD, 323 K): δ 7.77 (s, 1H), 7.46–7.42 (m, 4H), 7.41–7.32 (m, 3H), 7.24–7.22 (m, 5H), 7.06–6.95 (m, 3H), 6.72–6.67 (m, 4H), 5.36 (s, 2H), 3.88 (s, 2H), 3.80 (s, 3H), 3.69 (s, 3H), 1.73 (s, 3H), 1.49 (s, 6H), 1.01 (s, 9H) ppm. ^13^C{^1^H} NMR (100.62 MHz, CD_3_OD, 323 K): δ 172.3, 167.2, 160.2, 144.8, 139.4, 136.8 (4×), 134.7, 131.2, 130.8 (2×), 128.7 (4×), 127.8 (2×), 127.7, 126.9, 126.8, 123.1, 121.1, 120.7, 115.1 (2×), 115.0, 111.2, 109.5, 72.8, 55.8, 52.7, 49.9, 41.8, 27.6 (2×), 27.5 (3×), 22.4, 20.1 ppm. FTIR (NaCl): ν 3074 (w, C–H), 2994 (m, C–H), 2922 (m, C–H), 2858 (m, C–H), 1697 (s, C=O), 1683 (s, C=O), 1513 (s), 1250 (s), 1111 (s), 1086 (s) cm^–1^. HRMS (ESI) *m*/*z*: [M + H]^+^ calcd for C_42_H_49_N_2_O_5_Si 689.3405, found 689.3379.

#### Methyl (Z)-2-Acetamido-3-[2-(2-hydroxypropan-2-yl)-1-(4-methoxybenzyl)-1H-indol-3-yl]acrylate (**10f**)

Following the procedure for the preparation of *N*-PMB-dehydrotryptophans **10**, starting from **9f** (50 mg, 0.17 mmol) afforded 30 mg (41%) of **10f** as a brown solid. Mp: 86–87 °C (hexane/EtOAc). ^1^H NMR (400.16 MHz, CDCl_3_): δ 7.58–7.50 (m, 1H), 7.18–7.11 (m, 4H), 6.84–6.76 (m, 4H), 6.19 (br s, 1H, NH), 5.39 (s, 2H), 3.87 (s, 3H), 3.75 (s, 3H), 3.23 (br s, 1H, OH), 1.88 (s, 3H), 1.66 (s, 3H), 1.61 (s, 3H) ppm. ^13^C{^1^H} NMR (100.62 MHz, CDCl_3_): δ 170.7, 169.6, 159.2, 138.9, 137.9, 129.6, 127.5, 126.9 (2×), 126.6, 122.6, 122.5, 120.3, 118.3, 114.5 (2×), 110.6, 102.4, 74.6, 55.4, 53.1, 47.9, 30.6, 28.8, 23.2 (CH_3_) ppm. FTIR (NaCl): ν 3500–3100 (s, O–H), 2934 (m, C–H), 2928 (m, C–H), 2837 (m, C–H), 1748 (s, C=O), 1672 (s, C=O), 1513 (s), 1465 (s), 1248 (s), 1034 (w) cm^–1^. HRMS (ESI) *m*/*z*: [M + H]^+^ calcd for C_25_H_29_N_2_O_5_ 437.2071; found 437.2065.

#### Methyl (Z)-2-Acetamido-3-[2-(cyclohex-1-en-1-yl)-1-(4-methoxybenzyl)-1H-indol-3-yl]acrylate (**10g**)

Following the procedure for the preparation of *N*-PMB-dehydrotryptophans **10**, starting from **9g** (50 mg, 0.158 mmol) afforded 48 mg (67%) of **10g** as a solid. Mp: 59–60 °C (hexane/EtOAc). ^1^H NMR (400.16 MHz, CDCl_3_): δ 7.57–7.49 (m, 1H), 7.43 (s, 1H), 7.18–7.10 (m, 3H), 6.98–6.90 (m, 3H), 6.80 (d, *J* = 8.4 Hz, 2H), 5.91 (s, 1H), 5.24 (s, 2H), 3.85 (s, 3H), 3.76 (s, 3H), 2.27–2.19 (m, 2H), 2.14–2.07 (m, 2H), 2.02 (s, 3H), 1.77–1.64 (m, 4H) ppm. ^13^C{^1^H} NMR (100.62 MHz, CDCl_3_): δ 168.3, 165.9, 158.9, 146.0, 136.9, 134.2, 129.5, 129.3, 127.3 (2×), 126.1, 125.6, 122.3, 121.6, 120.7, 120.6, 114.2 (2×), 110.8, 107.7, 55.3, 52.4, 47.4, 29.7, 25.6, 23.3, 22.6, 21.7 ppm. FTIR (NaCl): ν 3500–3100 (br, m, N–H), 3006 (m, C–H), 2936 (s, C–H), 2840 (m, C–H), 1708 (s, C=O), 1678 (s, C=O), 1513 (s), 1250 (s), 750 (s) cm^–1^. HRMS (ESI) *m*/*z*: [M + H]^+^ calcd for C_28_H_31_N_2_O_4_ 459.2278; found 459.2296.

#### Methyl (Z)-2-Acetamido-3-[2-(*tert*-butyl)-1-(4-methoxybenzyl)-5-methyl-1H-indol-3-yl]acrylate (**10h**)

Starting from **9h** (60 mg, 0.20 mmol) afforded 46 mg (53%) of **10h** as a foam. ^1^H NMR (400.16 MHz, CDCl_3_, 323 K): δ 7.61 (s, 1H), 7.01 (s, 1H), 6.89 (d, *J* = 1.2 Hz, 2H), 6.80 (s, 4H), 6.60 (s, 1H), 5.56 (s, 2H), 3.89 (s, 3H), 3.75 (s, 3H), 2.38 (s, 3H), 1.83 (s, 3H), 1.49 (s, 9H) ppm. ^13^C{^1^H} NMR (100.62 MHz, CDCl_3_, 323 K): δ 165.7, 158.9, 146.3, 136.2, 129.9, 129.8, 128.2, 126.8 (2×), 125.8, 125.7, 123.8, 119.2, 114.1, 114.3 (2×), 110.4, 105.3, 55.4, 52.5, 48.9, 34.7, 32.0 (3×), 23.1, 21.4 ppm. FTIR (NaCl): ν 3327 (s, N–H), 2996 (w), 2955 (w), 2874 (w), 2837 (w), 1719 (s, C=O), 1676 (s, C=O), 1513 (s), 1249 (s), 1181 (w), 1036 (w) cm^–1^. HRMS (ESI) *m*/*z*: [M + H]^+^ calcd for C_27_H_33_N_2_O_4_ 449.2422; found 449.2434.

#### Methyl (Z)-2-Acetamido-)-3-[2-(*tert*-butyl)-5-methoxycarbonyl-1-(4-methoxybenzyl)-1H-indol-3-yl]acrylate (**10i**)

Starting from **9i** (90 mg, 0.26 mmol) afforded 60 mg (48%) of **10i** as a foam. ^1^H NMR (400.16 MHz, CDCl_3_, 323 K): δ 7.98 (d, *J* = 2.3 Hz, 1H), 7.76 (s, 1H), 7.74 (dd, *J* = 8.6, 1.6 Hz, 1H), 6.98 (d, *J* = 8.1 Hz, 1H), 6.79–6.76 (m, 4H), 5.60 (s, 2H), 3.90 (s, 3H), 3.89 (s, 3H), 3.75 (s, 3H), 1.75 (s, 3H), 1.54 (s, 9H) ppm. ^13^C{^1^H} NMR (100.62 MHz, CDCl_3_, 323 K): δ 168.2, 165.7, 159.0, 147.6, 140.5, 132.3, 129.9, 129.2, 128.7, 128.6, 126.8 (2×), 124.8, 123.5, 123.3, 114.4 (2×), 110.4, 55.4, 52.7, 51.9, 49.2, 34.9, 31.8 (3×), 23.1 ppm. FTIR (NaCl): ν 3332 (w, N–H), 2952 (w), 2919 (w), 2841 (w), 1715 (s, C=O), 1513 (w), 1436 (w), 1274 (s), 1248 (s), 1181 (w), 1132 (w) cm^–1^. HRMS (ESI) *m*/*z*: [M + H]^+^ calcd for C_28_H_33_N_2_O_6_ 493.2327; found 493.2333.

#### Methyl (Z)-2-Acetamido-3-[2-(cyclohex-1-en-1-yl)-1-(4-methoxybenzyl)-5-methyl-1H-indol-3-yl]acrylate (**10j**)

Starting from **9j** (75 mg, 0.23 mmol) afforded 53 mg (50%) of **10j** as a foam. ^1^H NMR (400.16 MHz, DMSO-*d*_6_, 333 K): δ 9.23 (s, 1H, NH), 7.34 (s, 1H), 7.23 (d, *J* = 8.2 Hz, 2H), 7.03–6.93 (m, 3H), 6.84 (d, *J* = 8.7 Hz, 2H), 5.86 (t, *J* = 3.8 Hz, 1H), 5.26 (s, 2H), 3.70 (s, 3H), 2.36 (s, 3H), 2.24–2.18 (m, 2H), 2.10–2.03 (m, 2H), 1.91 (s, 3H), 1.66–1.61 (m, 4H) ppm. ^13^C{^1^H} NMR (100.62 MHz, DMSO-*d*_6_, 333 K): δ 168.4, 168.3, 165.6, 158.3, 145.8, 134.9, 133.1, 129.6, 128.6, 128.4, 127.5 (2×), 126.3, 125.2, 123.0, 120.9, 113.8 (2×), 110.4, 106.7, 54.9, 51.5, 46.4, 29.3, 24.8, 22.3, 22.0, 21.0, 20.9 ppm. FTIR (NaCl): ν 2931 (s, N–H), 2856 (w), 2836 (w), 1710 (s, C=O), 1665 (s, C=O), 1512 (s), 1415 (s), 1249 (s), 1175 (w), 1035 (w) cm^–1^. HRMS (ESI) *m*/*z*: [M + H]^+^ calcd for C_29_H_33_N_2_O_4_, 473.2412; found 473.2434.

#### Methyl (Z)-2-Acetamido-3-[1-(4-methoxybenzyl)-1H-indol-3-yl]acrylate (**12**)

Following the procedure for the preparation of *N*-PMB-dehydrotryptophans **10**, starting from **9k** (25 mg, 0.071 mmol) afforded 20 mg (75%) of the desilylated **12**. Mp: 180–181 °C (hexane/EtOAc). ^1^H NMR (400.16 MHz, DMSO-*d*_6_): δ 9.63 (s, 1H, NH), 7.64 (d, *J* = 7.9 Hz, 1H), 7.55 (d, *J* = 8.4 Hz, 1H), 7.30 (s, 1H), 7.20 (t, *J* = 7.7 Hz, 1H), 7.11 (s, 1H), 7.08 (app t, *J* = 7.5 Hz, 1H), 6.98 (d, *J* = 8.4 Hz, 2H), 6.84 (d, *J* = 8.4 Hz, 2H), 5.51 (s, 2H), 3.68 (s, 6H), 2.08 (s, 3H) ppm. ^13^C{^1^H} NMR (100.62 MHz, DMSO-*d*_6_): δ 169.7, 165.7, 159.0, 137.8, 132.4, 130.5, 128.0 (2×), 127.8, 127.1, 123.9, 121.6, 120.7, 119.6, 114.5 (2×), 110.9, 106.4, 55.5, 52.6, 45.7, 23.0 ppm. FTIR (NaCl): ν 3001 (w, C–H), 2925 (s, C–H), 2853 (s, C–H), 1690 (s, C=O), 1674 (s, C=O), 1514 (s), 1333 (s), 1251 (s), 1032 (s) cm^–1^. HRMS (ESI) *m*/*z*: [M + H]^+^ calcd for C_22_H_23_N_2_O_4_ 379.1652; found 379.1644.

## Supporting Information Available

X-ray data; monitoring of reaction progress by HPLC-MS; copies of ^1^H NMR and ^13^C NMR spectra; computational data (PDF)

## Accession Codes

CCDC 1939395 contains the supplementary crystallographic data for this paper. These data can be obtained free of charge via www.ccdc.cam.ac.uk/data_request/cif, or by emailing data_request@ccdc.cam.ac.uk, or by contacting The Cambridge Crystallographic Data Centre, 12 Union Road, Cambridge CB2 1EZ, UK; fax: +44 1223 336033.
