# Supplementary material for: Palladium-Catalyzed Aminocyclization–Coupling Cascades: Preparation of Dehydrotryptophan Derivatives and Computational Study
Source: J Org Chem. 2021 Jun 14;86(13):8766–85. doi: 10.1021/acs.joc.1c00636 (PMC8929666; doi:10.1021/acs.joc.1c00636)
Supplement: Supplementary file 1 — jo1c00636_si_001.pdf [file jo1c00636_si_001.pdf]

PALLADIUM-CATALYZED AMINOCYCLIZATION-COUPLING CASCADES: PREPARATION OF DEHYDROTRYPTOPHAN DERIVATIVES AND COMPUTATIONAL STUDY

Belén Vaz,[a] Claudio Martínez,[a] Francisco Cruz,[a] J. Gabriel Denis,[a] Ángel R. de Lera,[a] José M. Aurrecoechea*[b] and Rosana Álvarez*[a]

[a] *Departamento de Química Orgánica, Facultad de Química (CINBIO) and Instituto de Investigación Biomédica de Vigo (IBIV), Universidade de Vigo, Lagoas-Marcosende, 36310 Vigo, Spain*

[b] *Departamento de Química Orgánica Orgánica e Inorgánica, Facultad de Ciencia y Tecnología, Universidad del País Vasco UPV/EHU, Apartado 644, 48080 Bilbao, Spain*

Corresponding Authors: R. Alvarez, rar@uvigo.es*; J.M. Aurrecoechea, jm.aurrecoechea@ehu.eus*

Keywords. Dehydrotryptophans, palladium, DFT calculations, Heck-type reaction, Sonogashira reaction.

SUPPORTING INFORMATION

Table of contents

	Page
1. X-ray data for compound **10c.** Figure S1 and Table S1	S2-S3
2. Monitoring of reaction progress by HPLC-MS	S4
3. Copies of ^{1}H NMR and ^{13}C NMR spectra	S5-S112
4. Computational data	S113-S159

Figure S1. ORTEP diagram of **10c** showing thermal ellipsoids at the 30% probability level. CCDC 1939395 contains the supplementary crystallographic data for this paper. These data can be obtained free of charge from The Cambridge Crystallographic Data Centre via www.ccdc.cam.ac.uk/data_request/cif.

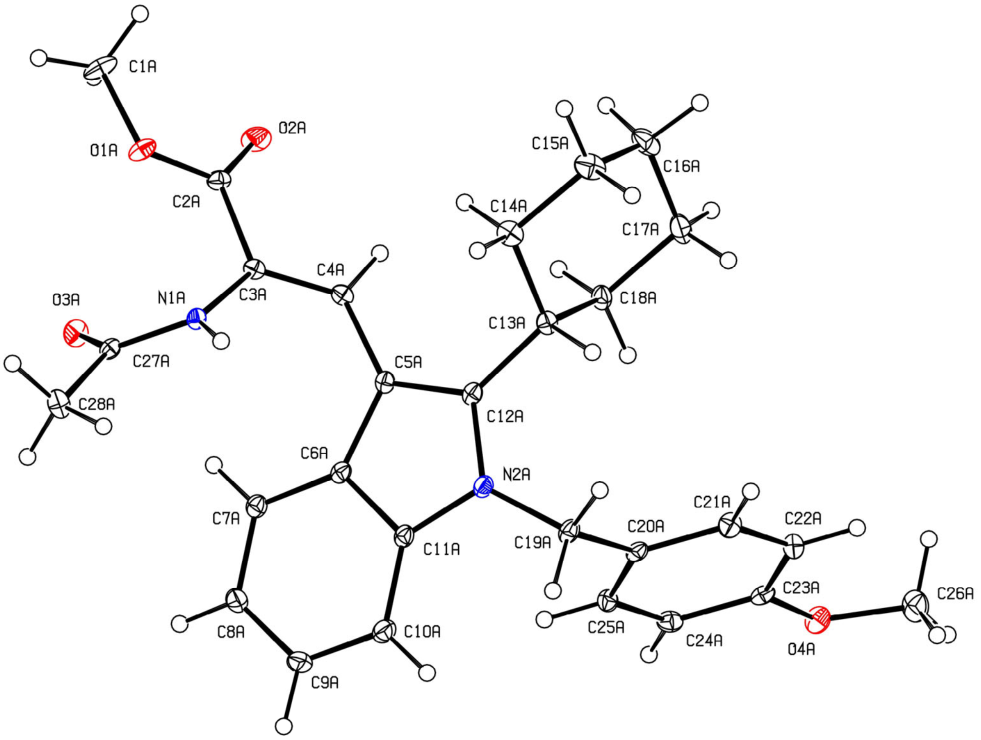

Table S1. Crystal data and structure refinement for **10c**.

Identification code	CCDC 1939395	
Empirical formula	C28 H32 N2 O4	
Formula weight	460.55	
Temperature	99.45 K	
Wavelength	0.71073 Å	
Crystal system	Triclinic	
Space group	P-1	
Unit cell dimensions	a = 9.5209(6) Å	α= 81.067(3)°.
	b = 12.9908(9) Å	β= 84.140(3)°.
	c = 19.8365(14) Å	γ = 88.695(3)°.
Volume	2411.0(3) Å^3	
Z	4	
Density (calculated)	1.269 Mg/m^3	

Absorption coefficient	0.085 mm^{-1}
F(000)	984
Crystal size	0.174 x 0.163 x 0.065 mm^3
Theta range for data collection	2.295 to 28.377°.
Index ranges	-12<=h<=12, -17<=k<=17, -26<=l<=26
Reflections collected	98485
Independent reflections	12052 [R(int) = 0.0571]
Completeness to theta = 25.242°	99.9 %
Absorption correction	Semi-empirical from equivalents
Max. and min. transmission	0.7457 and 0.7067
Refinement method	Full-matrix least-squares on F^2
Data / restraints / parameters	12052 / 0 / 627
Goodness-of-fit on F^2	1.046
Final R indices [I>2sigma(I)]	R1 = 0.0481, wR2 = 0.1020
R indices (all data)	R1 = 0.0689, wR2 = 0.1103
Extinction coefficient	n/a
Largest diff. peak and hole	0.328 and -0.271 e.$Å^{-3}$

Figure S2. Monitoring of the evolution of the reaction by HPLC-MS. Small aliquots of the reaction mixture were taken at different reaction times, and analysed. Column: LUNA, PFP(2), 100 Å, 100 x 10 mm. Eluent: A, H_2O (+0.1% HCO_2H); B, CH_3 (+0.1% HCO_2H), gradient from 95:5 to 50:50 A/B, 3 mL/min. Detection: PDA, ESI. t_R (starting alkene **3**) = 2.7 min; t_R (O=PPh_3) = 4.6 min; t_R (reaction product **4a**) = 5.1 min; t_R (starting aniline **1a**) = 8.8 min; t_R (**6a**) = 9.2 min; t_R (PPh_3) = 10.3 min. Both the coupling product **4a** and uncoupled indole **6a** are observed in the early stages of the reaction. PPh_3 was only detected by ESI at t = 0 whereas O=PPh_3 was first detected (PDA, and ESI) after 30 min and remained to the end of reaction.

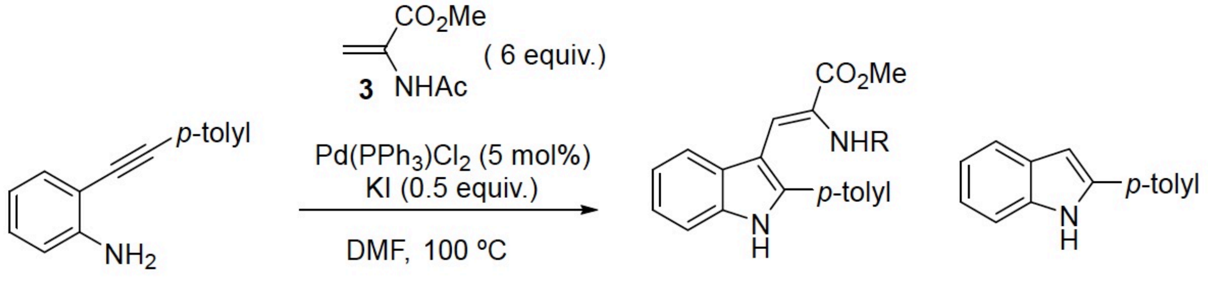

1a **4a** **6a**

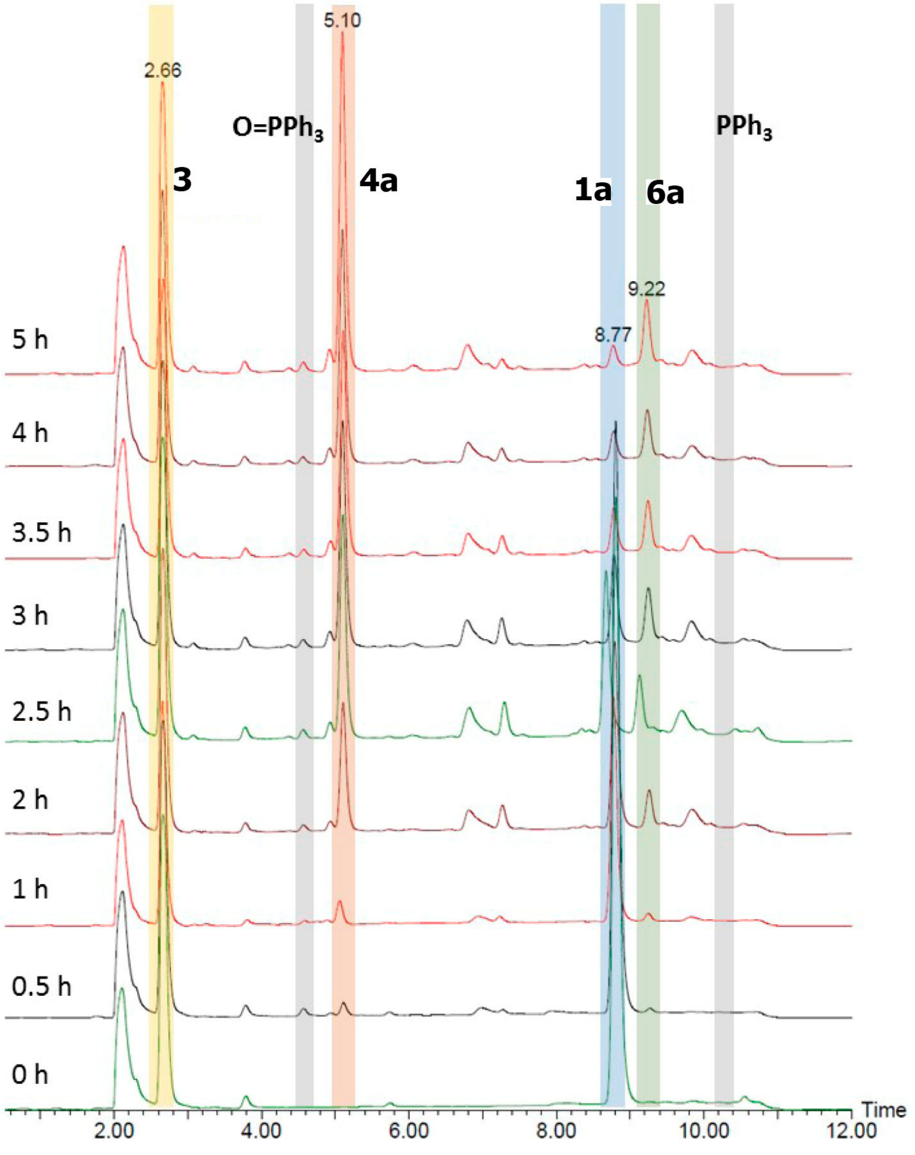

^{1}H NMR (400.16 MHz, $CDCl_3$) spectrum of 7b

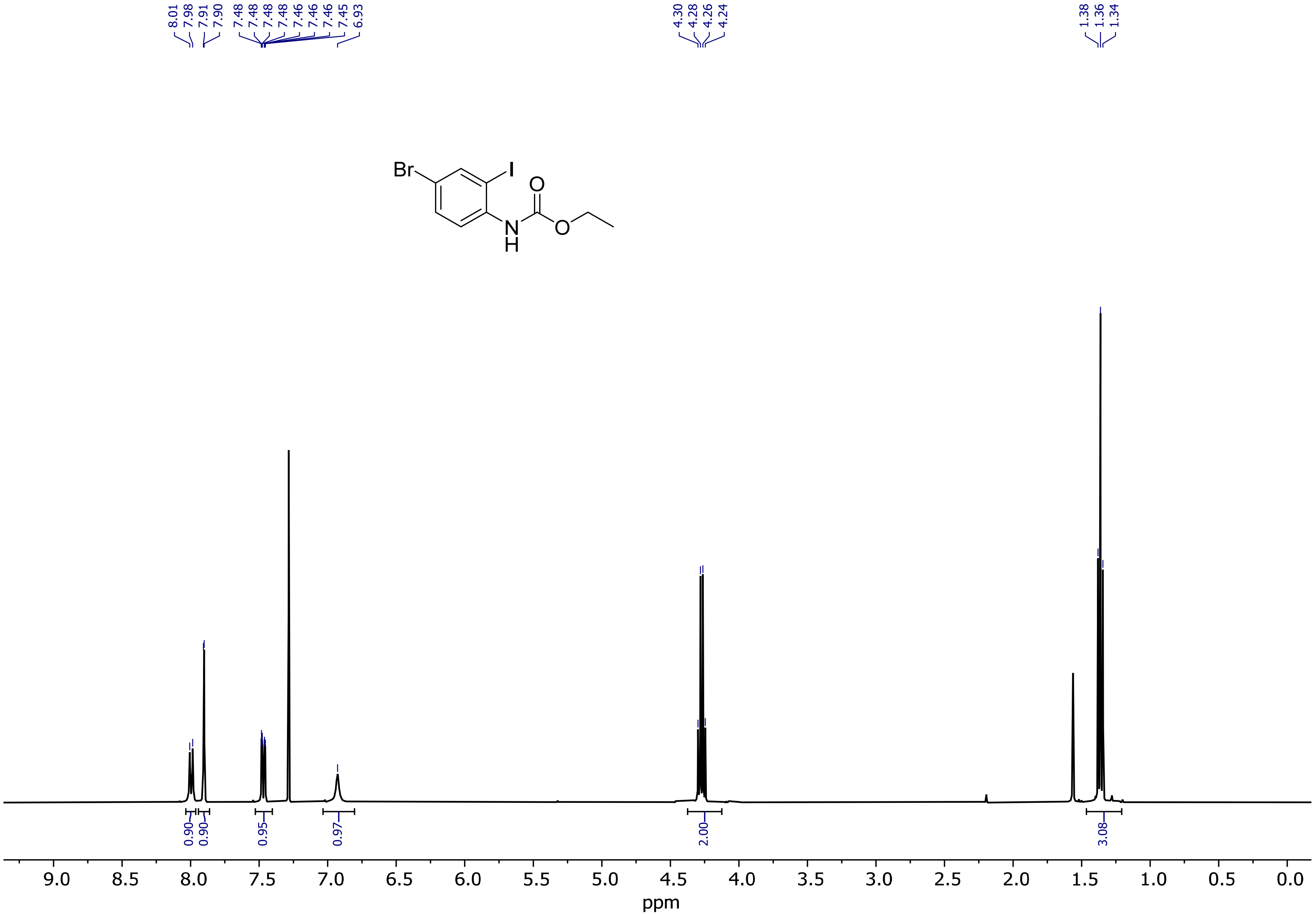

$^{13}C\ \{^{1}H\}$ NMR (100.62 MHz, $CDCl_3$) spectrum of 7b

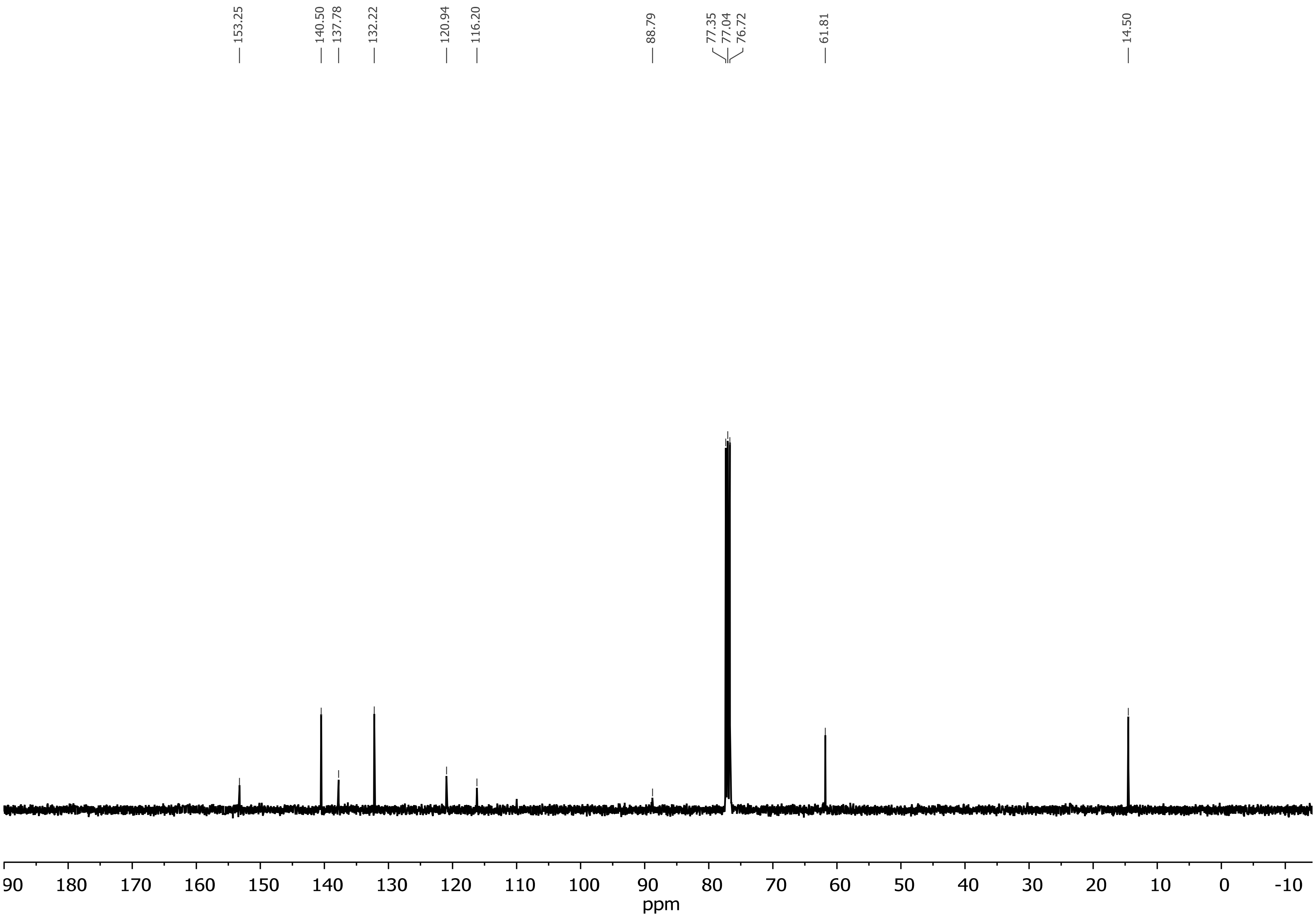

^{1}H NMR (400.16 MHz, $CDCl_3$) spectrum of 7c

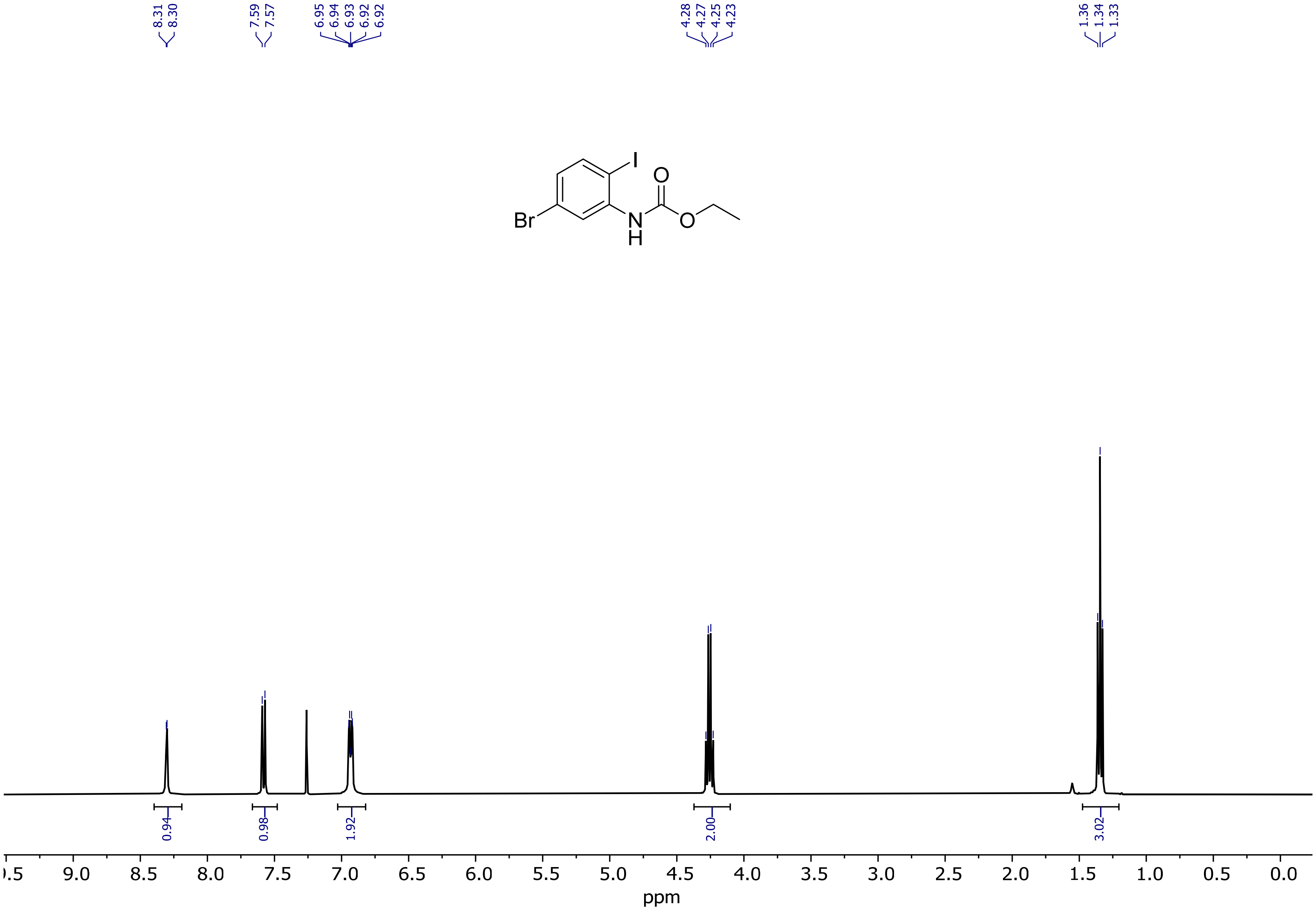

$^{13}C\{^{1}H\}$ NMR (100.62 MHz, CDCl$_3$) spectrum of 7c

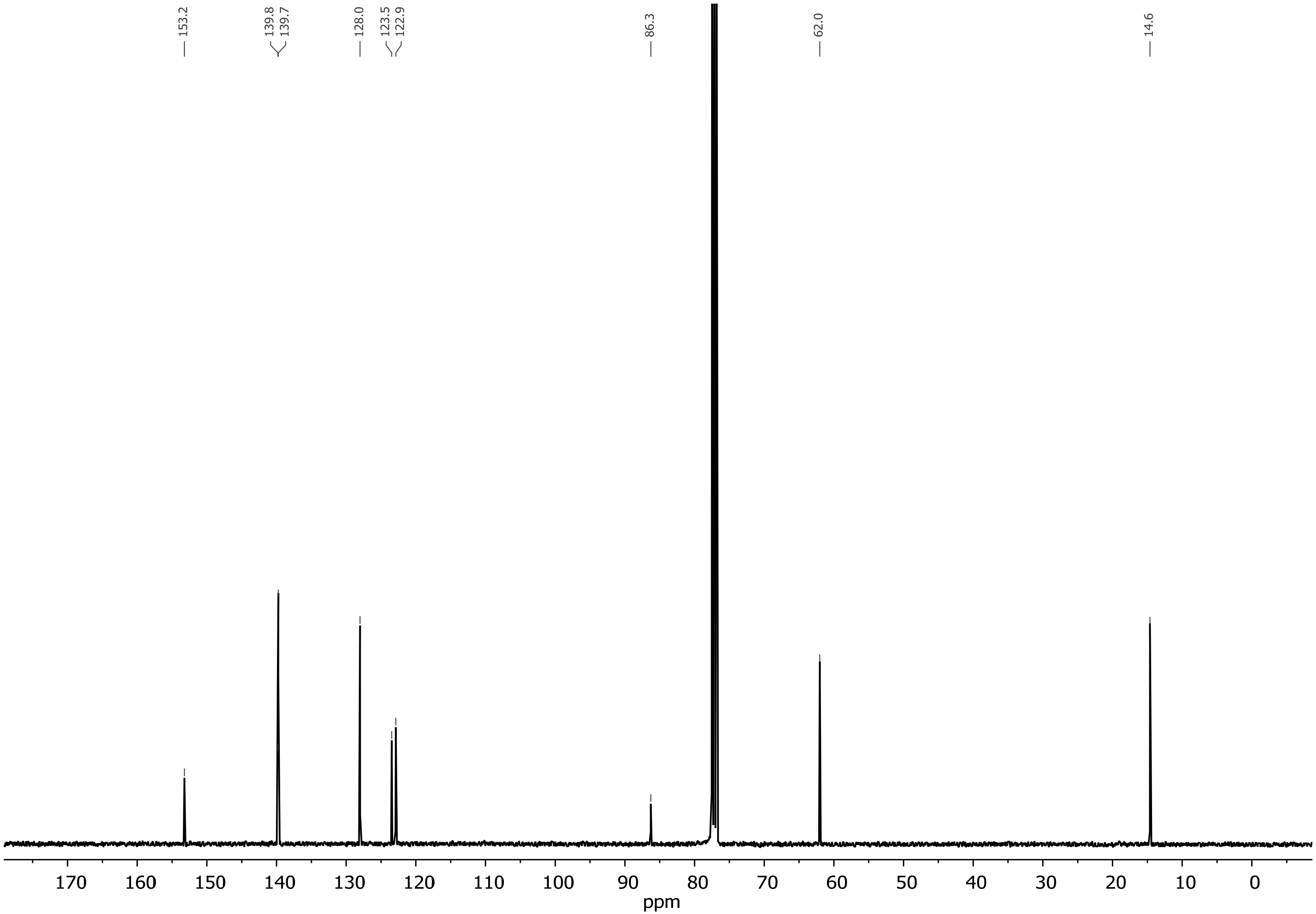

^{1}H NMR (400.16 MHz, $CDCl_3$) spectrum of 7d

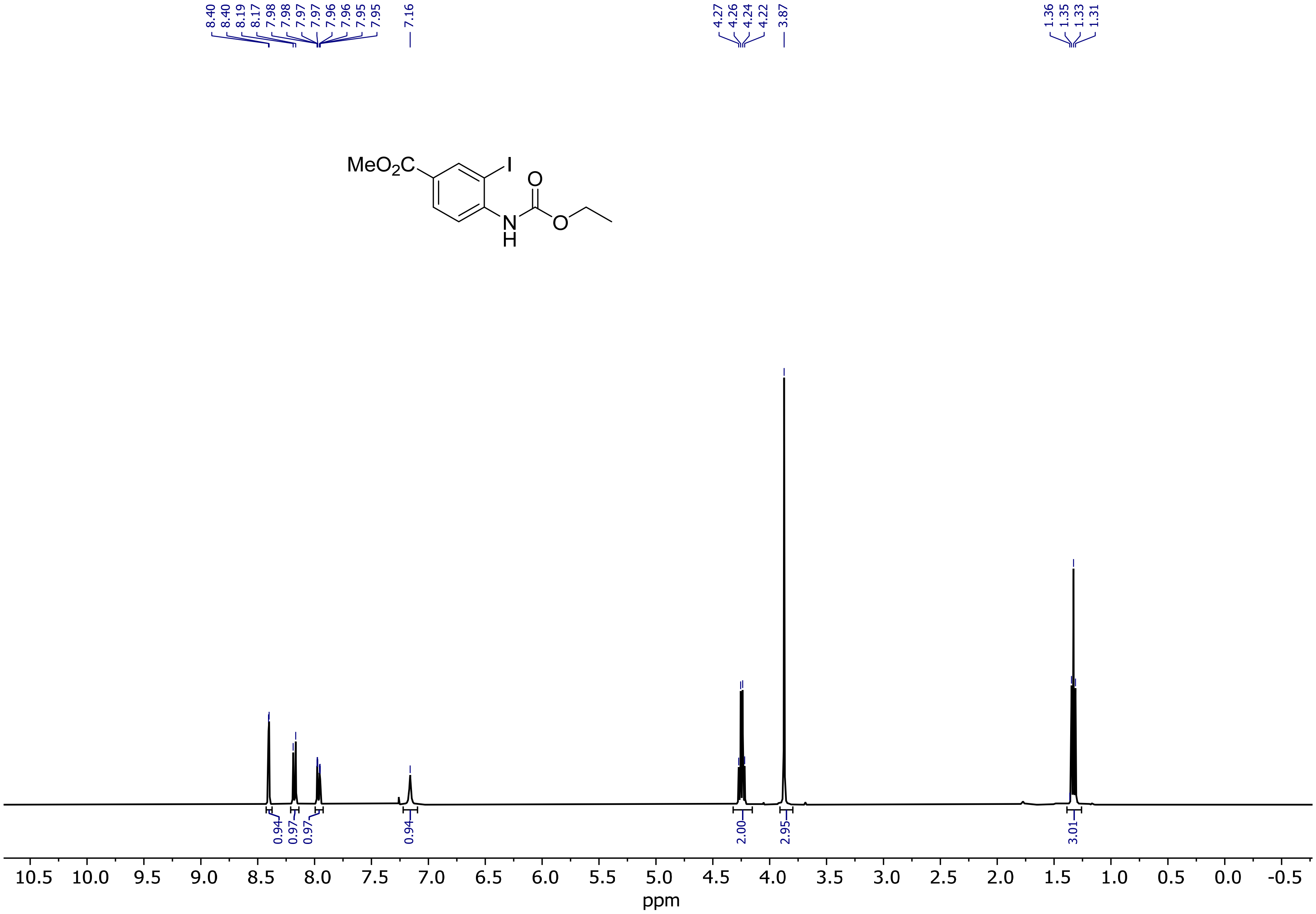

$^{13}C\{^1H\}$ NMR (100.62 MHz, $CDCl_3$) spectrum of 7d

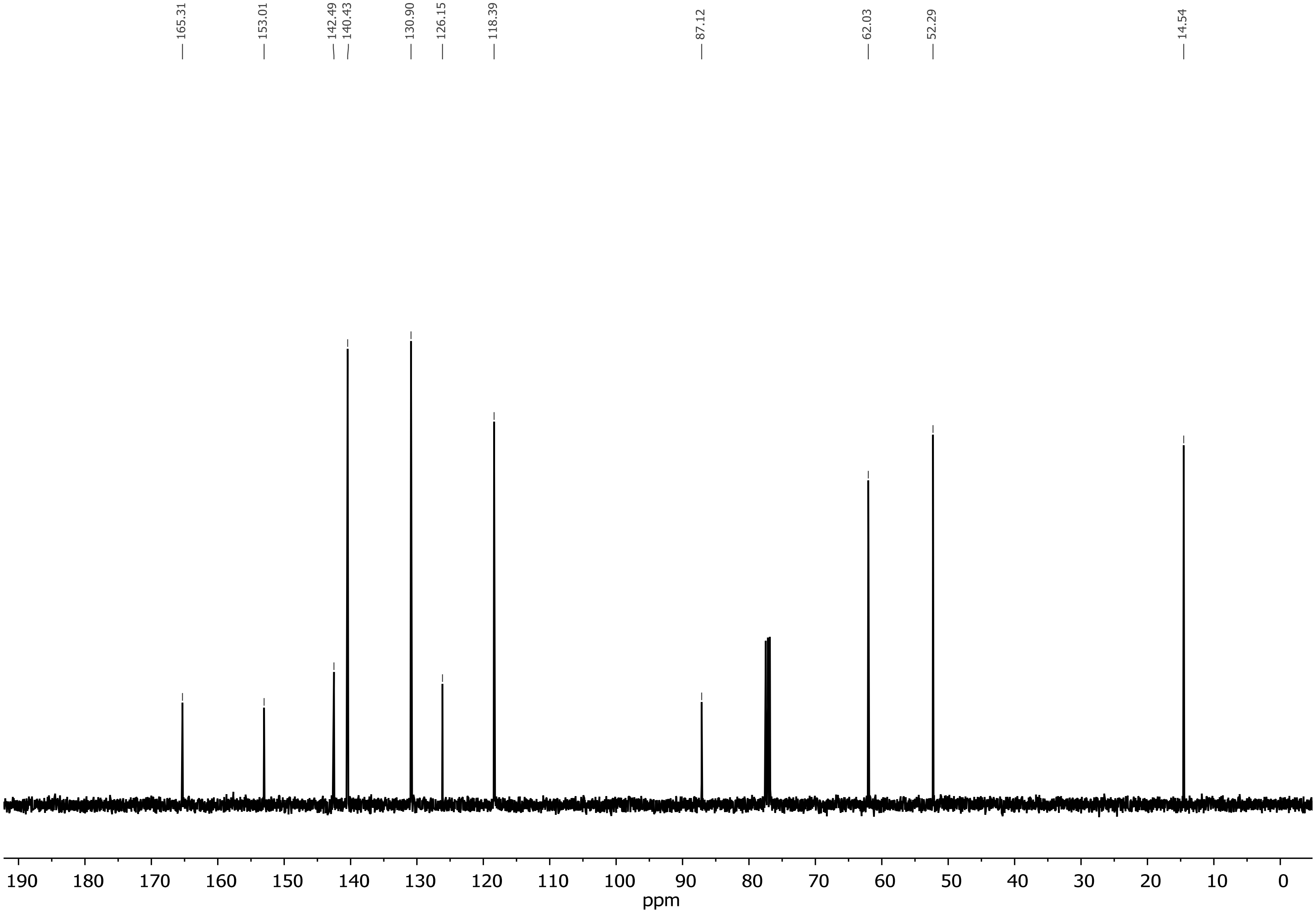

^{1}H NMR (400.16 MHz, CDCl$_3$) spectrum of 7e

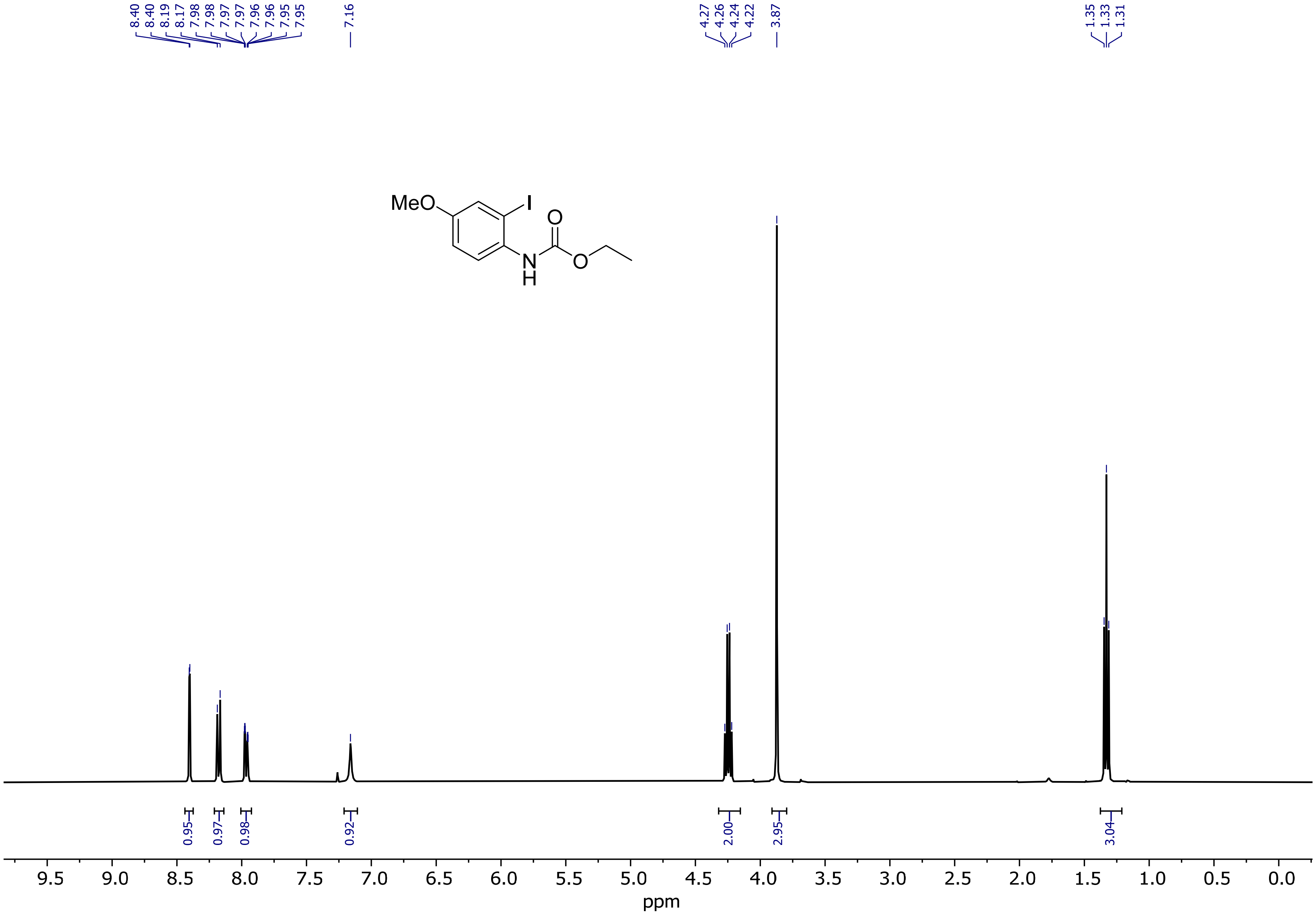

^{13}C {^{1}H} NMR (100.62 MHz, $CDCl_3$) spectrum of 7e

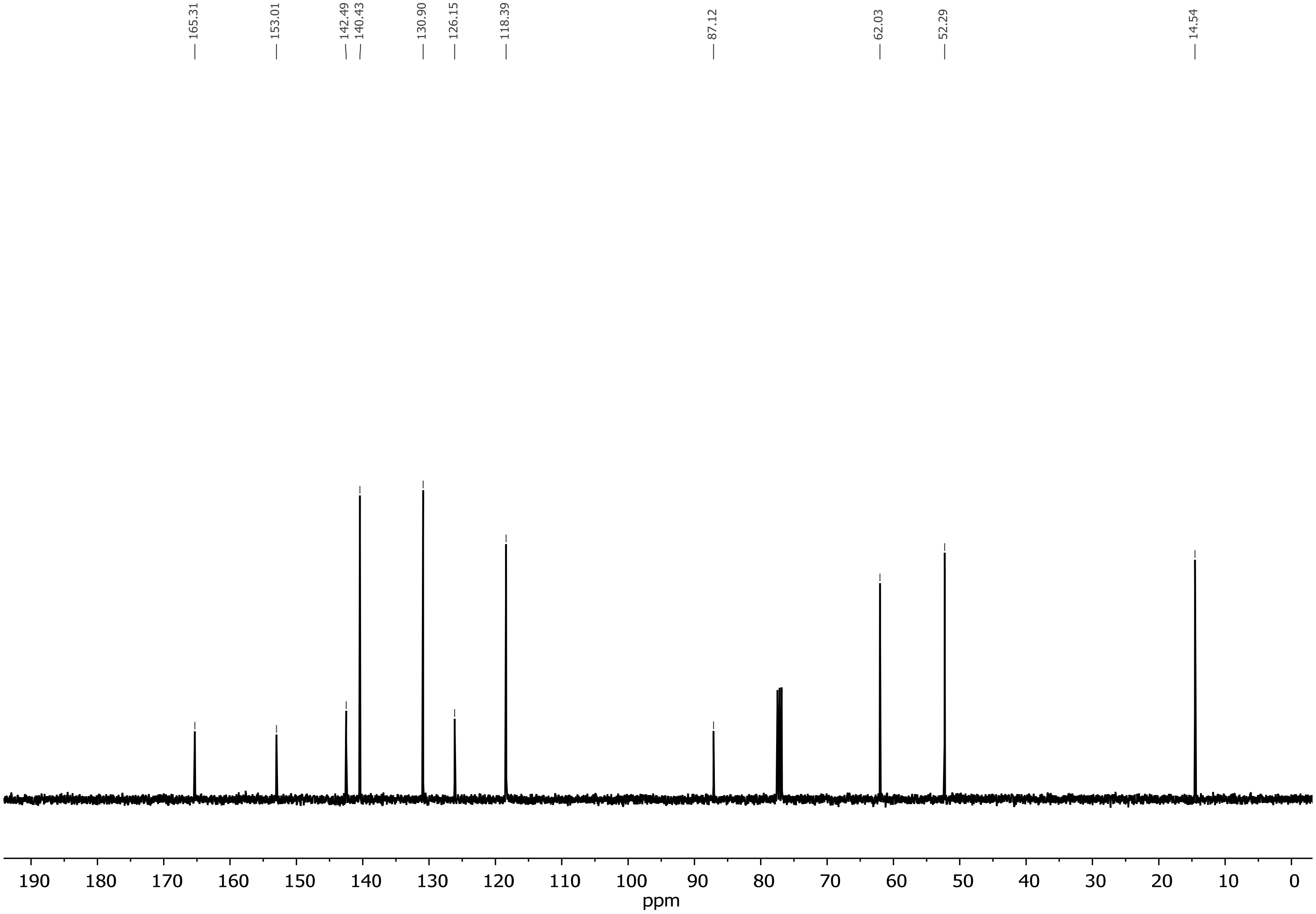

^{1}H NMR (400.16 MHz, CDCl3) spectrum of 1c

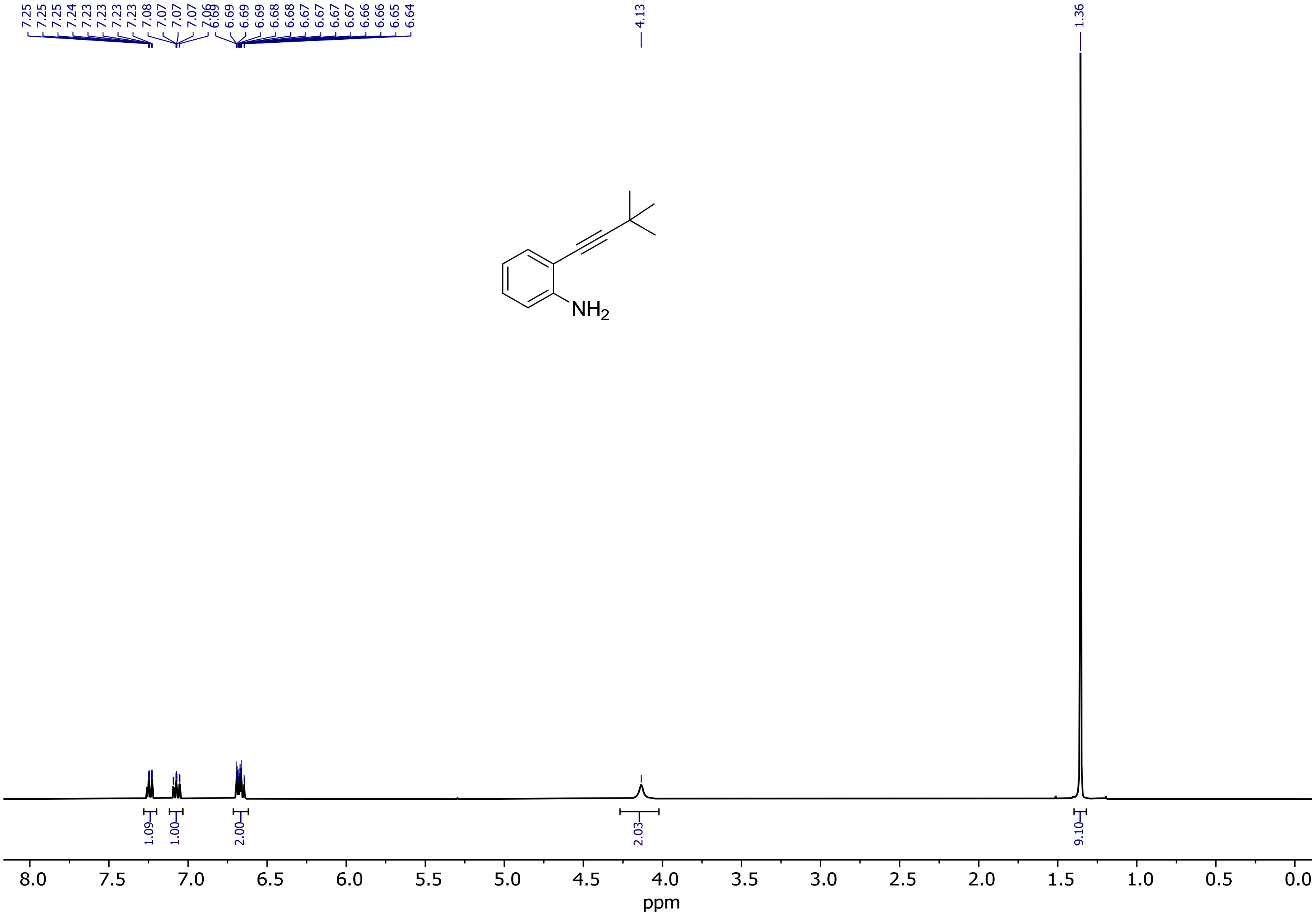

^{13}C {^{1}H} NMR (100.62 MHz, $CDCl_3$) spectrum of 1c

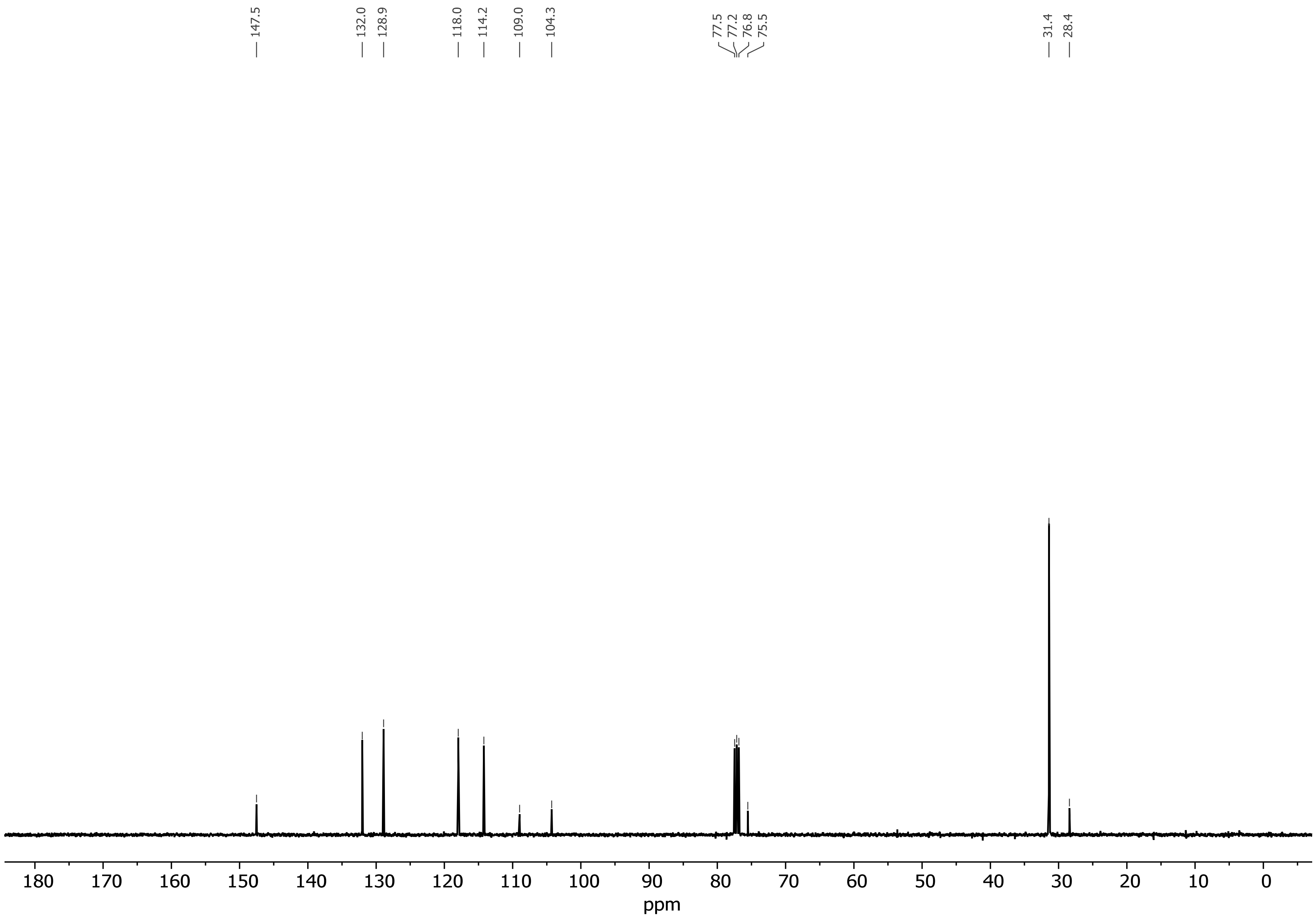

^{1}H NMR (400.16 MHz, $CDCl_3$) spectrum of 2a

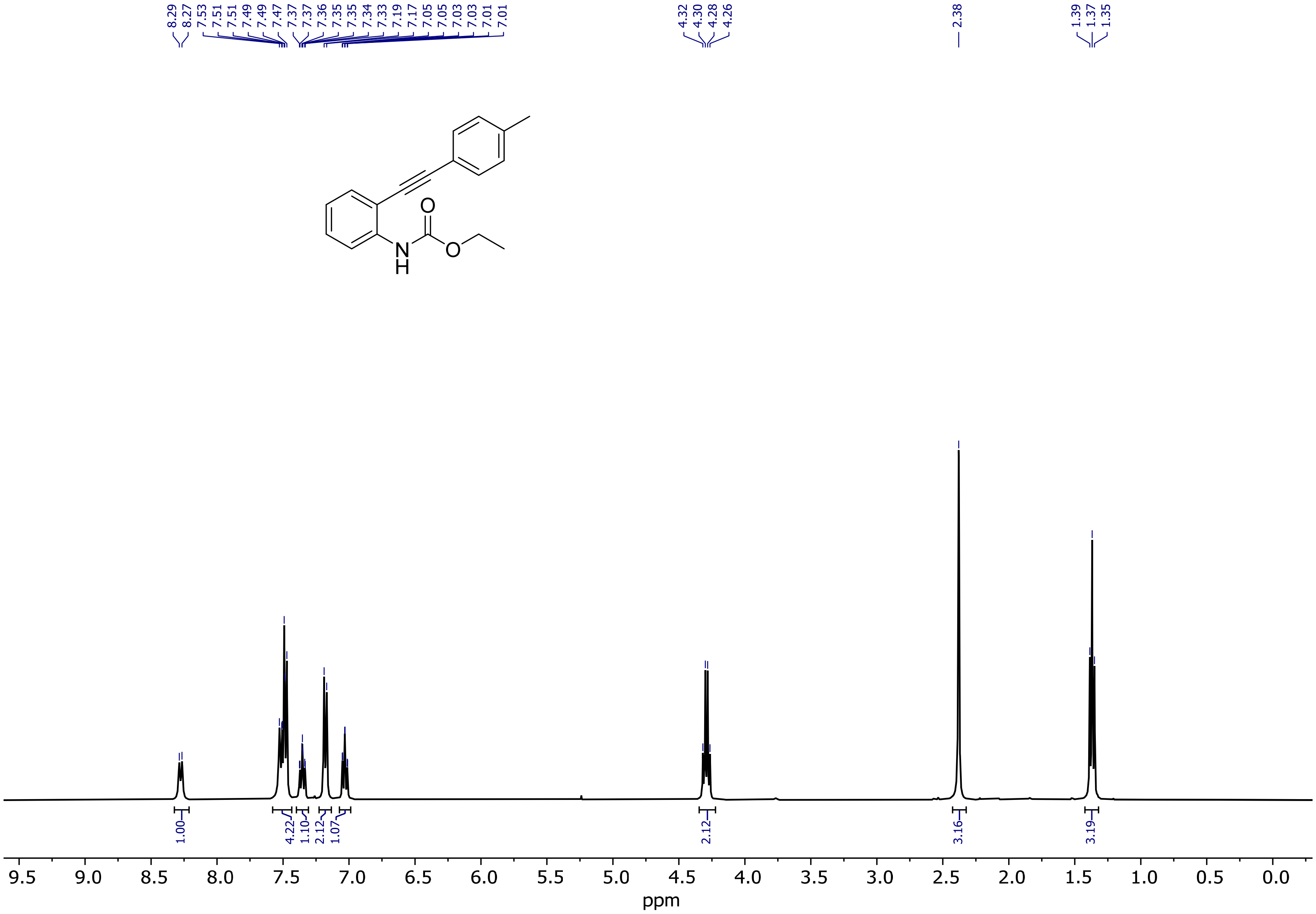

^{13}C {^{1}H} NMR (100.62 MHz, $CDCl_3$) spectrum of 2a

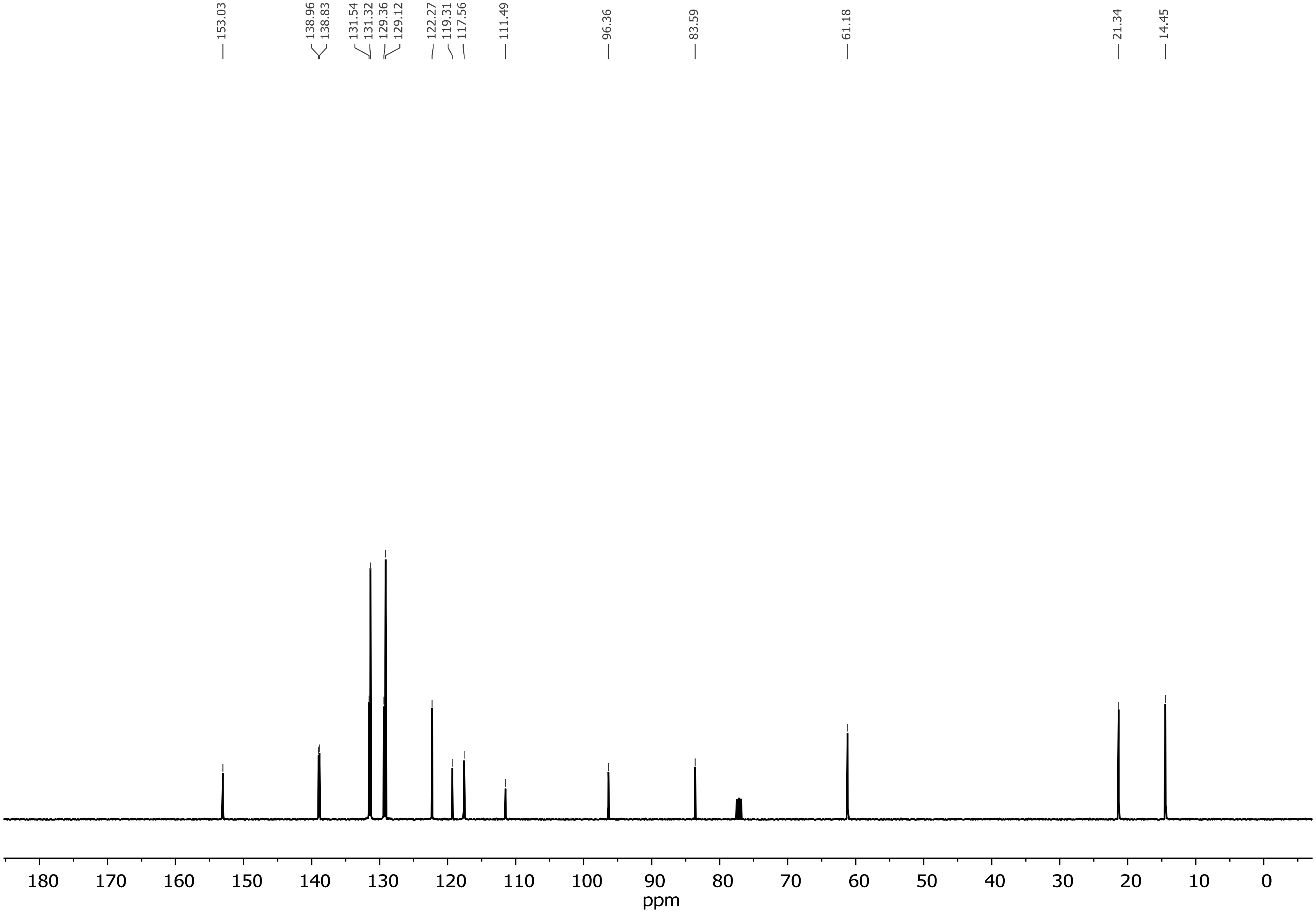

^{1}H NMR (400.16 MHz, CDCl$_3$) spectrum of 2b

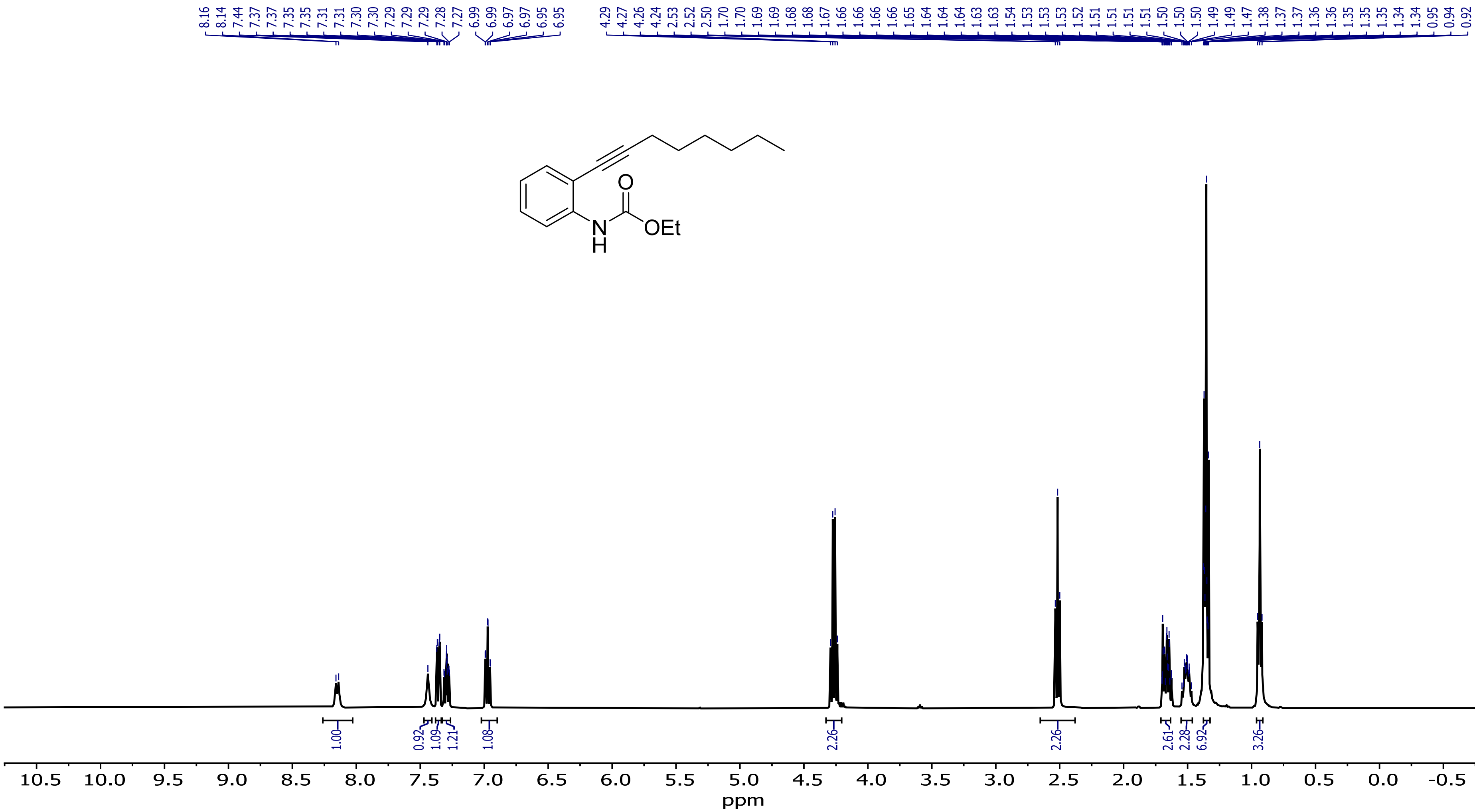

$^{13}C\{^{1}H\}$ NMR (100.62 MHz, $CDCl_3$) spectrum of 2b

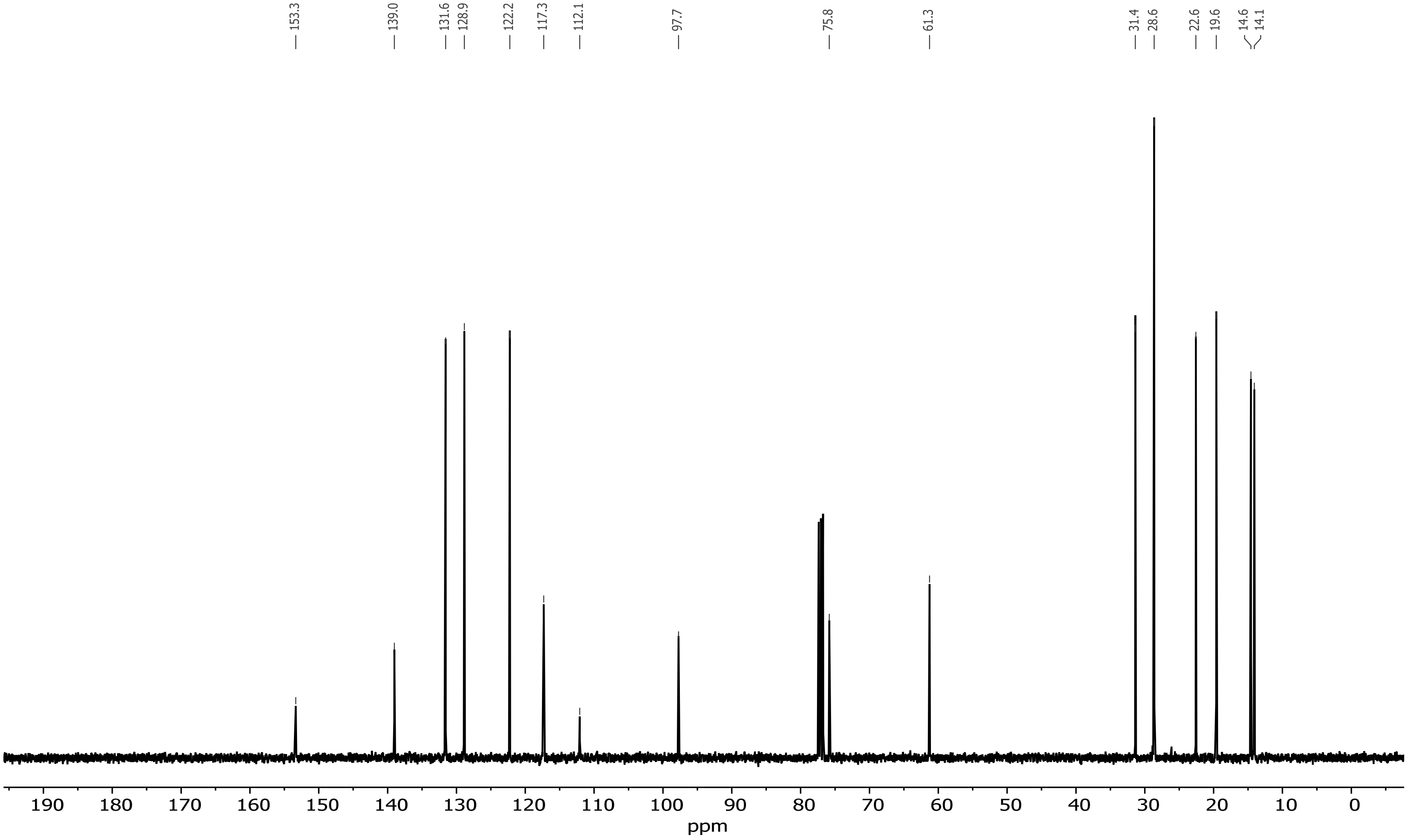

^{1}H NMR (400.16 MHz, $CDCl_3$) spectrum of 2c

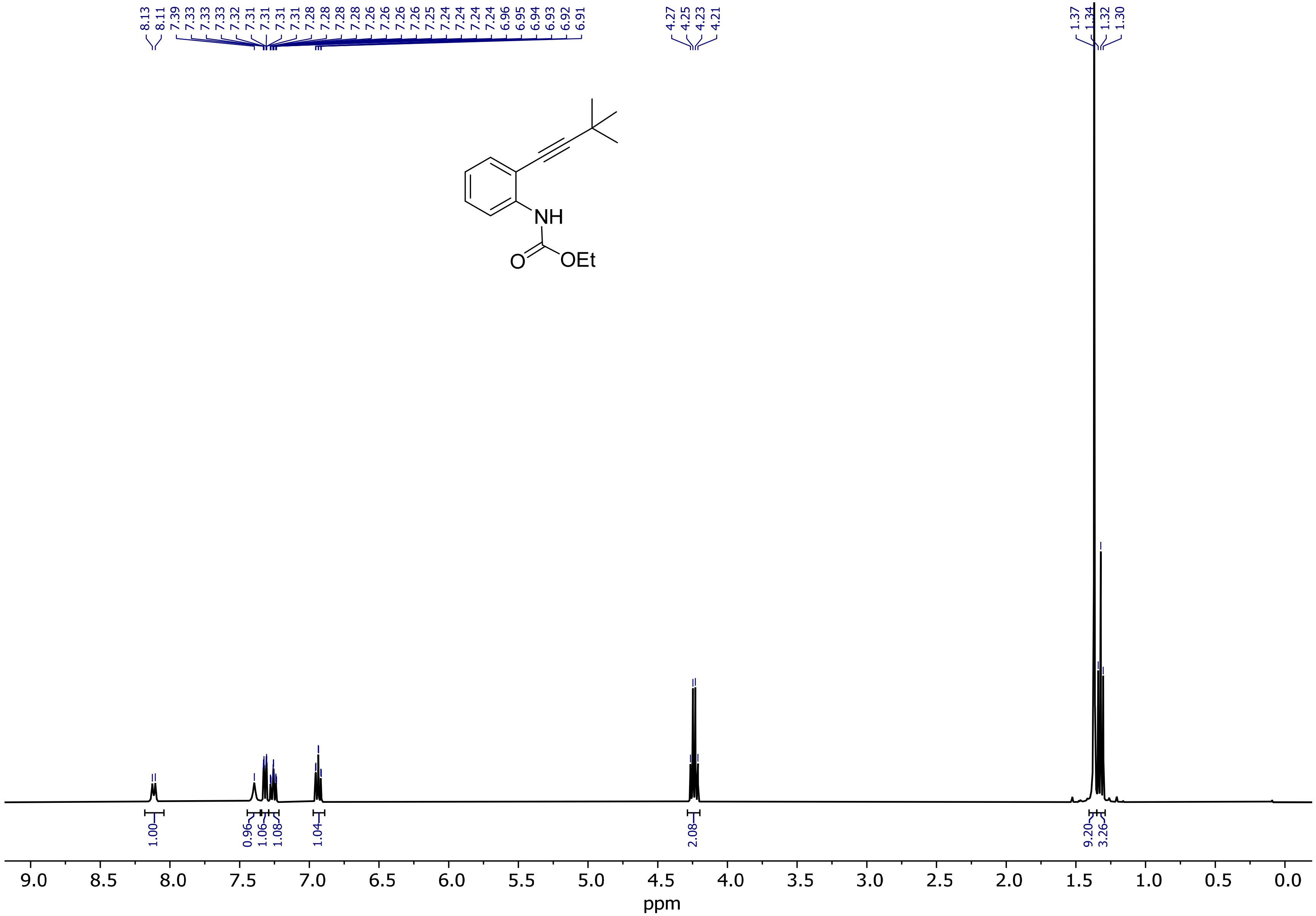

^{13}C {^{1}H} NMR (100.62 MHz, $CDCl_3$) spectrum of 2c

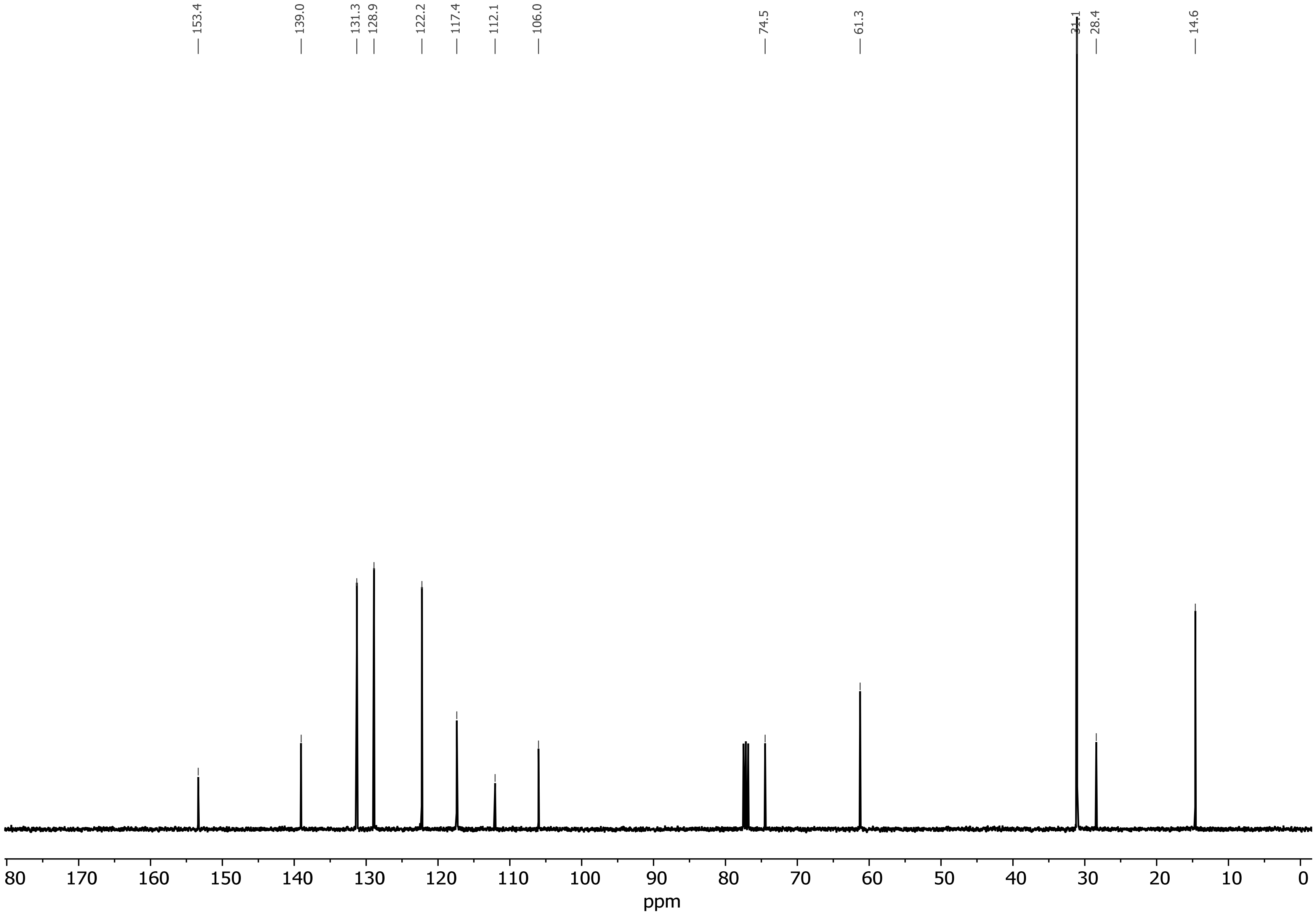

^{1}H NMR (400.16 MHz, $CDCl_3$) spectrum of 2o

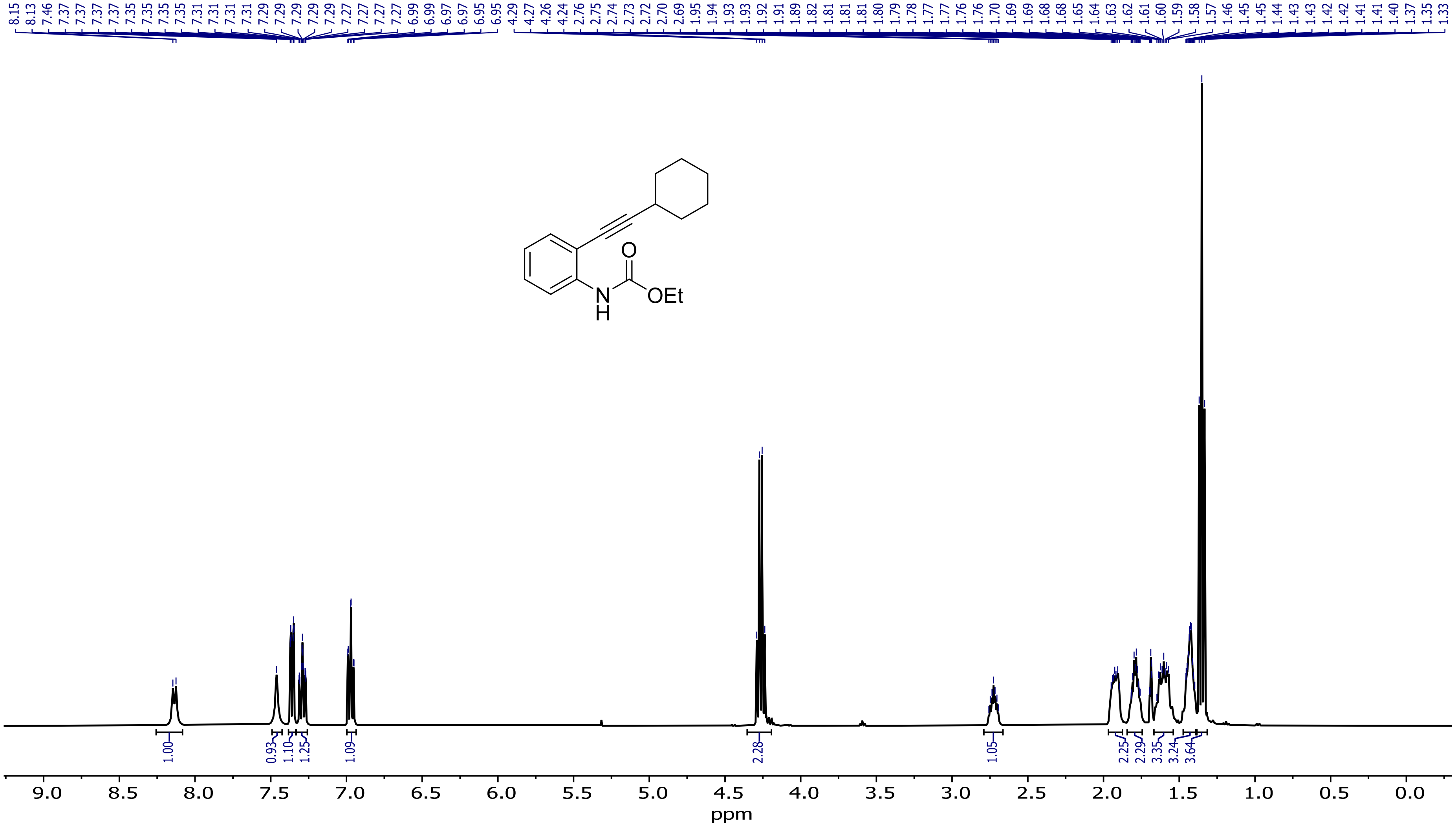

^{13}C {^{1}H} NMR (100.62 MHz, $CDCl_3$) spectrum of 2o

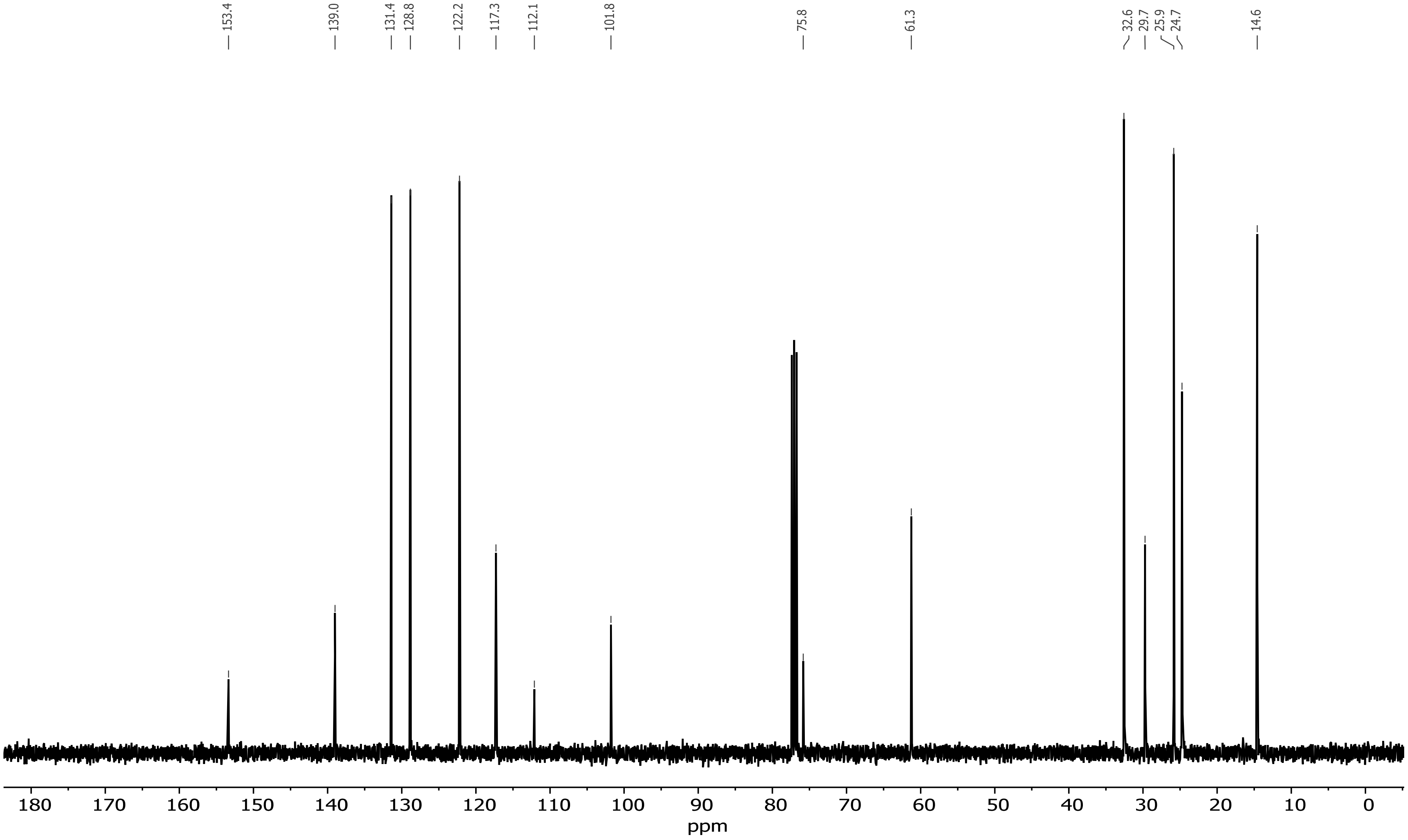

^{1}H NMR (400.16 MHz, $CDCl_3$) spectrum of 2p

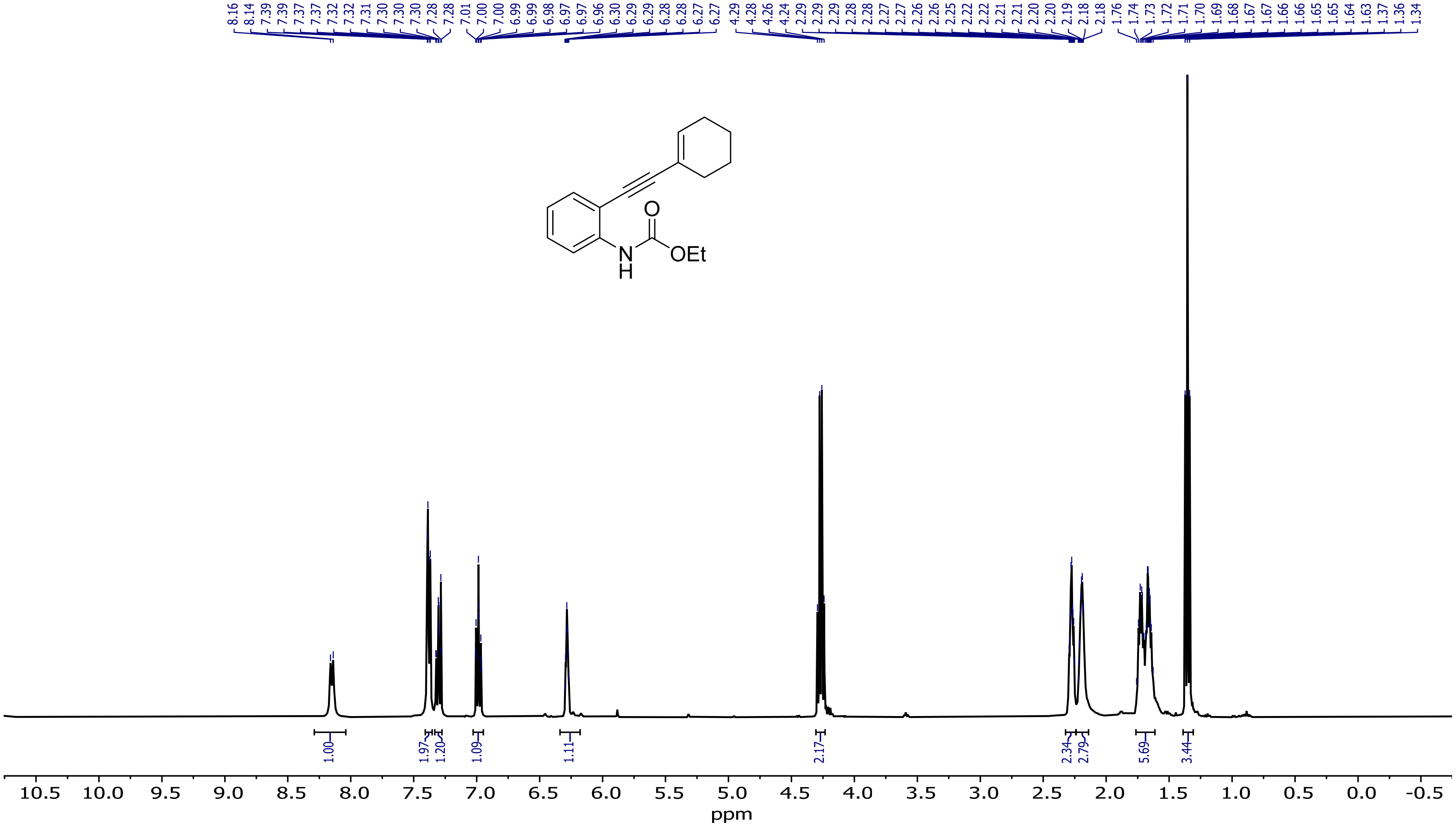

^{13}C {^1H} NMR (100.62 MHz, $CDCl_3$) spectrum of 2p

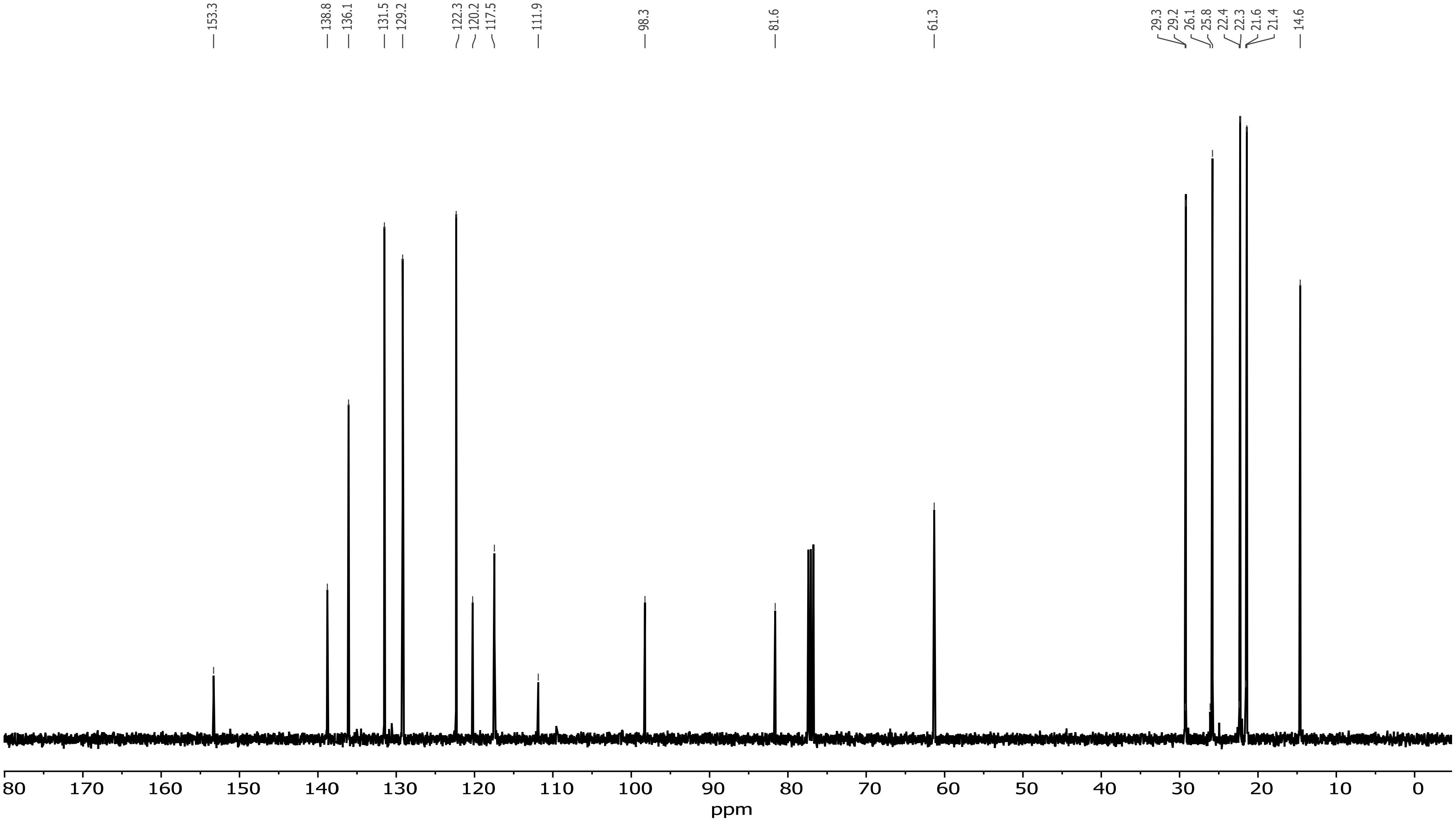

^{1}H NMR (400.16 MHz, CDCl₃) spectrum of 4-[(*tert*-butyldiphenylsilyl)oxy]-3,3-dimethylbutan-2-one

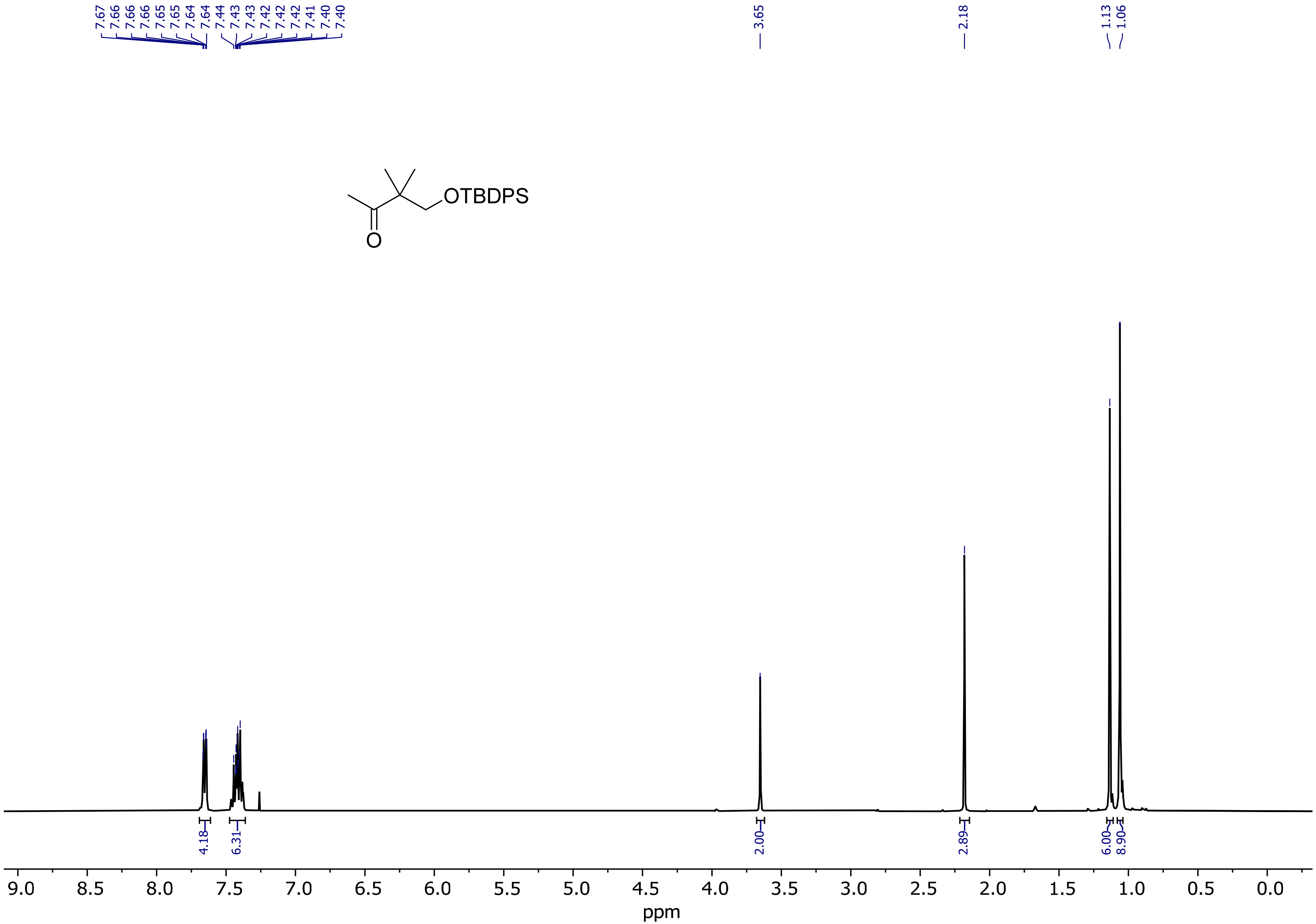

^{13}C {^{1}H} NMR (100.62 MHz, $CDCl_3$) spectrum of 4-[(*tert*-butyldiphenylsilyl)oxy]-3,3-dimethylbutan-2-one

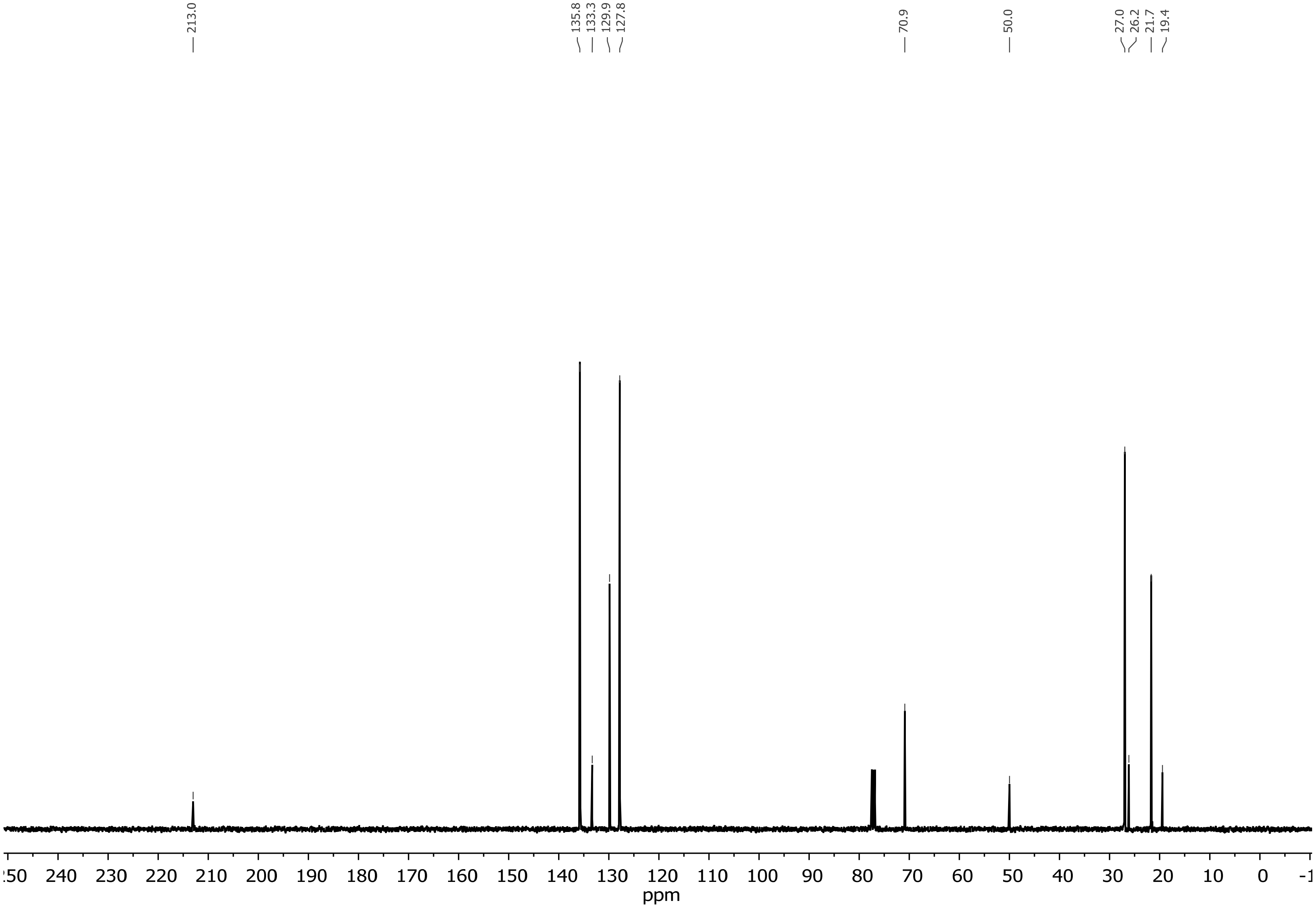

^{1}H NMR (400.16 MHz, CDCl$_3$) spectrum of 8n

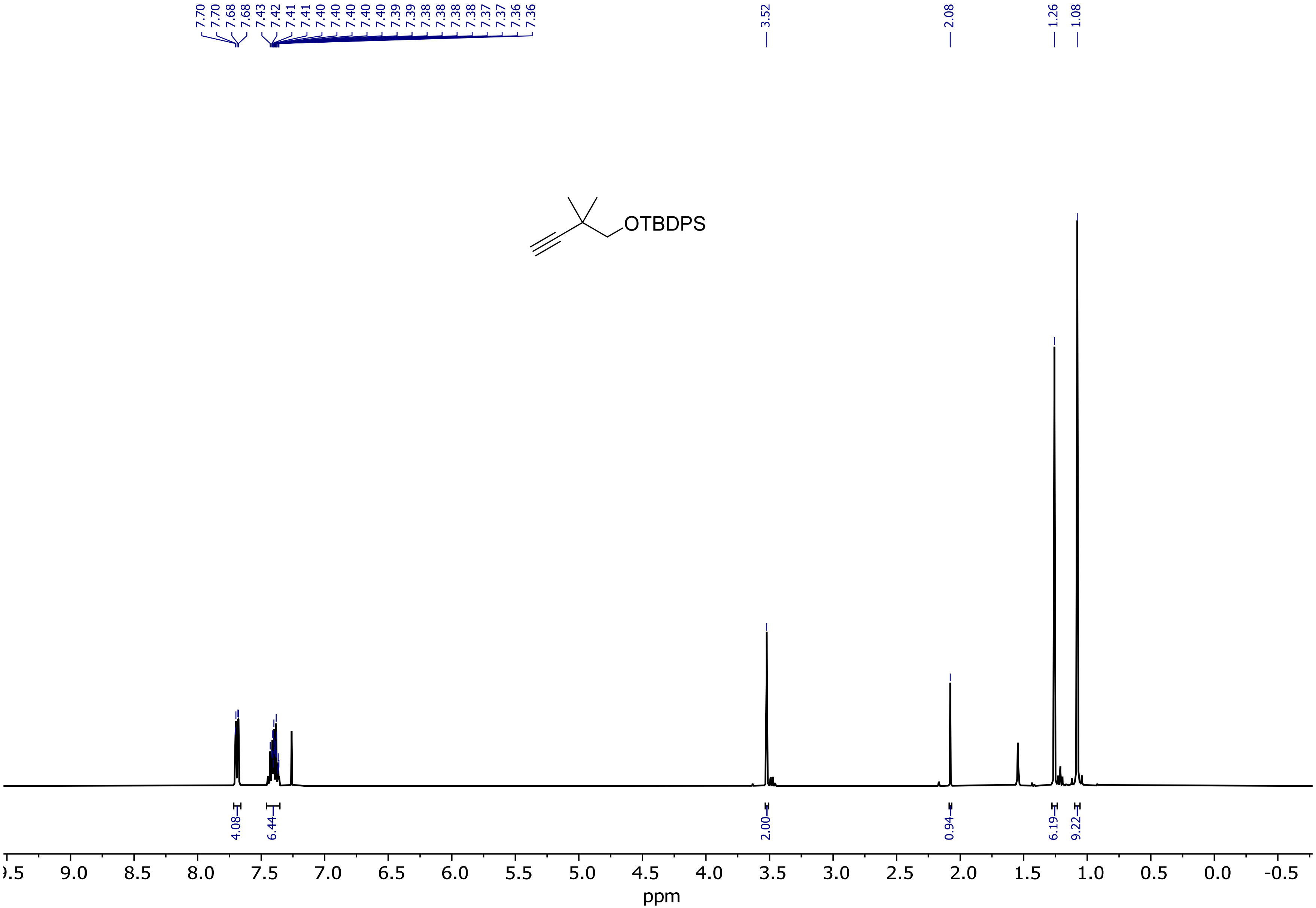

$^{13}C\ \{^{1}H\}$ NMR (100.62 MHz, CDCl$_3$) spectrum of 8n

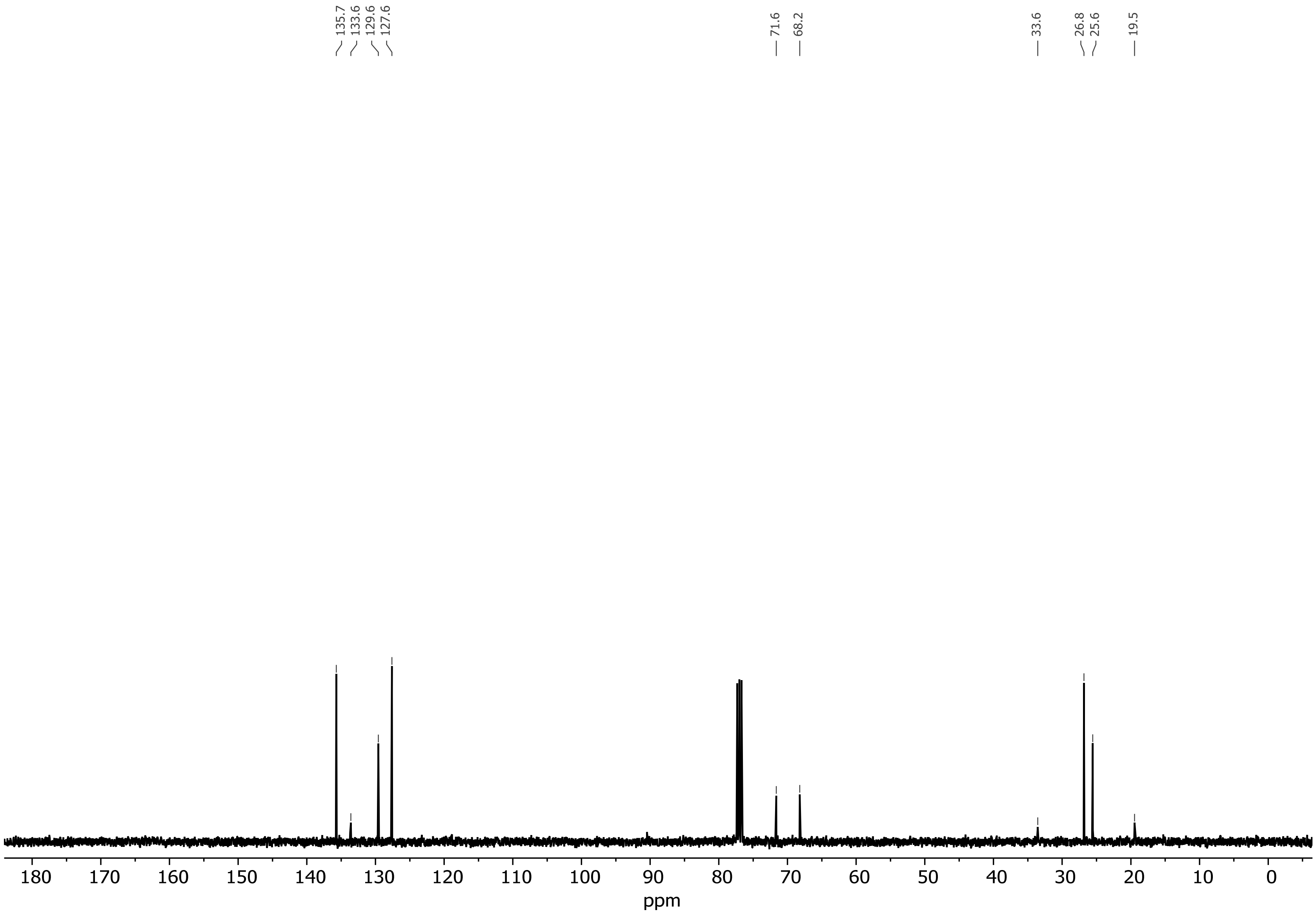

^{1}H NMR (400.16 MHz, $CDCl_3$) spectrum of 9a

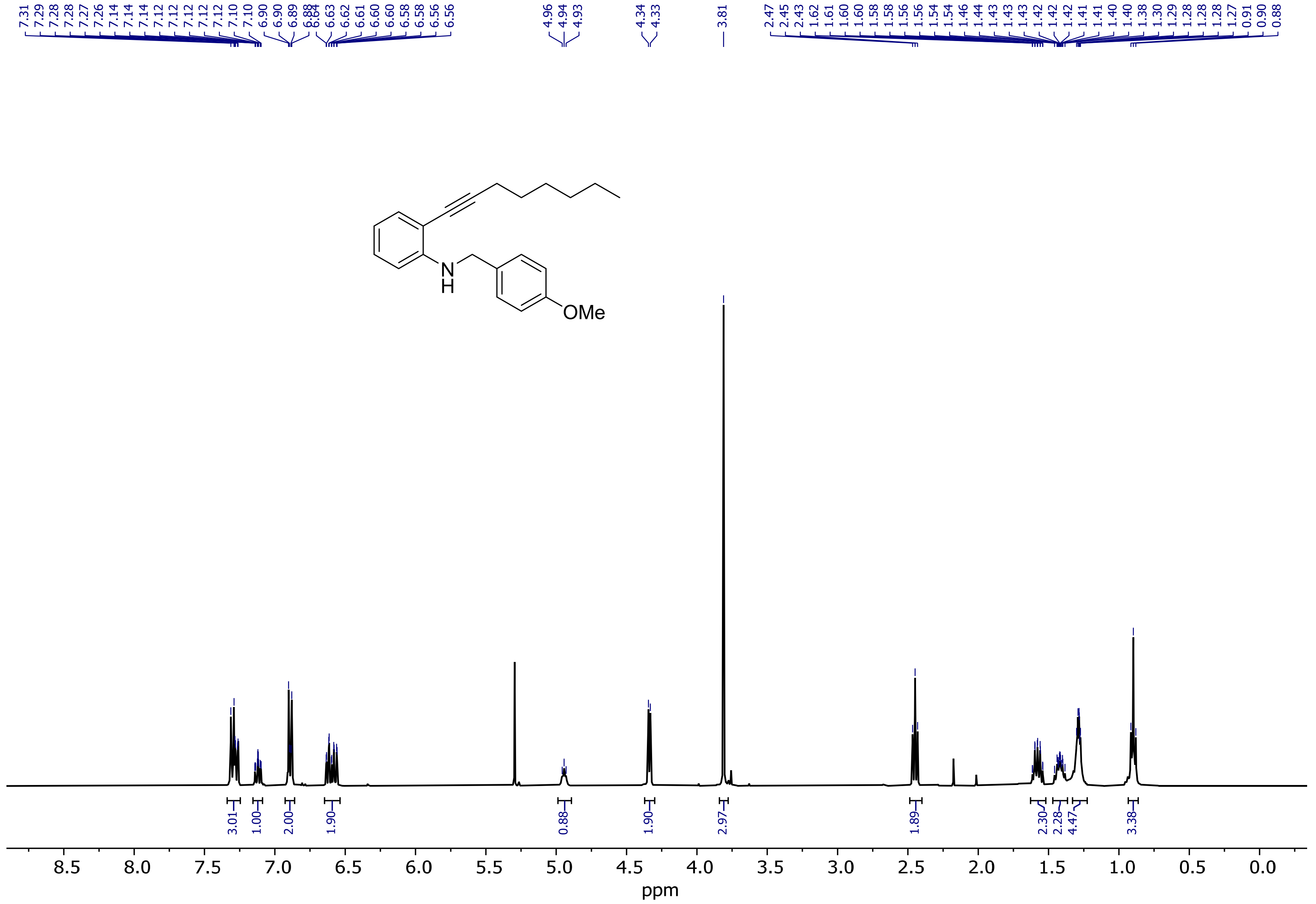

$^{13}C\ \{^1H\}$ NMR (100.62 MHz, $CDCl_3$) spectrum of 9a

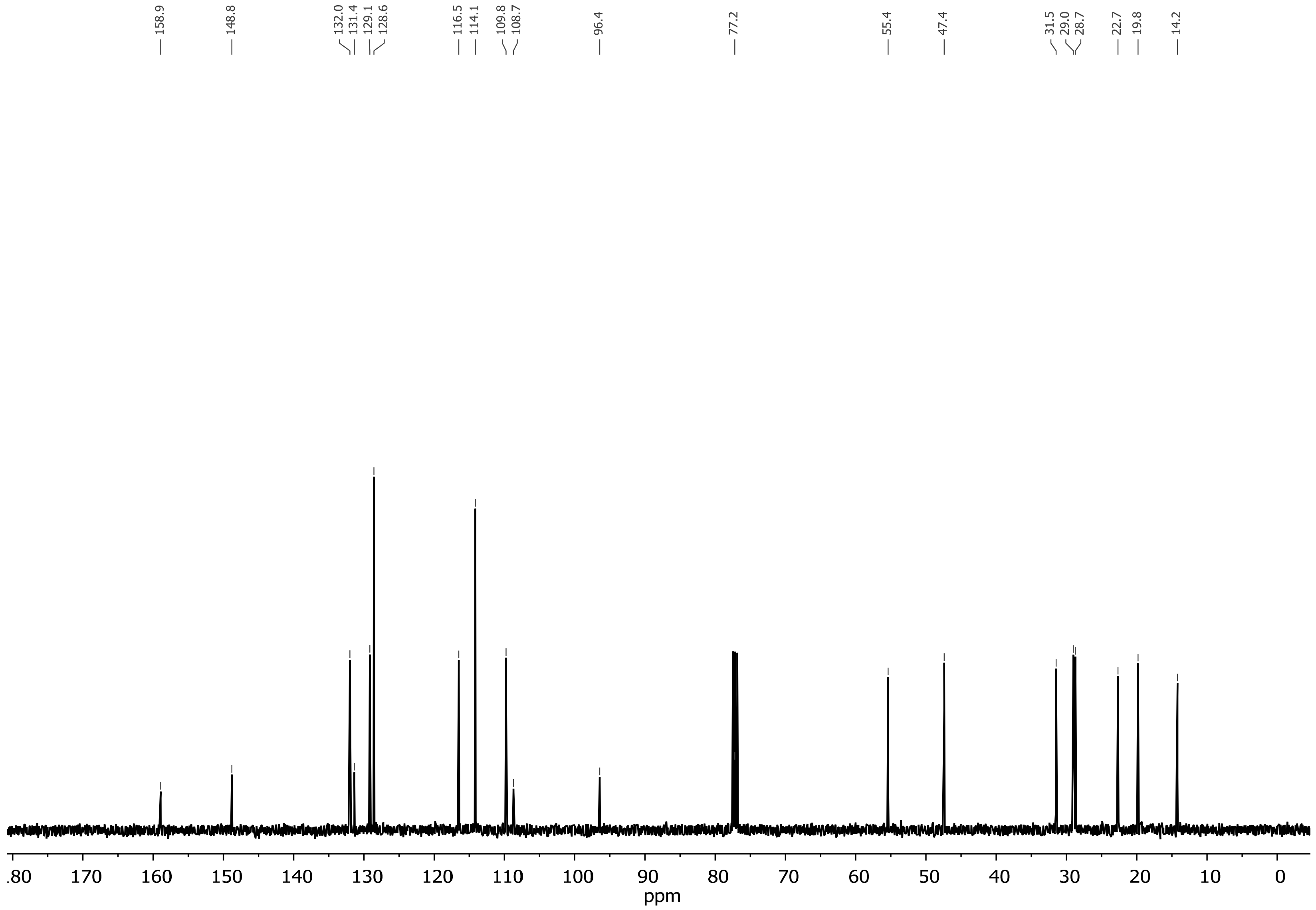

^{1}H NMR (400.16 MHz, CDCl$_3$) spectrum of 9b

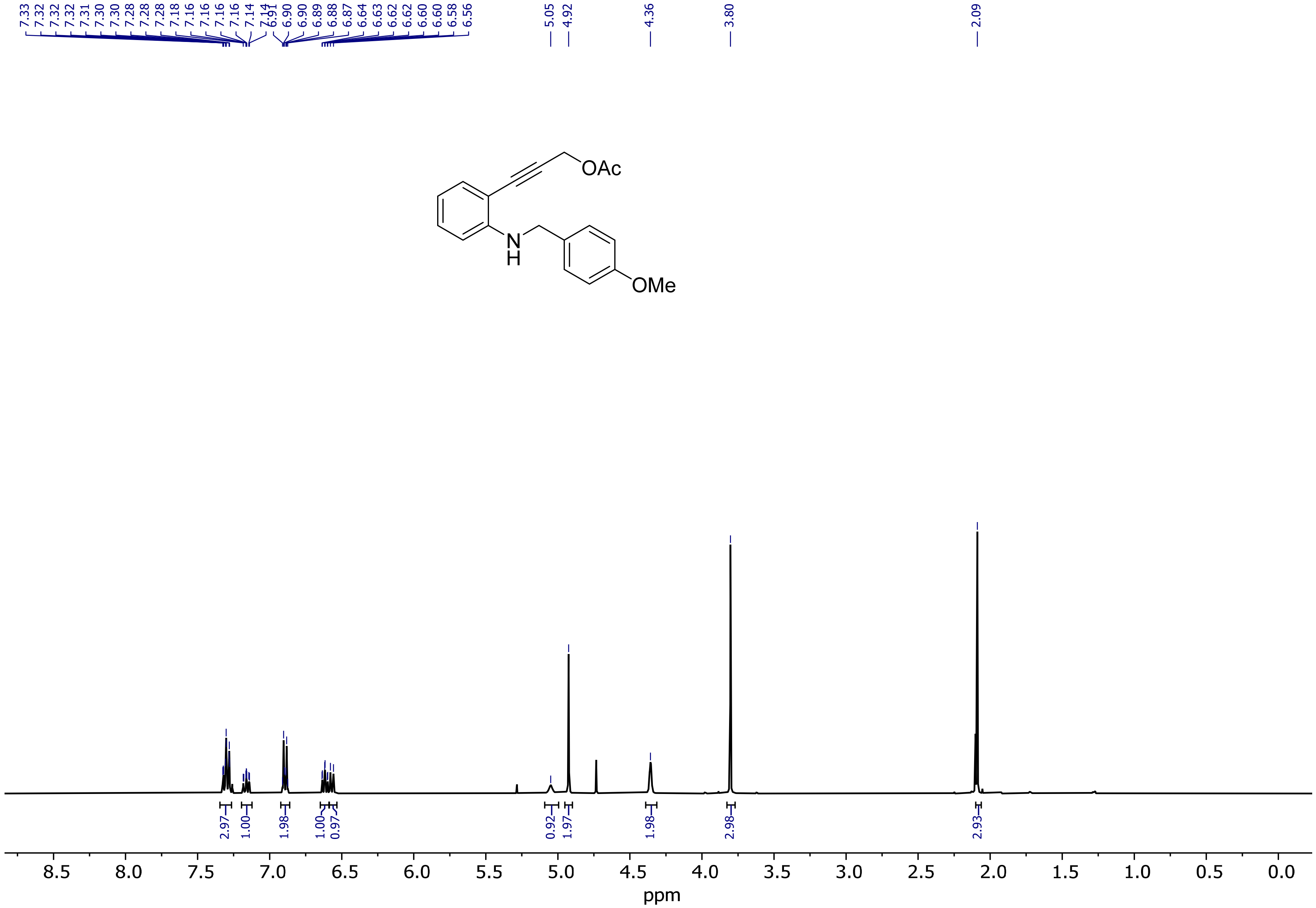

$^{13}C\{^1H\}$ NMR (100.62 MHz, $CDCl_3$) spectrum of 9b

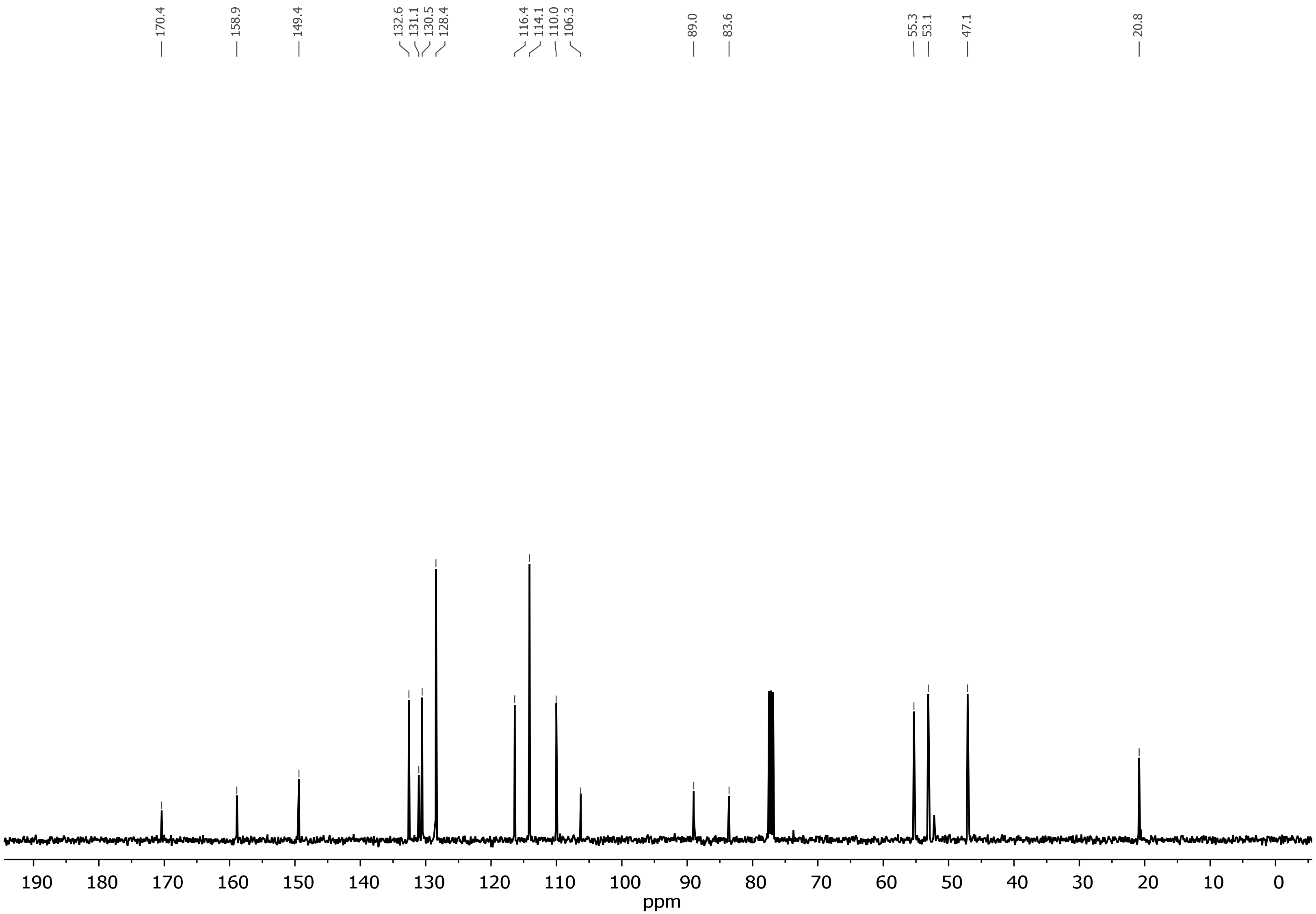

^{1}H NMR (400.16 MHz, CDCl$_3$) spectrum of 9c

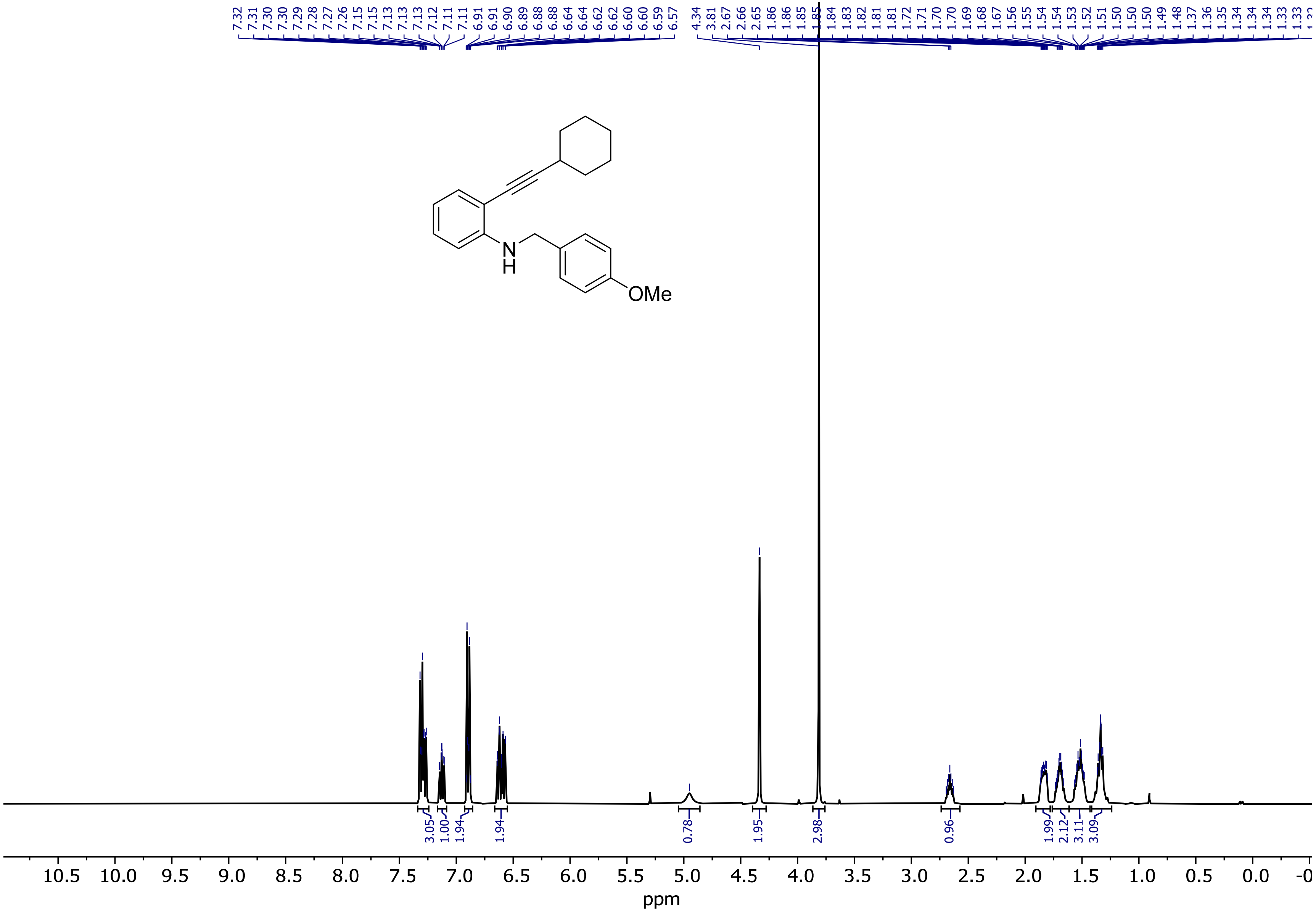

$^{13}C\{^1H\}$ NMR (100.62 MHz, $CDCl_3$) spectrum of 9c

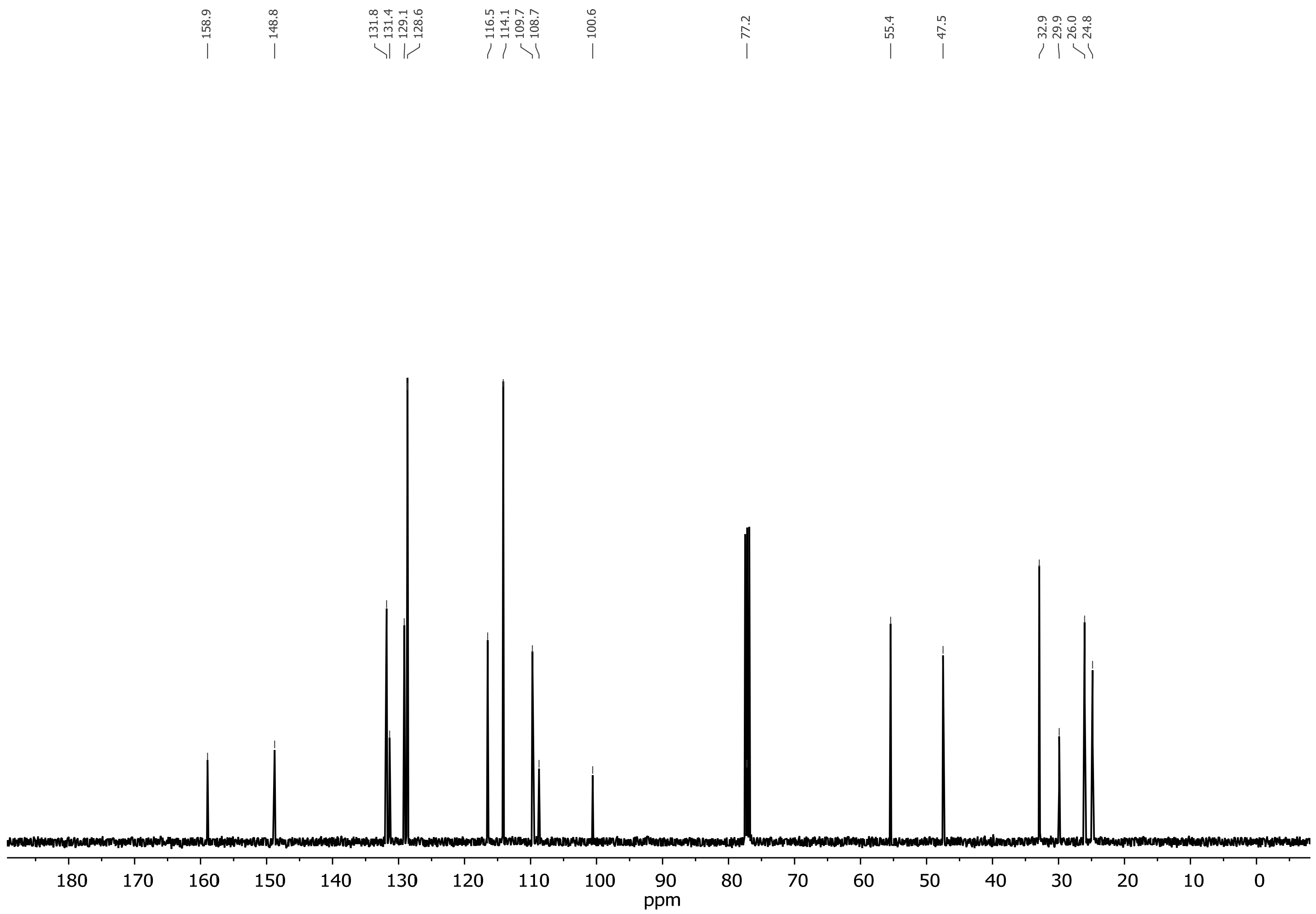

^{1}H NMR (400.16 MHz, $CDCl_3$) spectrum of 9d

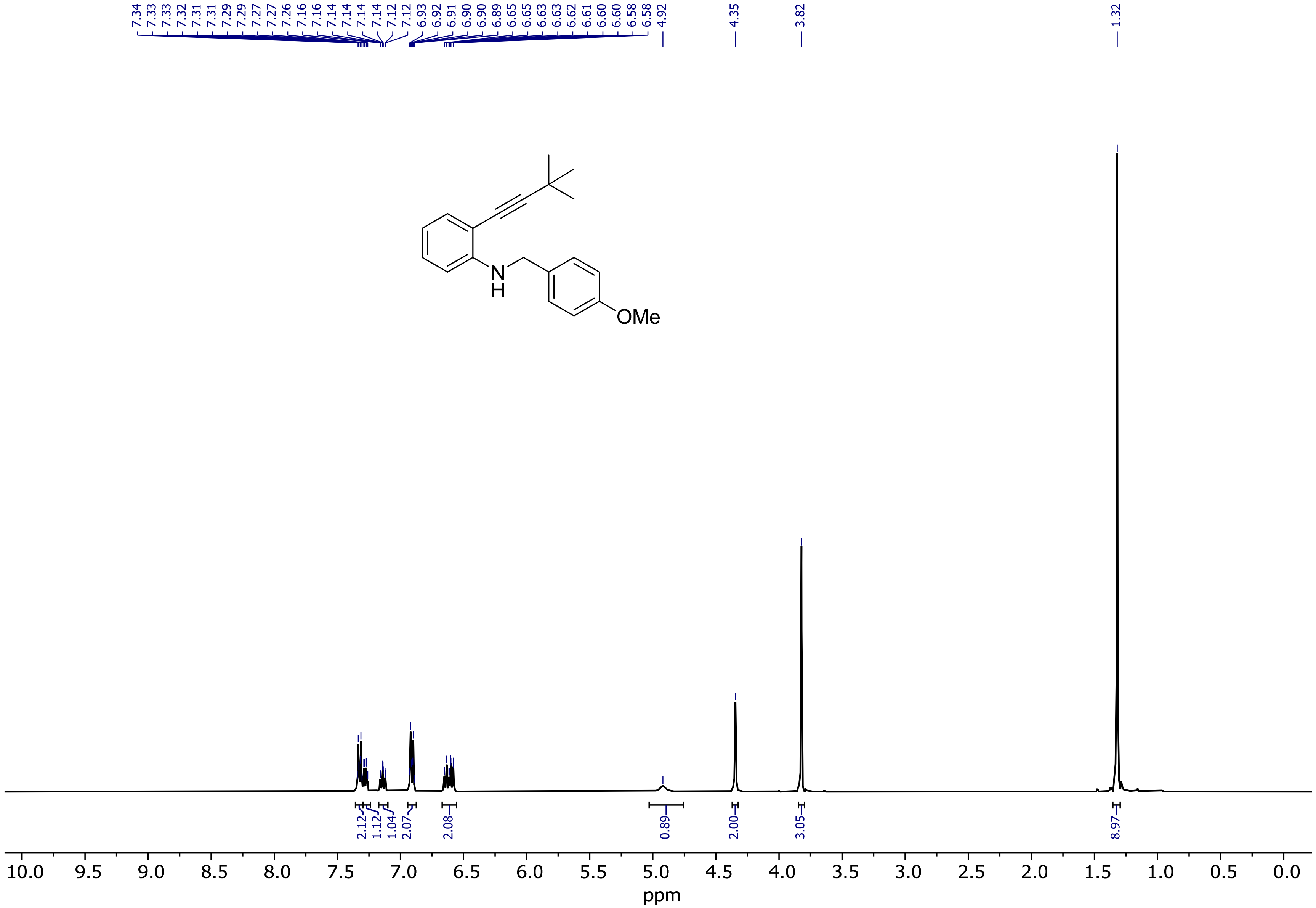

$^{13}C\{^{1}H\}$ NMR (100.62 MHz, $CDCl_3$) spectrum of 9d

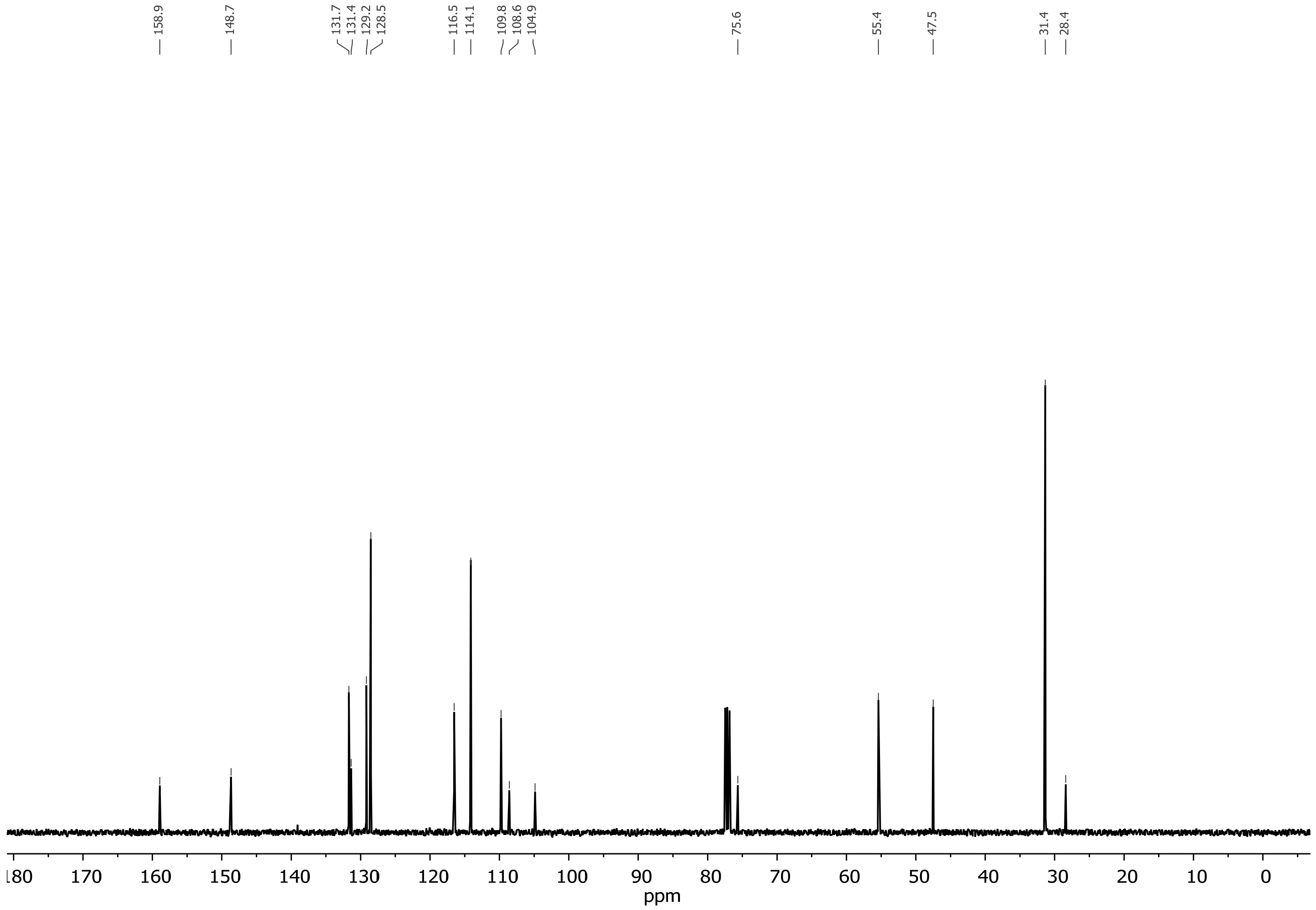

^{1}H NMR (400.16 MHz, $CDCl_3$) spectrum of 9e

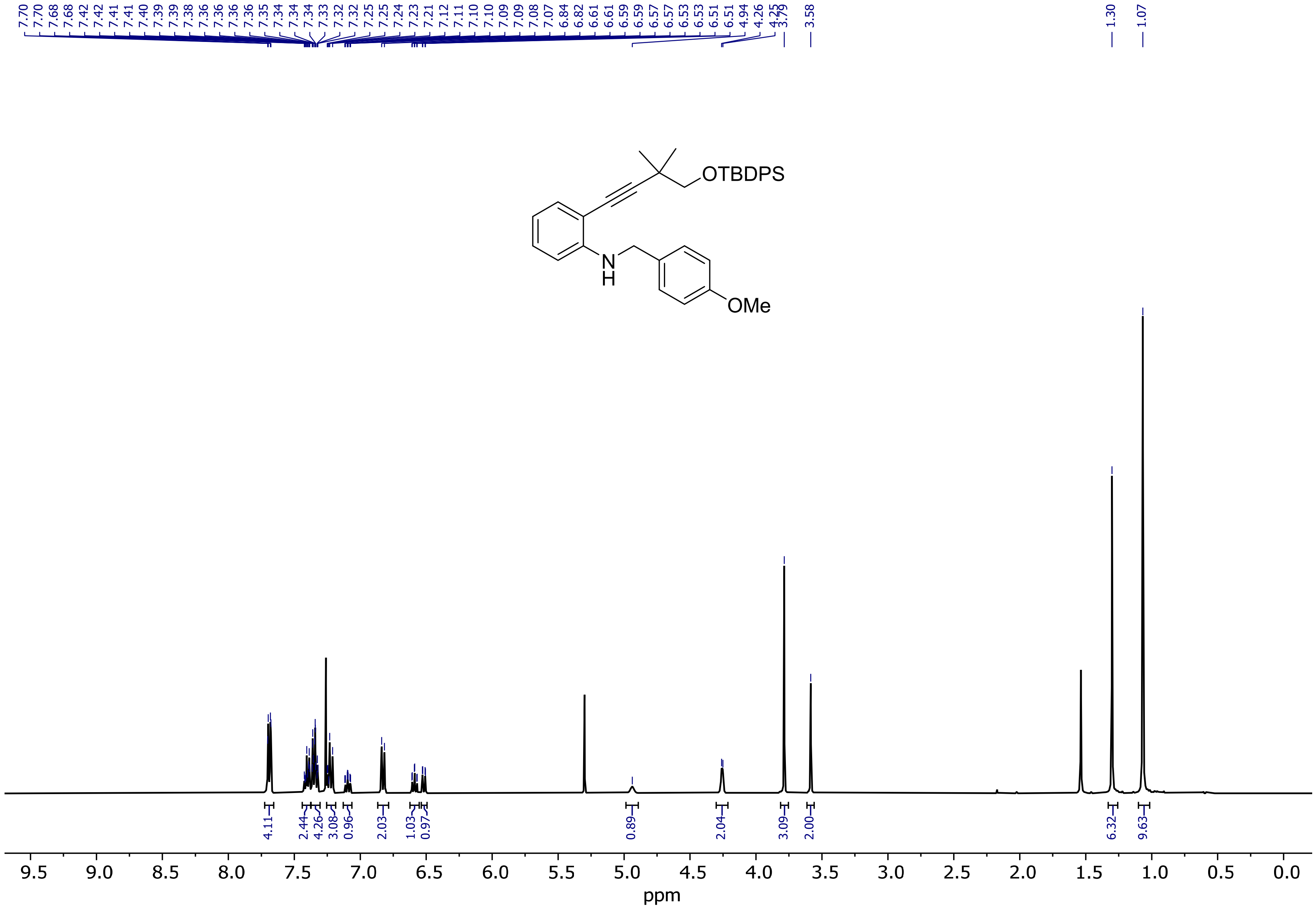

$^{13}C\{^{1}H\}$ NMR (100.62 MHz, $CDCl_3$) spectrum of 9e

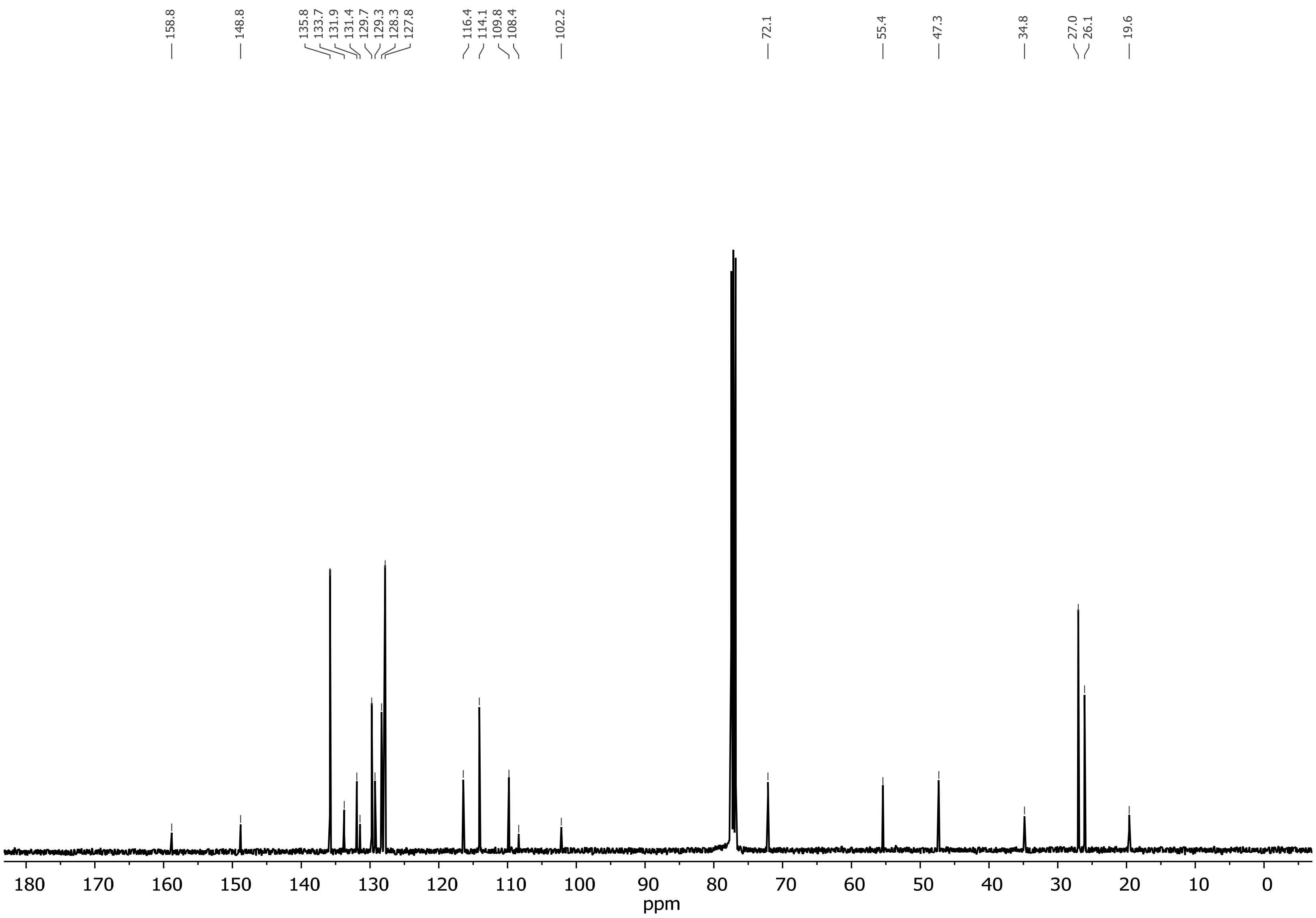

^{1}H NMR (400.16 MHz, $CDCl_3$) spectrum of 9f

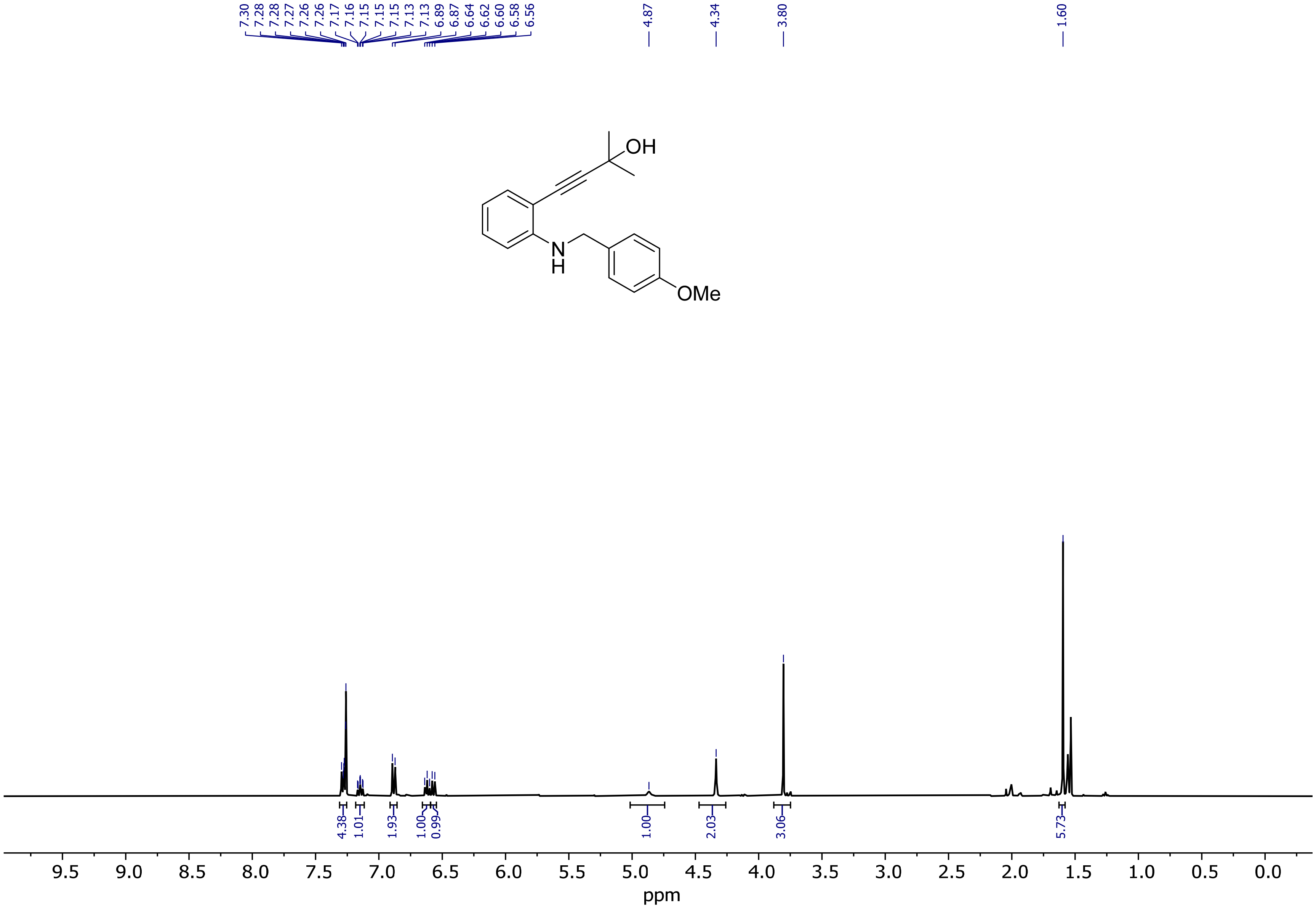

$^{13}C\{^1H\}$ NMR (100.62 MHz, $CDCl_3$) spectrum of 9f

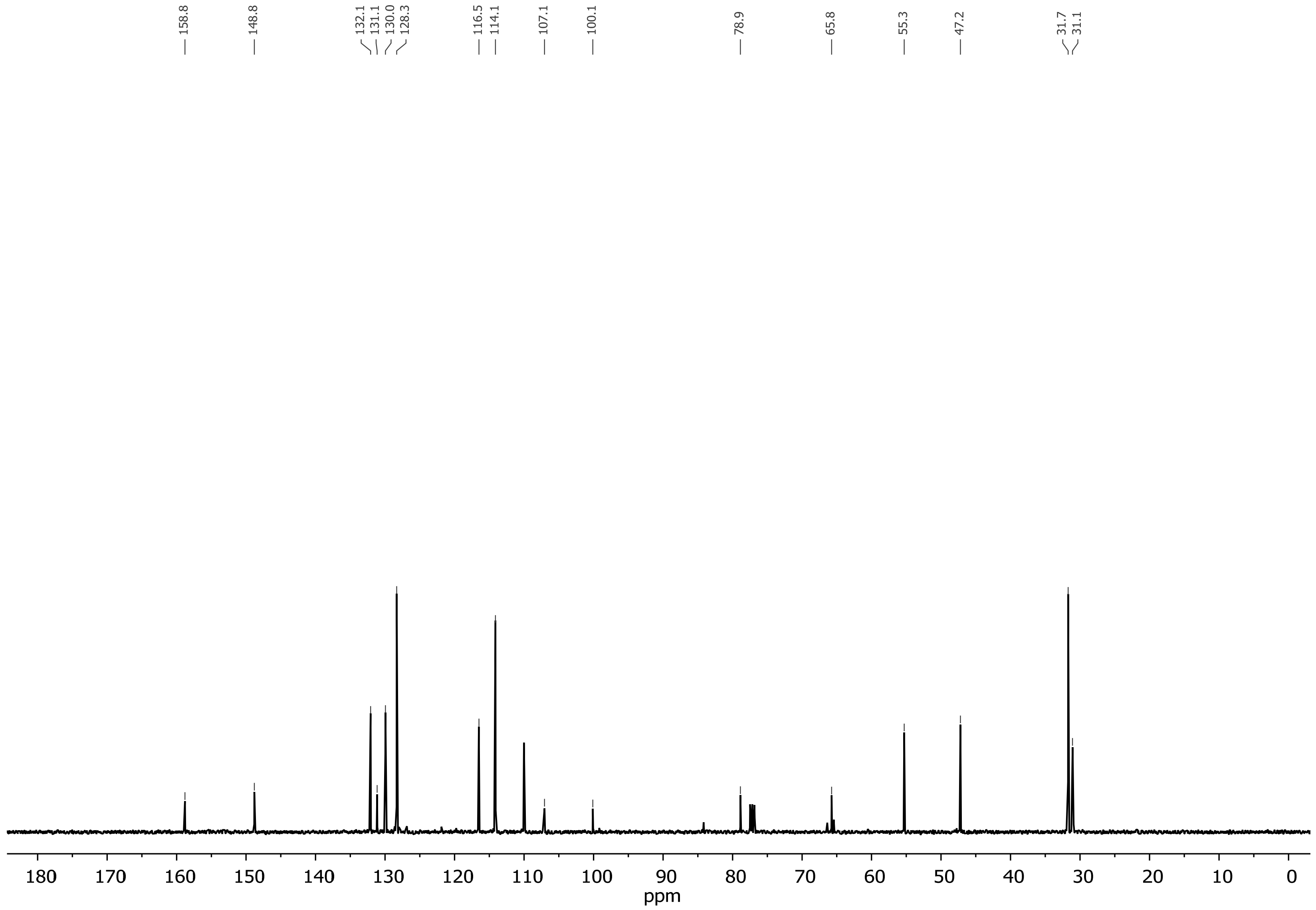

^{1}H NMR (400.16 MHz, CDCl$_3$) spectrum of 9g

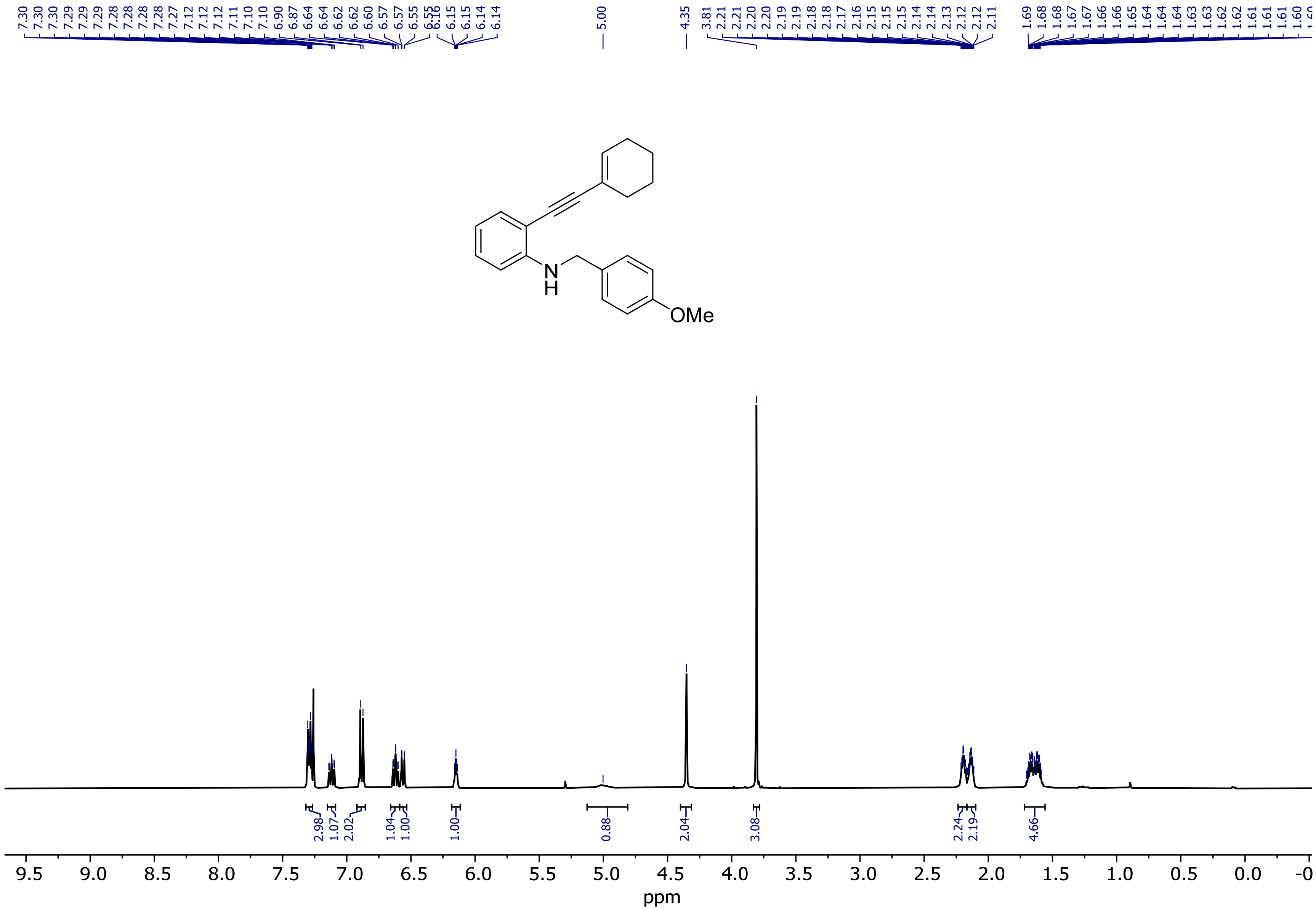

$^{13}C\ \{^{1}H\}$ NMR (100.62 MHz, $CDCl_3$) spectrum of 9g

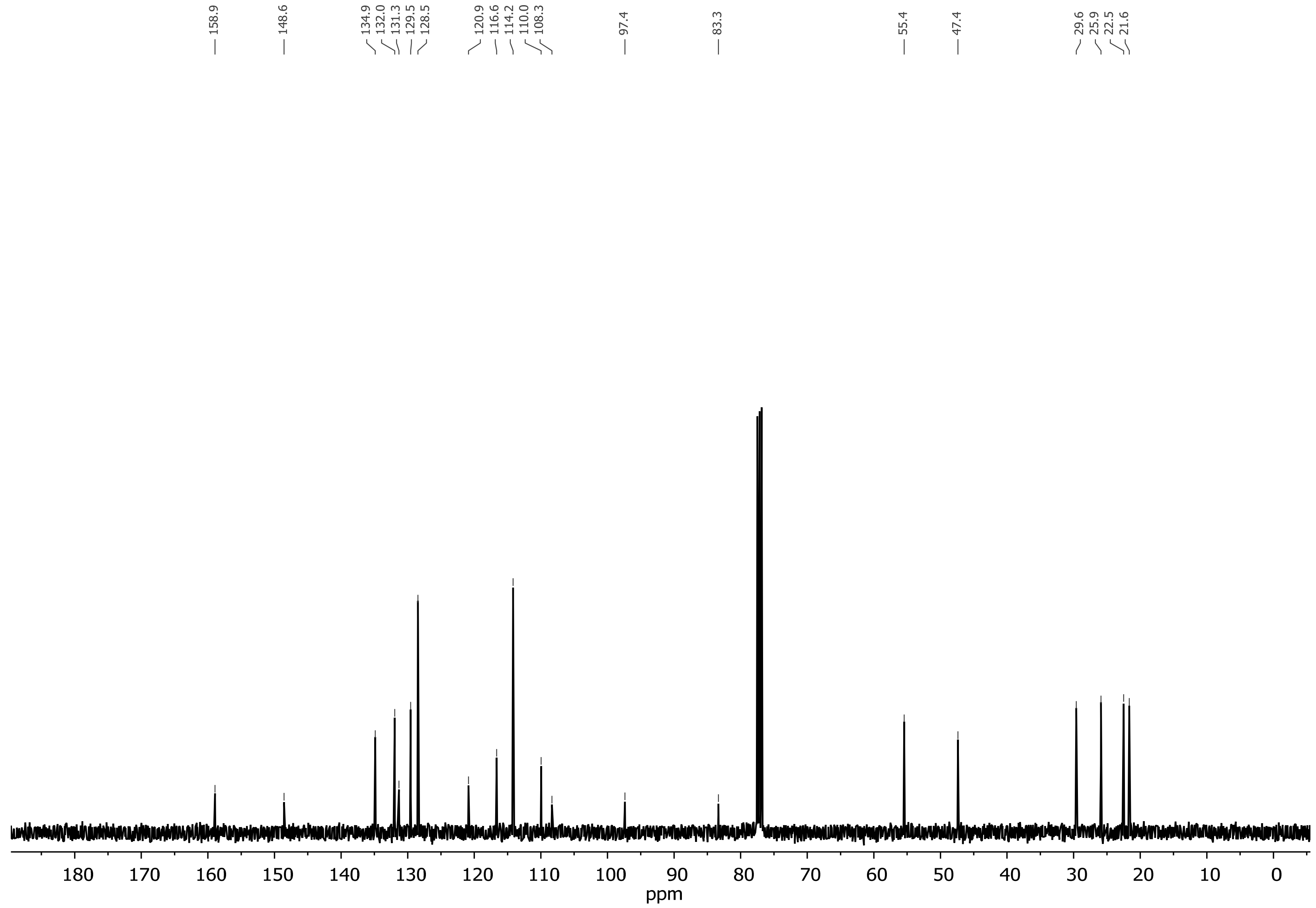

^{1}H NMR (400.16 MHz, $CDCl_3$) spectrum of 9h

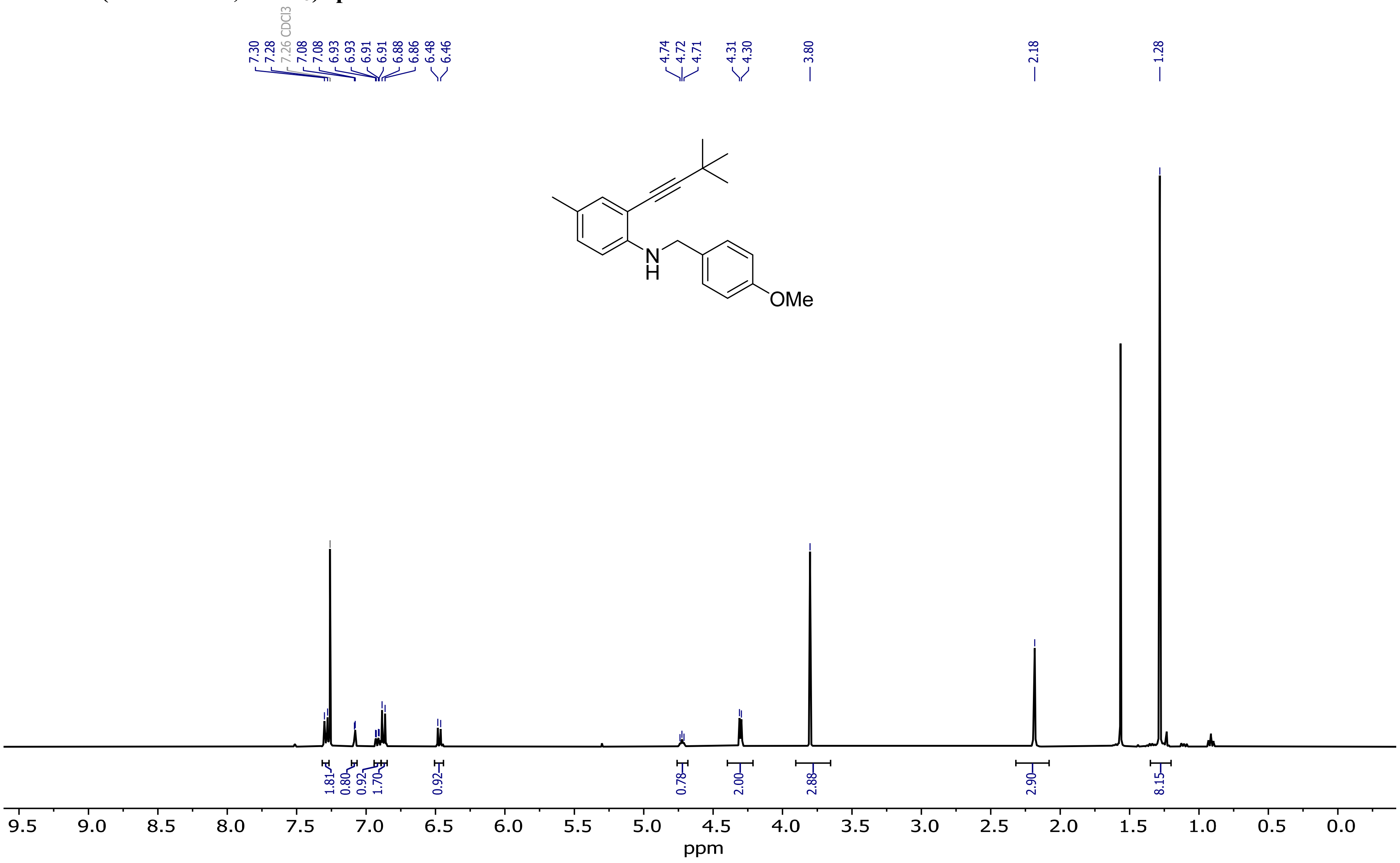

$^{13}C\ \{^{1}H\}$ NMR (100.62 MHz, $CDCl_3$) spectrum of 9h

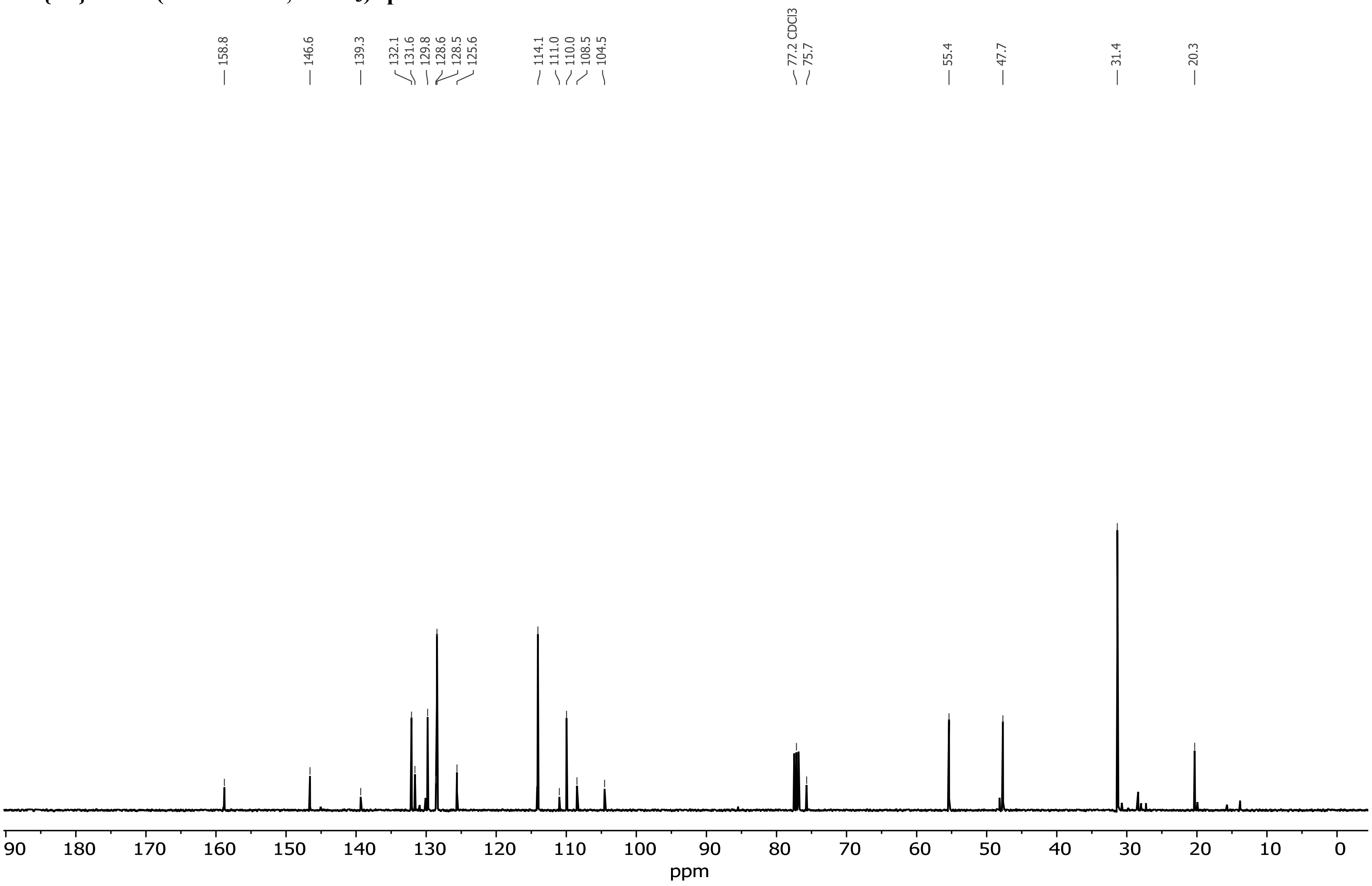

^{1}H NMR (400.16 MHz, $CDCl_3$) spectrum of 9i

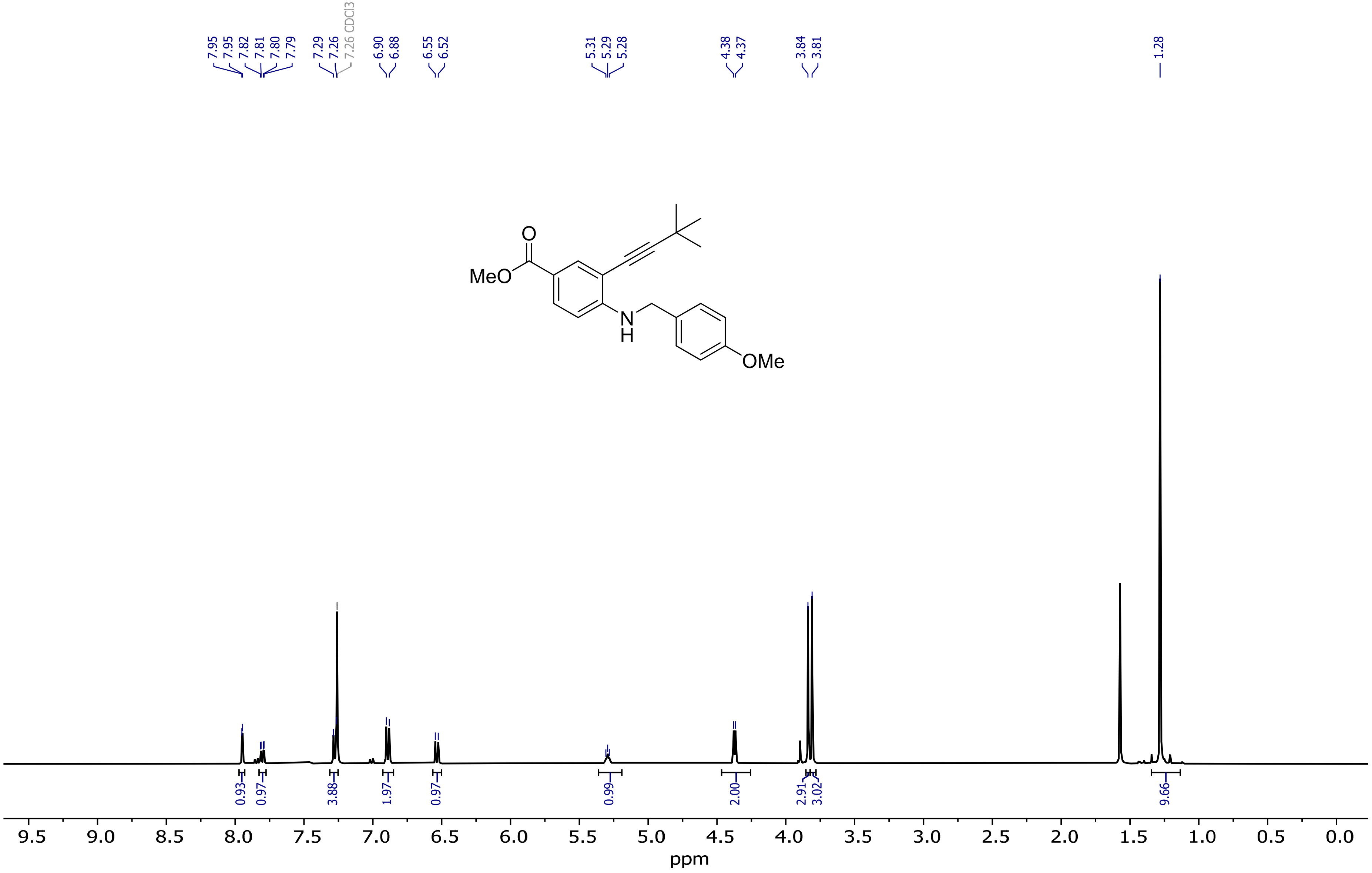

$^{13}C\{^1H\}$ NMR (100.62 MHz, $CDCl_3$) spectrum of 9i

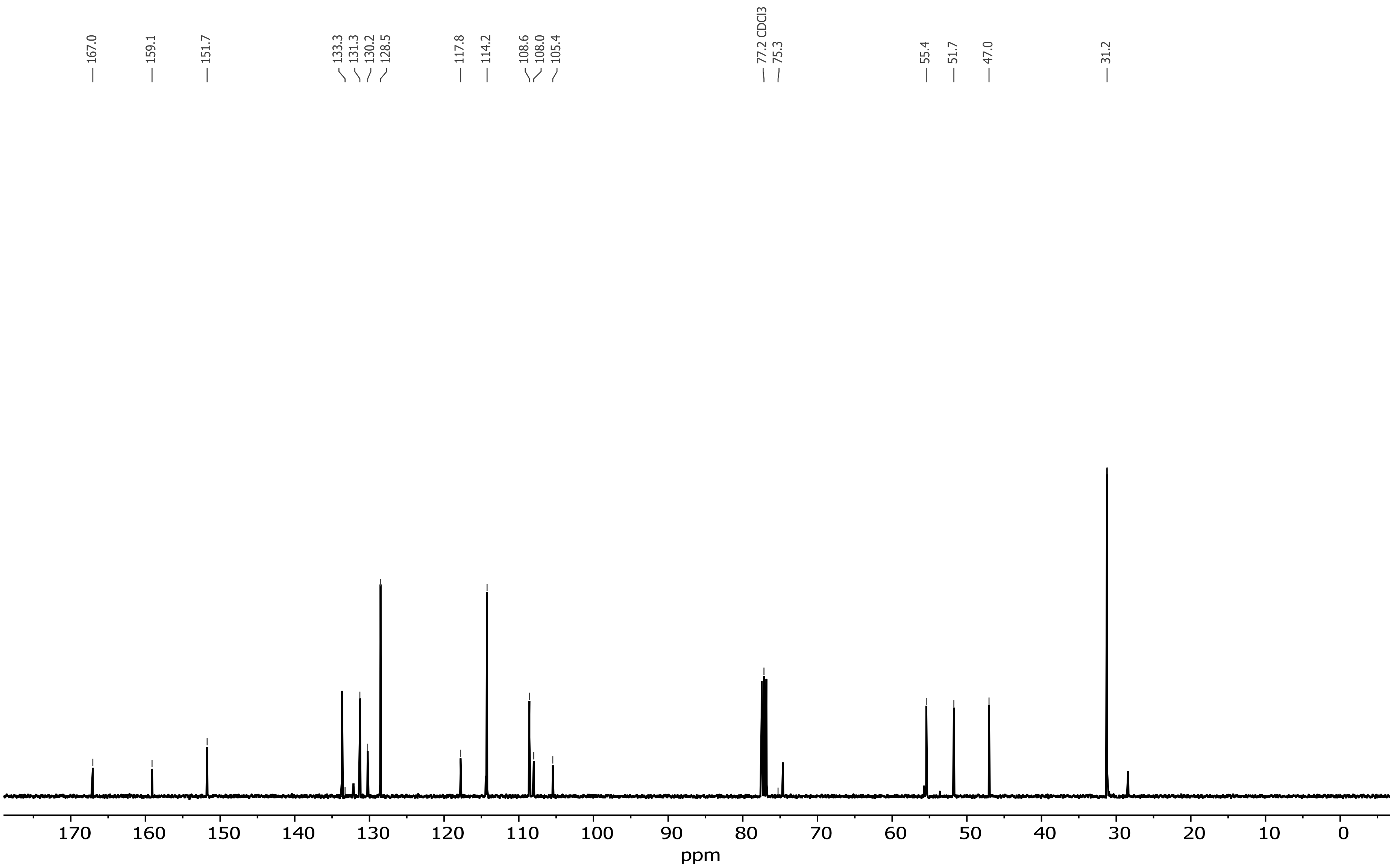

^{1}H NMR (400.16 MHz, $CDCl_3$) spectrum of 9j

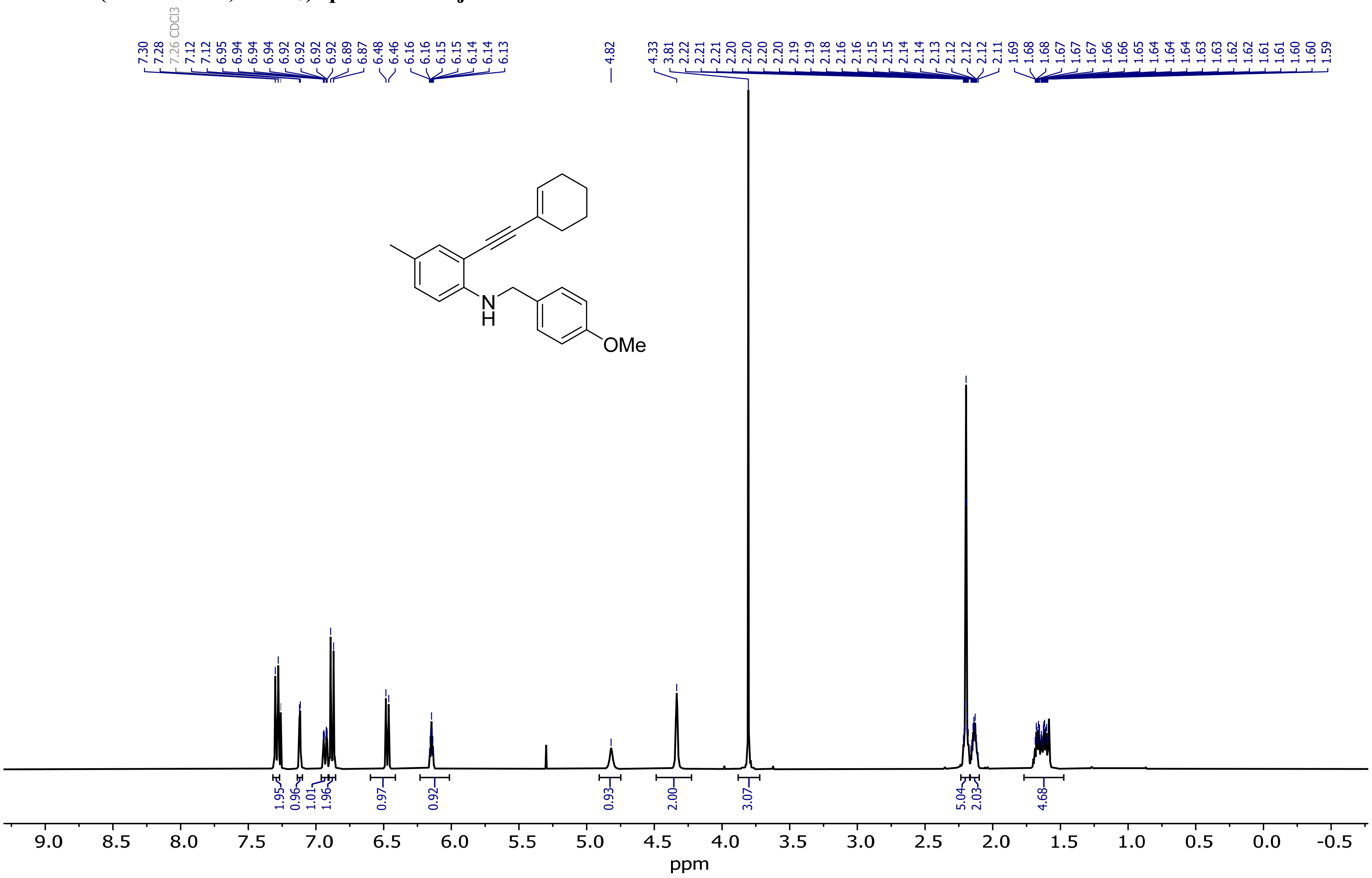

$^{13}C\ \{^1H\}$ NMR (100.62 MHz, $CDCl_3$) spectrum of 9j

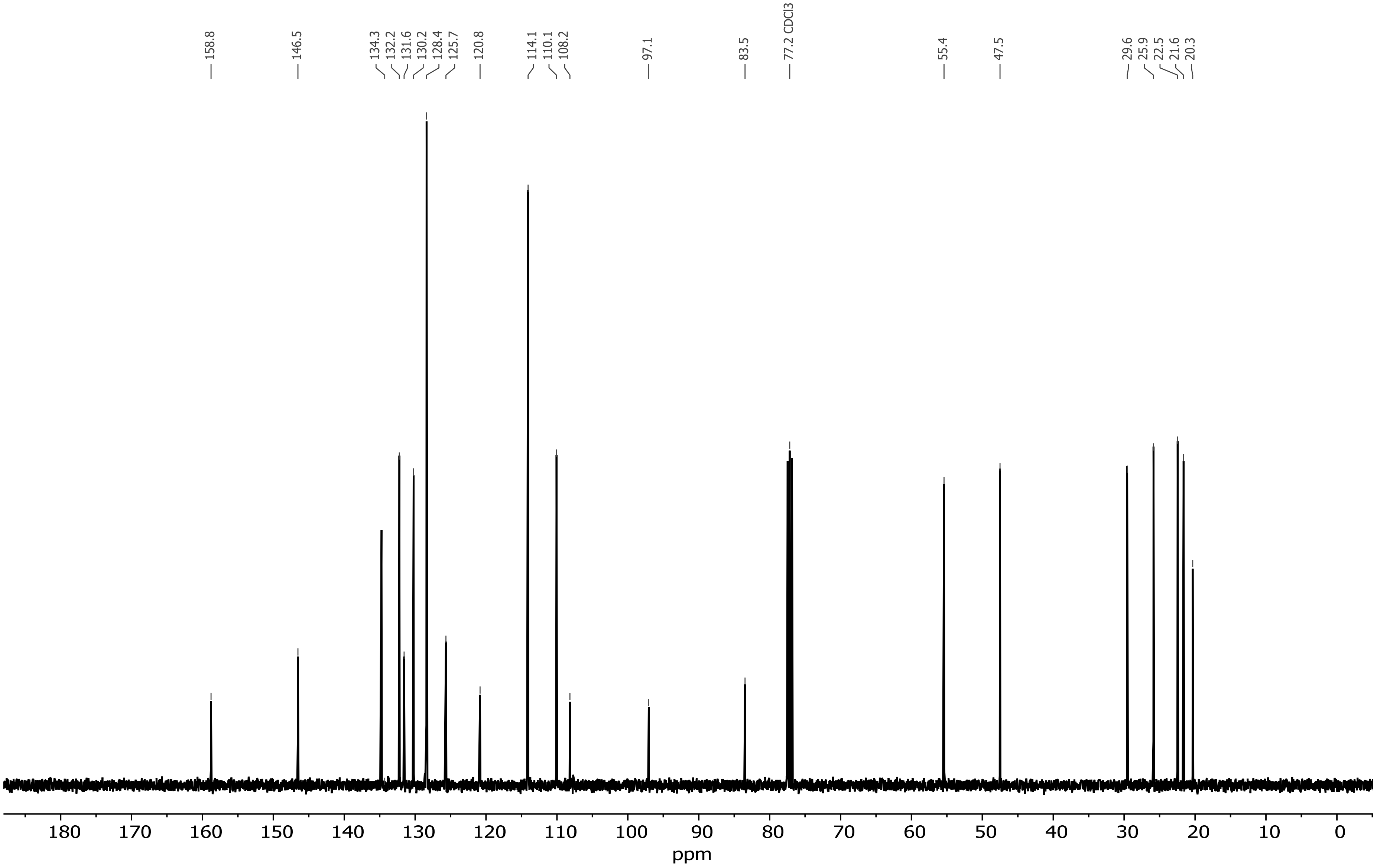

^{1}H NMR (400.16 MHz, $CDCl_3$) spectrum of 9k

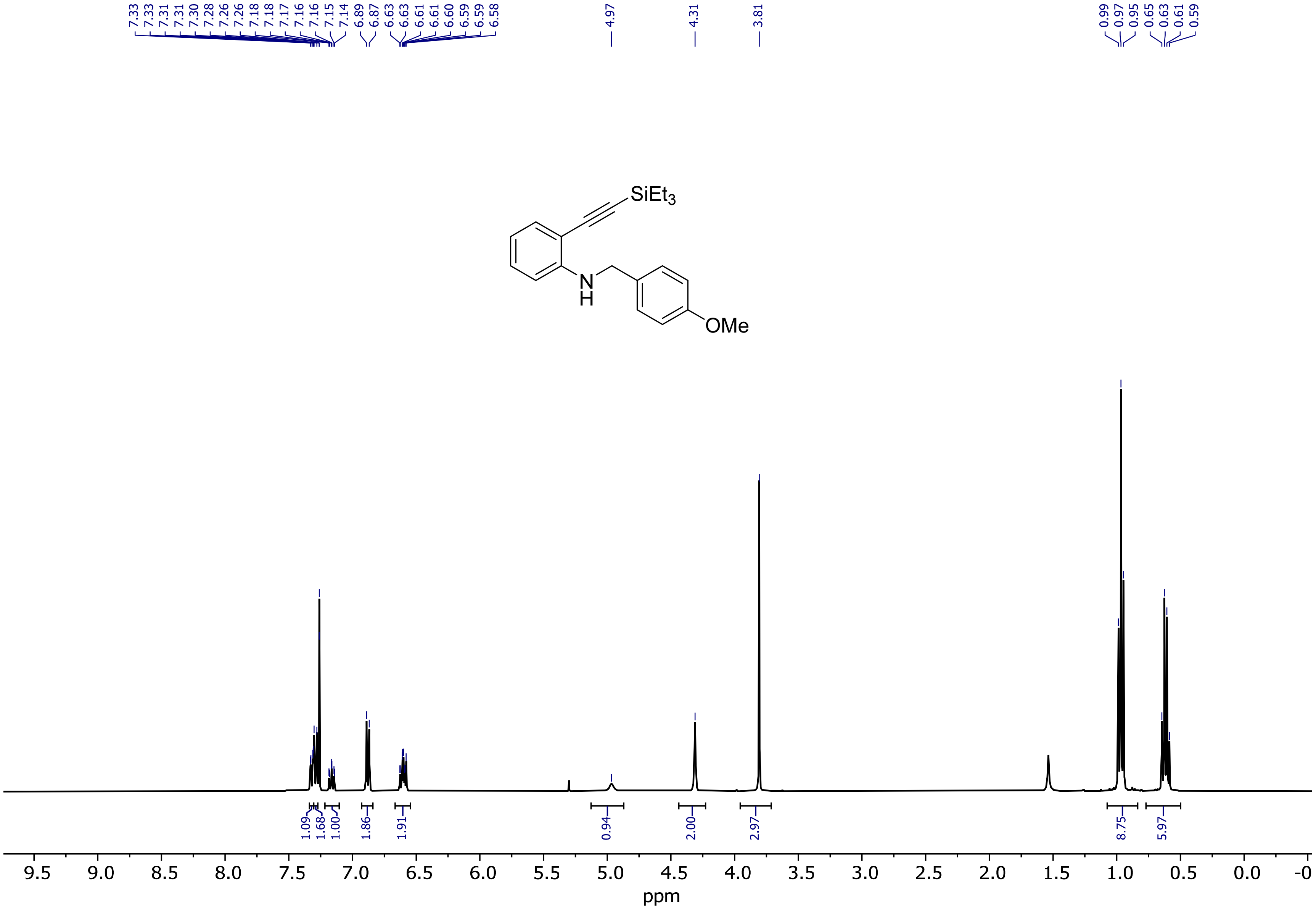

$^{13}C\ \{^{1}H\}$ NMR (100.62 MHz, $CDCl_3$) spectrum of 9k

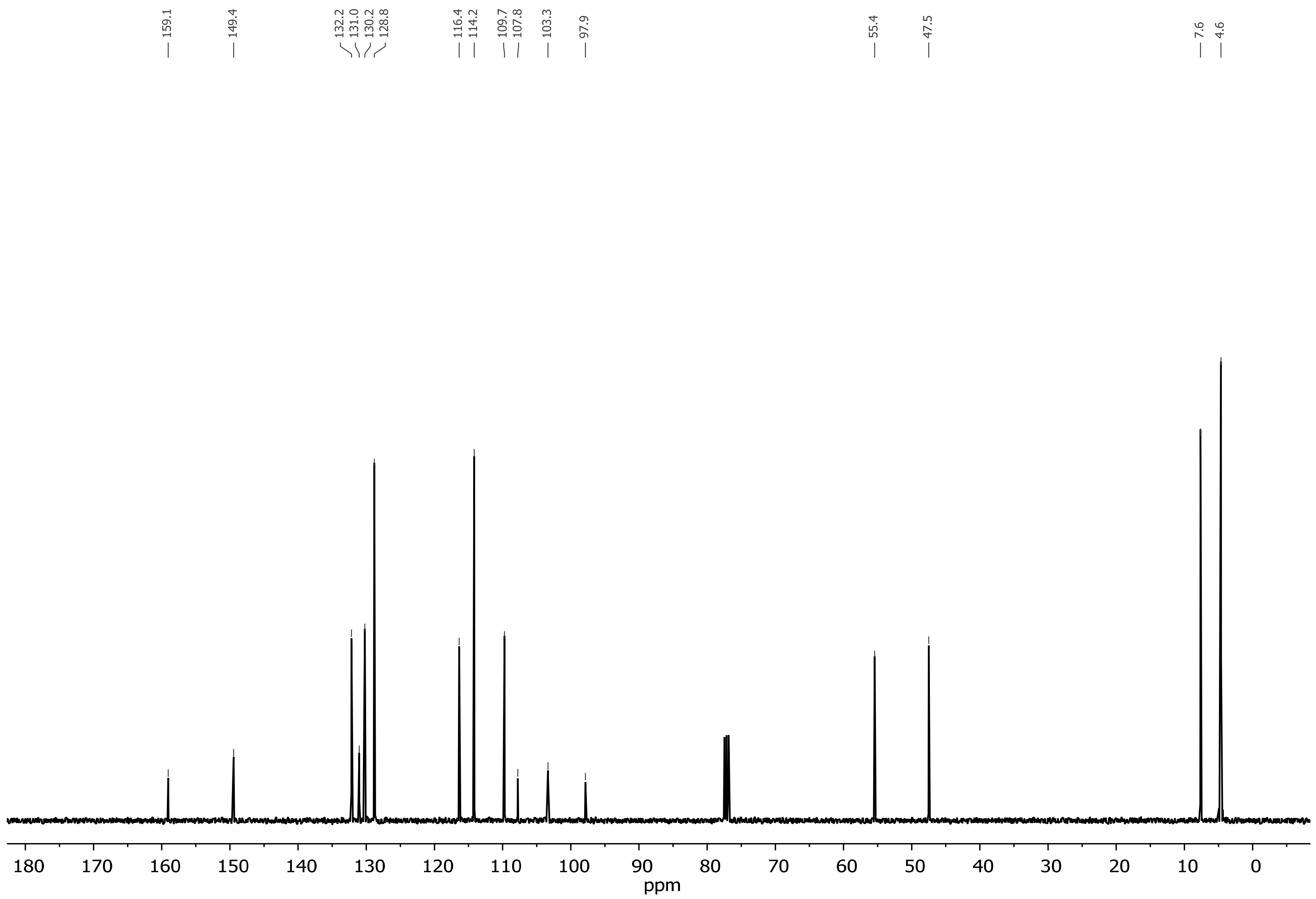

^{1}H NMR (400.16 MHz, acetone-d$_6$) spectrum of 4a

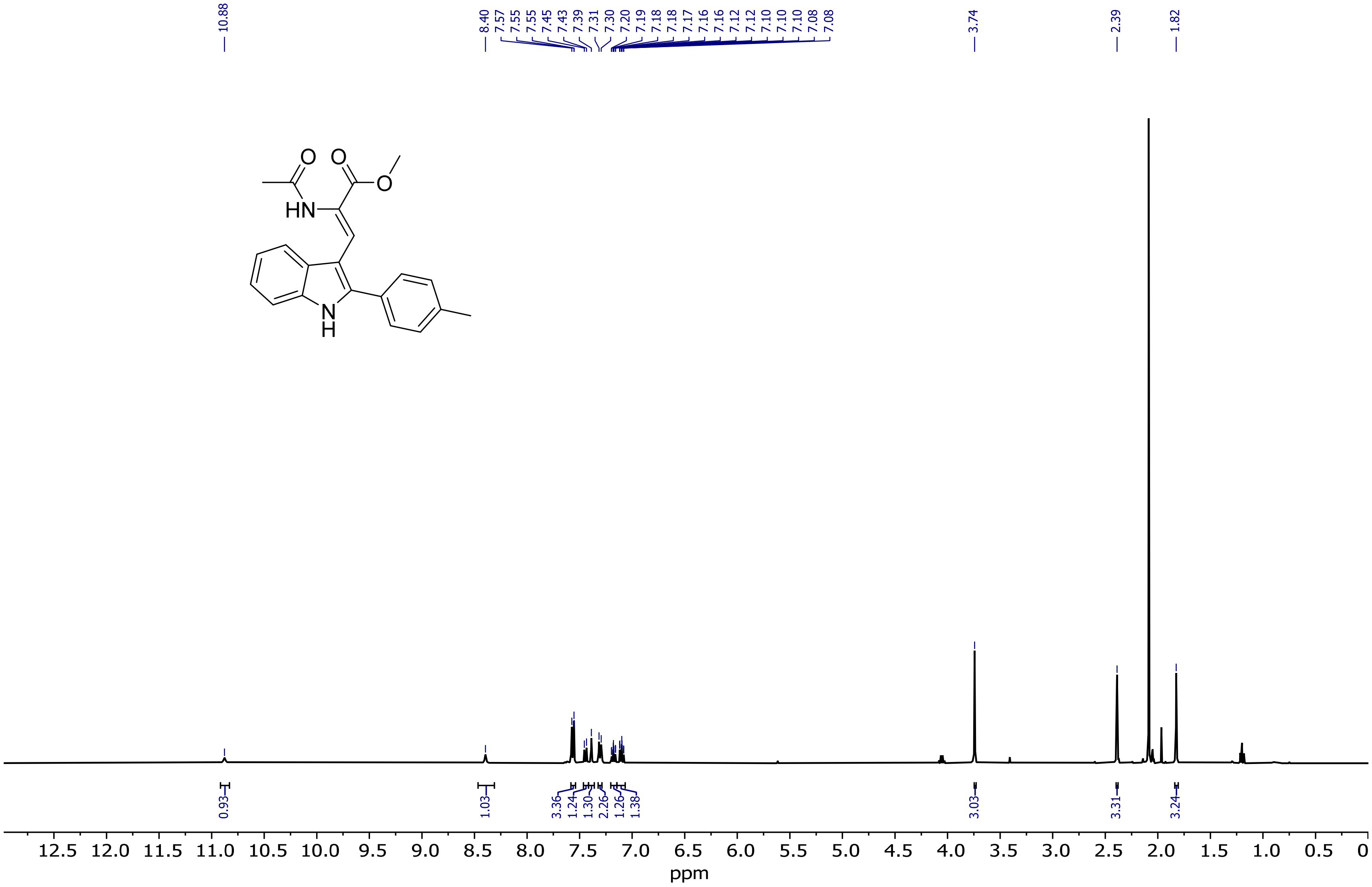

^{13}C {^{1}H} NMR (100.62 MHz, acetone-d_6) spectrum of 4a

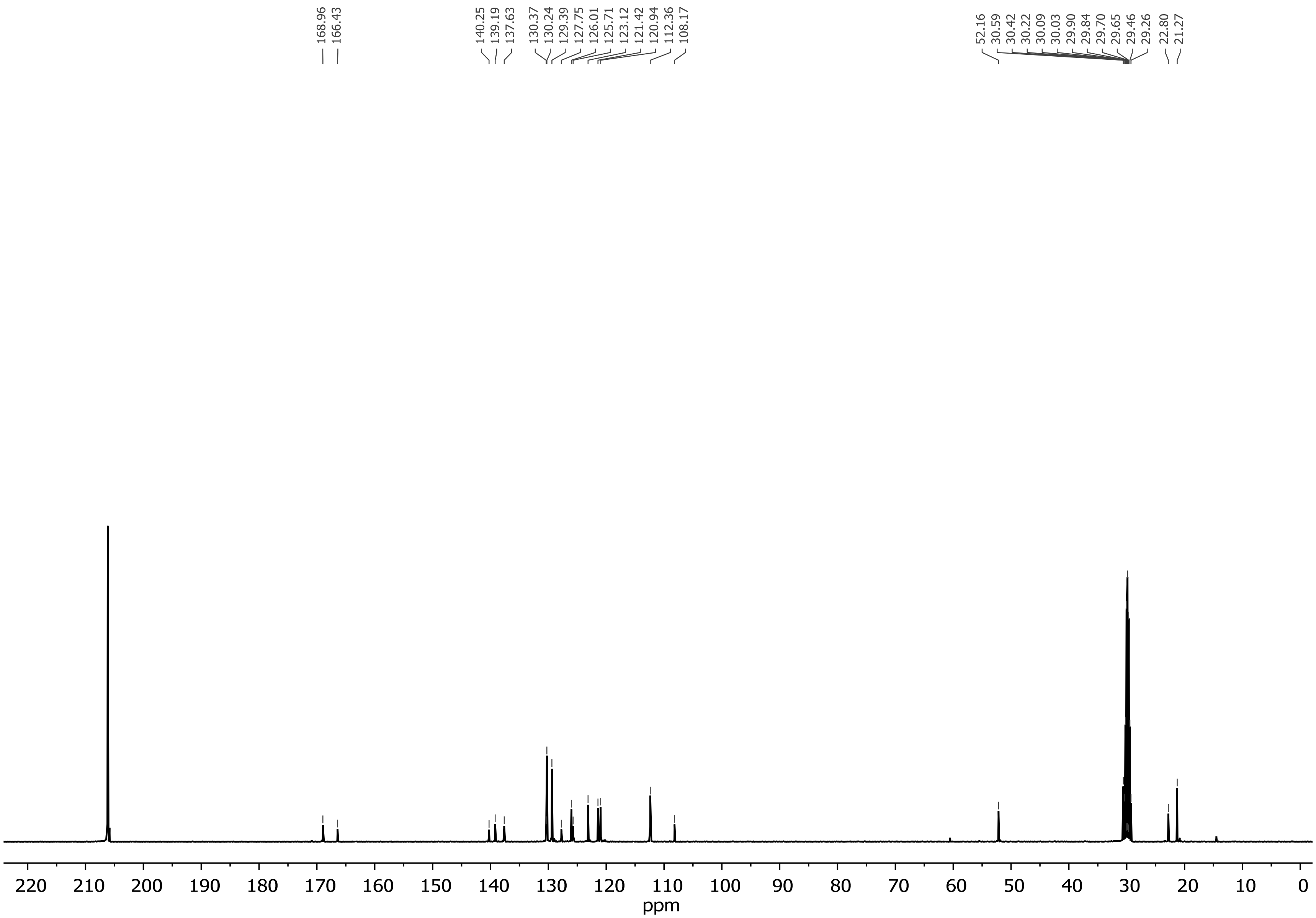

^{1}H NMR (400.16 MHz, acetone-d$_6$) spectrum of 6a

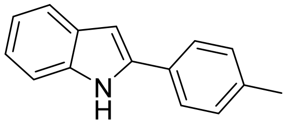

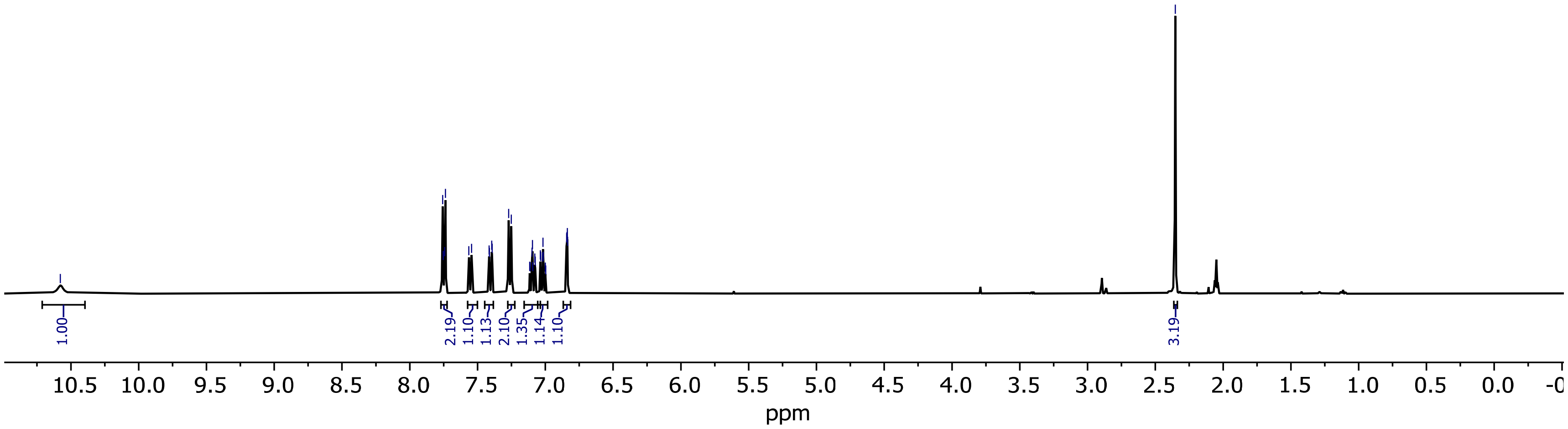

^{13}C {^{1}H} NMR (100.62 MHz, acetone-d_6) spectrum of 6a

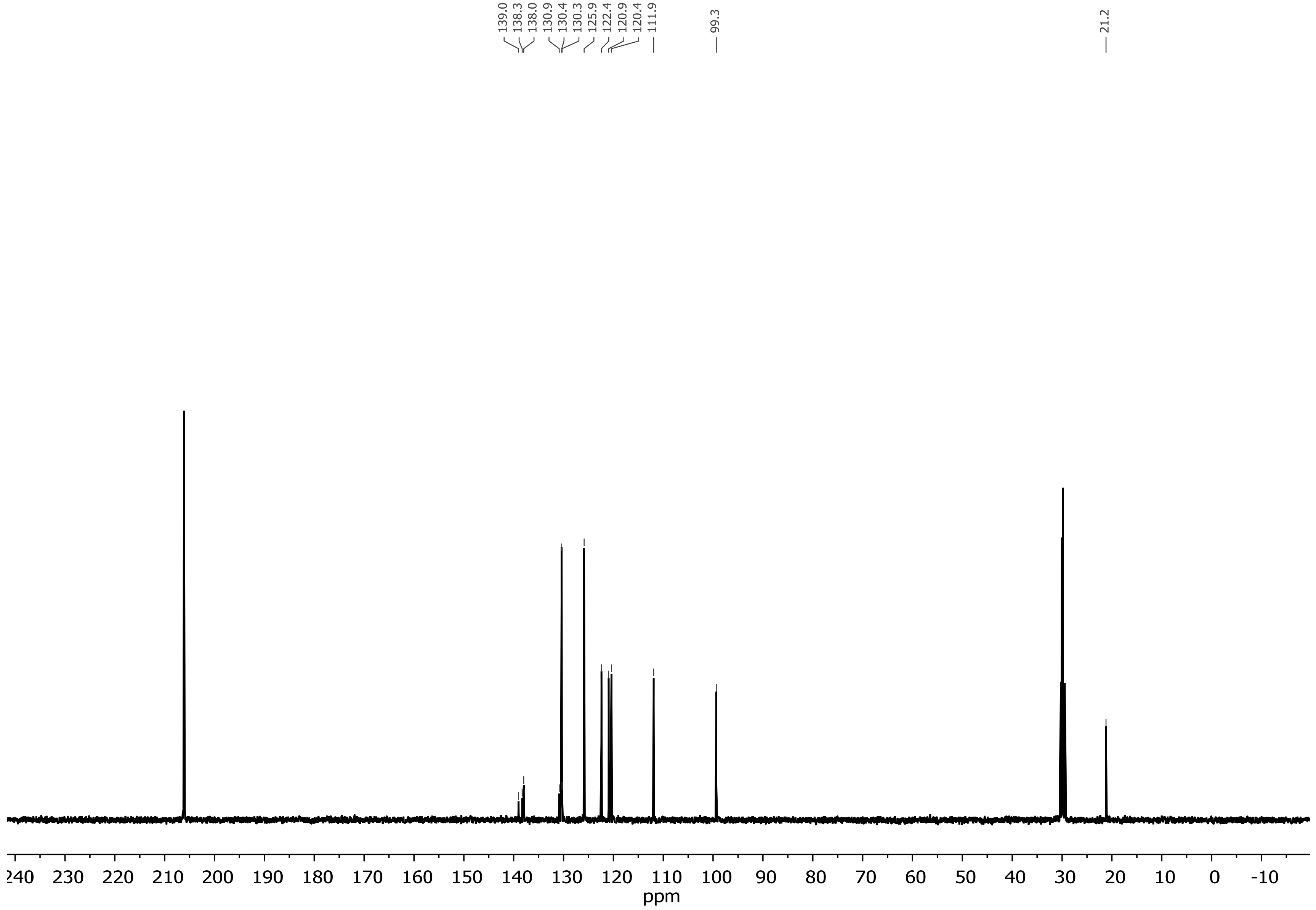

^{1}H NMR (400.16 MHz, $CDCl_3$) spectrum of 4b

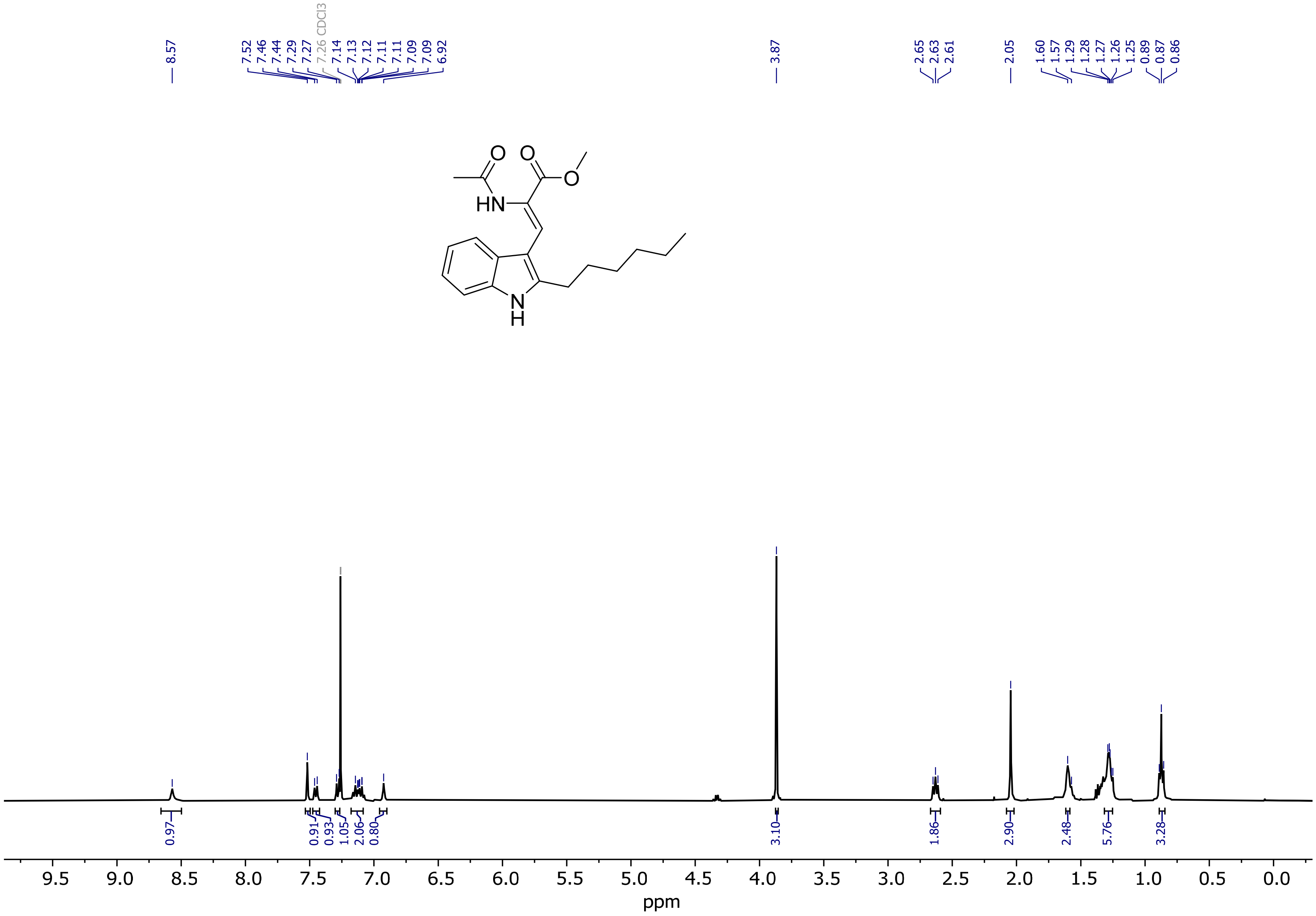

$^{13}C\ \{^1H\}$ NMR (100.62 MHz, $CDCl_3$) spectrum of 4b

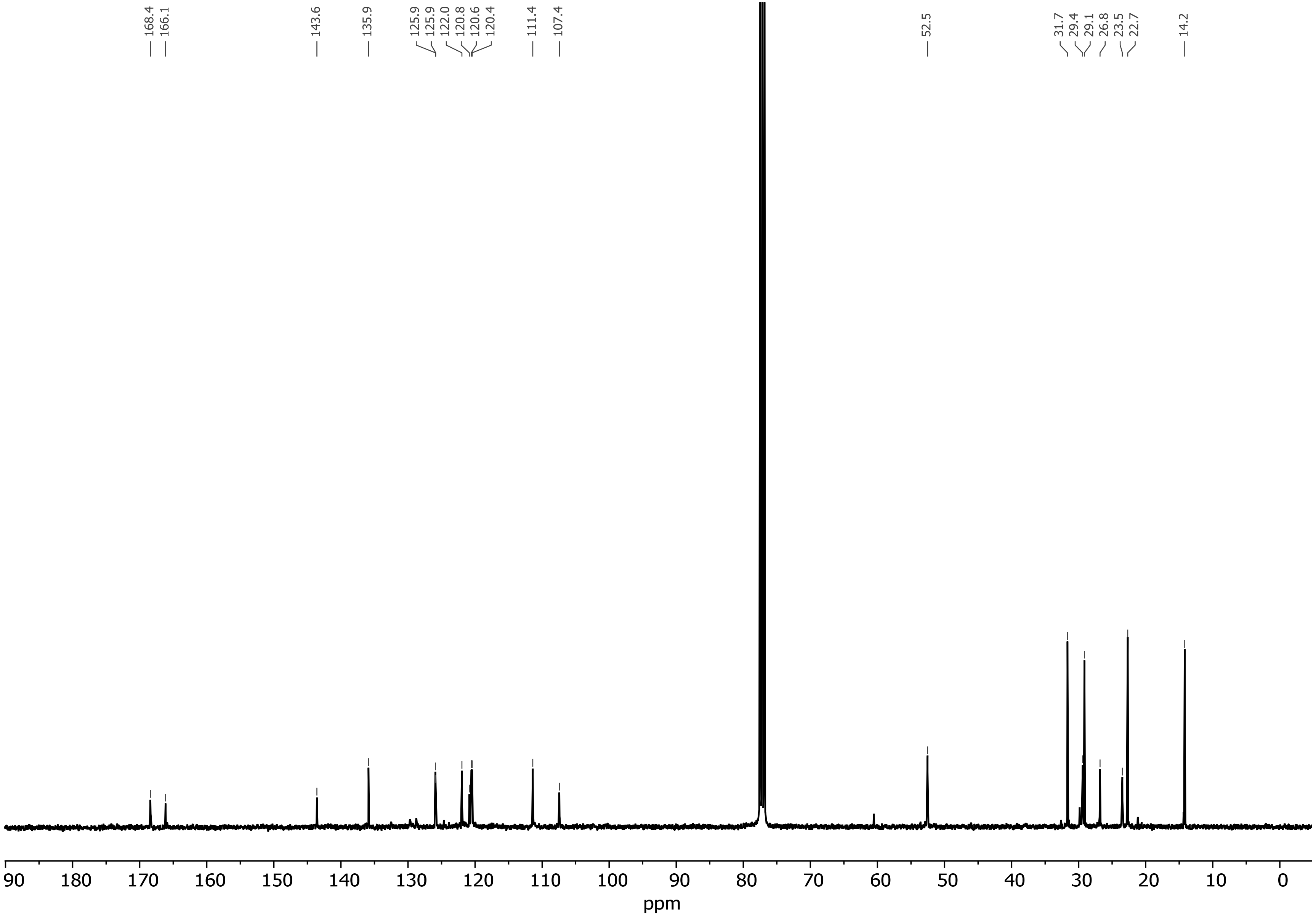

^{1}H NMR (400.16 MHz, CDCl$_3$) spectrum of 4c

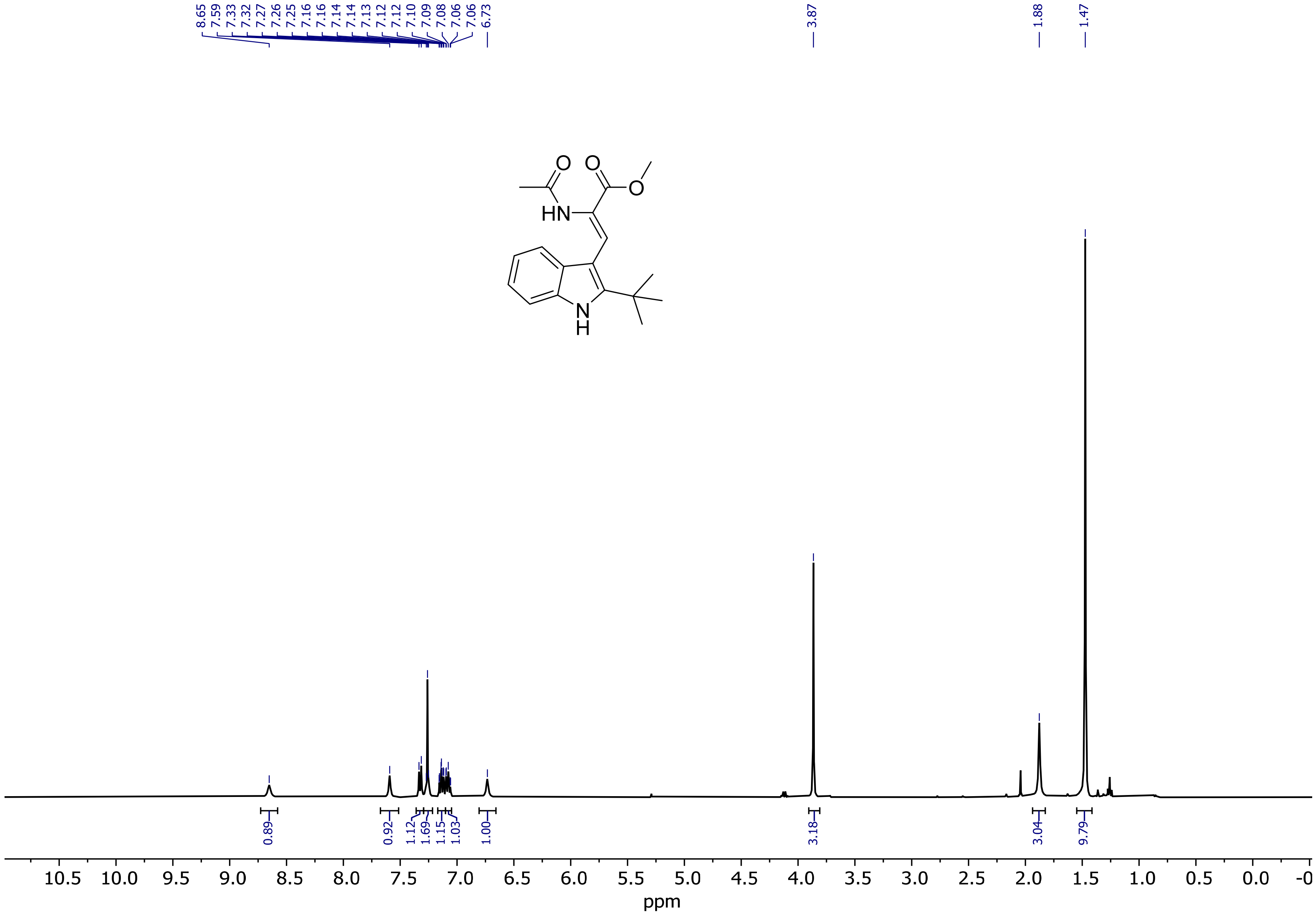

$^{13}C\ \{^1H\}$ NMR (100.62 MHz, CDCl$_3$) spectrum of 4c

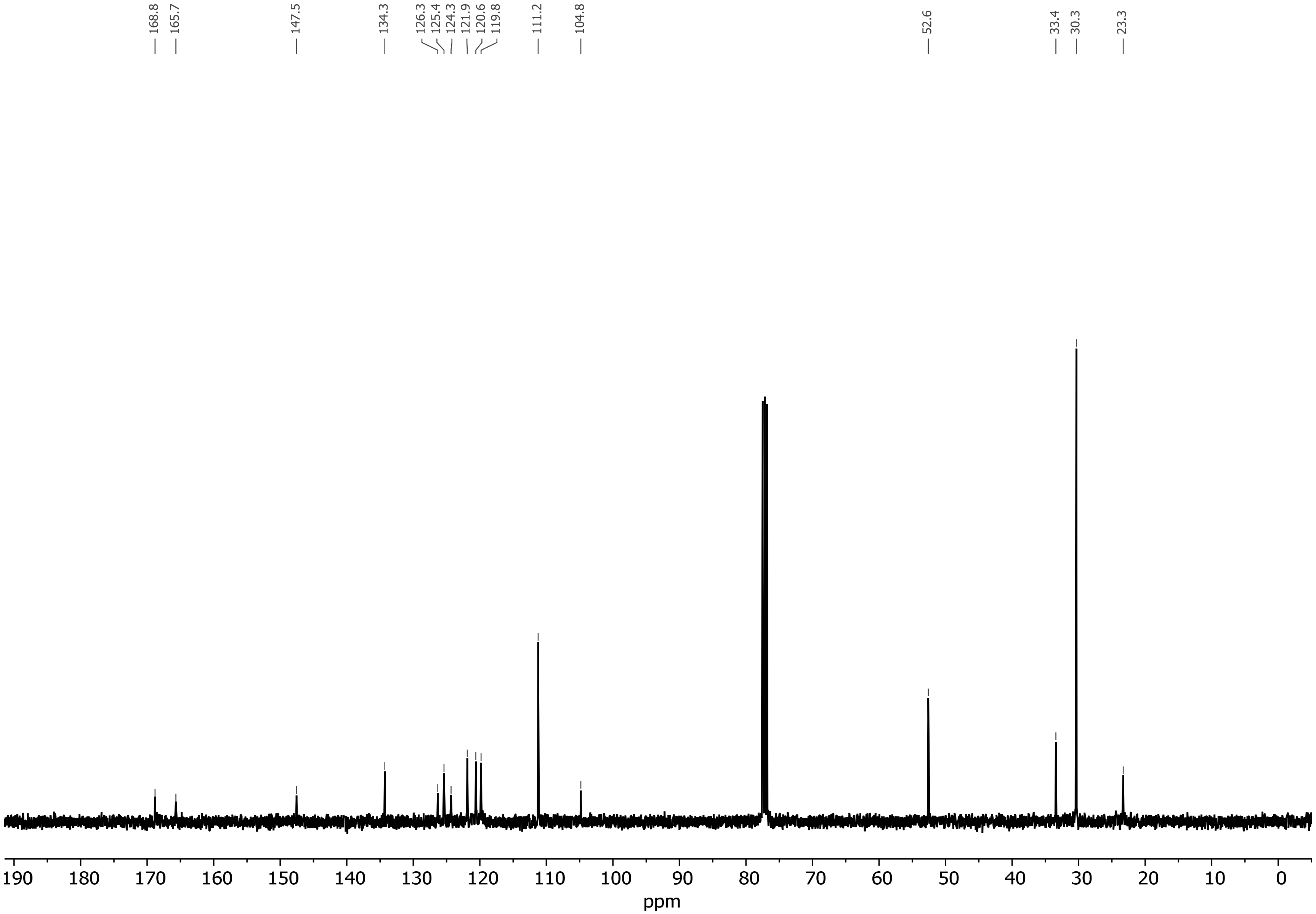

^{1}H NMR (400.16 MHz, acetone-d$_6$) spectrum of 5a

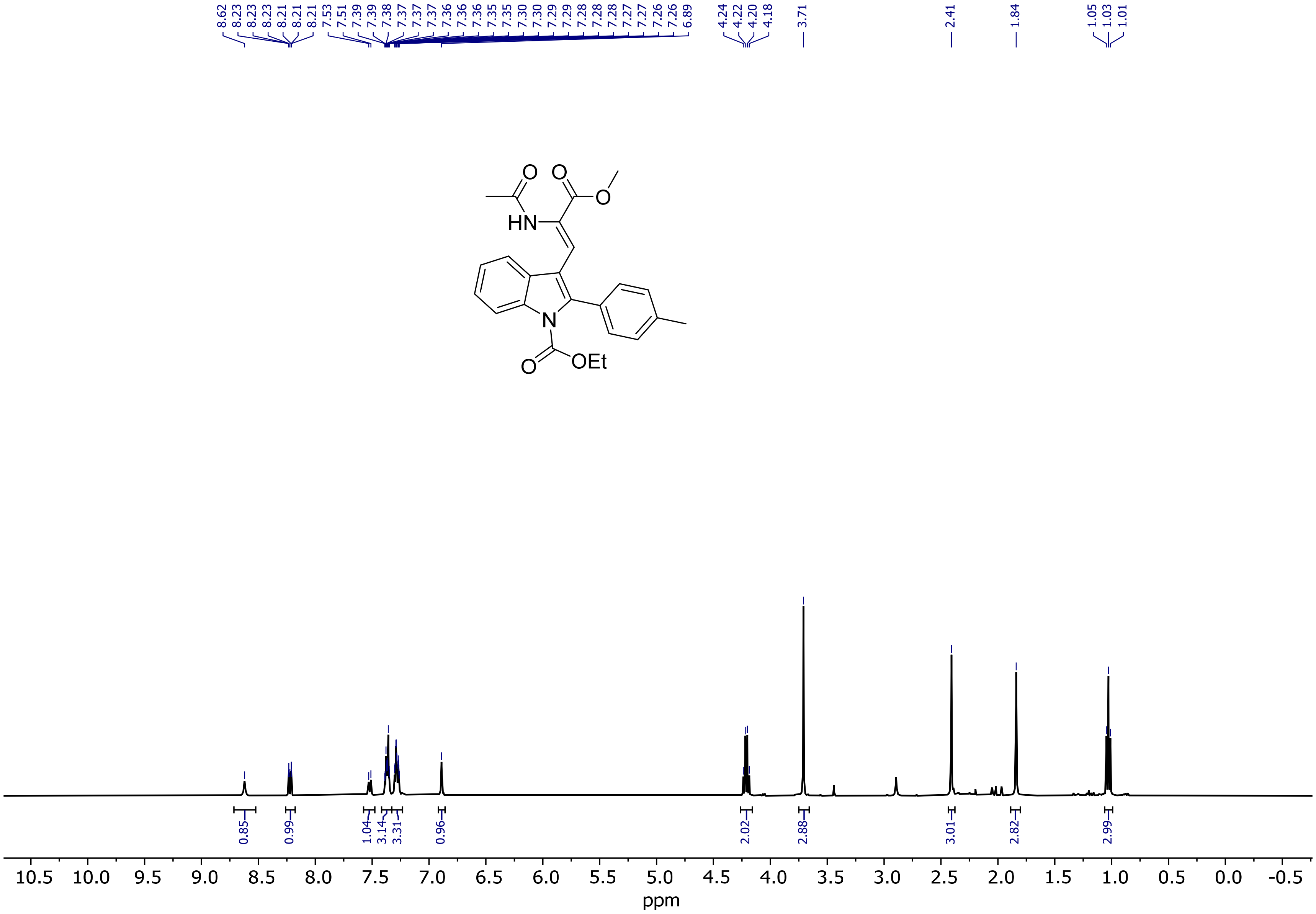

^{13}C {^{1}H} NMR (100.62 MHz, acetone-d_6) spectrum of 5a

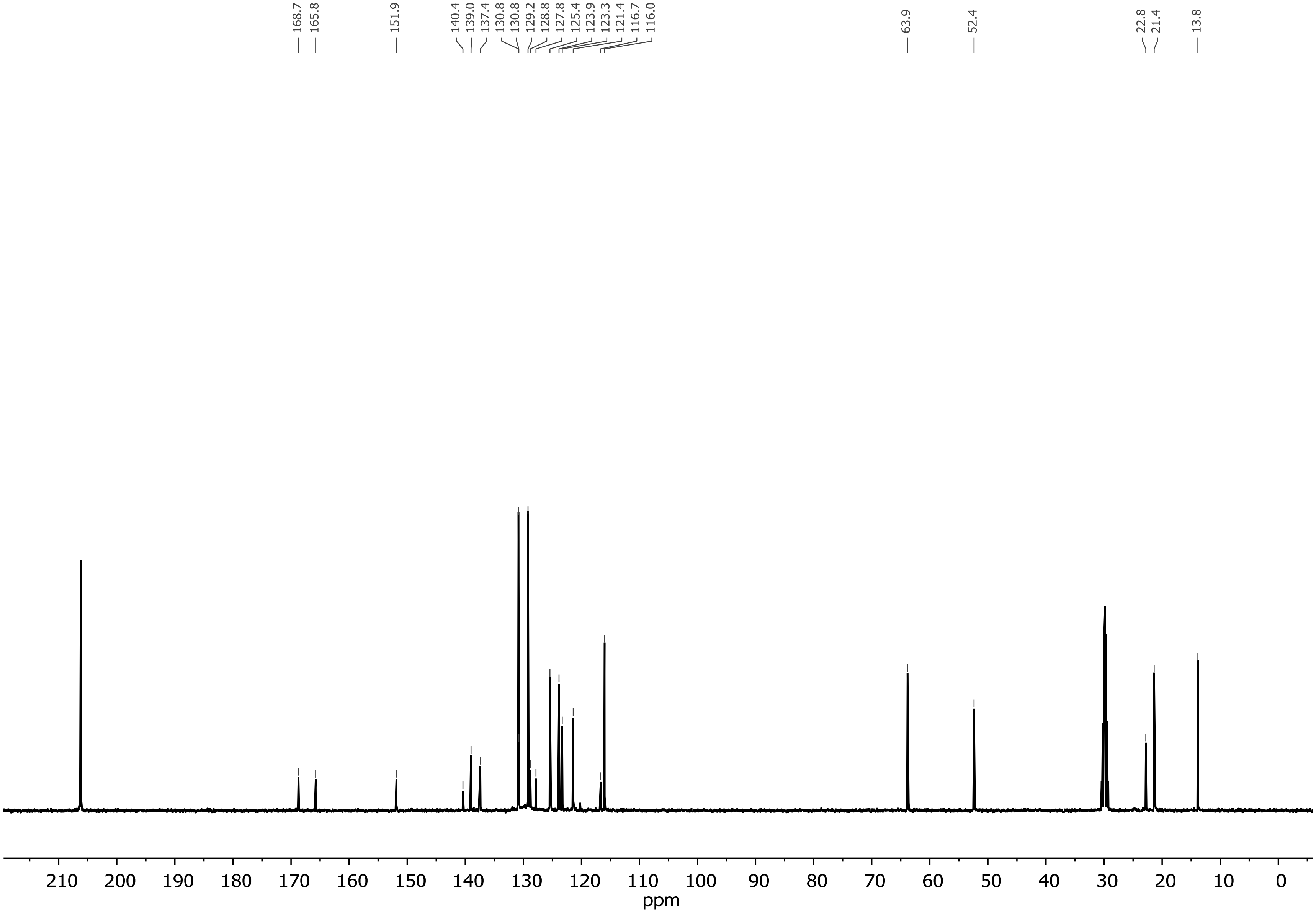

^{1}H NMR (400.16 MHz, CDCl₃) spectrum of 5b

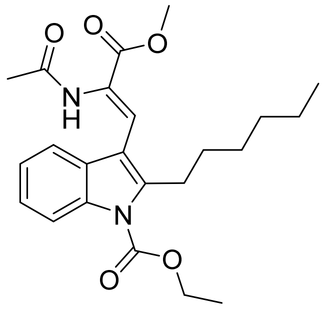

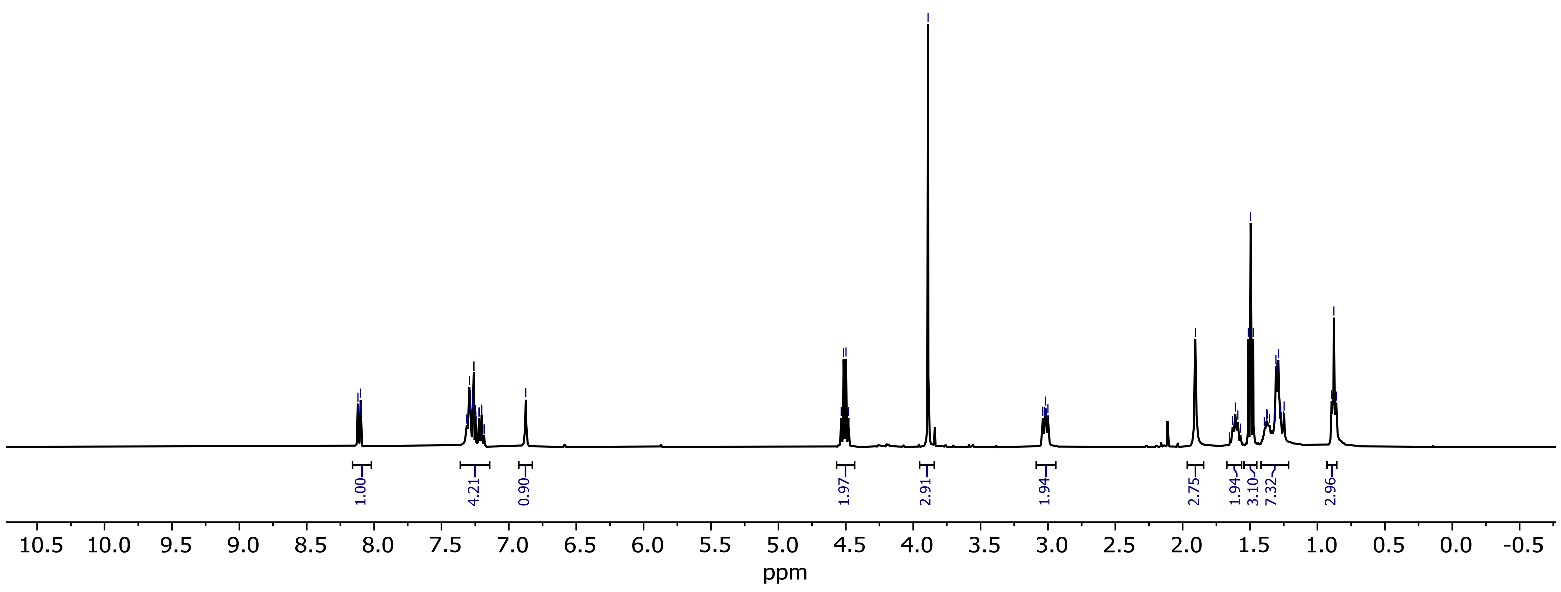

$^{13}C\{^1H\}$ NMR (100.62 MHz, $CDCl_3$) spectrum of 5b

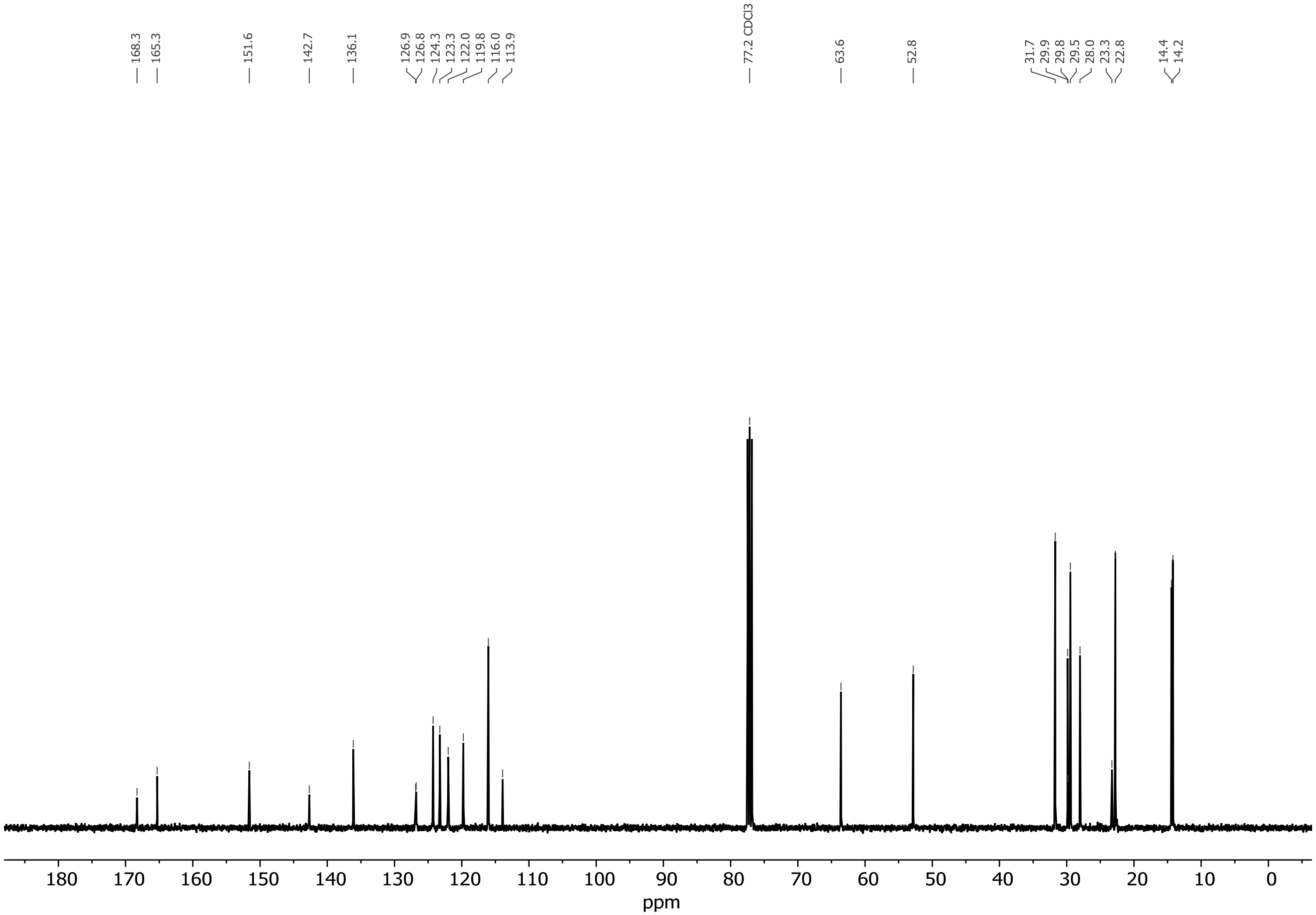

^{1}H NMR (400.16 MHz, $CDCl_3$) spectrum of 4d

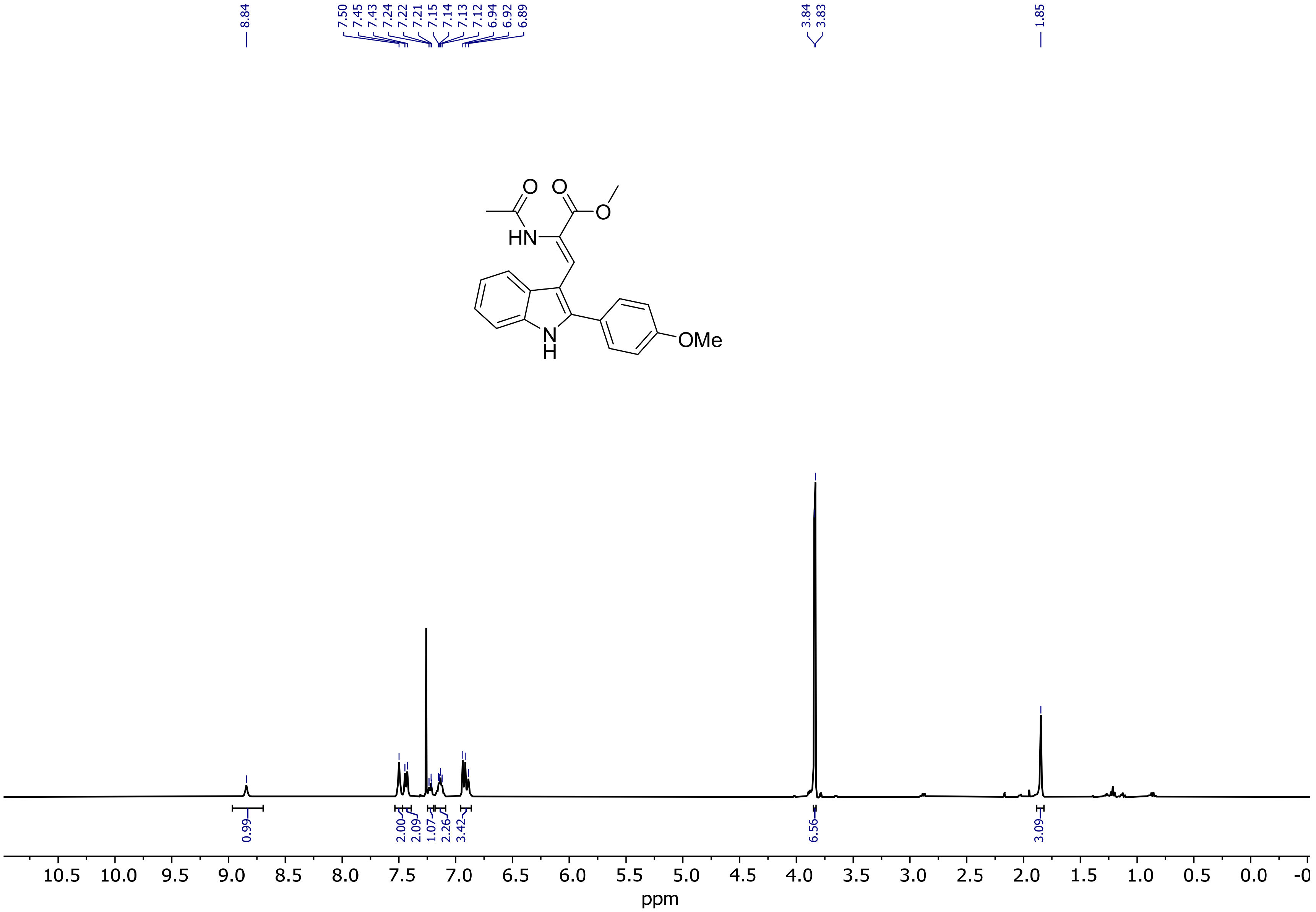

$^{13}C\{^1H\}$ NMR (100.62 MHz, $CDCl_3$) spectrum of 4d

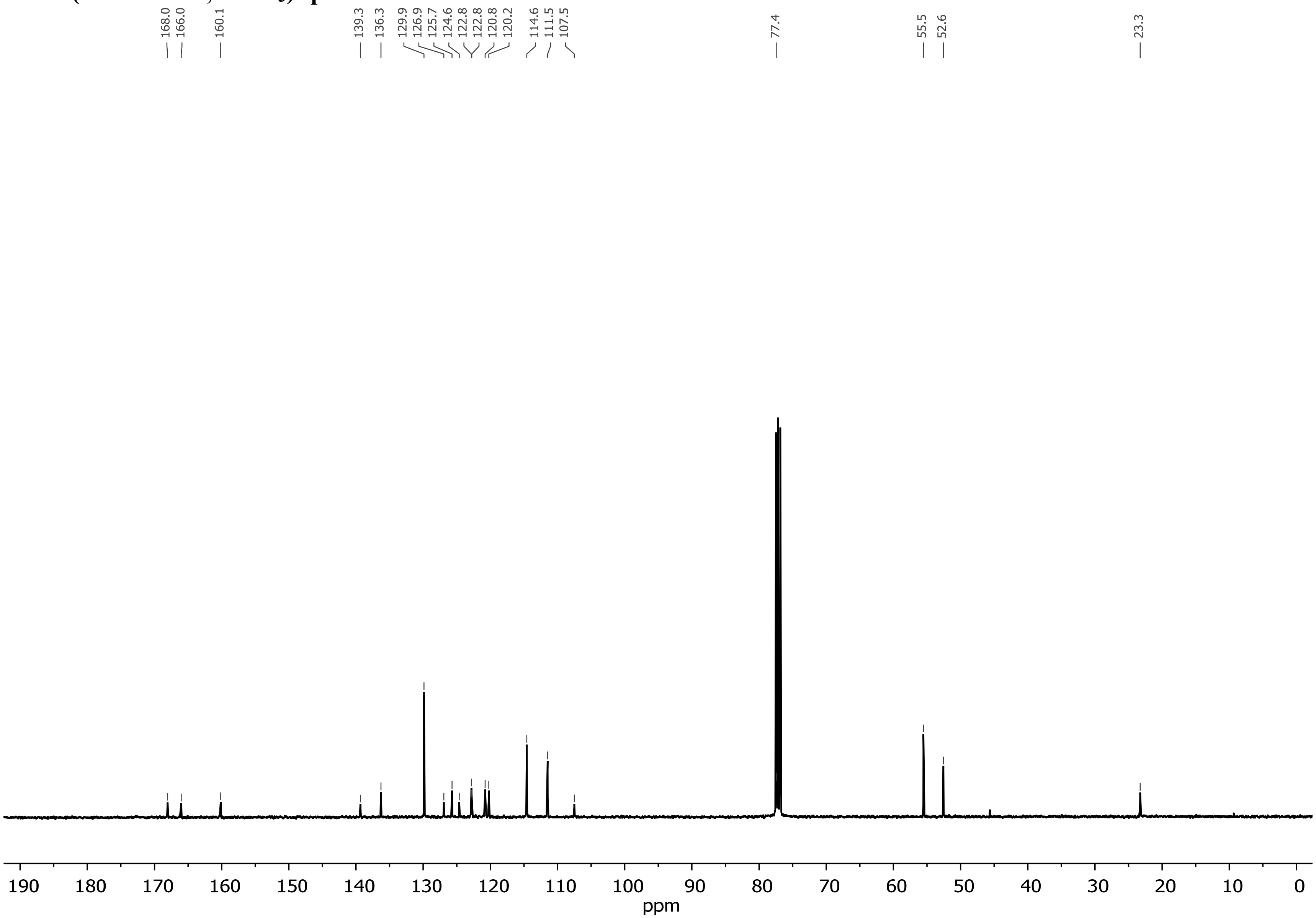

^{1}H NMR (400.16 MHz, DMSO-d$_6$) spectrum of 4e

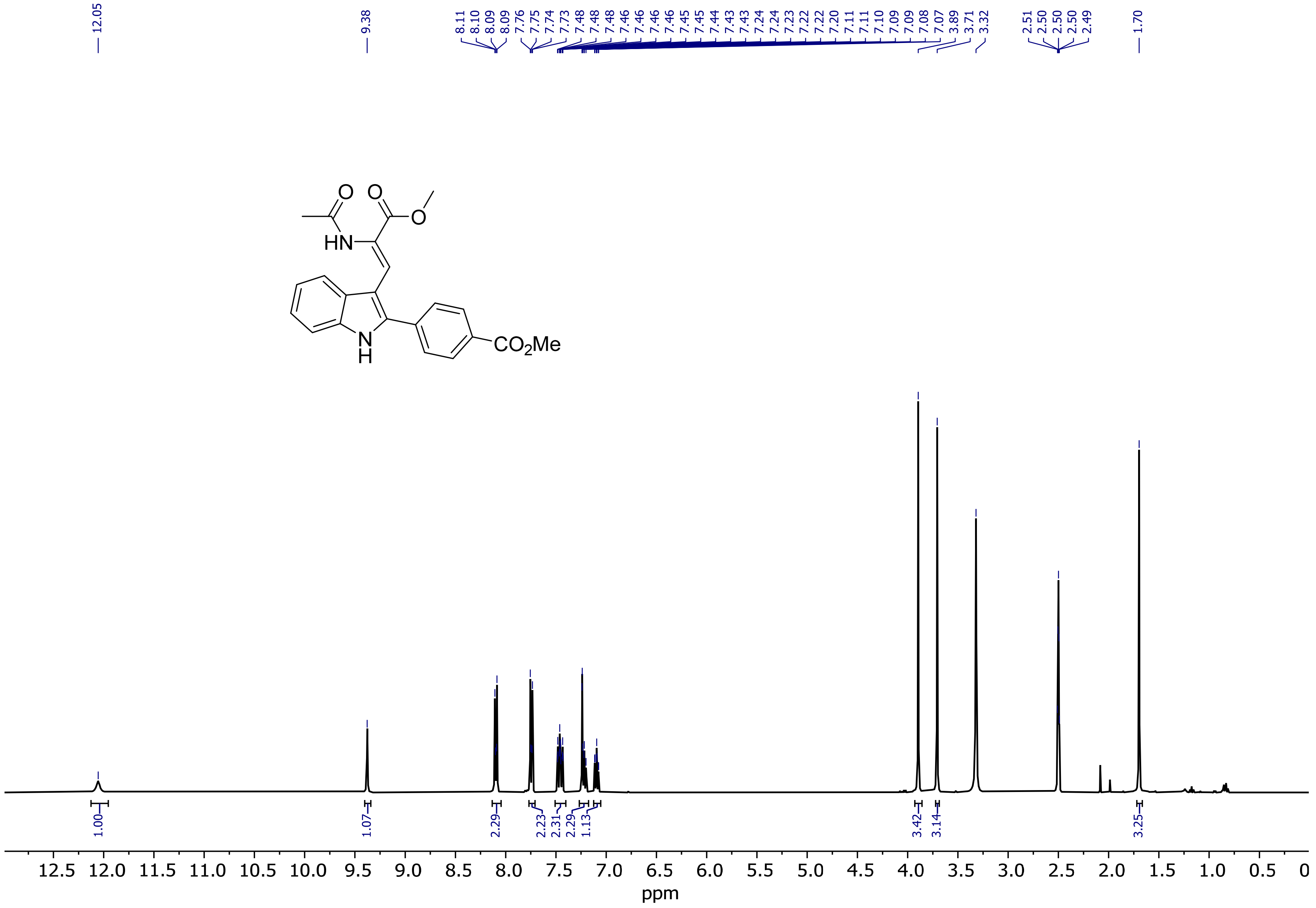

^{13}C {^{1}H} NMR (100.62 MHz, DMSO-d_6) spectrum of 4e

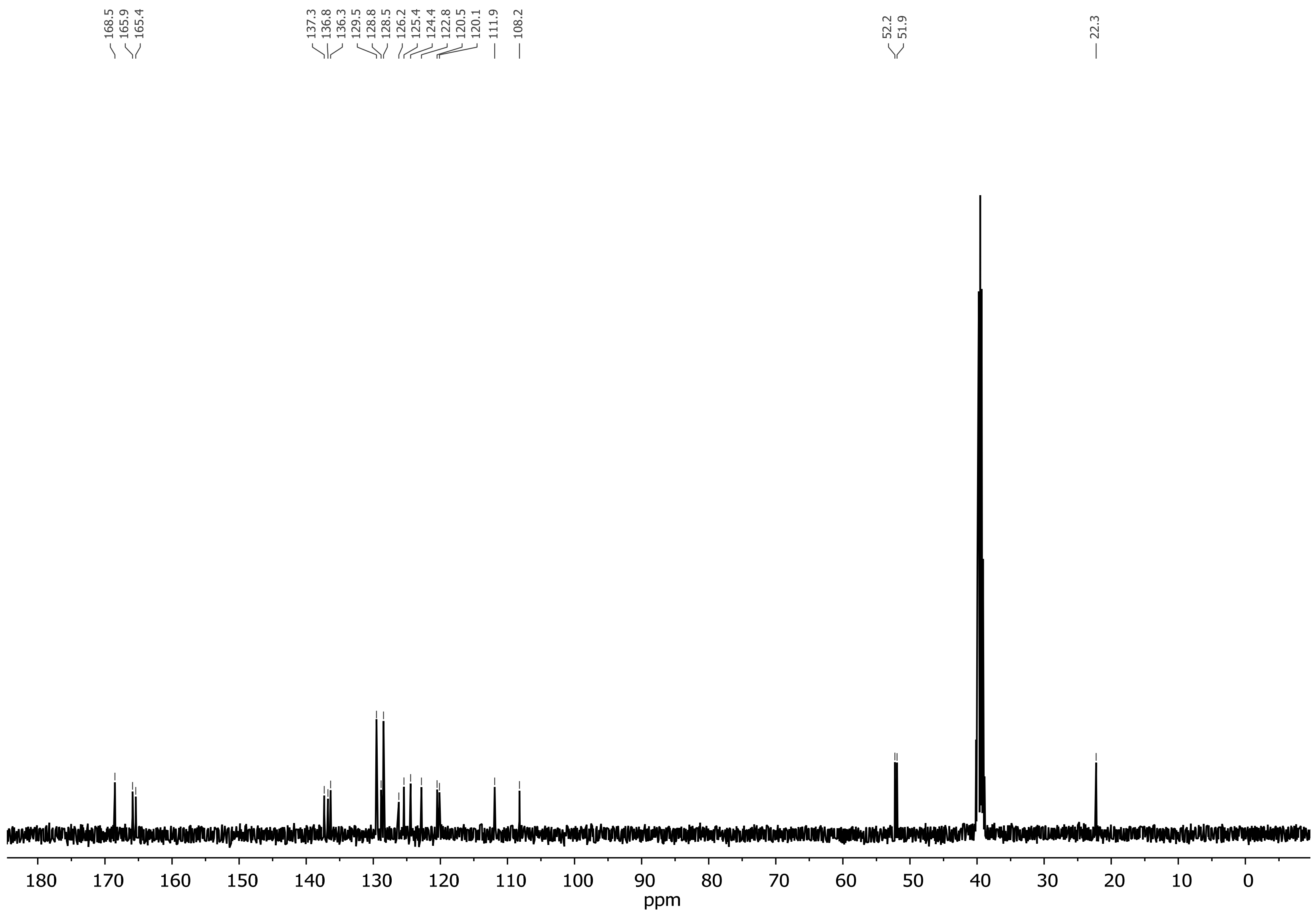

^{1}H NMR (400.16 MHz, MeOD) spectrum of 4f

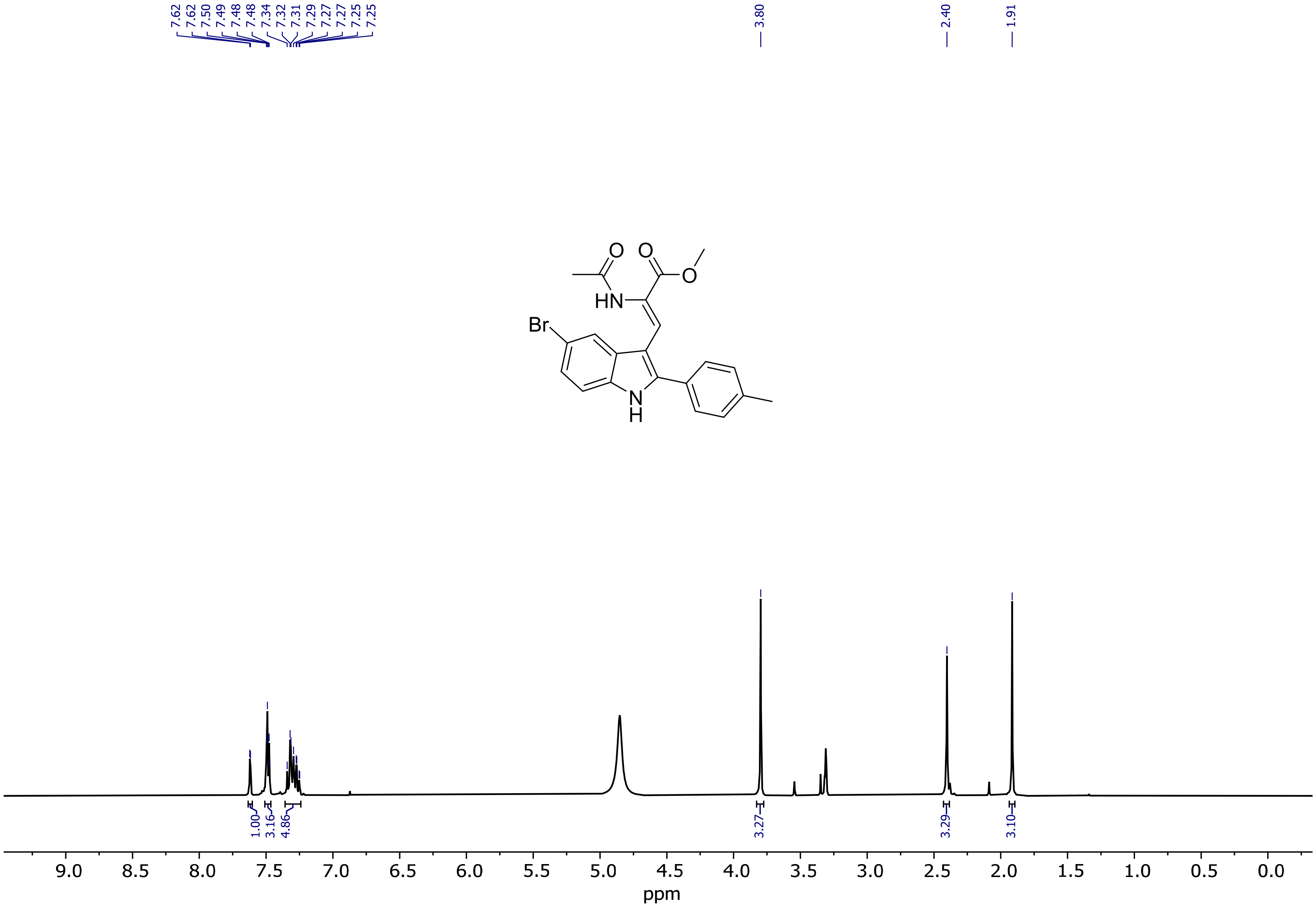

$^{13}C\{^1H\}$ NMR (100.62 MHz, MeOD) spectrum of 4f

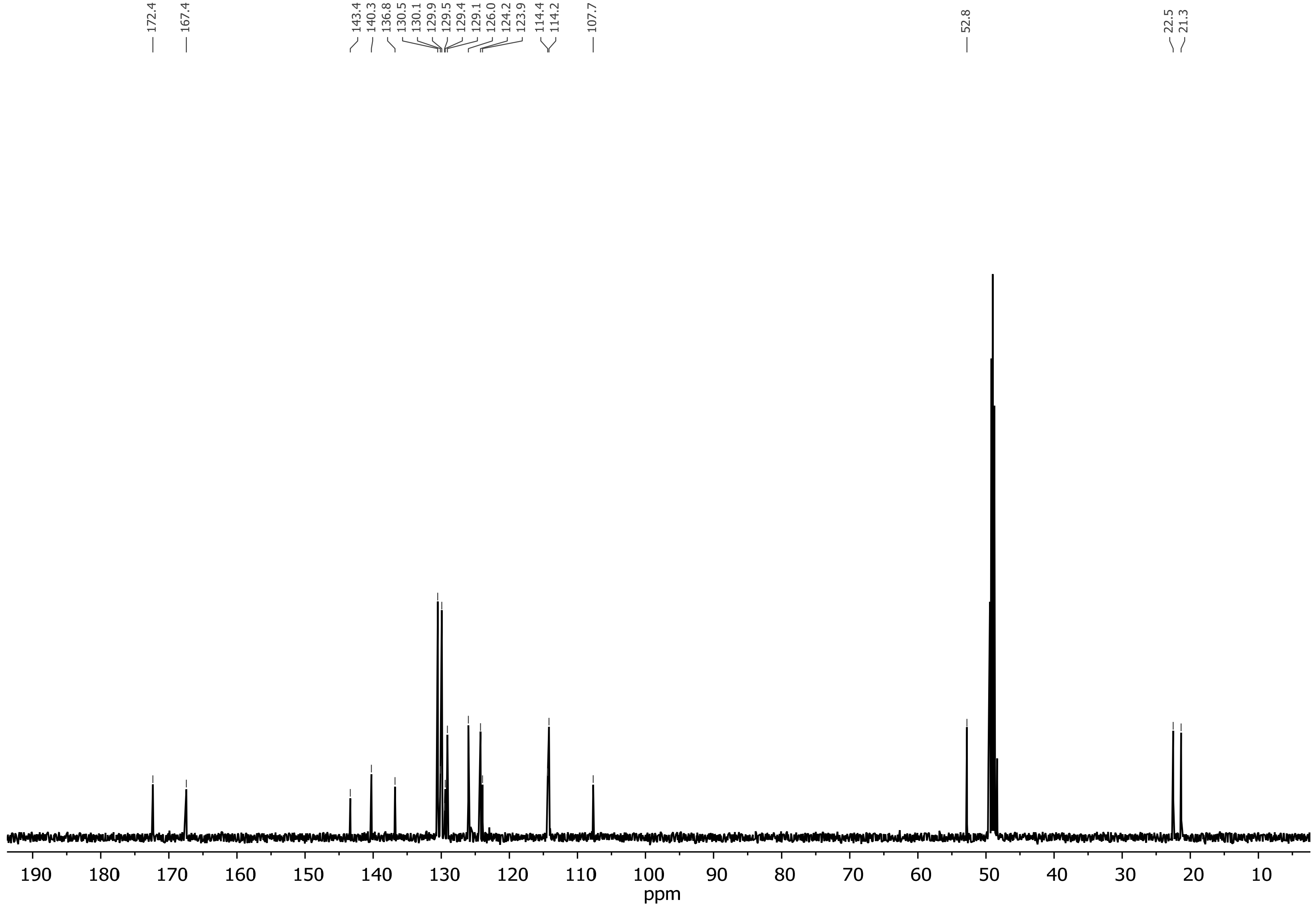

^{1}H NMR (400.16 MHz, MeOD) spectrum of 4g

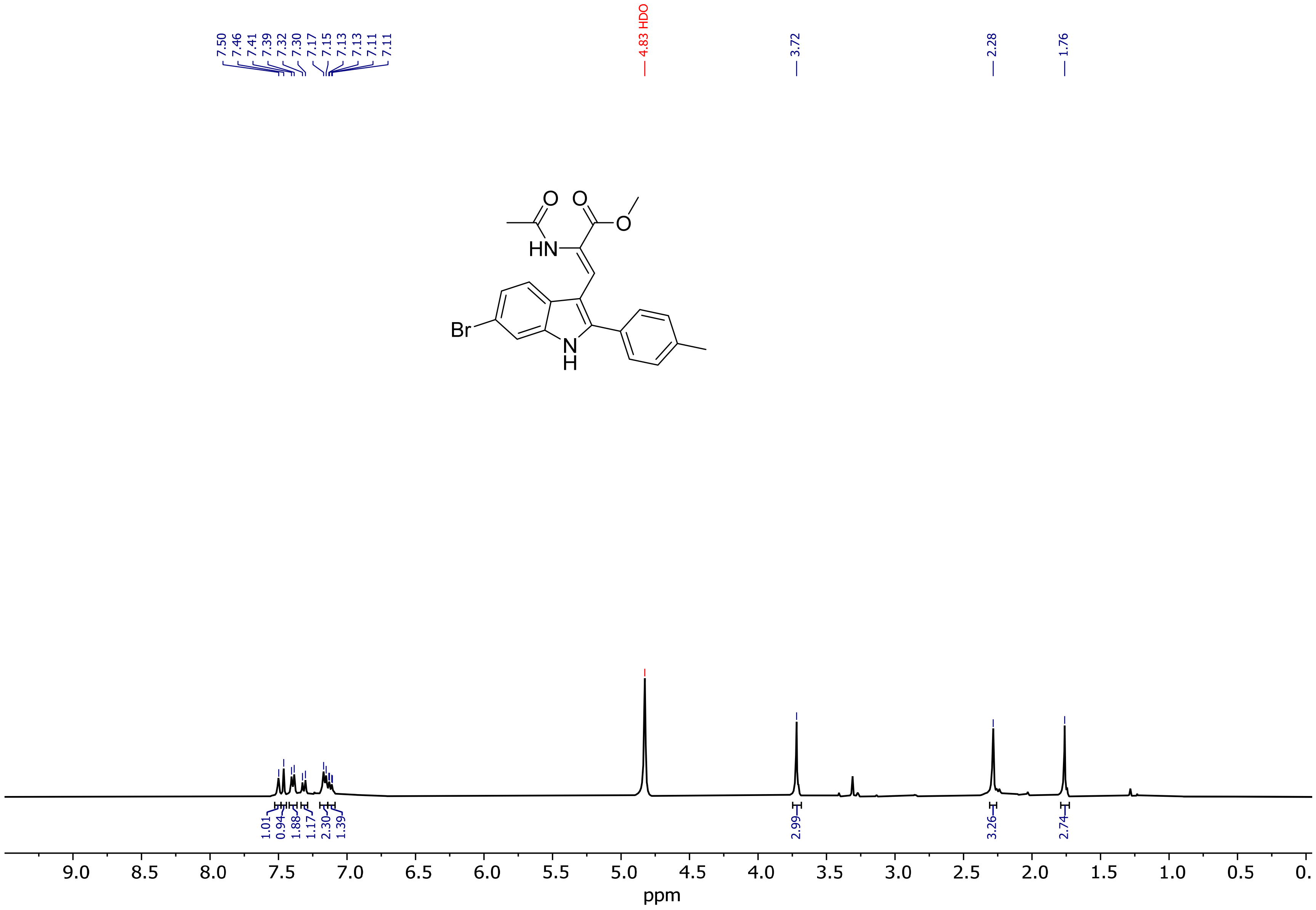

^{13}C {^{1}H} NMR (100.62 MHz, MeOD) spectrum of 4g

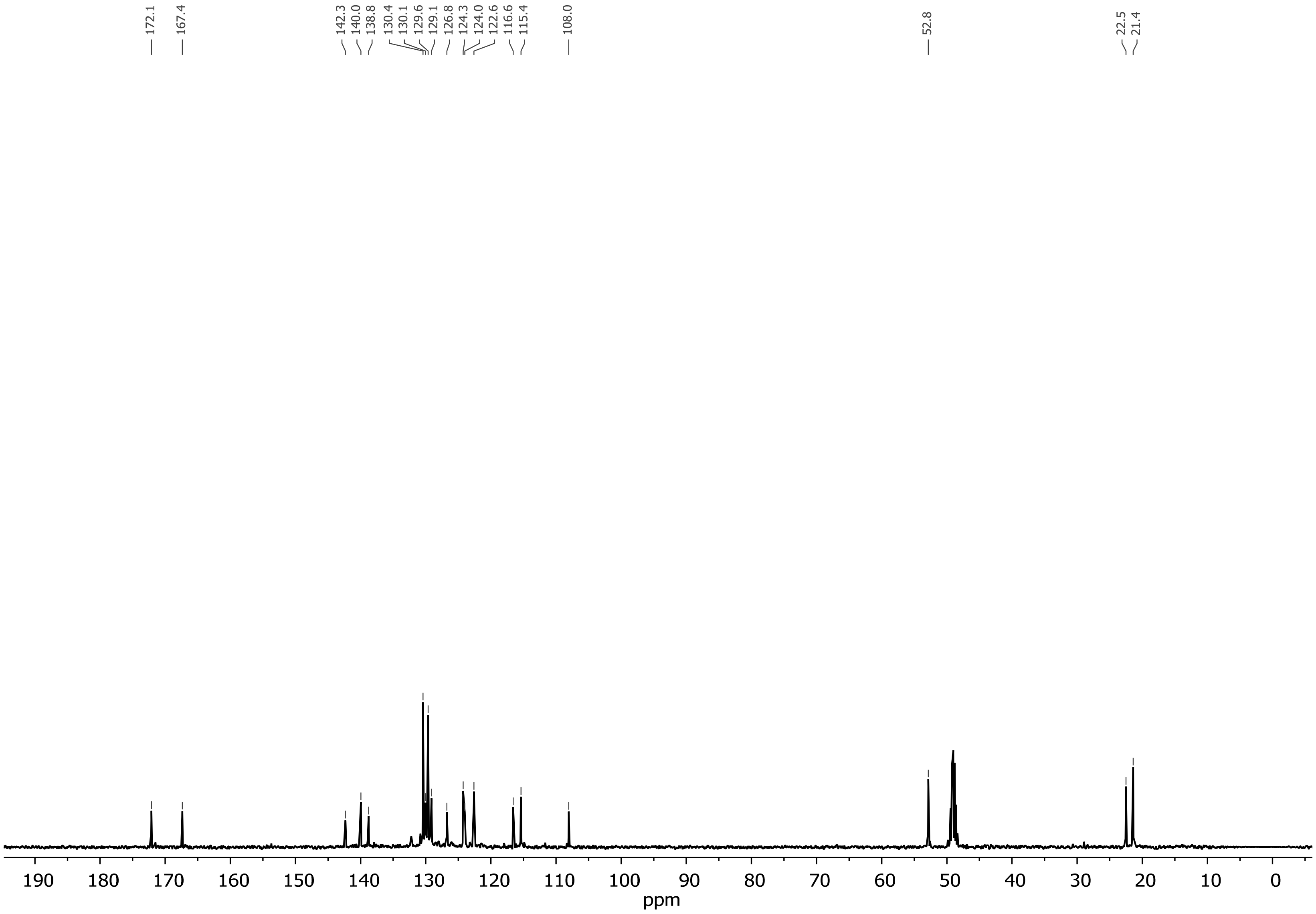

^{1}H NMR (400.16 MHz, DMSO-d$_6$) spectrum of 4h

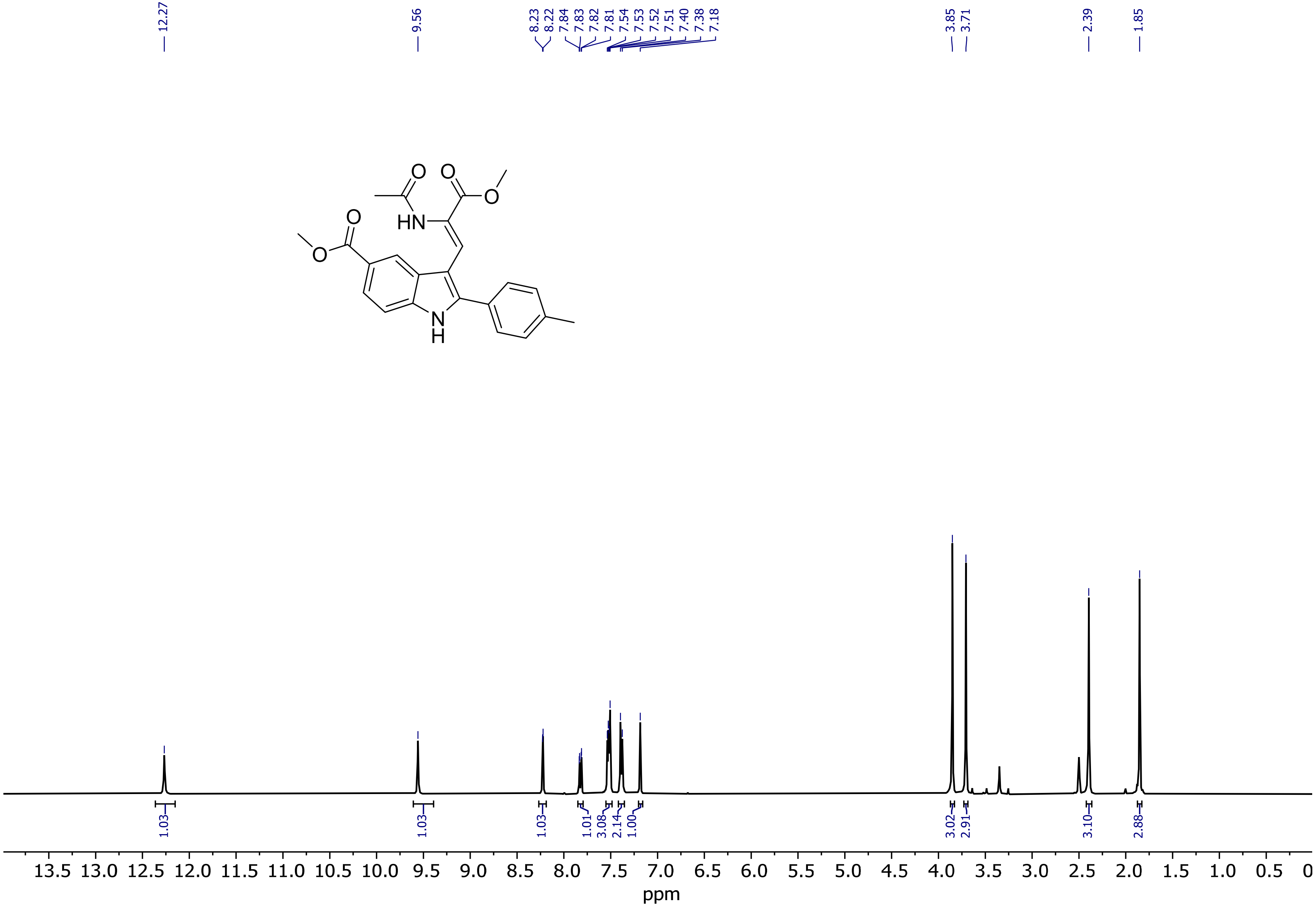

$^{13}C\{^{1}H\}$ NMR (100.62 MHz, DMSO-d$_6$) spectrum of 4h

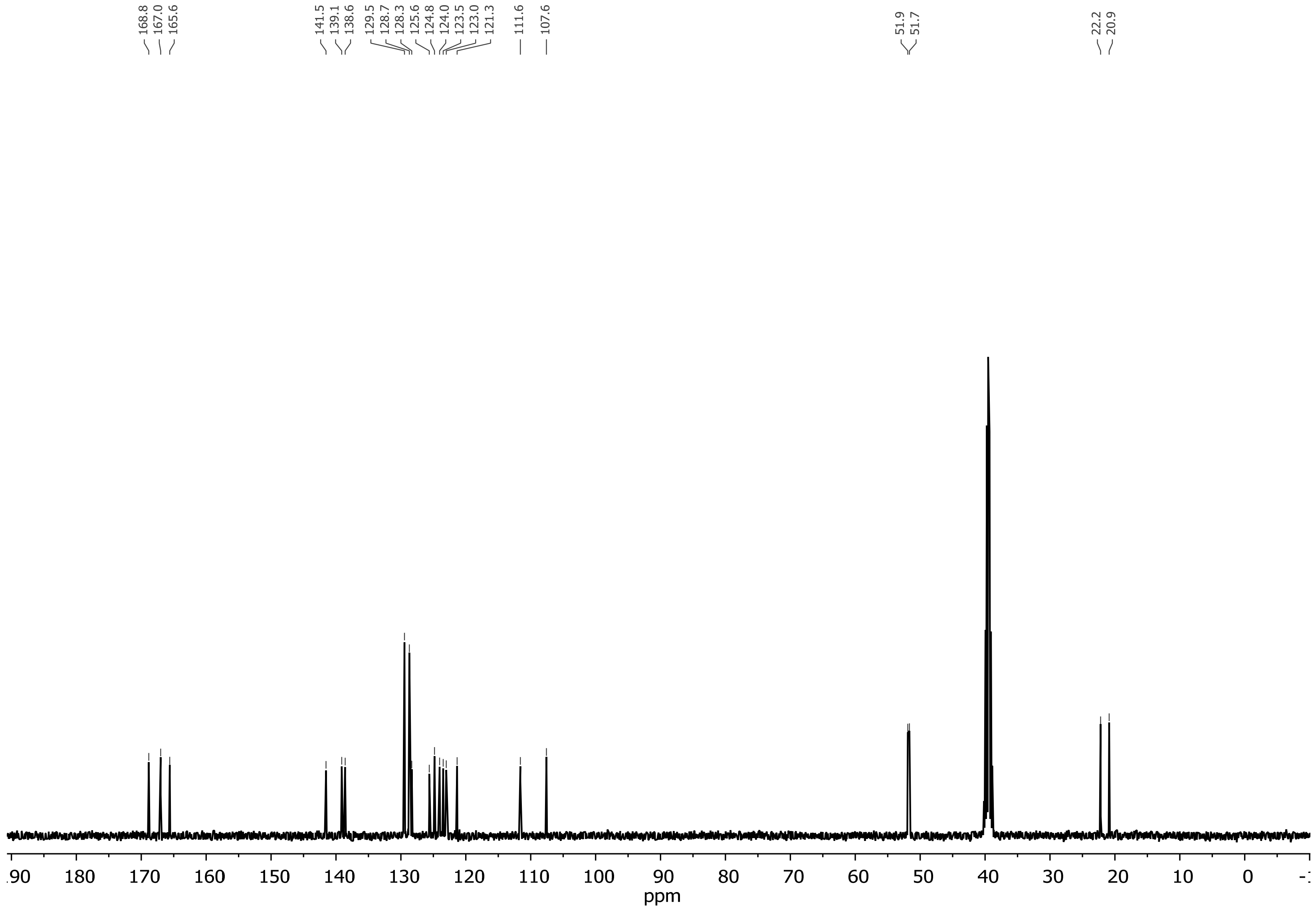

^{1}H NMR (400.16 MHz, MeOD) spectrum of 4i

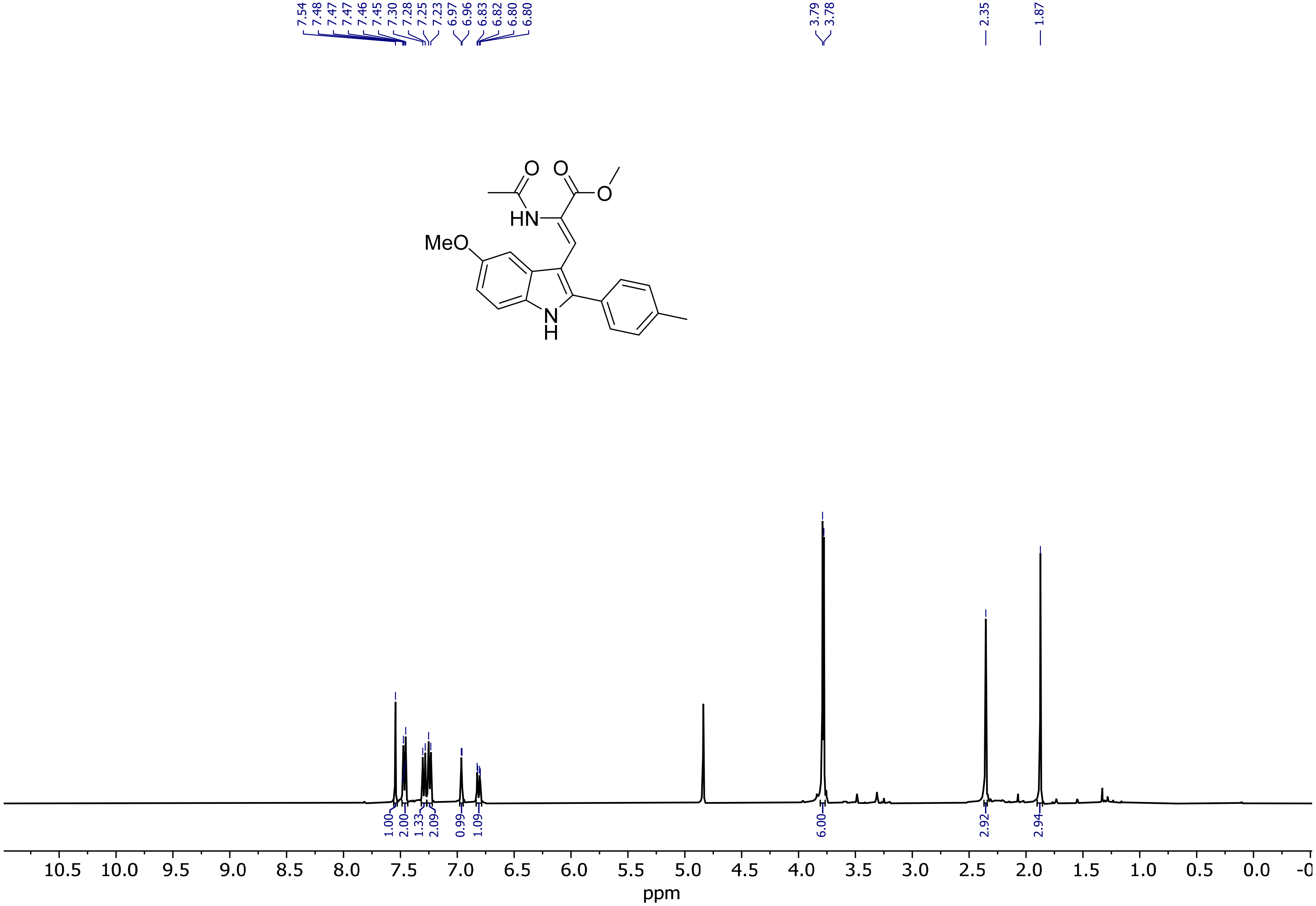

^{13}C {^{1}H} NMR (100.62 MHz, MeOD) spectrum of 4i

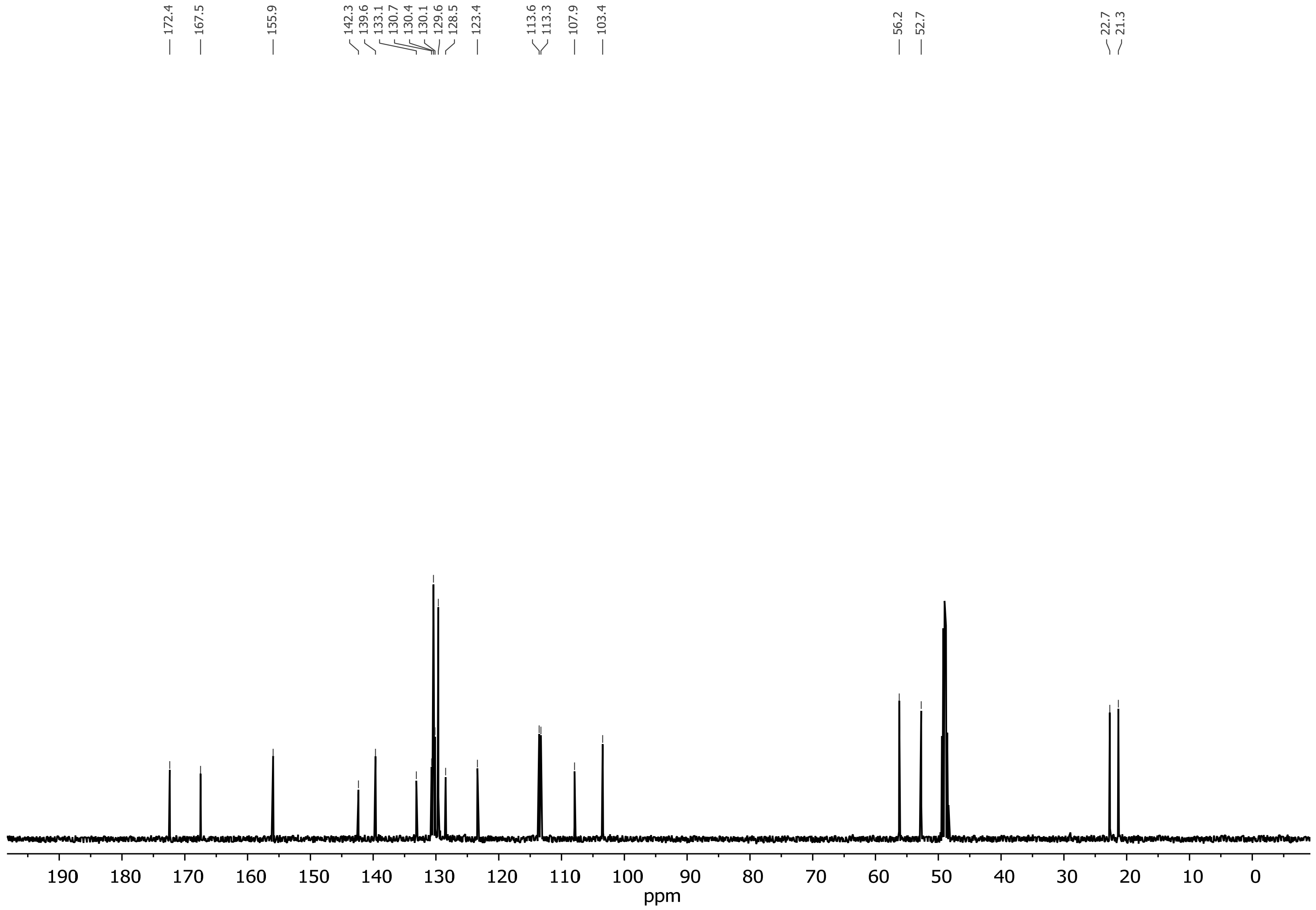

^{1}H NMR (400.16 MHz, MeOD) spectrum of 4j

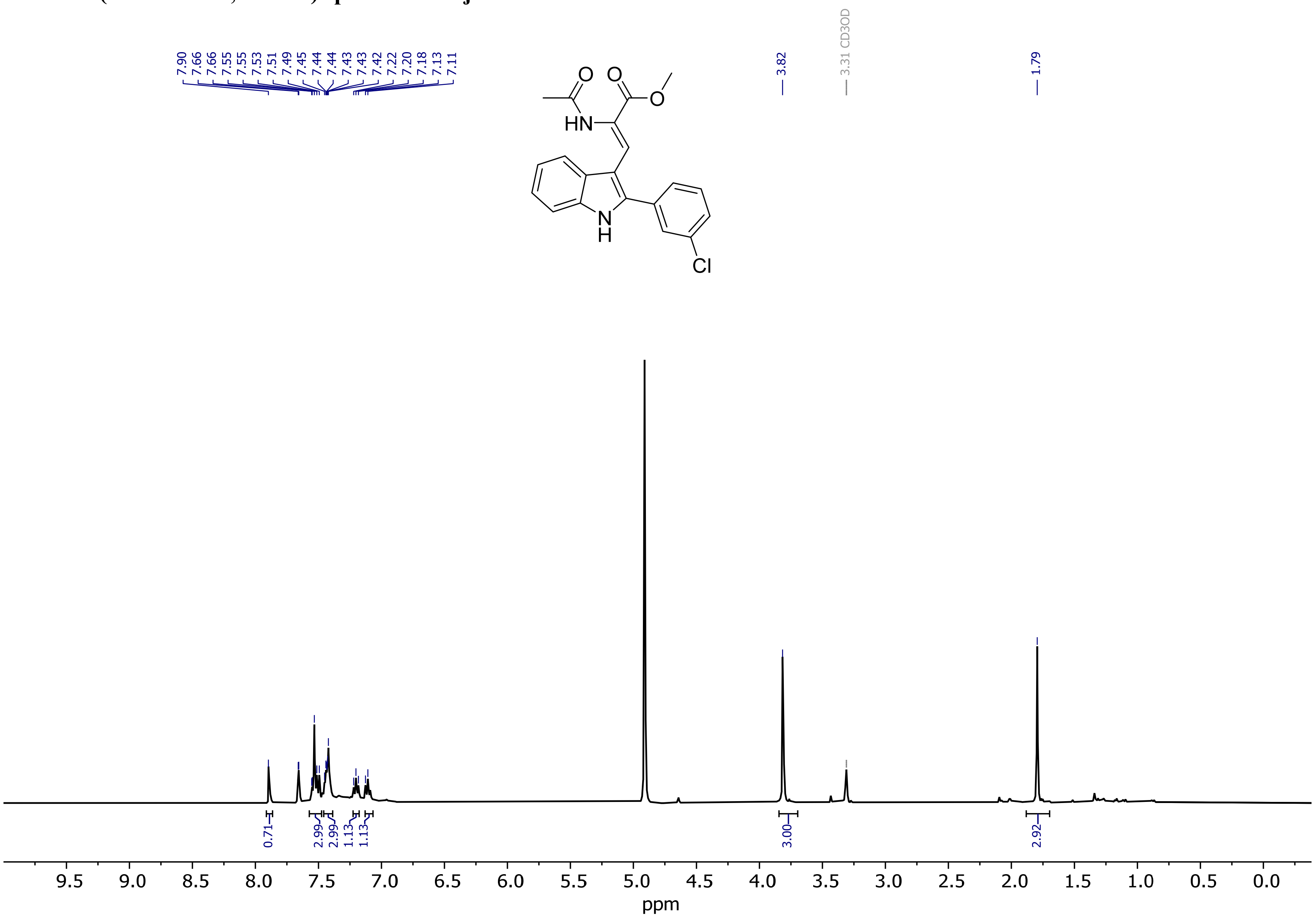

^{13}C {^{1}H} NMR (100.62 MHz, MeOD) spectrum of 4j

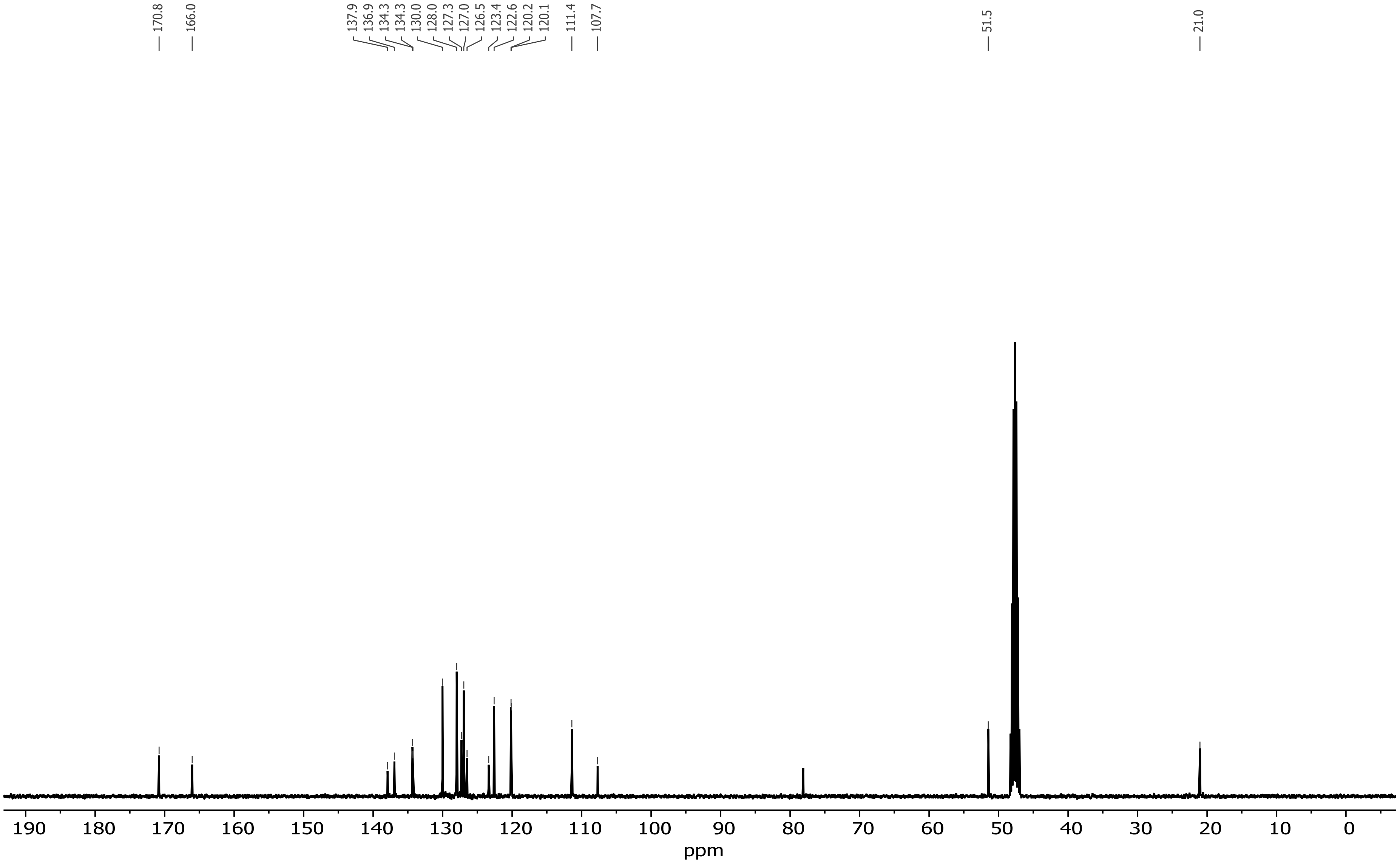

^{1}H NMR (400.16 MHz, MeOD) spectrum of 4k

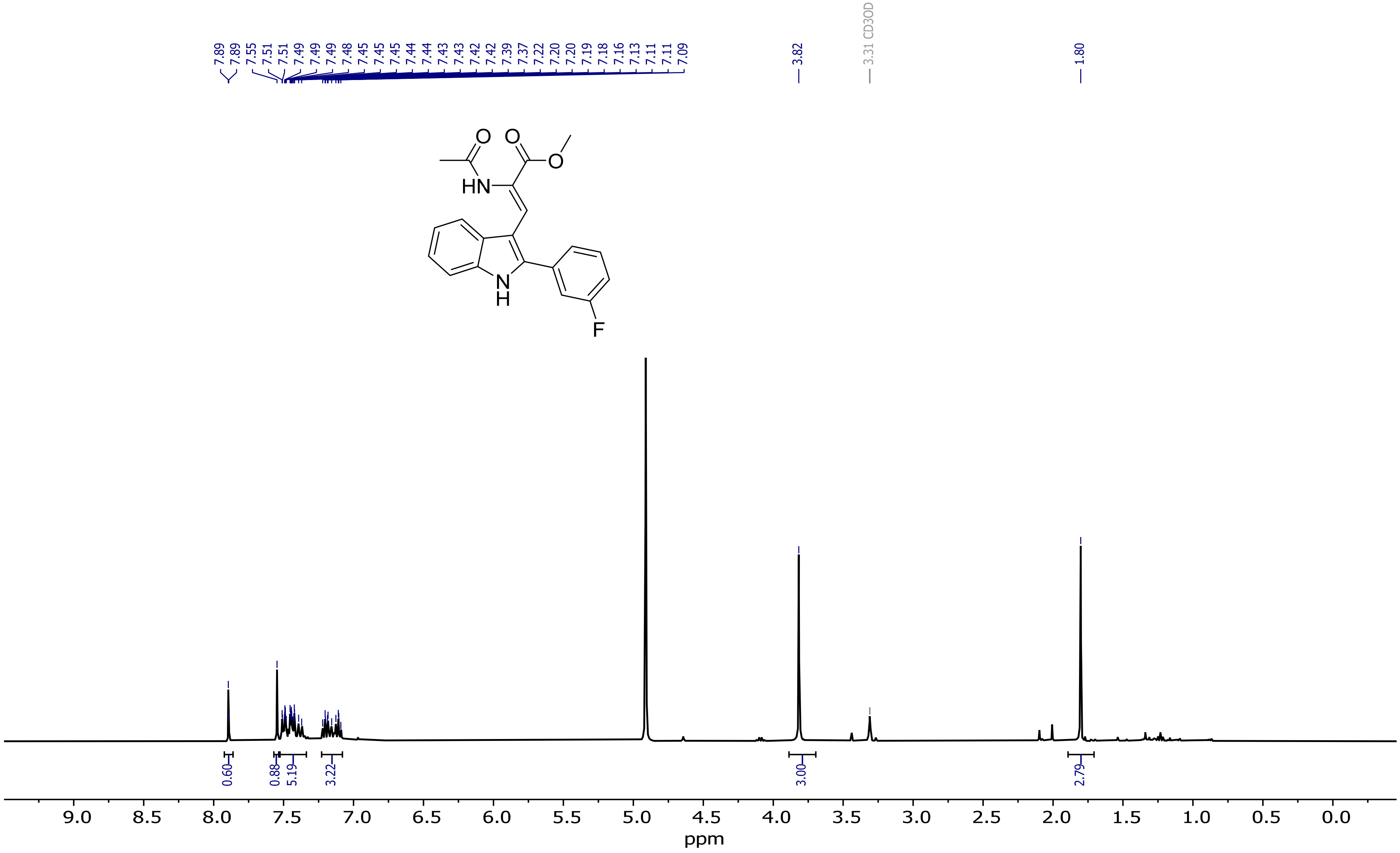

$^{13}C\ \{^{1}H\}$ NMR (100.62 MHz, MeOD) spectrum of 4k

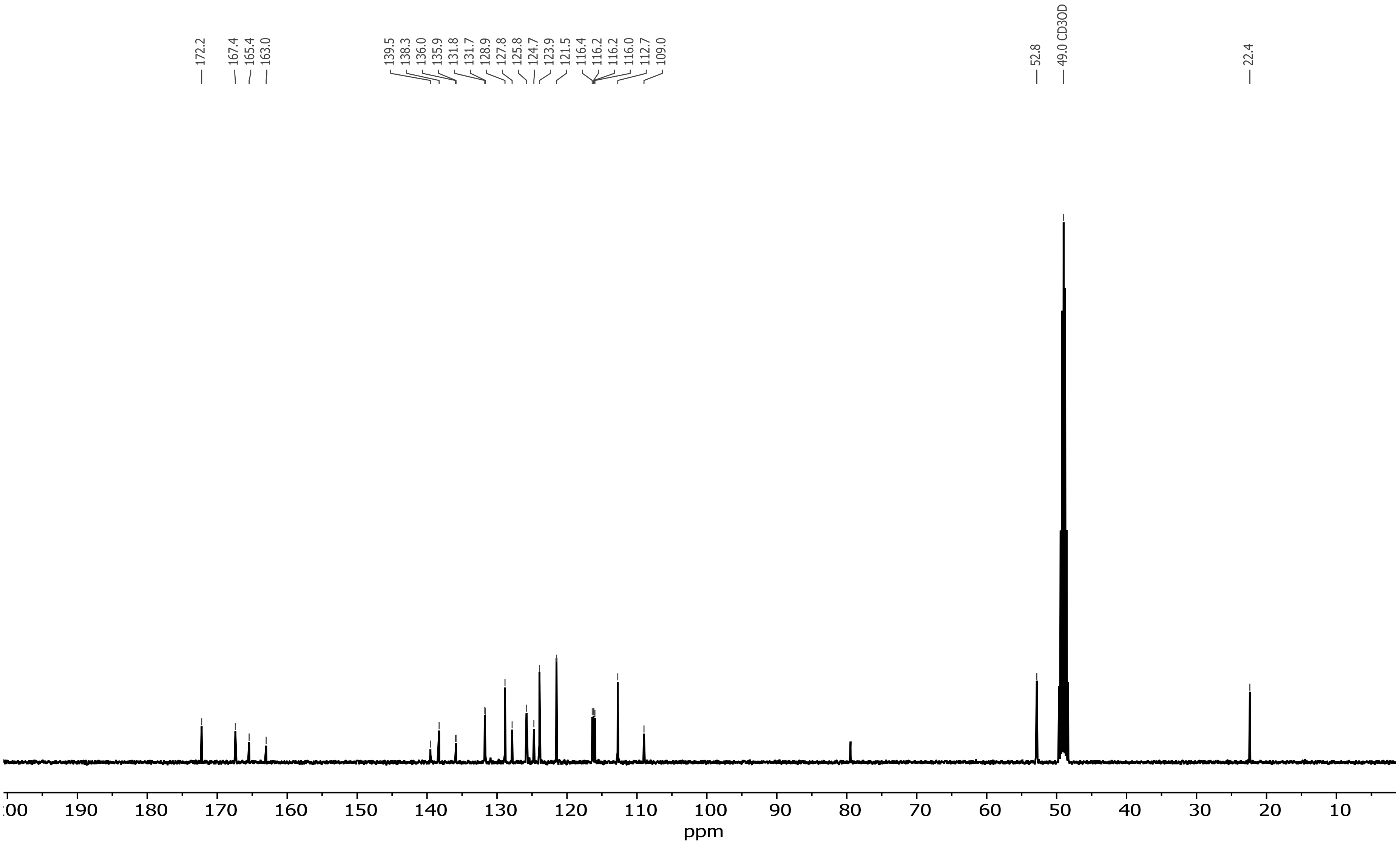

^{1}H NMR (400.16 MHz, DMSO-d6) spectrum of 4l

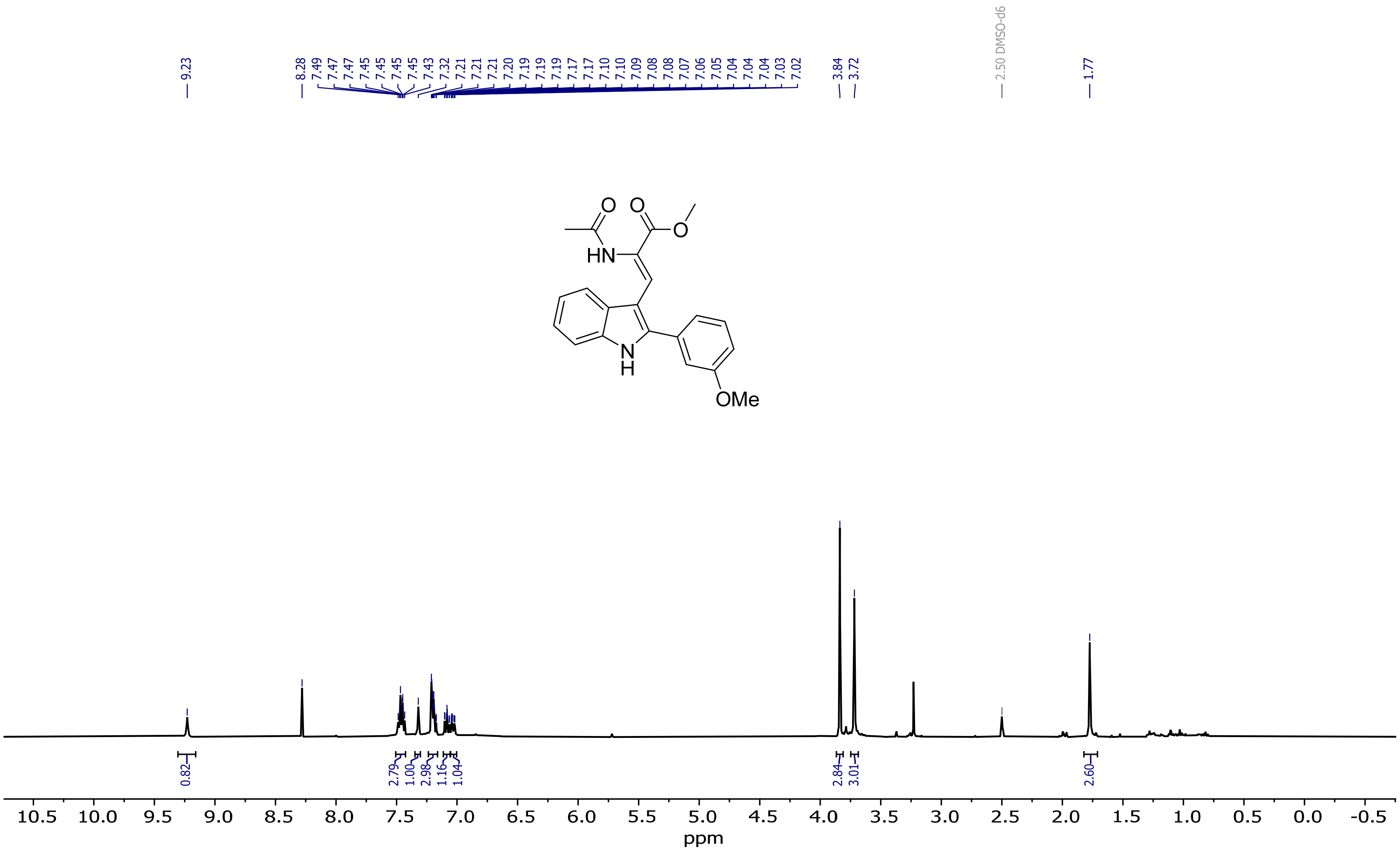

^{13}C {^{1}H} NMR (100.62 MHz, DMSO-d6) spectrum of 4l

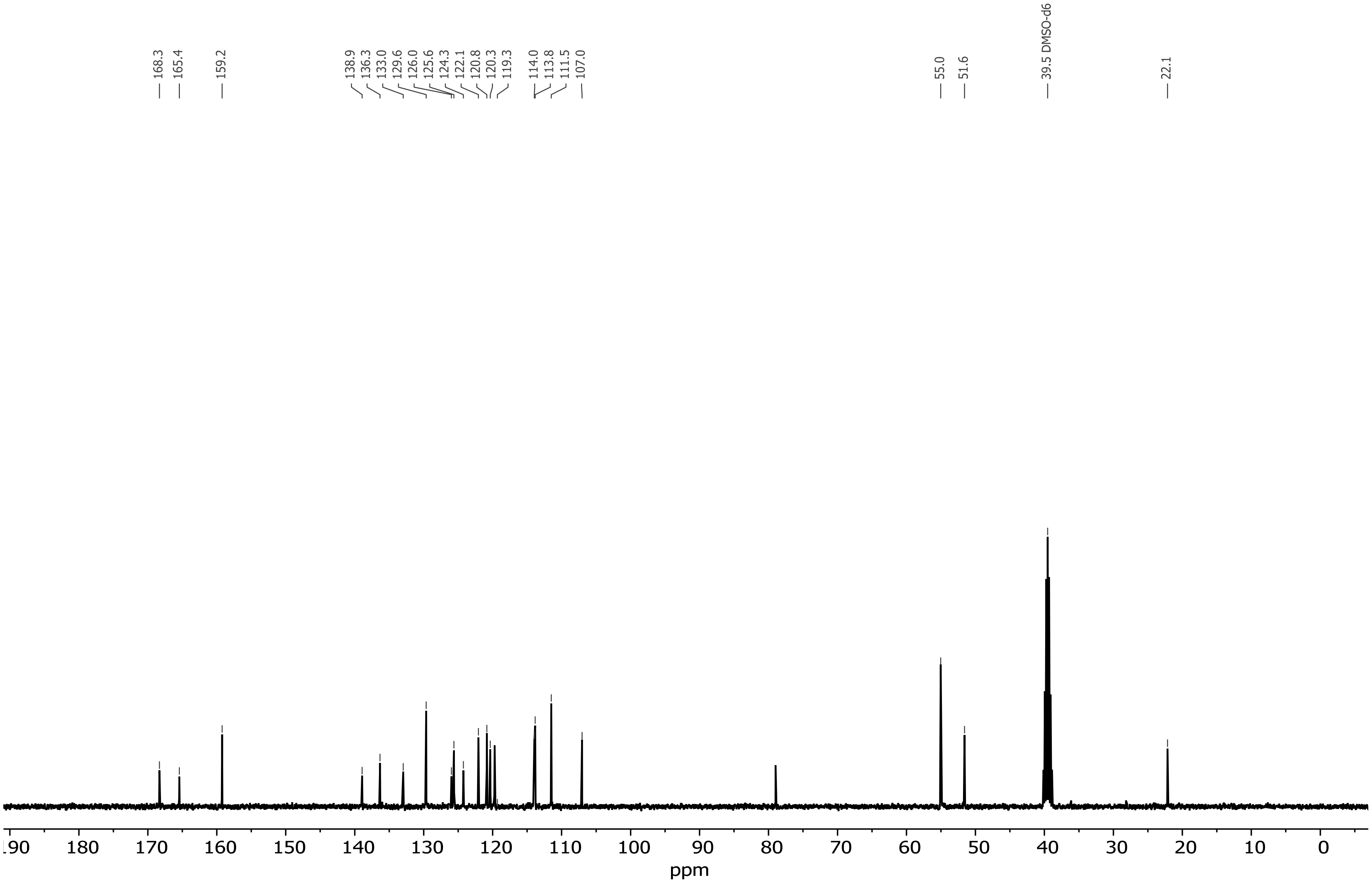

^{1}H NMR (400.16 MHz, DMSO-d6) spectrum of 4m

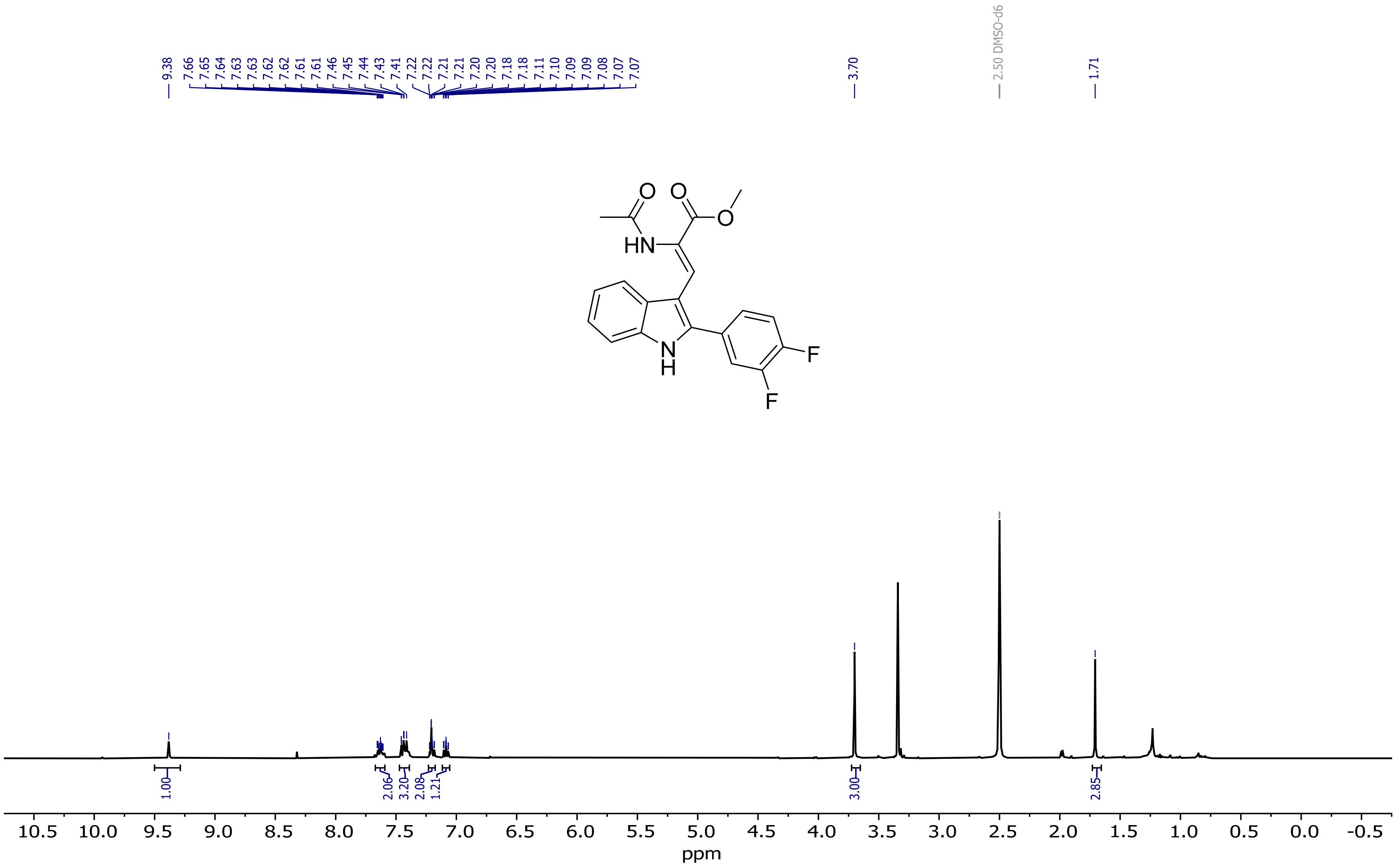

$^{13}C\ \{^{1}H\}$ NMR (100.62 MHz, DMSO-d6) spectrum of 4m

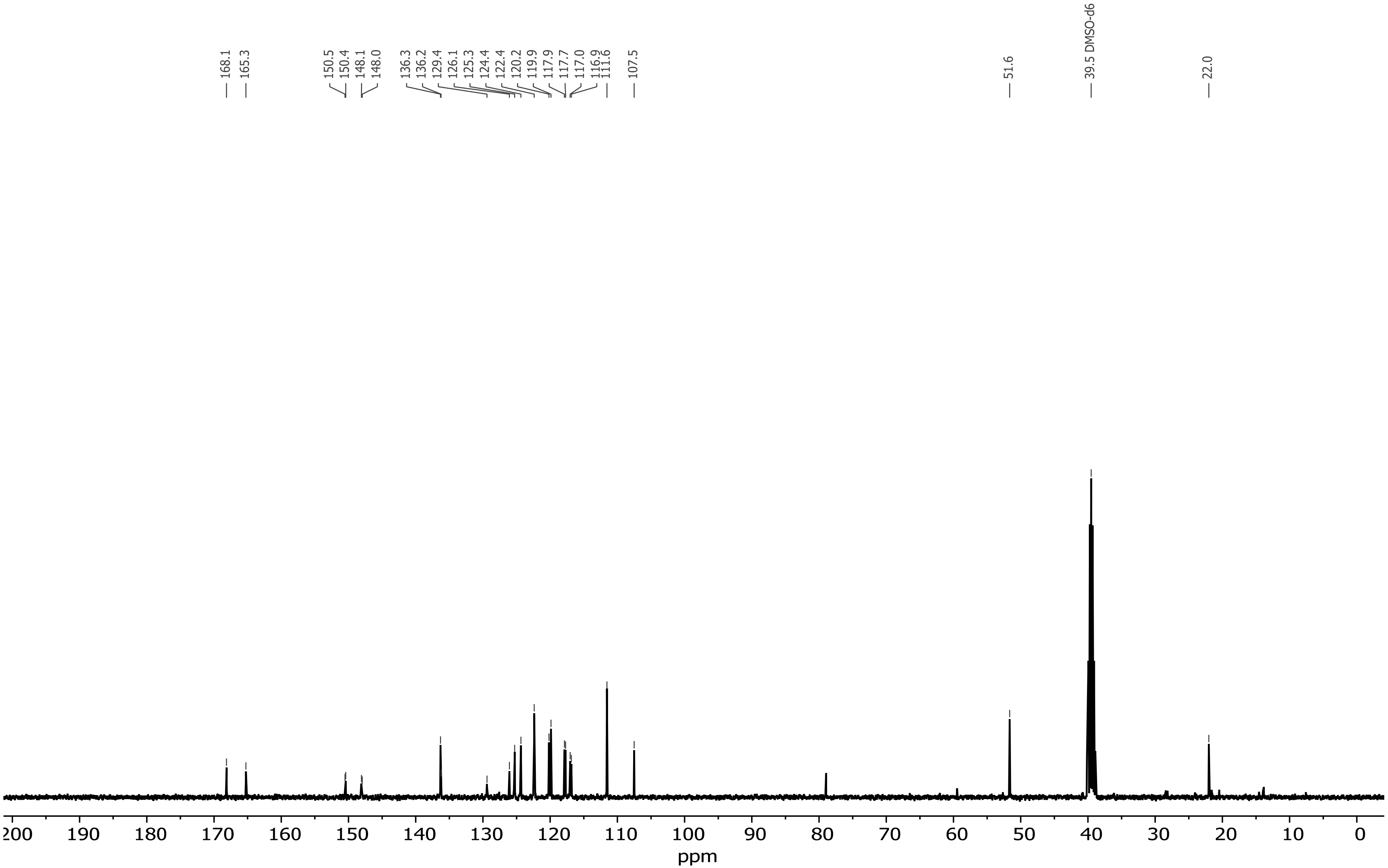

^{1}H NMR (400.16 MHz, DMSO-d6) spectrum of 4n

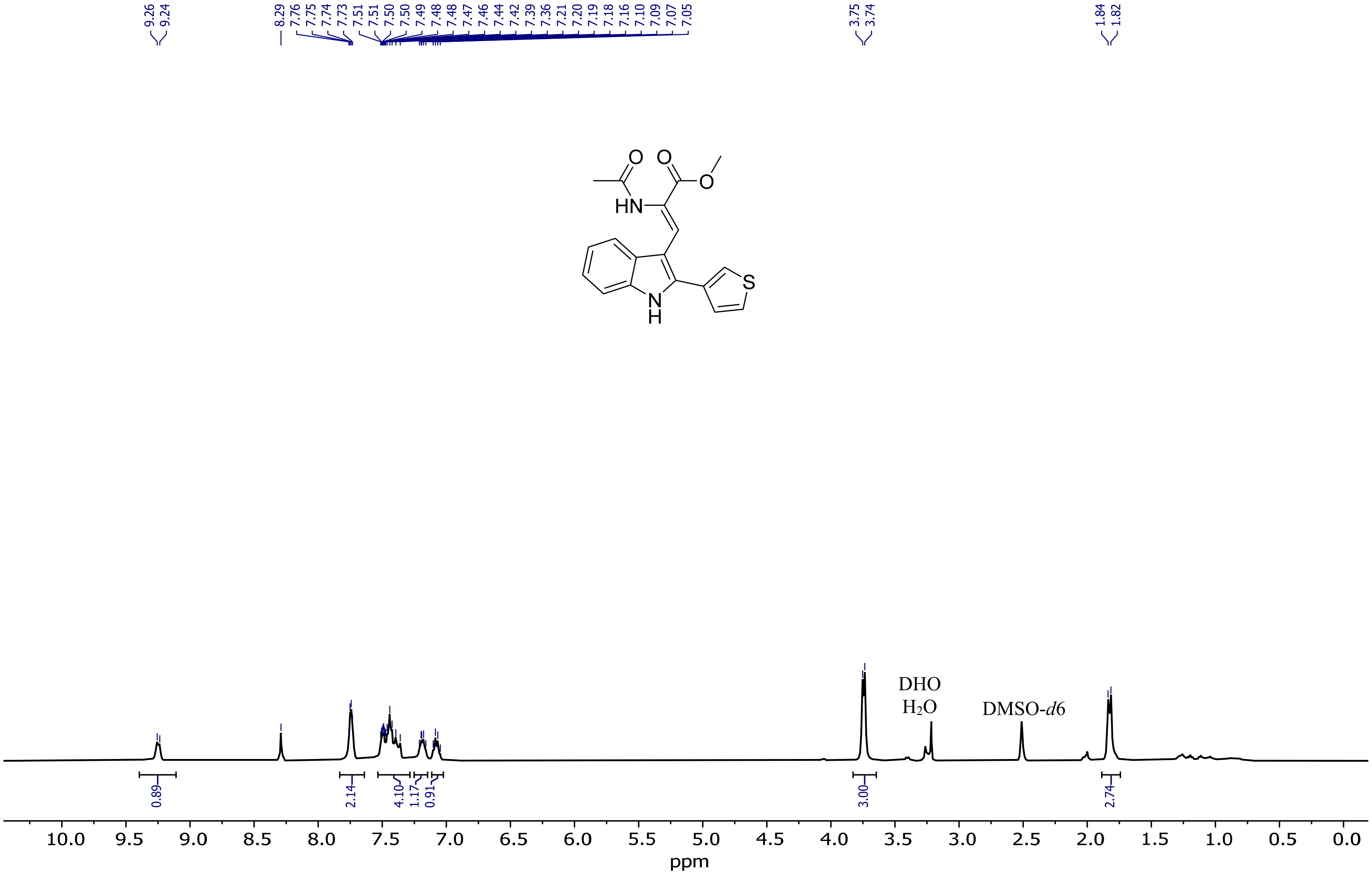

^{13}C {^{1}H} NMR (100.62 MHz, DMSO-d6) spectrum of 4n

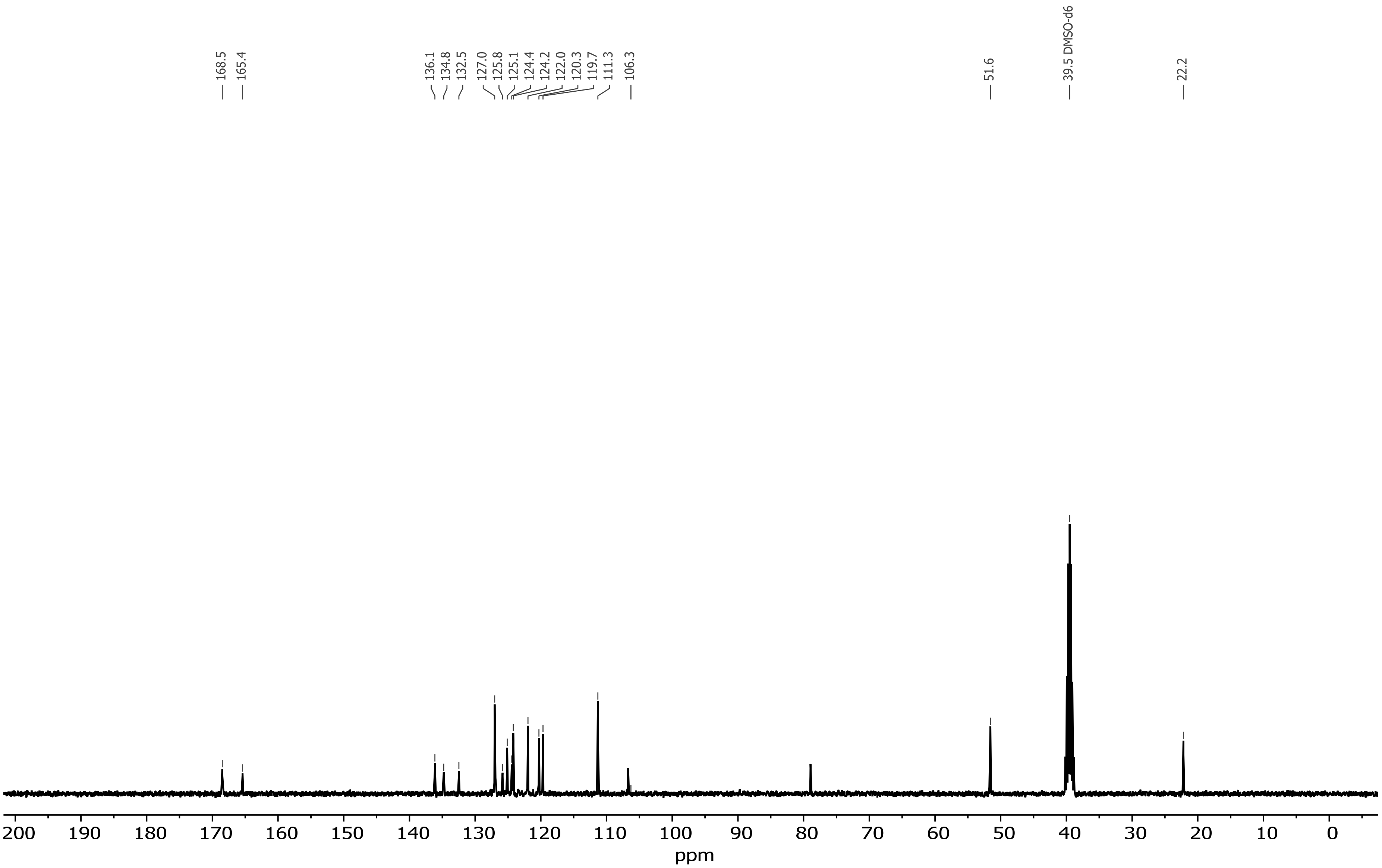

^{1}H NMR (400.16 MHz, MeOD) spectrum of 4o

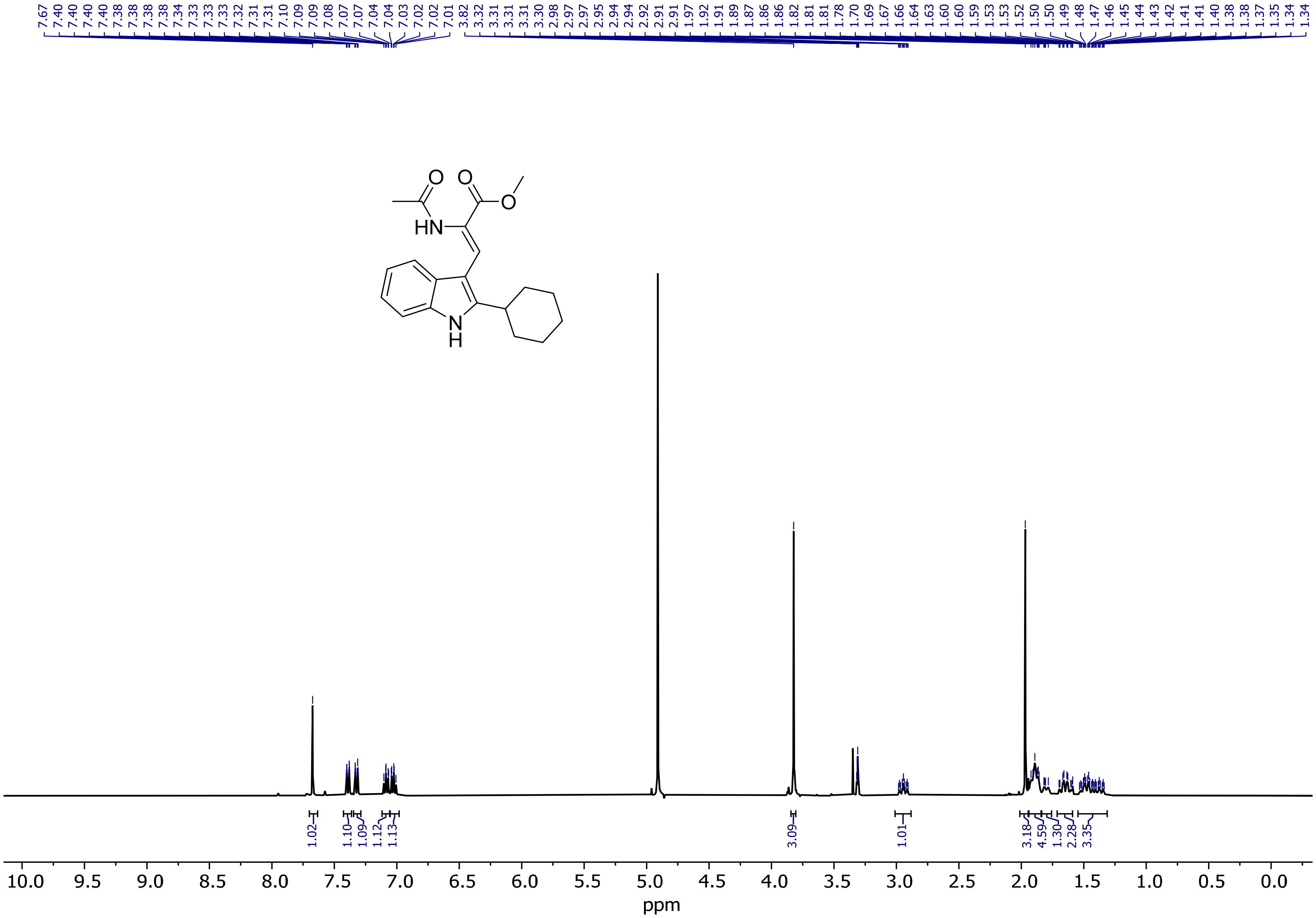

^{13}C {^{1}H} NMR (100.62 MHz, MeOD) spectrum of 4o

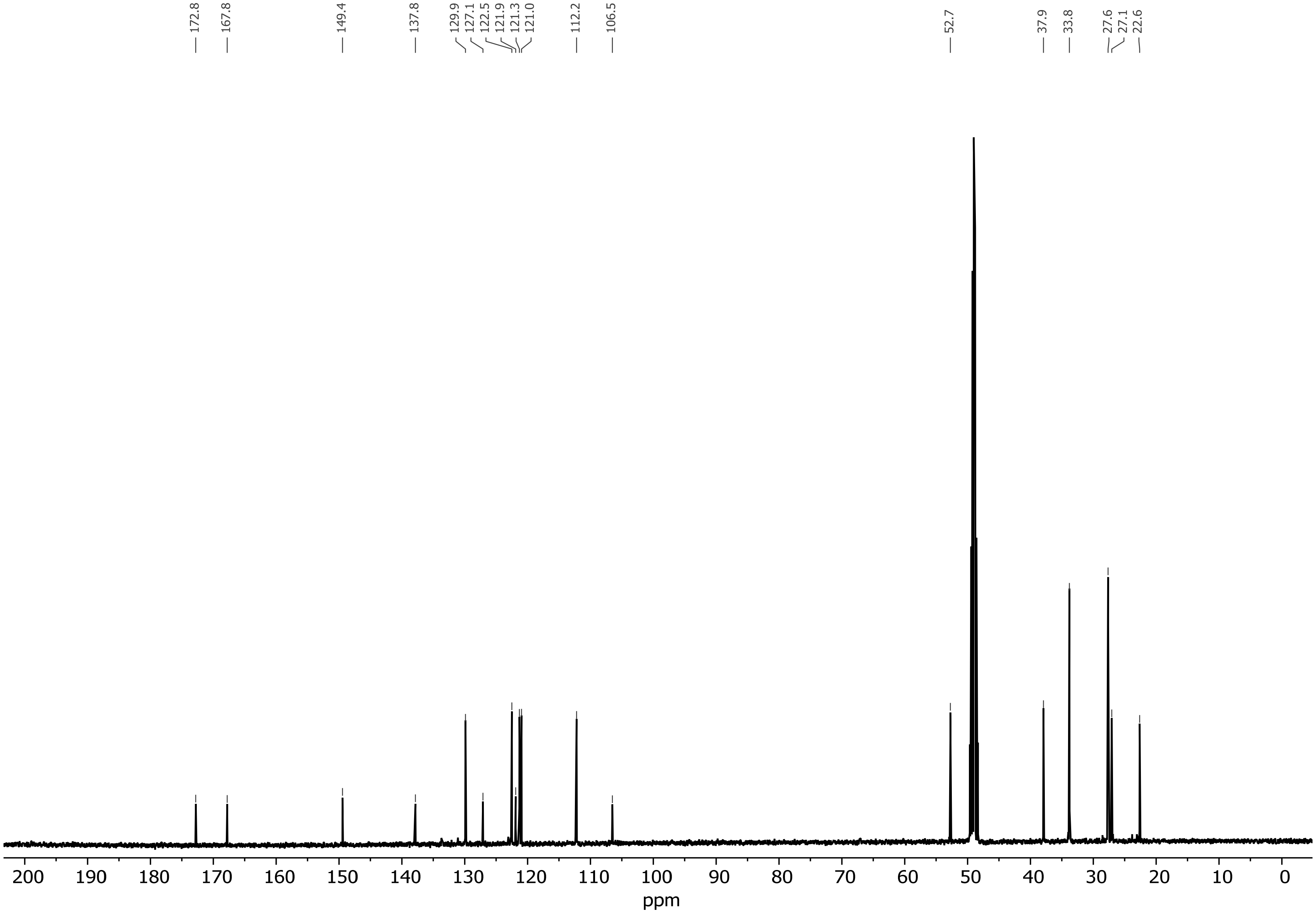

^{1}H NMR (400.16 MHz, $CDCl_3$) spectrum of 4p

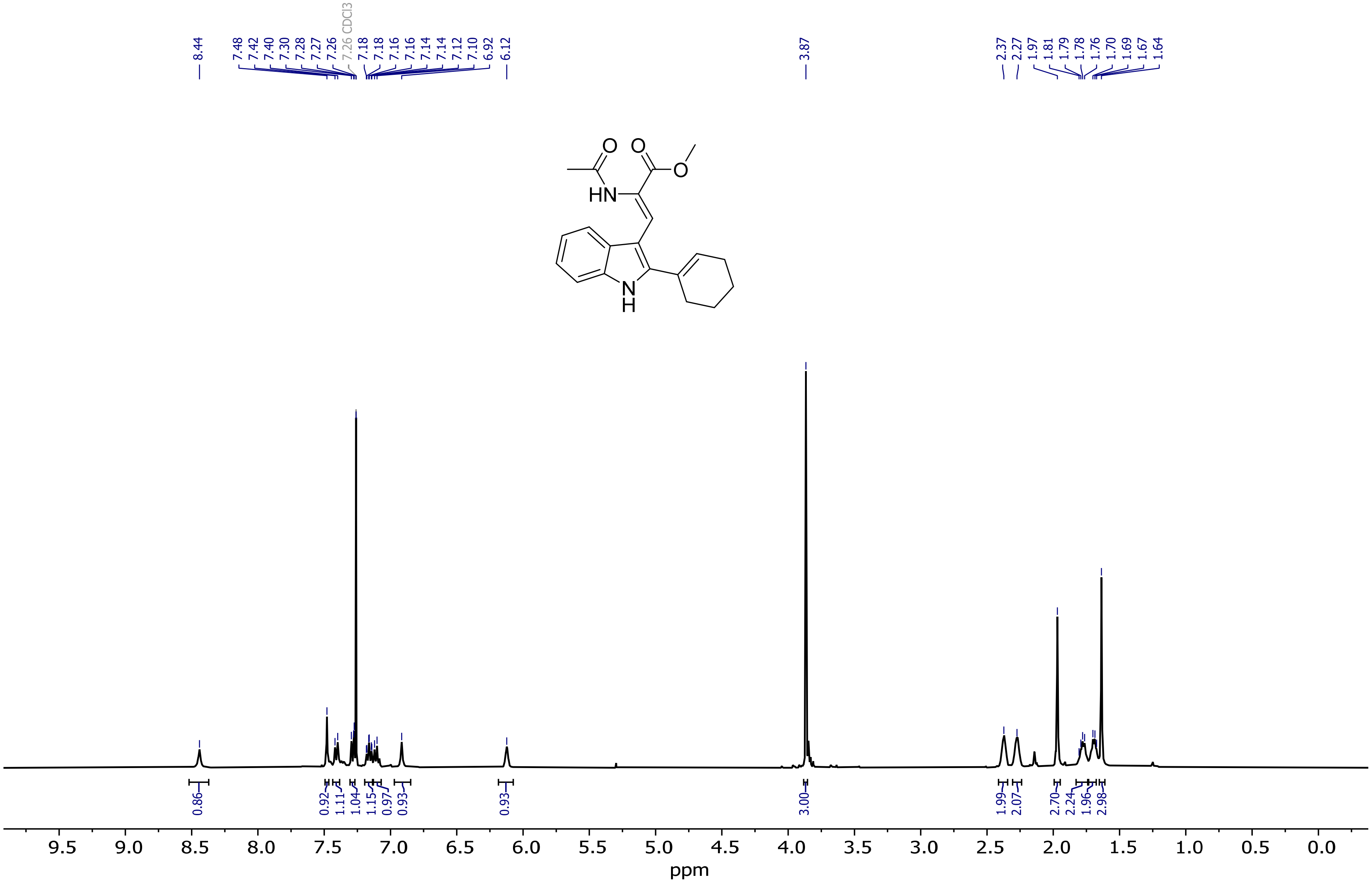

$^{13}C\{^{1}H\}$ NMR (100.16 MHz, $CDCl_3$) spectrum of 4p

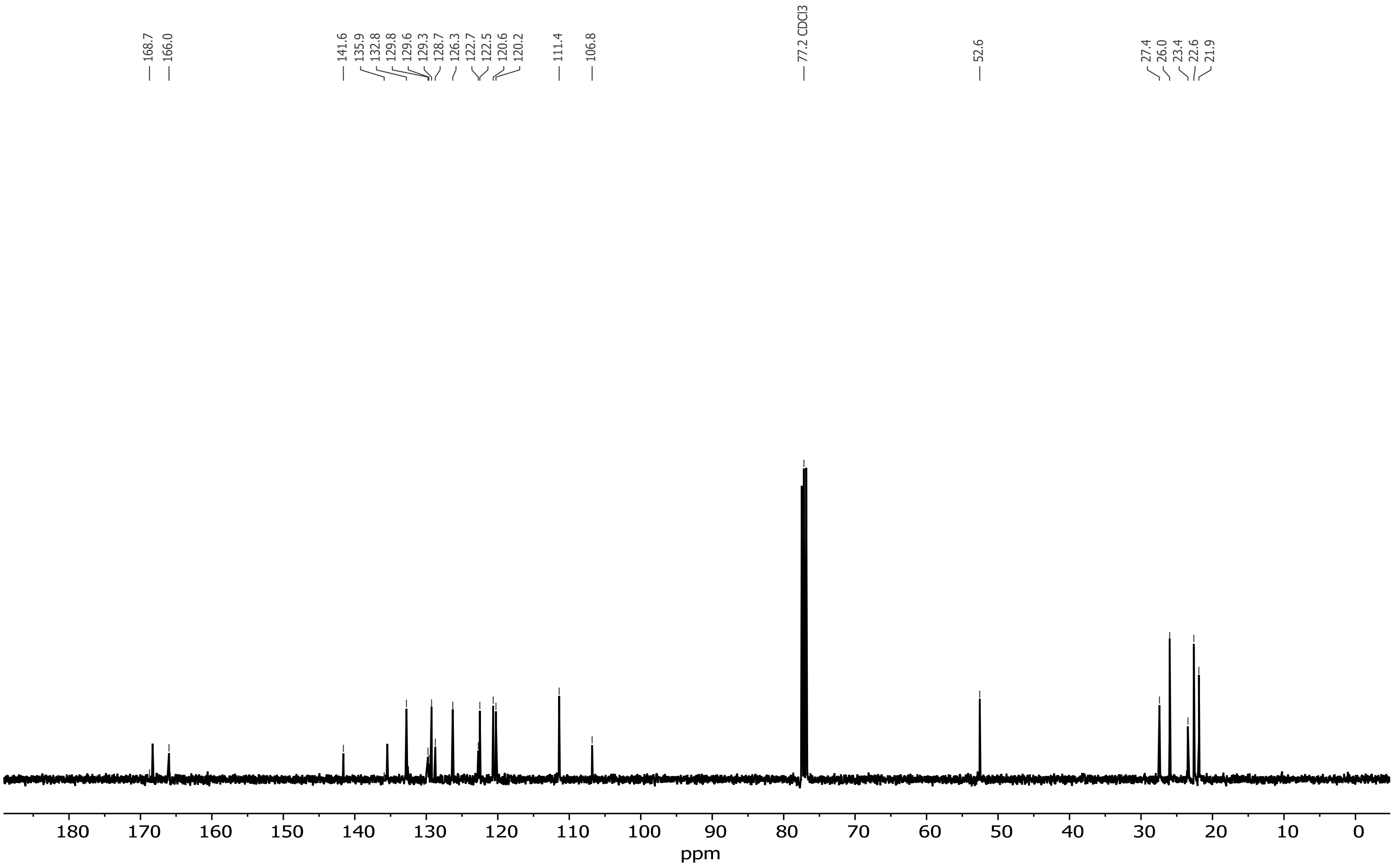

^{1}H NMR (400.16 MHz, 323 K, MeOD) spectrum of 10a

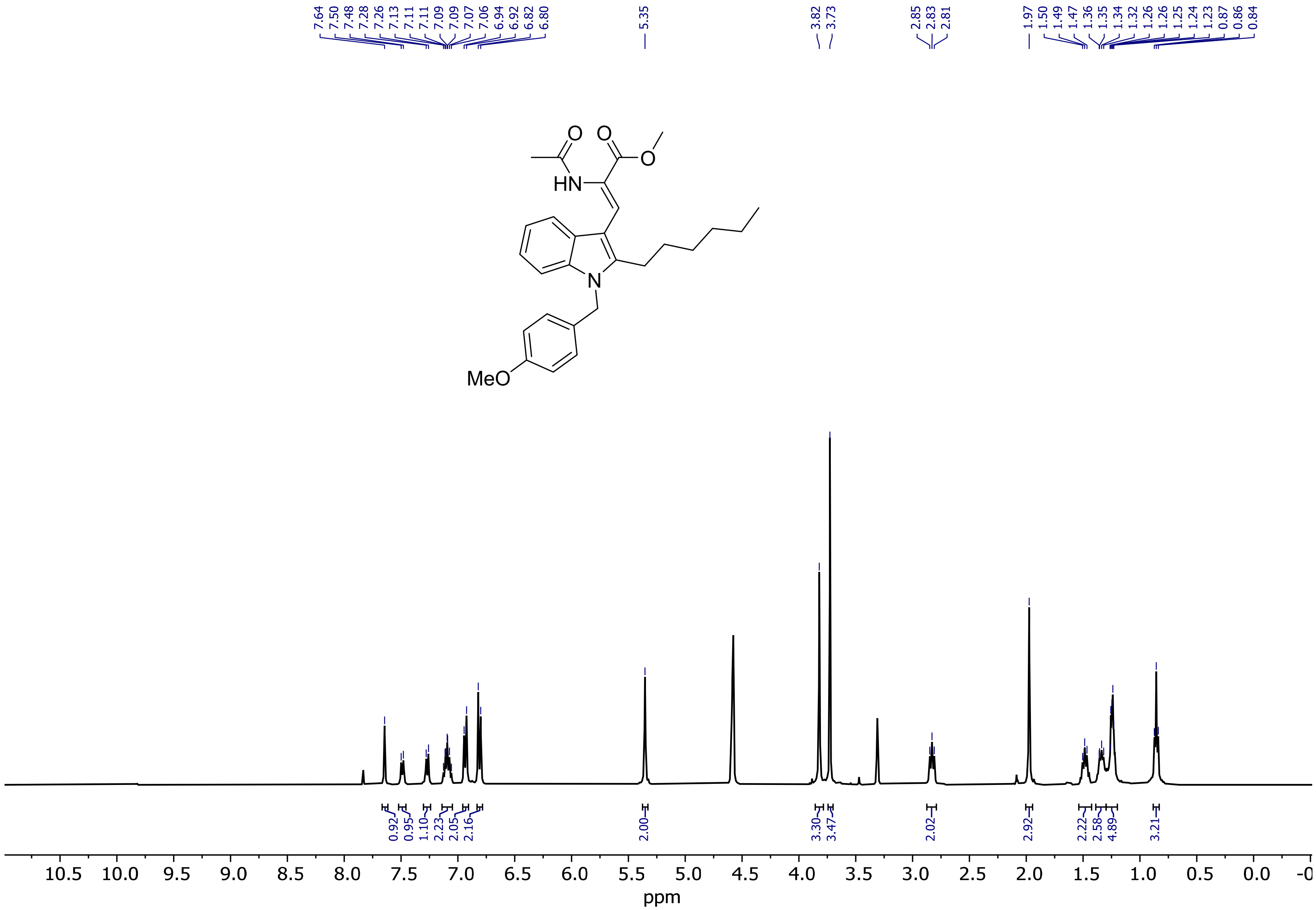

$^{13}C\ \{^{1}H\}$ NMR (100.62 MHz, 323 K, MeOD) spectrum of 10a

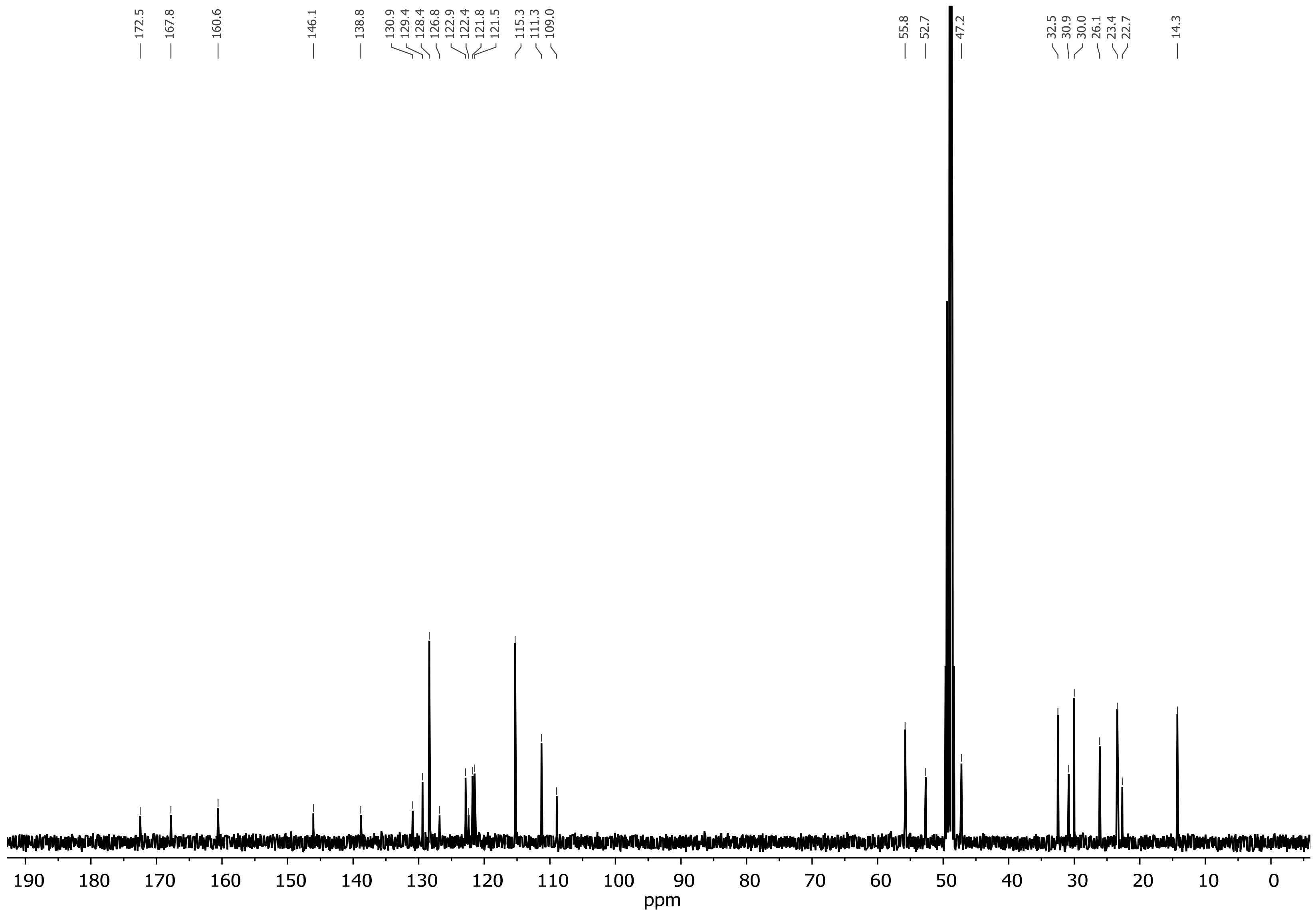

^{1}H NMR (400.16 MHz, 323 K, MeOD) spectrum of 10b

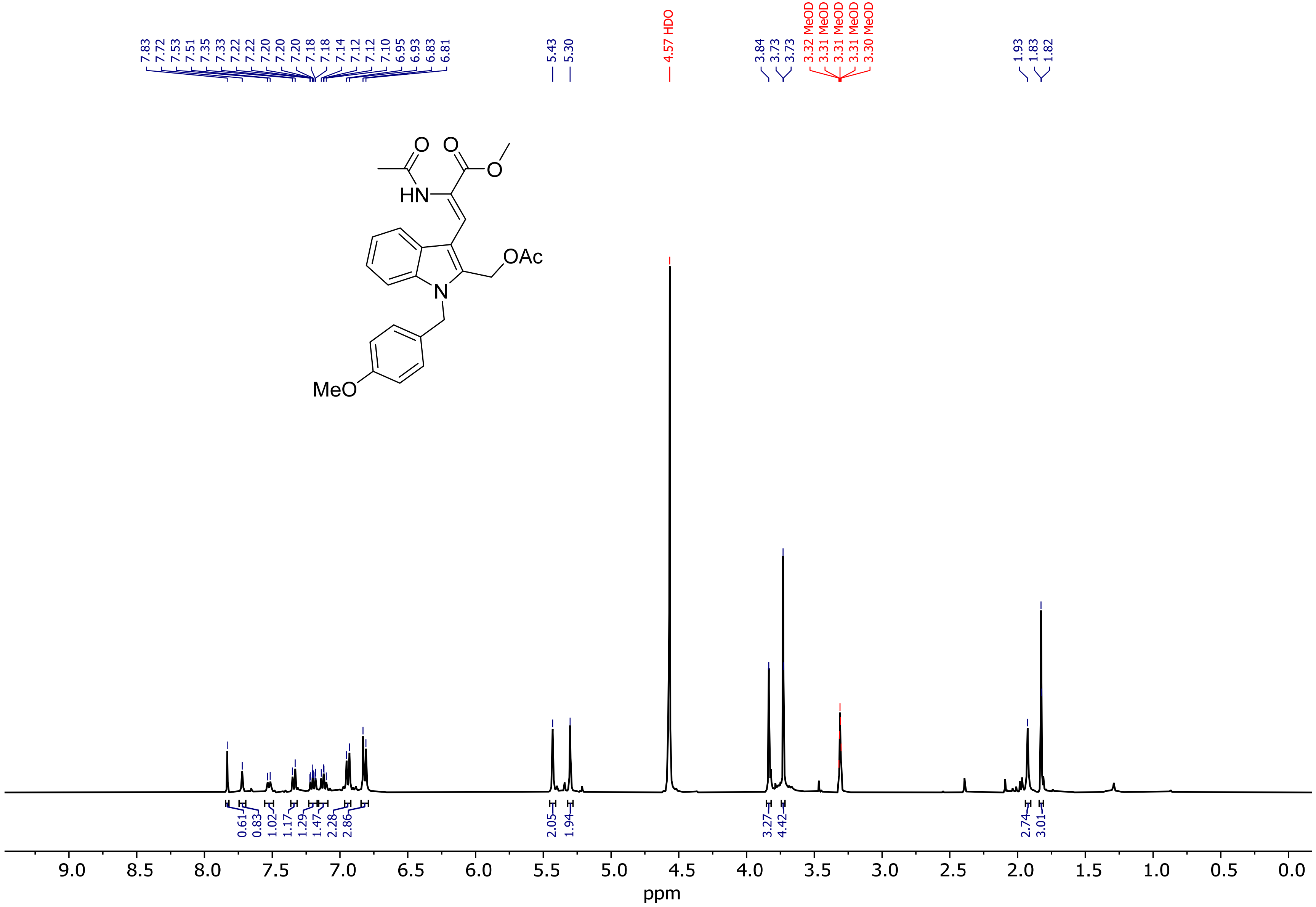

$^{13}C\{^1H\}$ NMR (100.62 MHz, 323 K, MeOD) spectrum of 10b

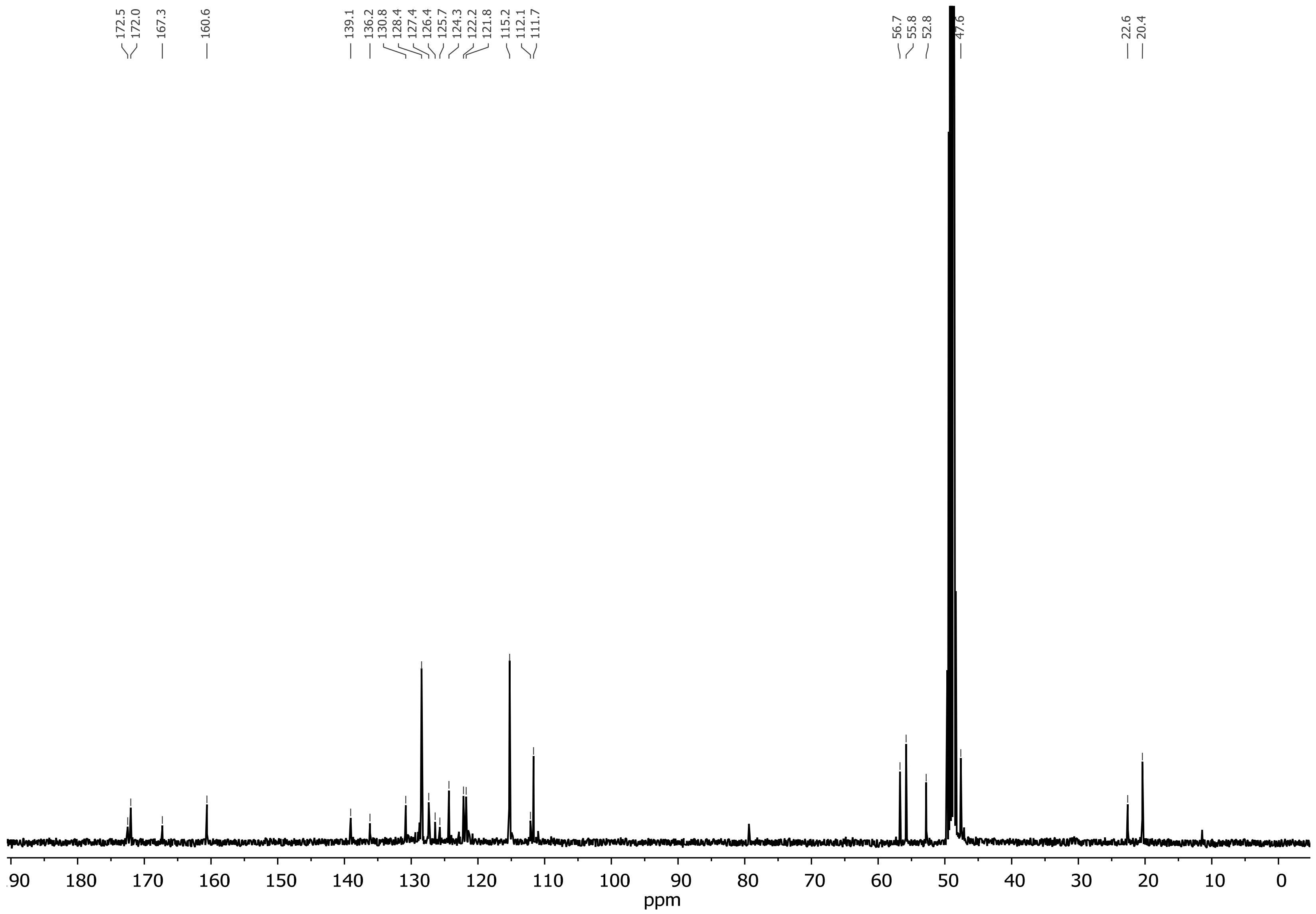

^{1}H NMR (400.16 MHz, $CDCl_3$) spectrum of 10c

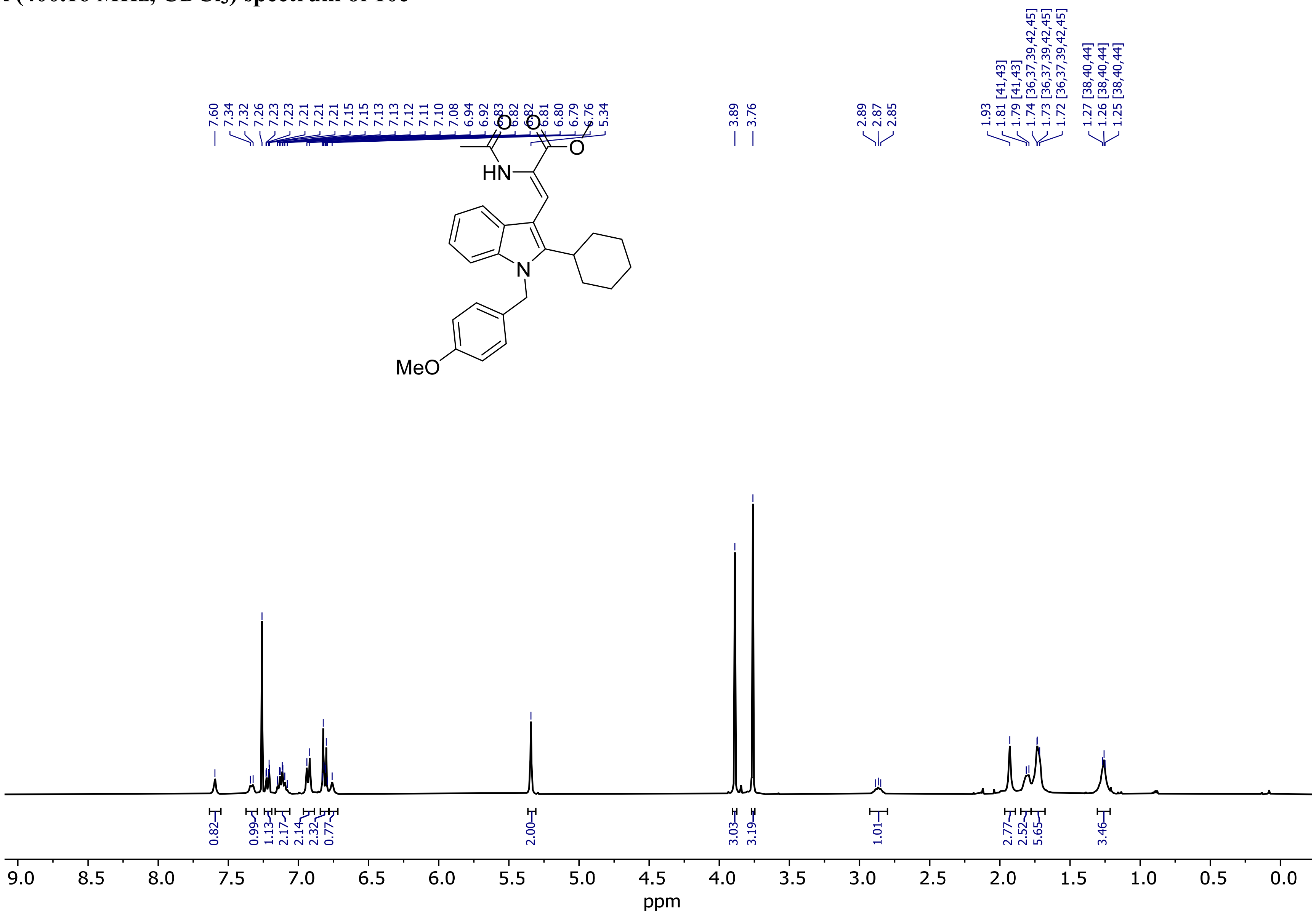

^{13}C {^{1}H} NMR (100.62 MHz, $CDCl_3$) spectrum of 10c

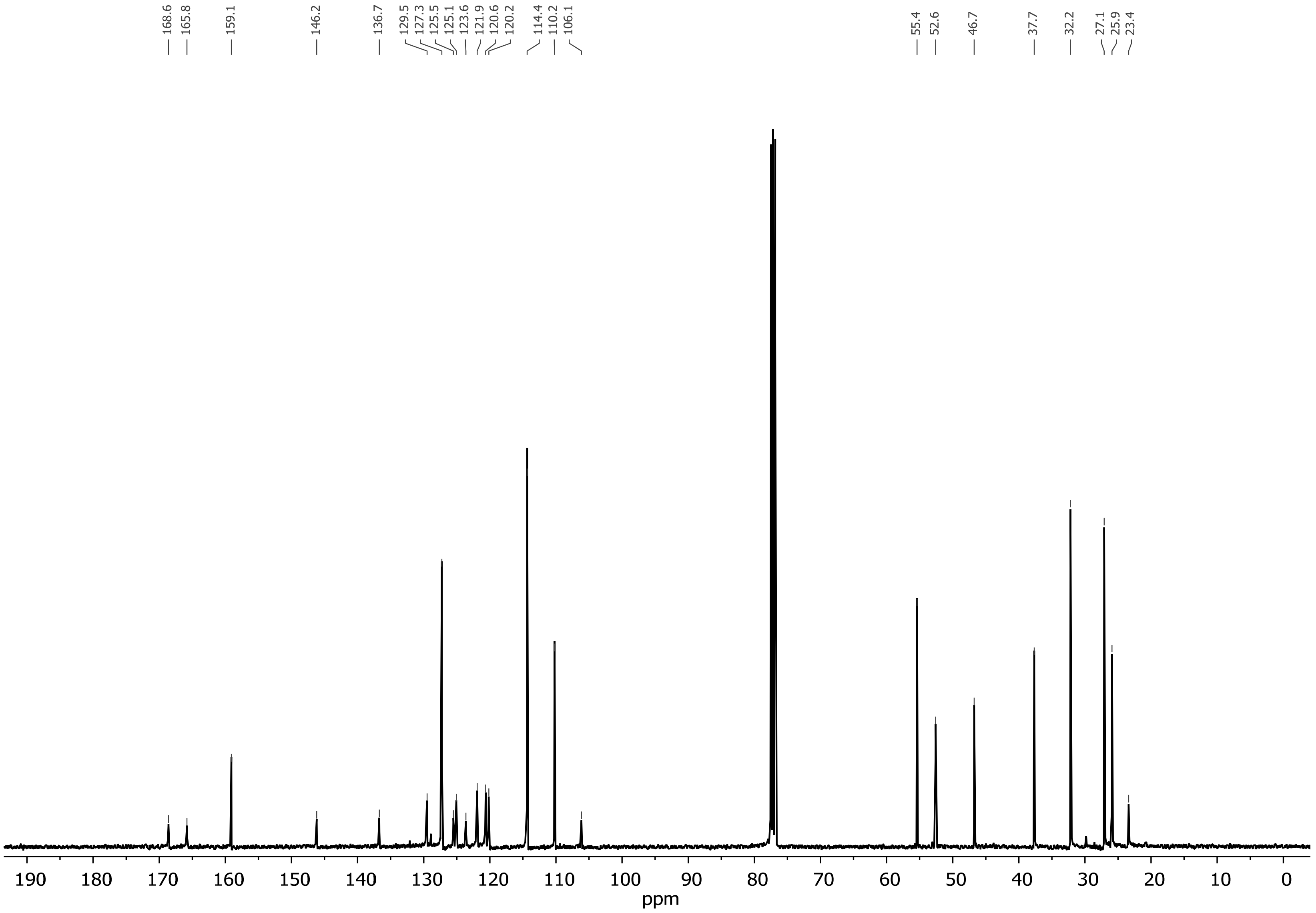

^{1}H NMR (400.16 MHz, $CDCl_3$) spectrum of 10d

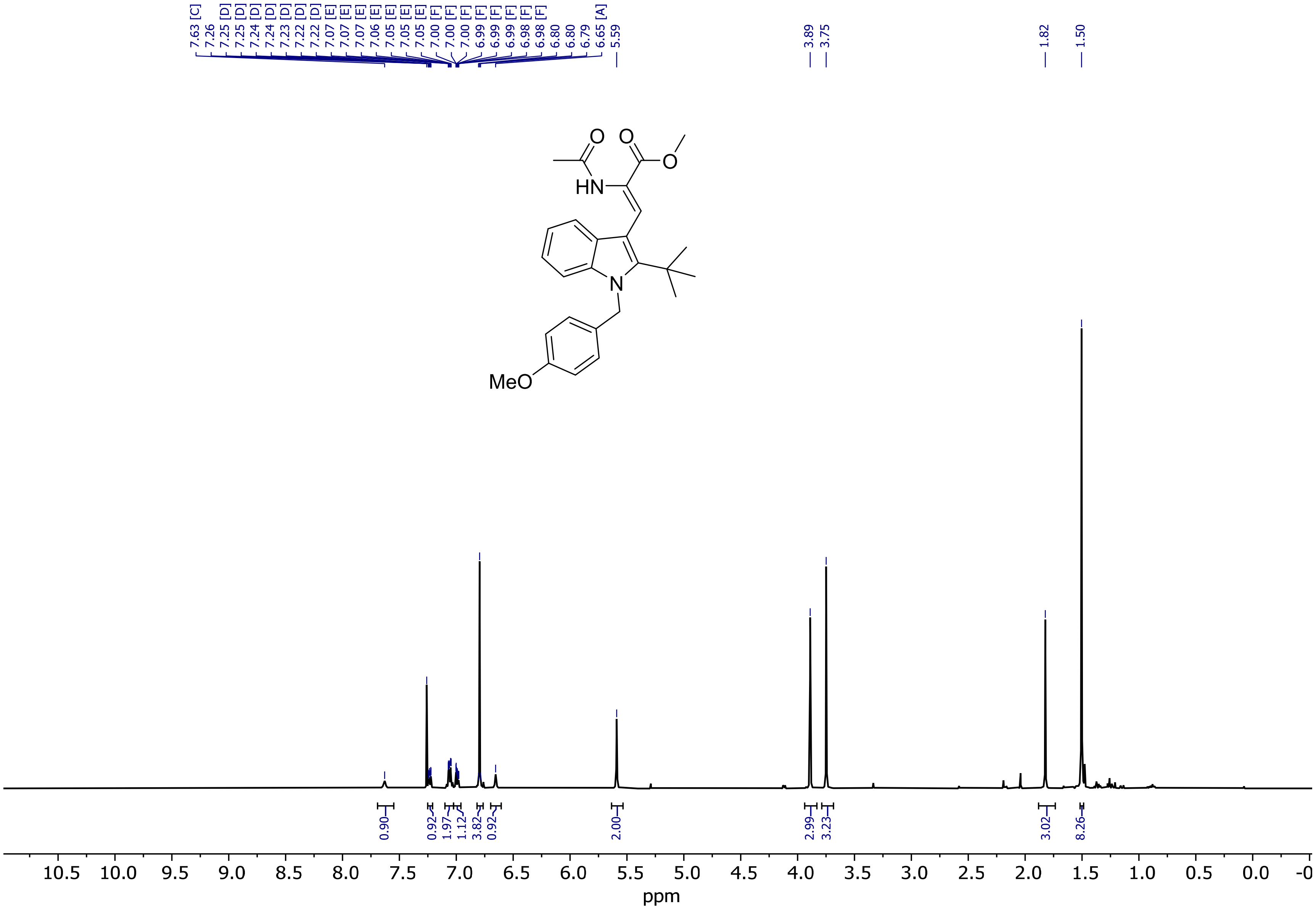

^{13}C {^{1}H} NMR (100.62 MHz, $CDCl_3$) spectrum of 10d

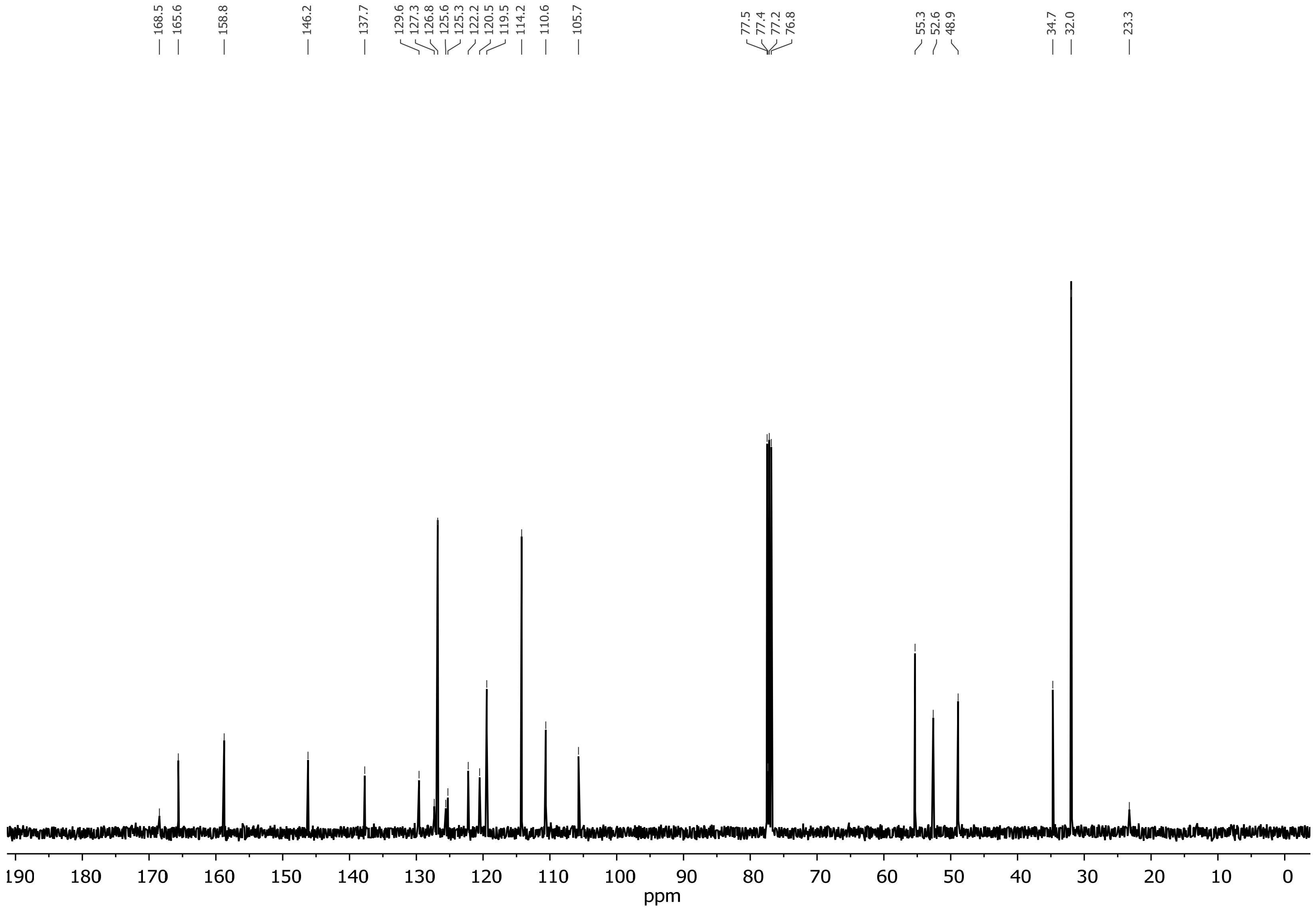

^{1}H NMR (400.16 MHz, $CDCl_3$) spectrum of 11d

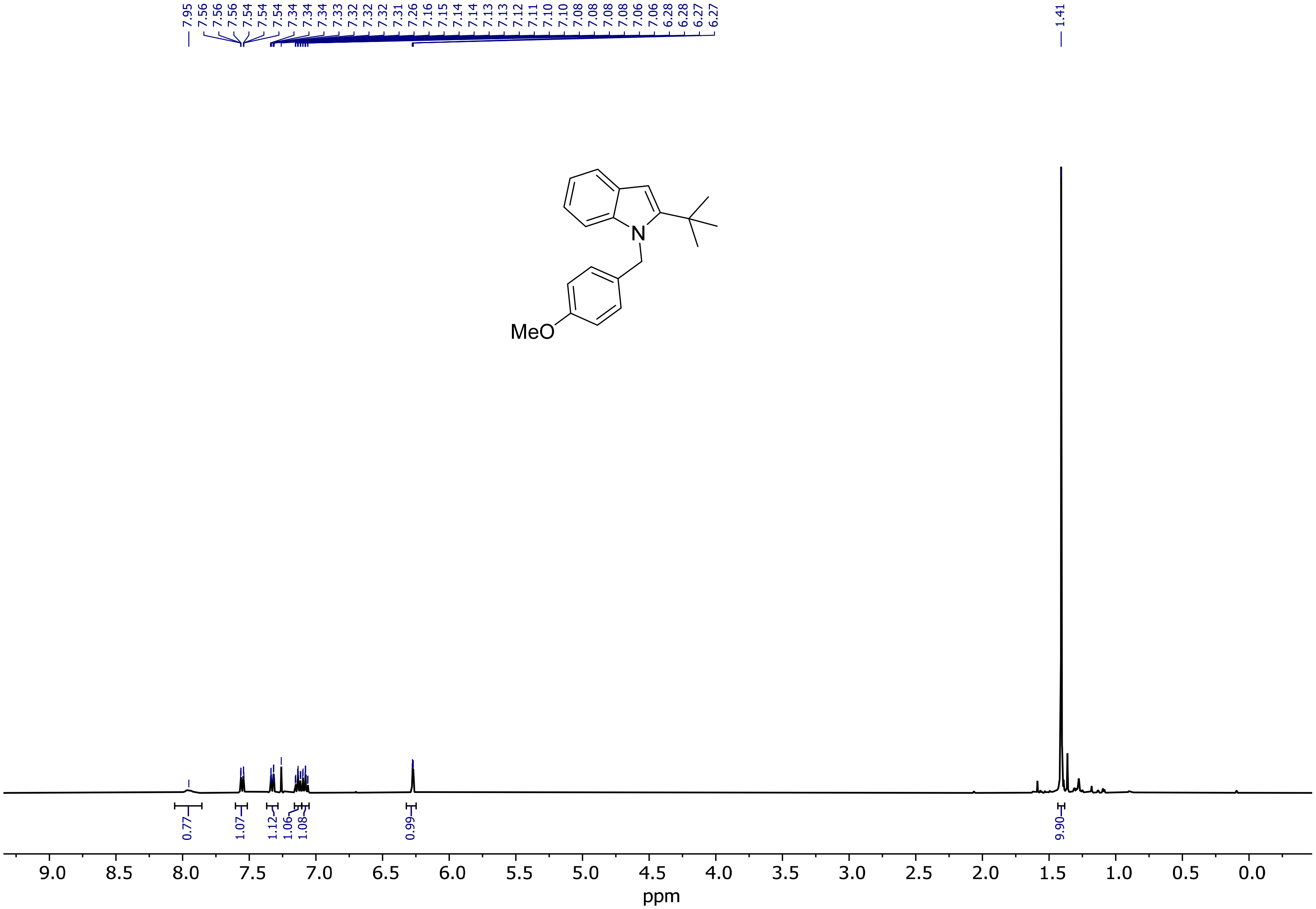

$^{13}C\{^1H\}$ NMR (100.62 MHz, $CDCl_3$) spectrum of 11d

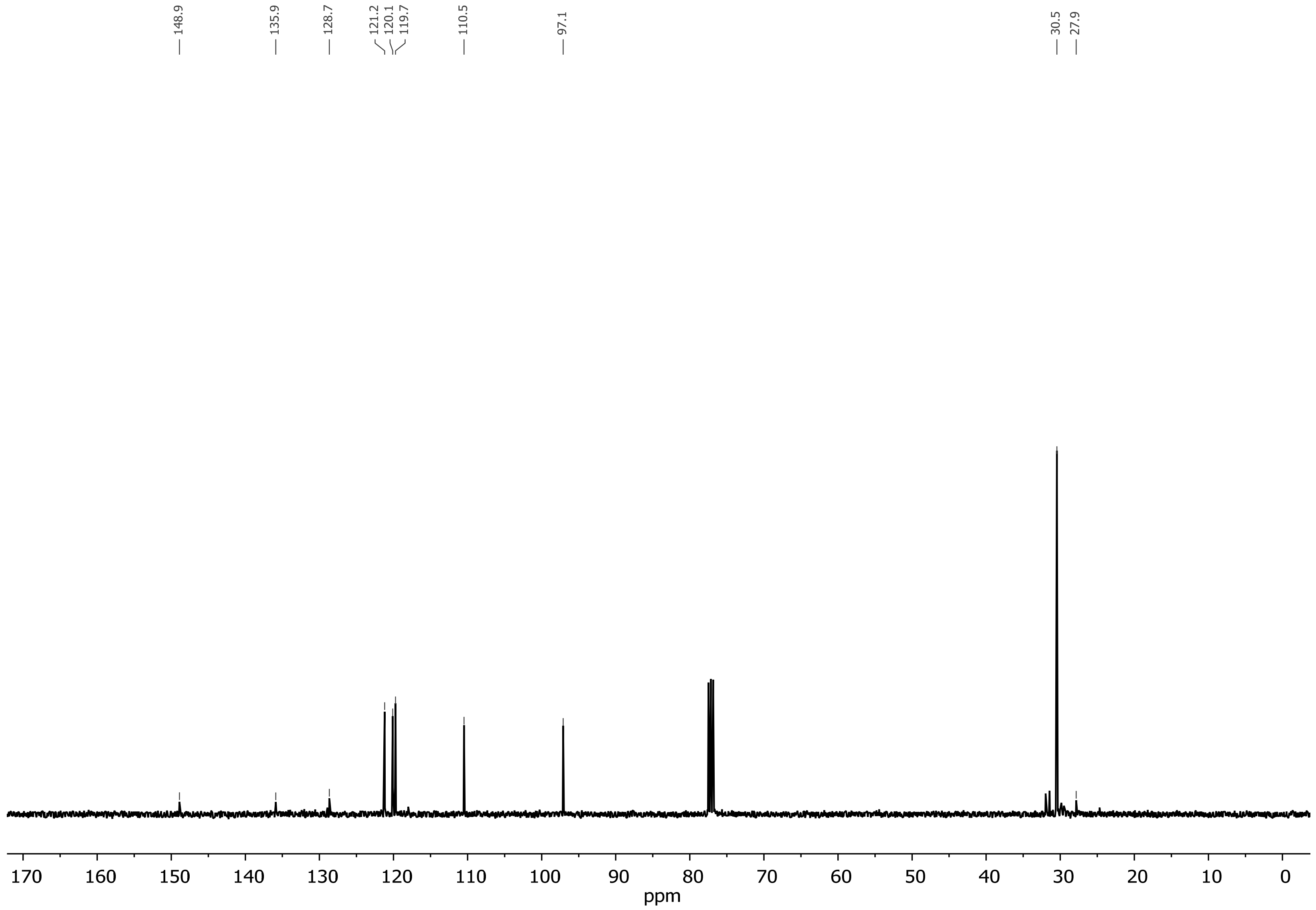

^{1}H NMR (400.16 MHz, 323 K, MeOD) spectrum of 10e

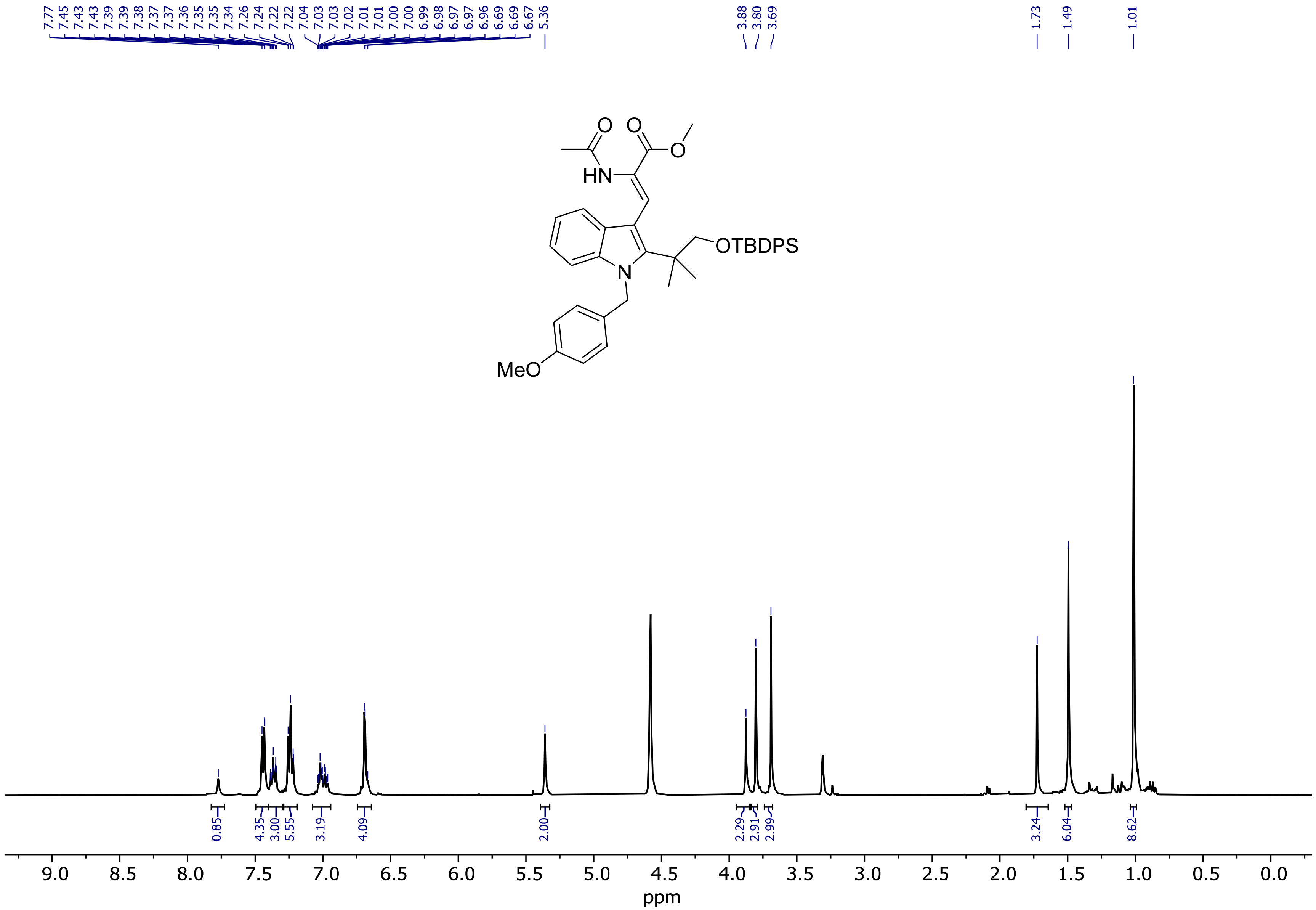

^{13}C {^{1}H} NMR (100.62 MHz, 323 K, MeOD) spectrum of 10e

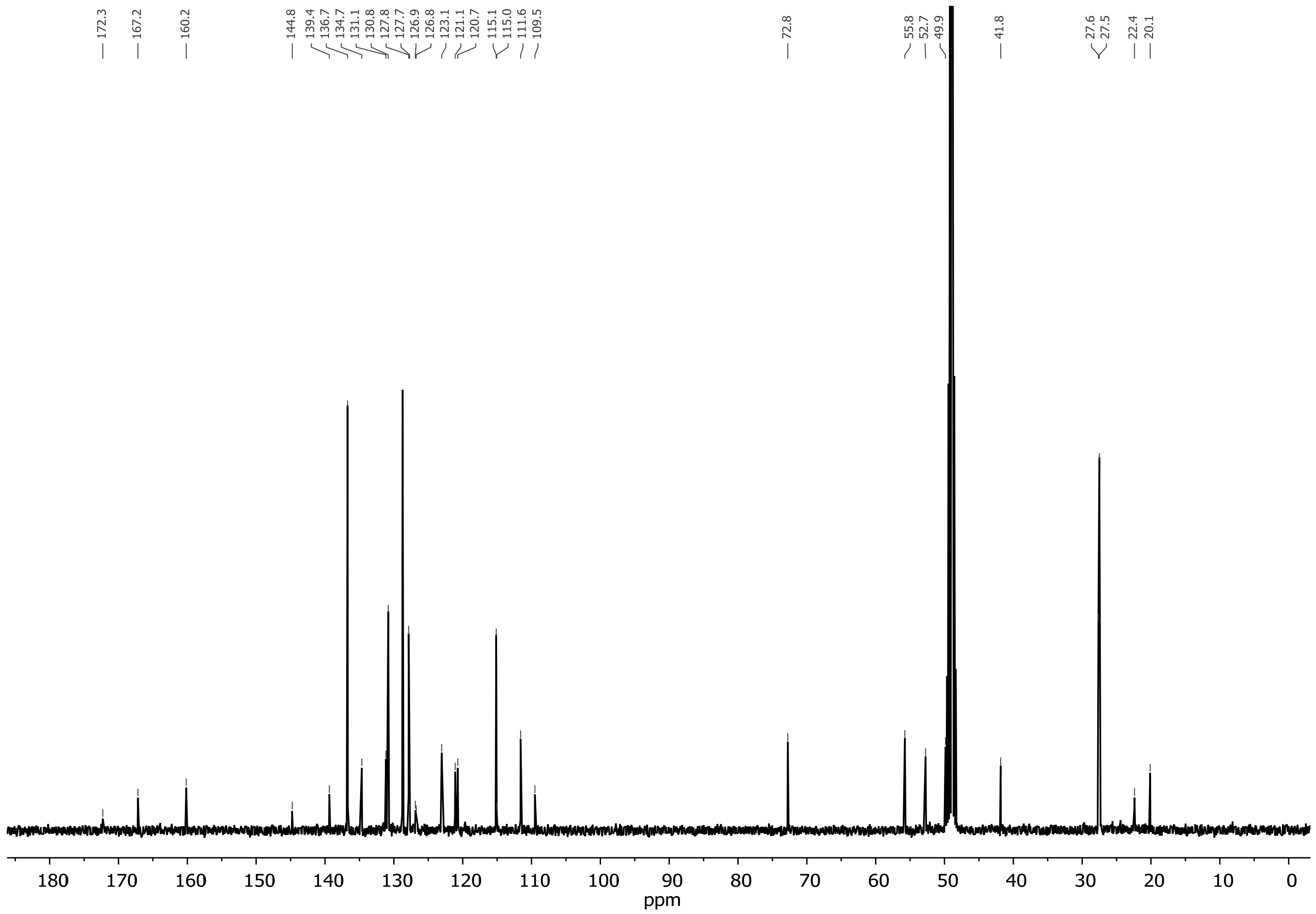

^{1}H NMR (400.16 MHz, $CDCl_3$) spectrum of 10f

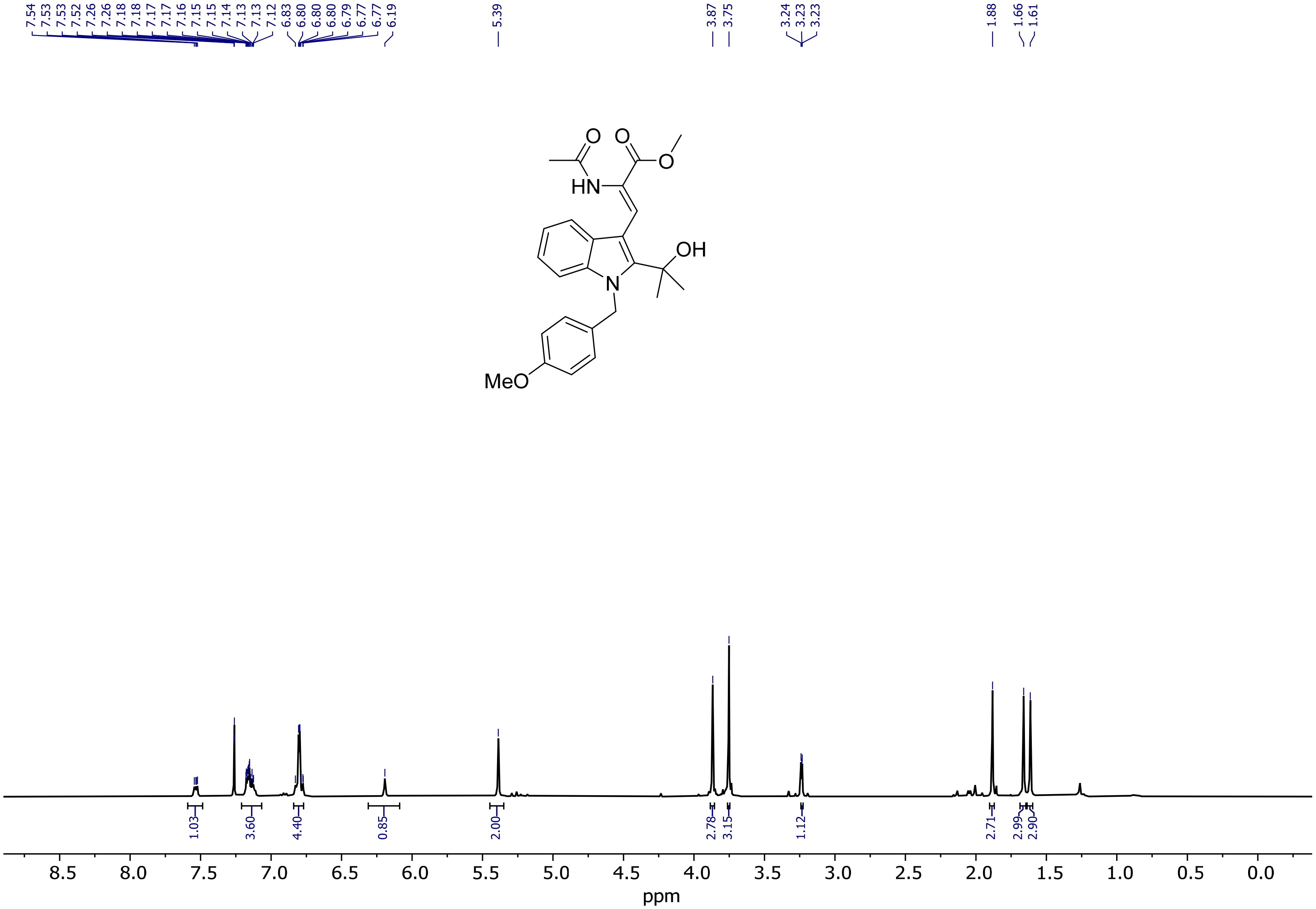

$^{13}C\{^{1}H\}$ NMR (100.62 MHz, $CDCl_3$) spectrum of 10f

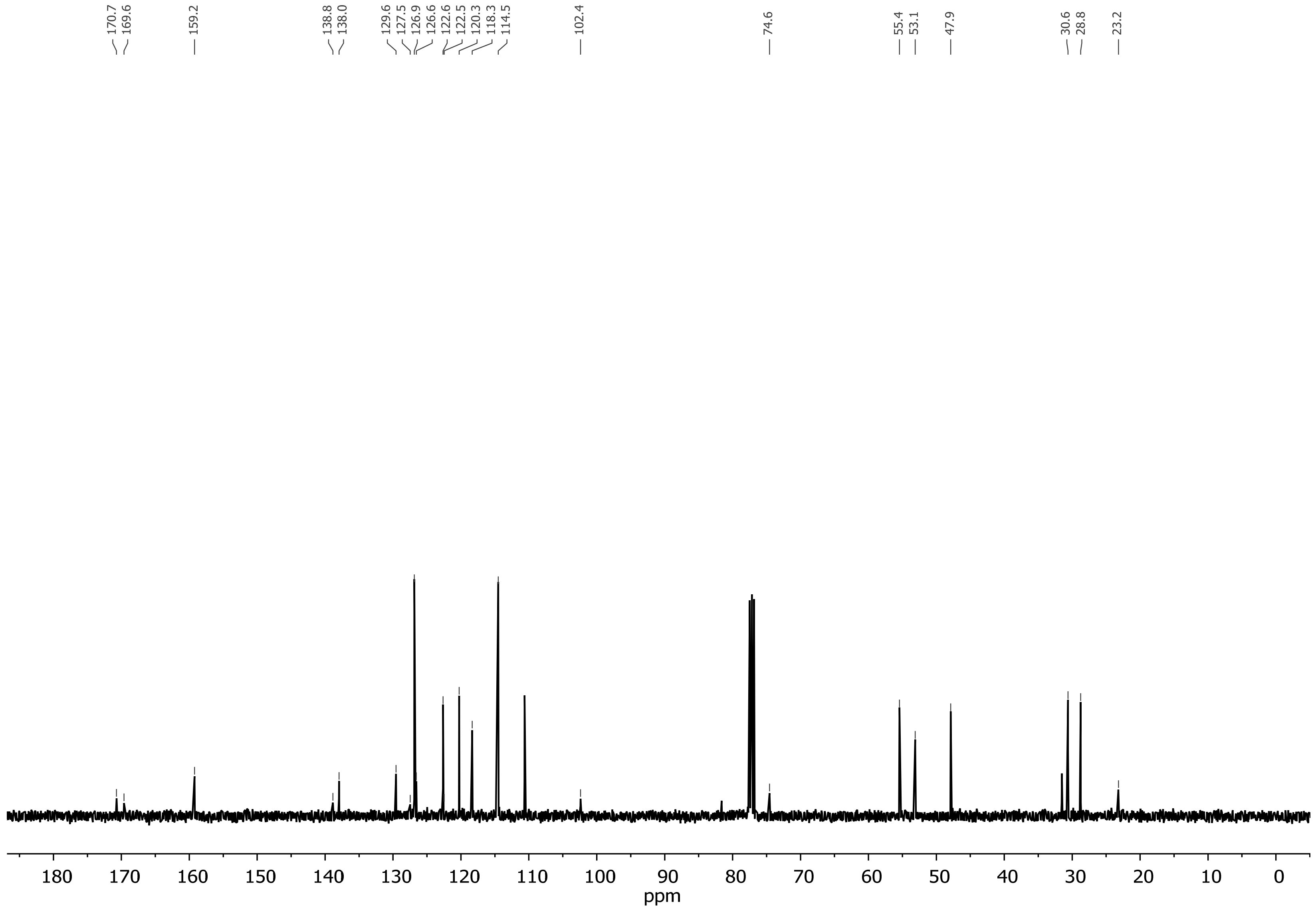

^{1}H NMR (400.16 MHz, CDCl$_3$) spectrum of 10g

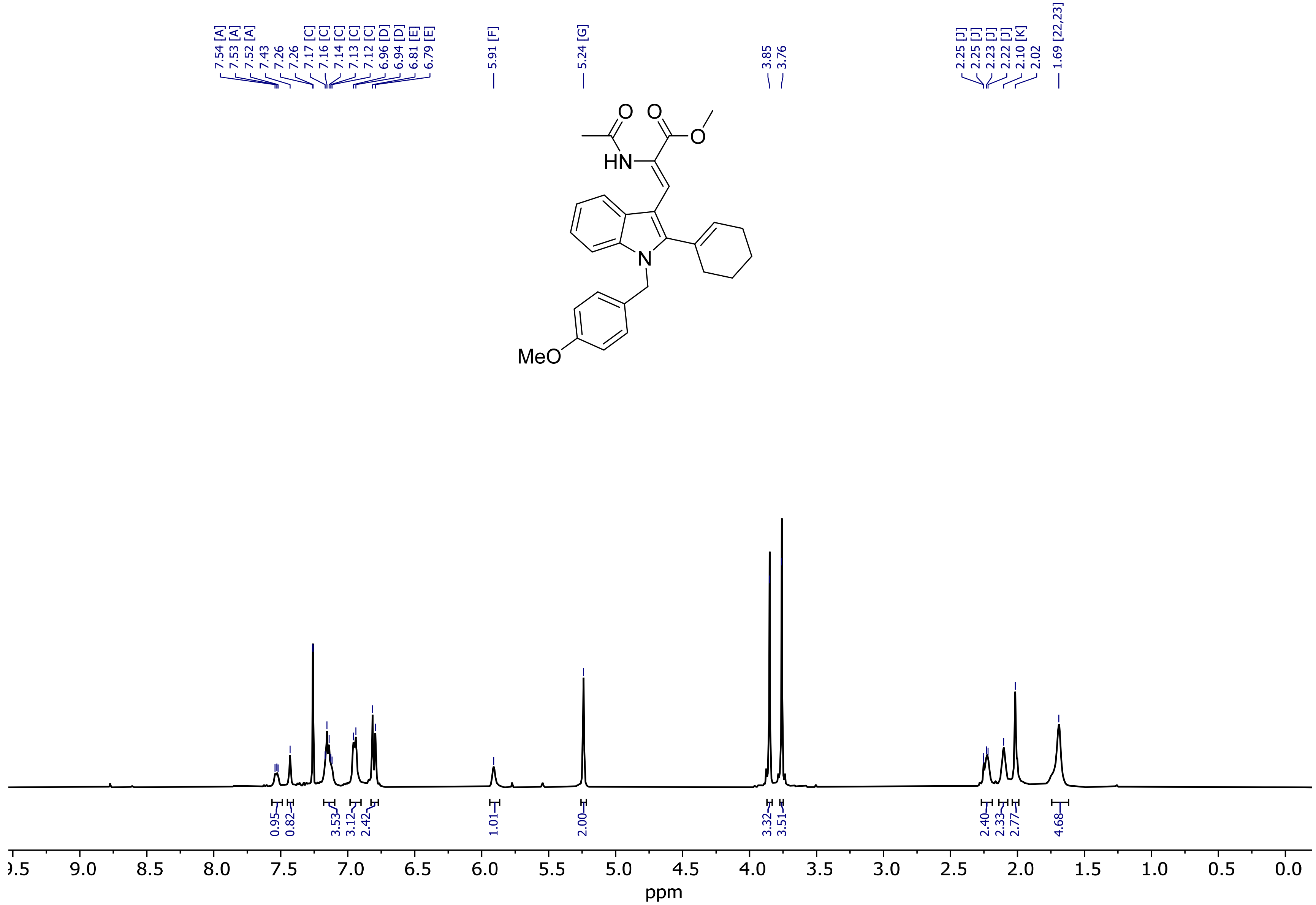

$^{13}C\{^1H\}$ NMR (100.62 MHz, $CDCl_3$) spectrum of 10g

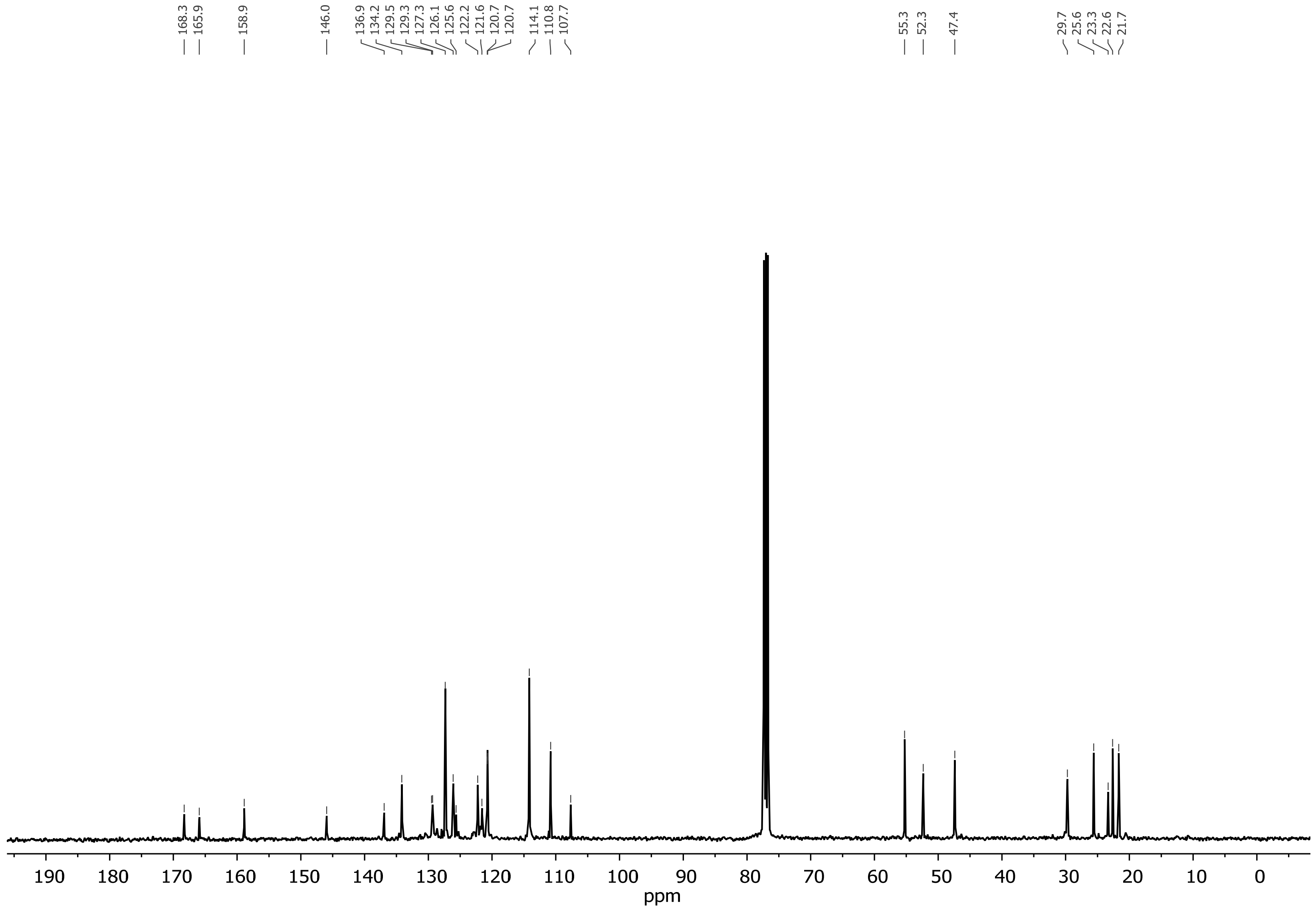

^{1}H NMR (400.16 MHz, $CDCl_3$) spectrum of 10h

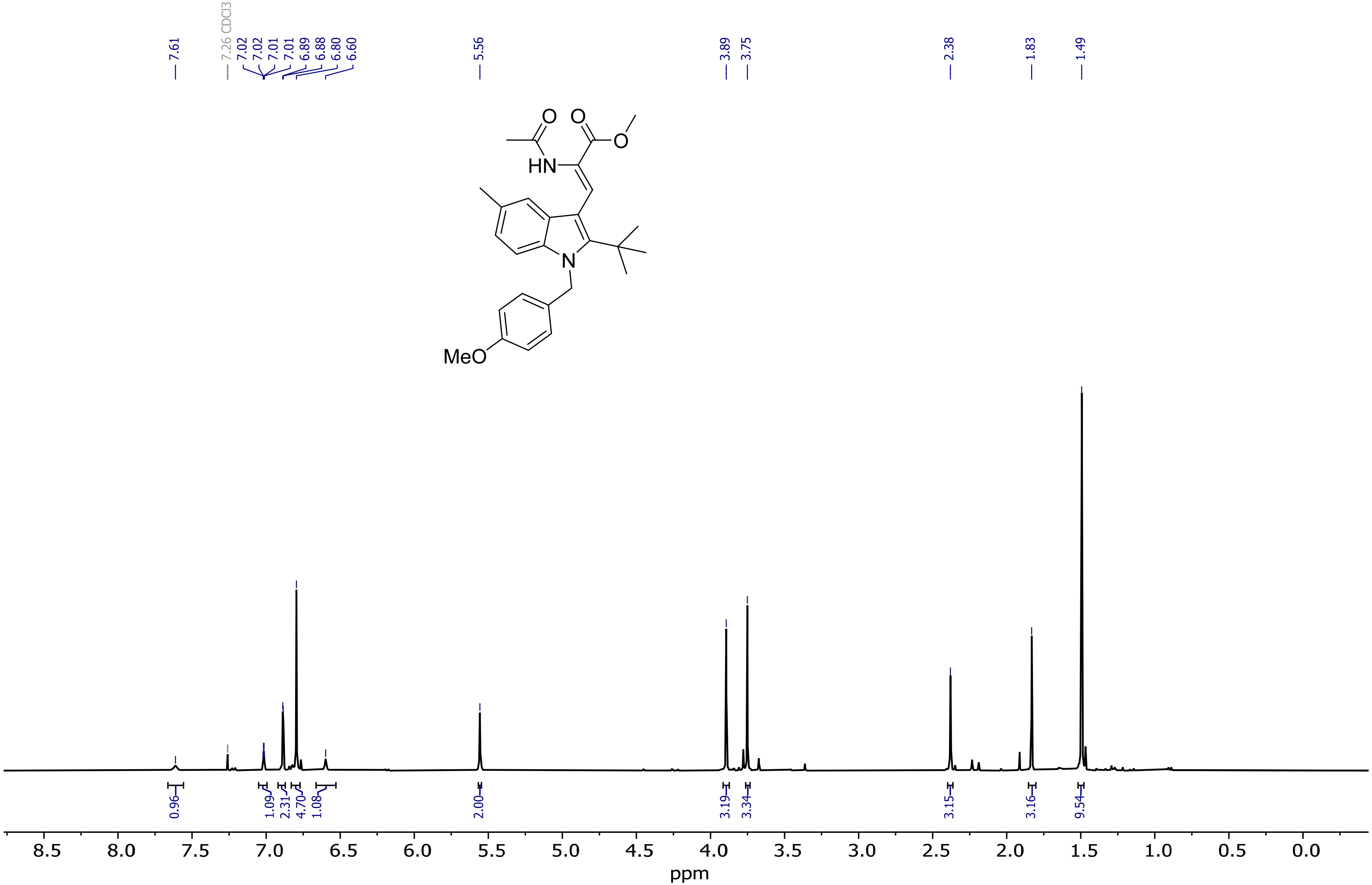

$^{13}C\{^1H\}$ NMR (100.62 MHz, $CDCl_3$) spectrum of 10h

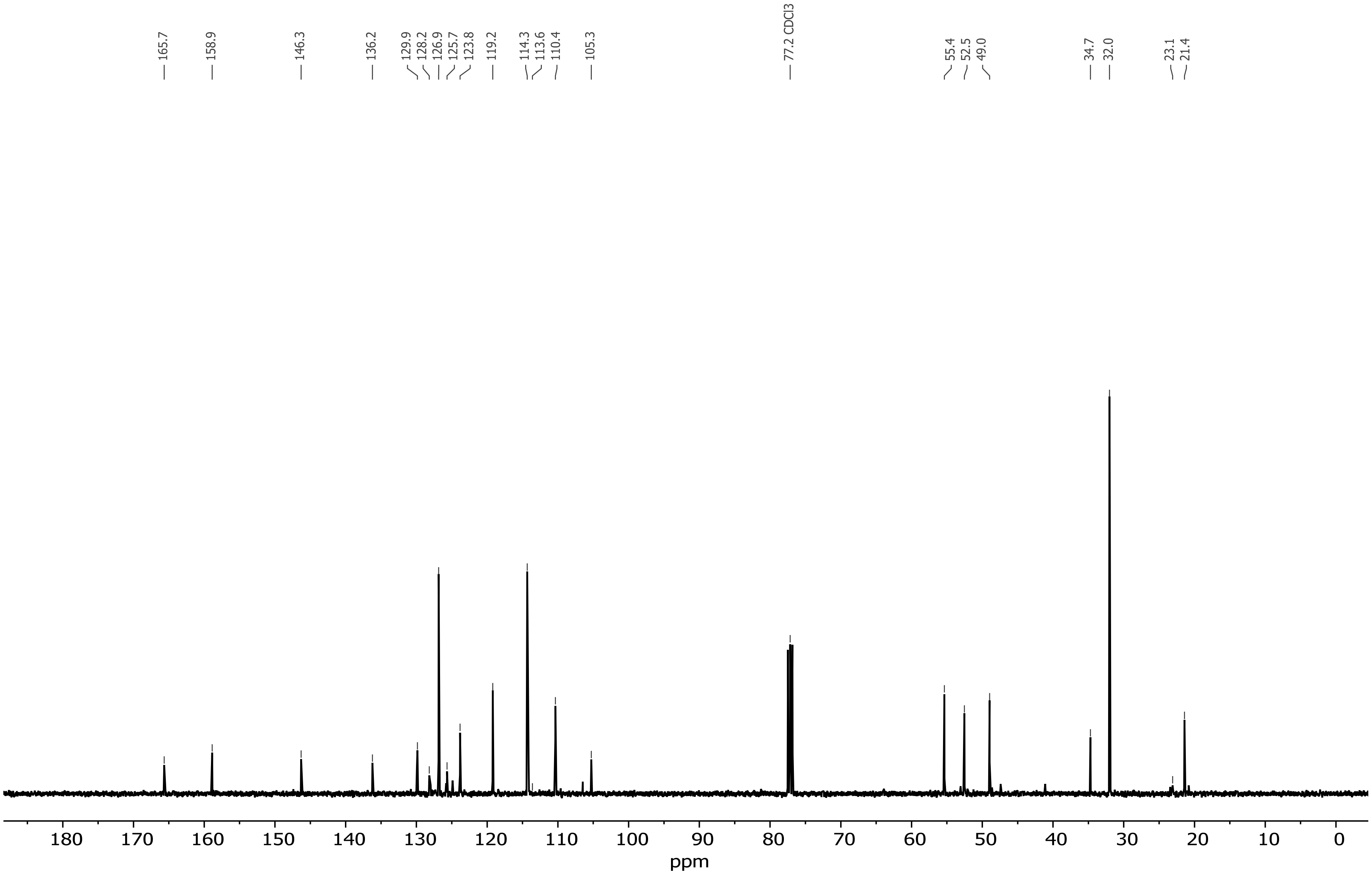

^{1}H NMR (400.16 MHz, $CDCl_3$) spectrum of 10i

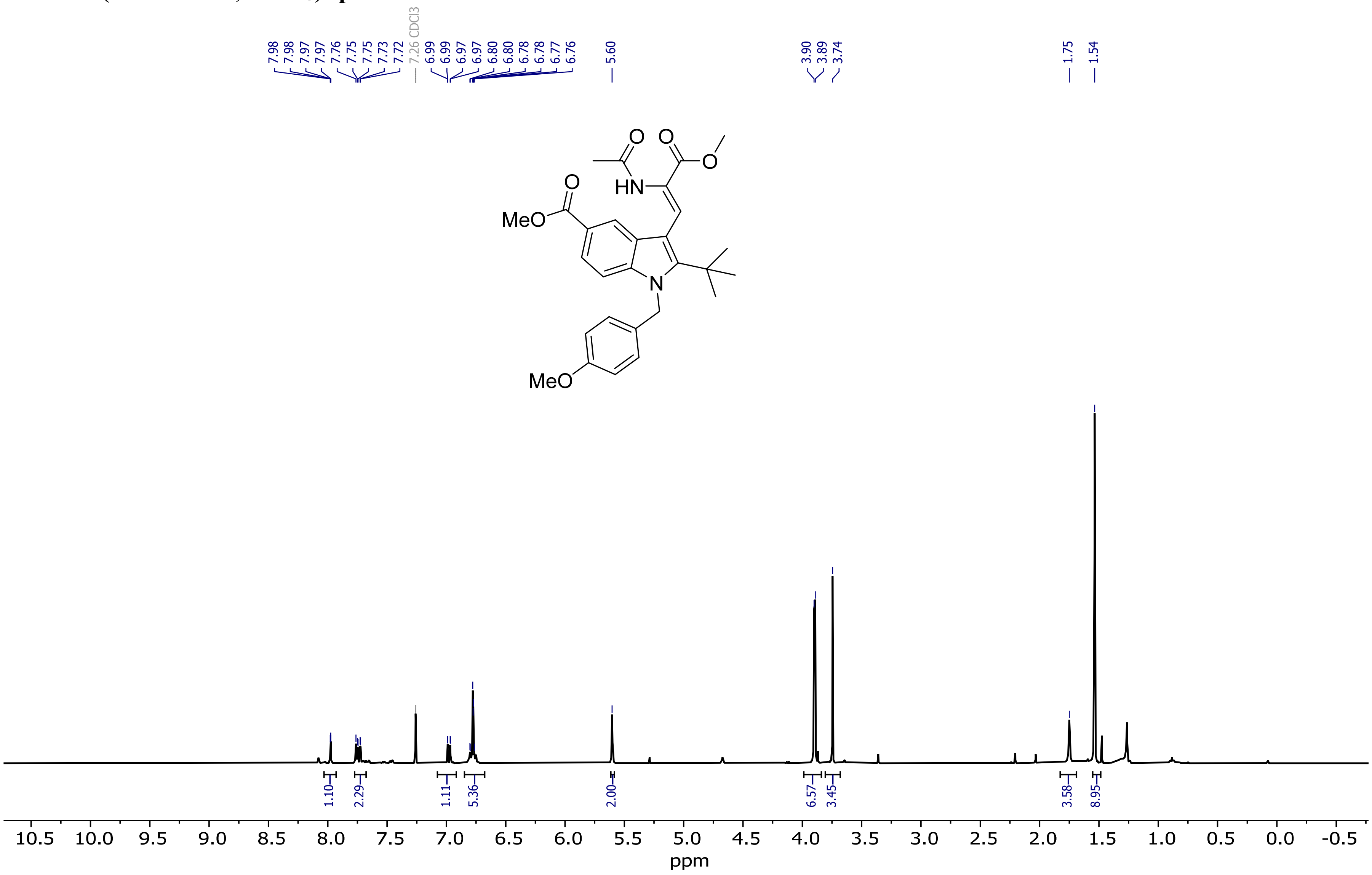

$^{13}C\{^1H\}$ NMR (100.62 MHz, $CDCl_3$) spectrum of 10i

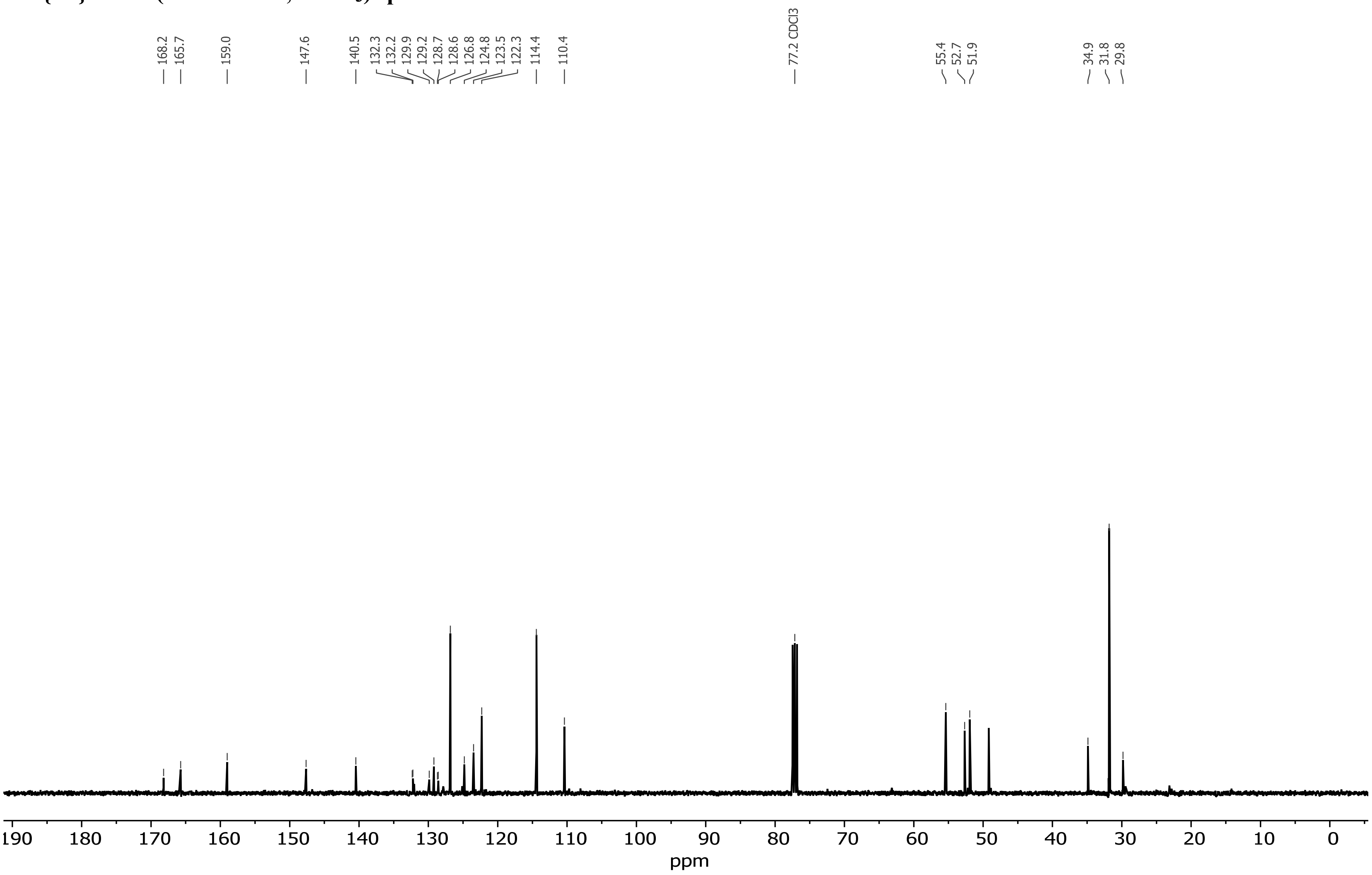

^{1}H NMR (400.16 MHz, DMSO-d₆) spectrum of 10j

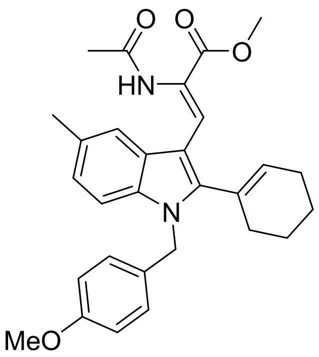

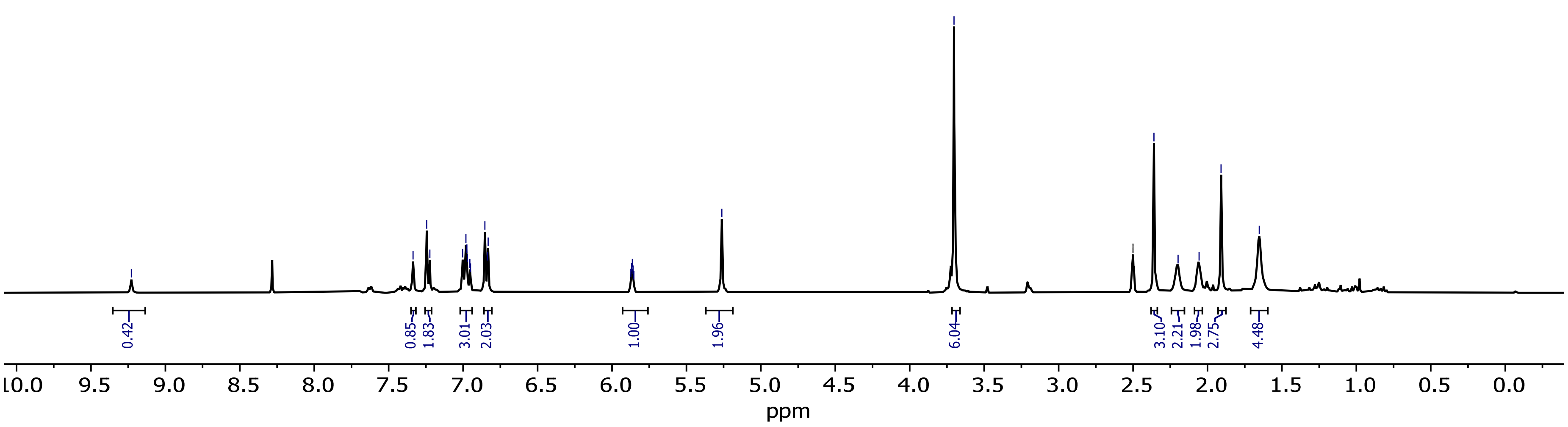

^{13}C {^{1}H} NMR (100.62 MHz, DMSO-d_6) spectrum of 10j

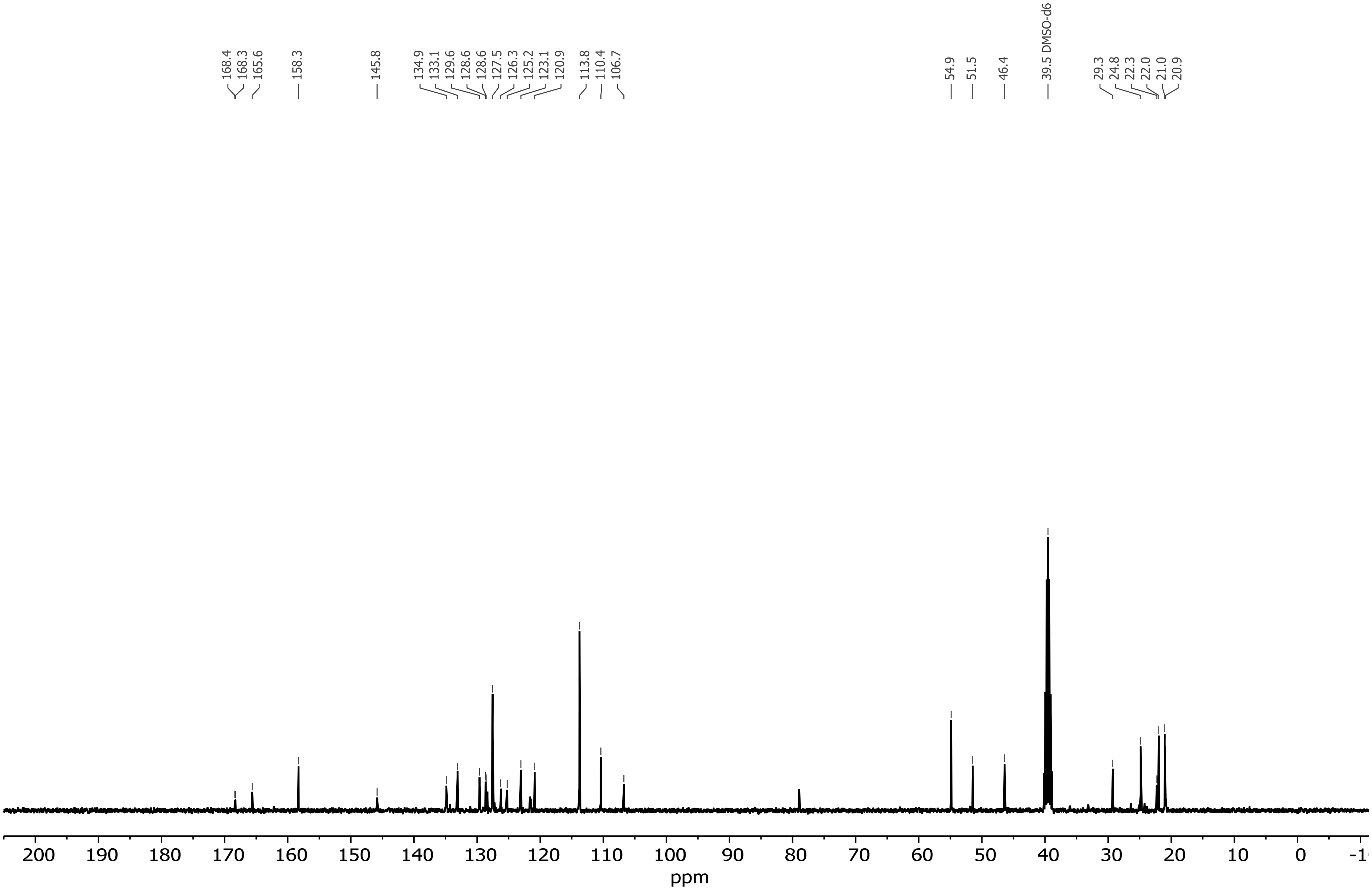

^{1}H NMR (400.16 MHz, DMSO-d$_6$) spectrum of 12

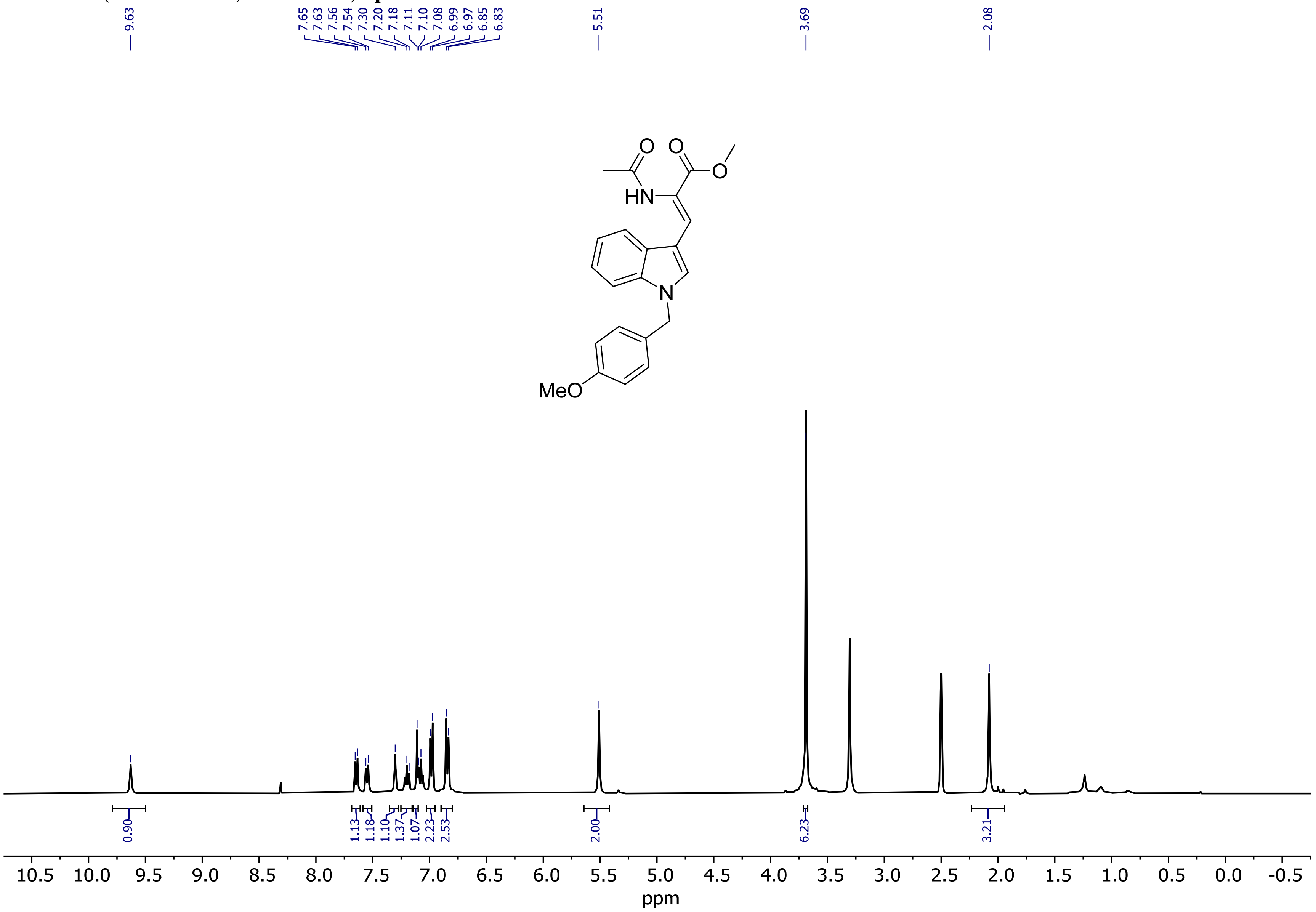

$^{13}C\{^{1}H\}$ NMR (100.62 MHz, DMSO-d$_6$) spectrum of 12

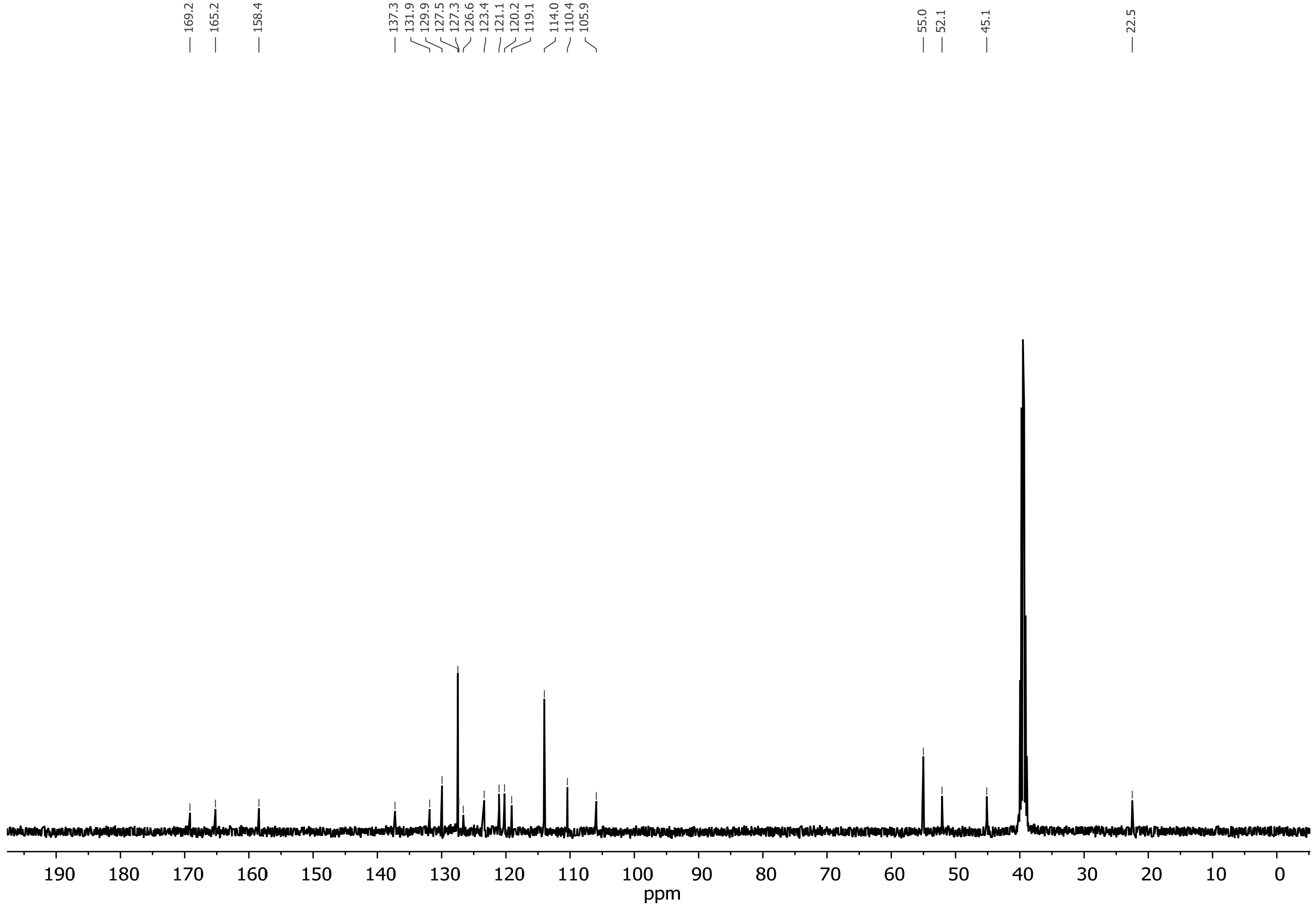

Table S2. Energies [kcal/mol; WB97XD/def2SVPP-LANL2DZ (SMD, DMF)//B97XD/def2TZVP(SMD, DMF)] for structures involved in reaction profiles of Figure 1

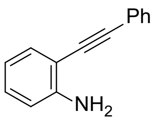

A_a

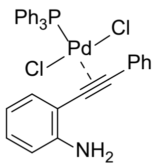

B_{aa}

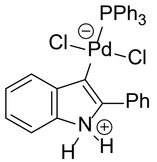

C_{aa}

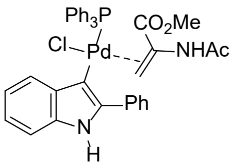

D_{aaa}

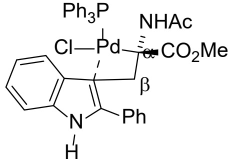

E_{aaa}

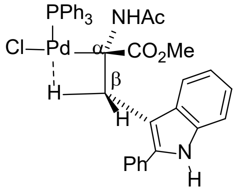

rot-E_{aaa}

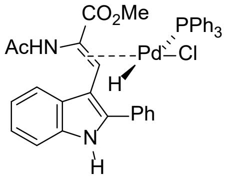

F_{aaa}

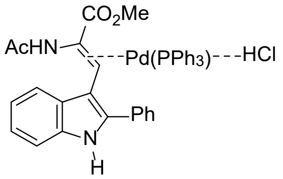

G_{aaa}

**wB97XD(SMD-DMF)/def2svpp-LANL2DZ**							**wB97XD(SMD-DMF)/def2TZVP**	
	**SCF**	**SCF+zpve**	**Enthalpy**	**TS**	**Free**	**ΔG**	**Free (TZ)**	**ΔG (TZ)**
$PdCl_2(PPh_3)_2$	-1957288.6	-1956937.3	-1956912.5	73.3	-1956985.8		-1958618.0	
methyl α-acetamidoacrylate (**3**)	-322506.0	-322410.7	-322403.2	31.3	-322434.5		-322885.8	
methyl acrylate	-192105.1	-192044.9	-192040.1	23.9	-192064.1		-192333.4	
PPh_3	-649763.4	-649589.9	-649580.0	37.4	-649617.5		-650318.5	
HCl	-289064.9	-289060.7	-289058.7	13.3	-289072.0		-289172.2	
**A_a**	-372860.3	-372728.5	-372720.2	33.9	-372754.0		-373277.0	-16.7
**B_{aa}**	-1680370.2	-1680061.2	-1680038.6	68.4	-1680107.0	**ref**	-1681559.8	**ref**
**$TSB_{aa}C_{aa}$**	-1680351.2	-1680042.5	-1680020.3	67.4	-1680087.7	19.3	-1681541.1	18.7
**C_{aa}**	-1680382.1	-1680070.2	-1680048.5	65.9	-1680114.4	-7.4	-1681570.0	-10.1
**D_{aaa}**	-1713820.6	-1713420.4	-1713393.0	76.1	-1713469.1	0.4	-1715277.5	-4.1
**$TSD_{aaa}E_{aaa}$**	-1713803.4	-1713403.4	-1713376.4	75.2	-1713451.6	17.9	-1715260.6	12.8
**E_{aaa}**	-1713848.7	-1713447.0	-1713419.9	75.3	-1713495.3	-25.7	-1715303.5	-30.1
**rot-E_{aaa}**	-1713848.6	-1713447.3	-1713420.3	75.7	-1713496.0	-26.5	-1715303.9	-30.6
**TSrot-$E_{aaa}F_{aaa}$**	-1713819.2	-1713421.5	-1713394.0	78.0	-1713472.0	-2.5	-1715276.9	-3.6
**F_{aaa}**	-1713827.2	-1713428.3	-1713400.7	77.3	-1713478.0	-8.4	-1715285.2	-11.8
**$TSF_{aaa}G_{aaa}$**	-1713823.3	-1713426.0	-1713398.7	76.0	-1713474.8	-5.2	-1715280.7	-7.4
**G_{aaa}**	-1713826.1	-1713429.6	-1713401.3	81.0	-1713482.3	-12.7	-1715283.8	-10.5

Table S2 (cont). Energies [kcal/mol; WB97XD/def2SVPP-LANL2DZ (SMD, DMF)//B97XD/def2TZVP(SMD, DMF)] for structures involved in reaction profiles of Figure 1.

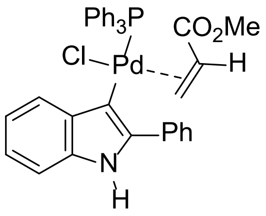

D$_{aab}$

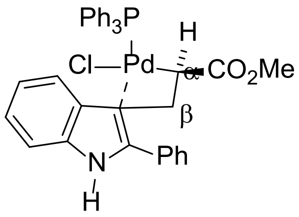

E$_{aab}$

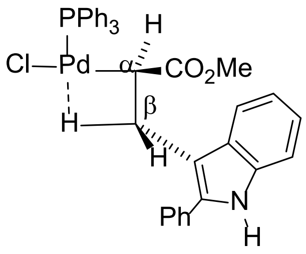

rot-E$_{aab}$

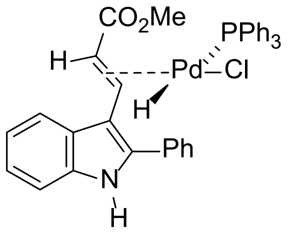

F$_{aab}$

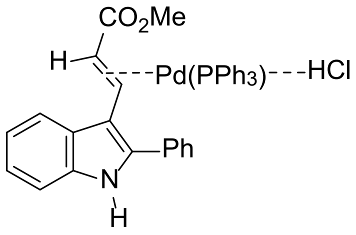

G$_{aab}$

	SCF	SCF+zpve	Enthalpy	TS	Free	ΔG	Free (TZ)	ΔG (TZ)
**D$_{aab}$**	-1583425.1	-1583060.2	-1583035.3	72.0	-1583107.3	-8.2	-1584731.9	-10.8
**TSD$_{aab}$E$_{aab}$**	-1583417.4	-1583052.5	-1583028.0	71.4	-1583099.4	-0.3	-1584724.3	-3.3
**E$_{aab}$**	-1583454.6	-1583087.8	-1583063.5	69.6	-1583133.1	-34.0	-1584759.0	-38.0
**rot-E$_{aab}$**	-1583450.0	-1583083.7	-1583059.4	69.7	-1583129.1	-30.0	-1584755.3	-34.3
**TSrot-E$_{aab}$F$_{aab}$**	-1583424.2	-1583060.5	-1583036.1	71.4	-1583107.5	-8.4	-1584730.8	-9.8
**F$_{aab}$**	-1583433.6	-1583069.6	-1583044.9	71.9	-1583116.8	-17.7	-1584740.6	-19.6
**TSF$_{aab}$G$_{aab}$**	-1583427.3	-1583065.2	-1583040.5	71.2	-1583111.7	-12.6	-1584733.3	-12.3
**G$_{aab}$**	-1583431.9	-1583069.8	-1583044.5	74.1	-1583118.6	-19.5	-1584740.3	-19.3

Table S2 (cont.). Energies [kcal/mol; WB97XD/def2SVPP-LANL2DZ (SMD, DMF)//B97XD/def2TZVP(SMD, DMF)] for structures involved in reaction profiles of Figure 1

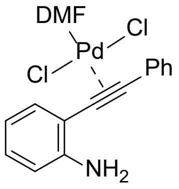

$\mathbf{B_{ab}}$

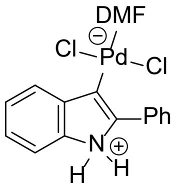

$\mathbf{C_{ab}}$

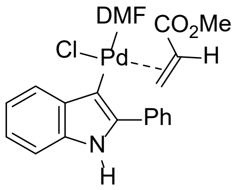

$\mathbf{D_{abb}}$

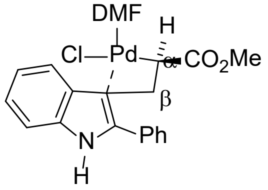

$\mathbf{E_{abb}}$

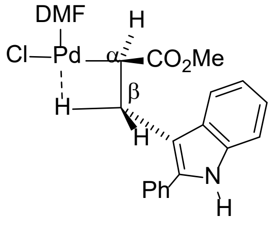

rot-$\mathbf{E_{abb}}$

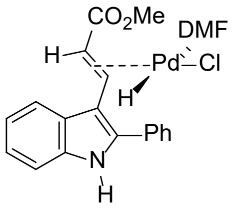

$\mathbf{F_{abb}}$

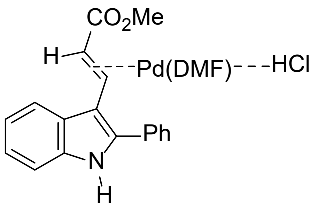

$\mathbf{G_{abb}}$

	SCF	SCF+zpve	Enthalpy	TS	Free	ΔG	Free (TZ)	ΔG (TZ)
**B**$_{ab}$	-1186365.5	-1186165.2	-1186148.7	54.0	-1186202.8	**ref**	-1187185.6	**Ref**
**TSB**$_{ab}$**C**$_{ab}$	-1186351.1	-1186150.9	-1186134.9	52.9	-1186187.8	14.9	-1187171.7	13.9
**C**$_{ab}$	-1186386.7	-1186183.6	-1186167.9	53.0	-1186220.9	-18.1	-1187205.3	-33.6
**D**$_{abb}$	-1089433.4	-1089176.9	-1089158.2	57.8	-1089215.9	-21.1	-1090369.9	-23.1
**TSD**$_{abb}$**E**$_{abb}$	-1089420.3	-1089164.3	-1089145.7	58.6	-1089204.3	-9.5	-1090357.2	-10.4
**E**$_{abb}$	-1089450.1	-1089192.4	-1089174.1	57.2	-1089231.2	-36.4	-1090384.7	-37.9
**rot-E**$_{abb}$	-1089438.8	-1089183.2	-1089164.5	58.6	-1089223.1	-28.3	-1090375.2	-28.4
**TSrot-E**$_{abb}$**F**$_{abb}$	-1089435.1	-1089178.5	-1089160.3	57.5	-1089217.8	-23.0	-1090369.6	-22.8
**F**$_{abb}$	-1089441.8	-1089186.1	-1089167.4	58.5	-1089225.9	-31.0	-1090378.5	-31.7
**TSF**$_{abb}$**G**$_{abb}$	-1089424.7	-1089171.9	-1089153.1	61.1	-1089214.2	-19.4	-1090361.2	-14.4
**G**$_{abb}$	-1089428.2	-1089174.4	-1089155.4	60.4	-1089215.8	-20.9	-1090365.6	-18.8

Figure S3. Reaction profiles starting from complexes $\mathbf{B_{ba}}$, $\mathbf{B_{ba\_carb}}$ or $\mathbf{B_{ca}}$ and methyl α-acetamidoacrylate (**3**) (profiles A-C, respectively) or from complex $\mathbf{B_{aa}}$ and methyl methacrylate (profile D) using $PdCl_2(PPh_3)_2$ as catalyst. Energy values in kcal/mol [WB97XD/def2SVPP-LANL2DZ (SMD, DMF)//B97XD/def2TZVP(SMD, DMF)].

A.

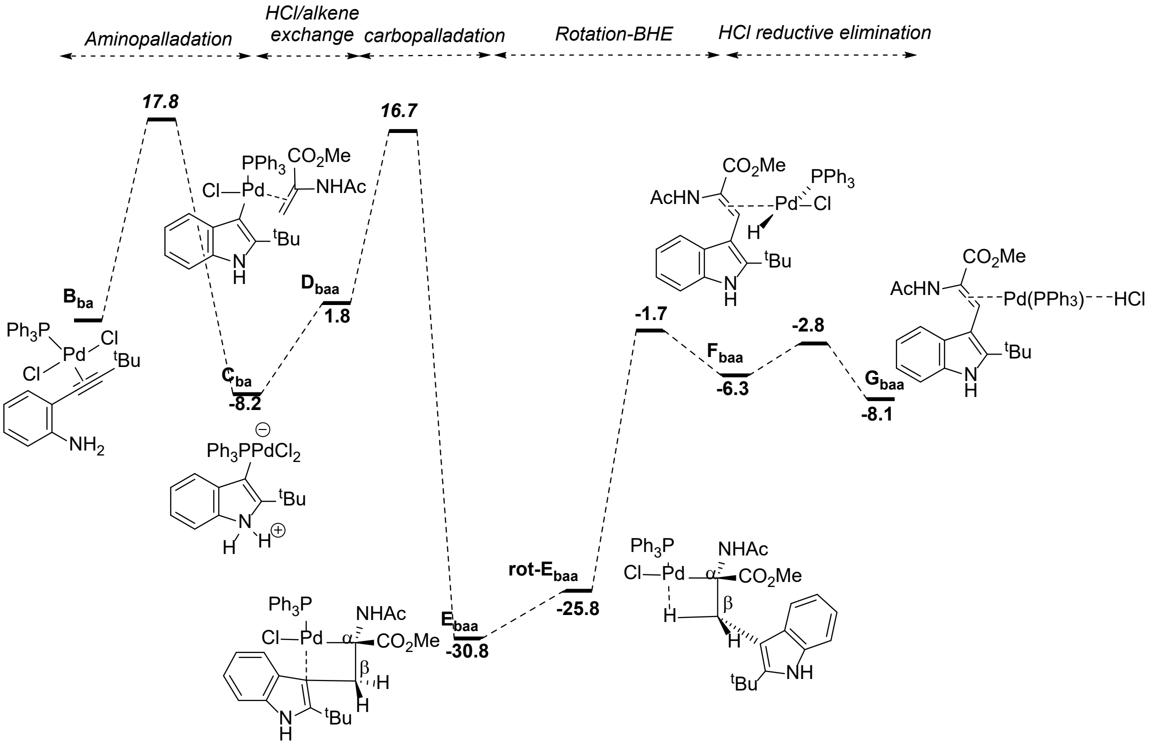

B.

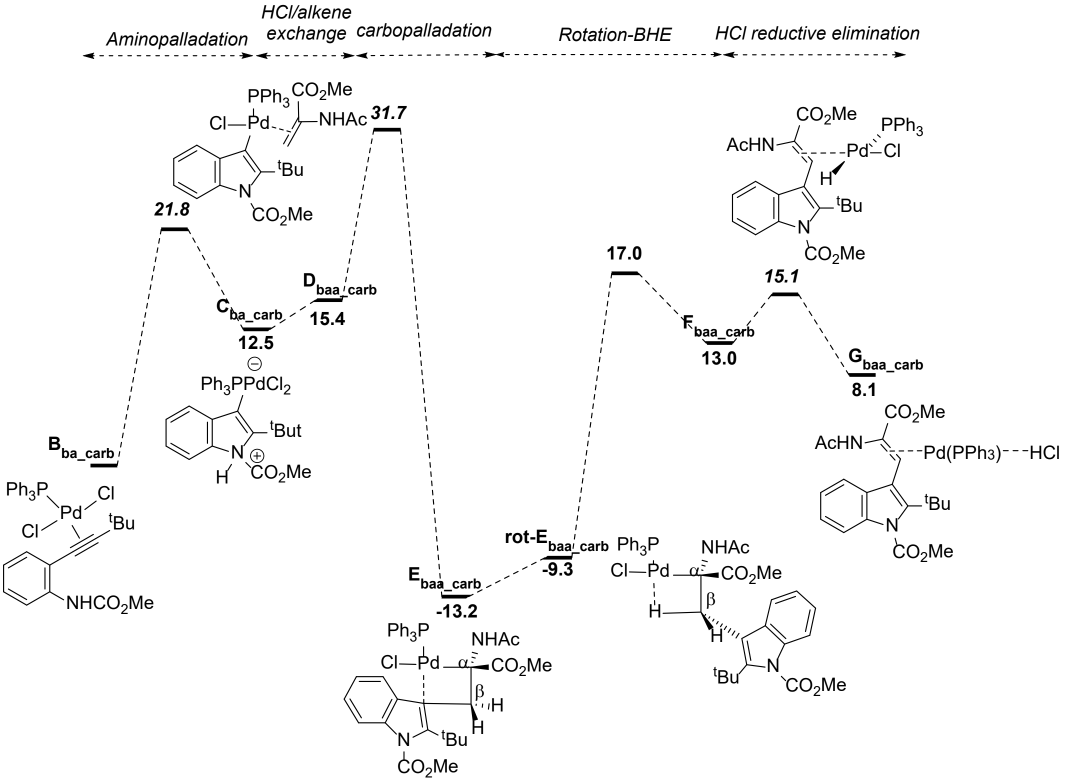

C.

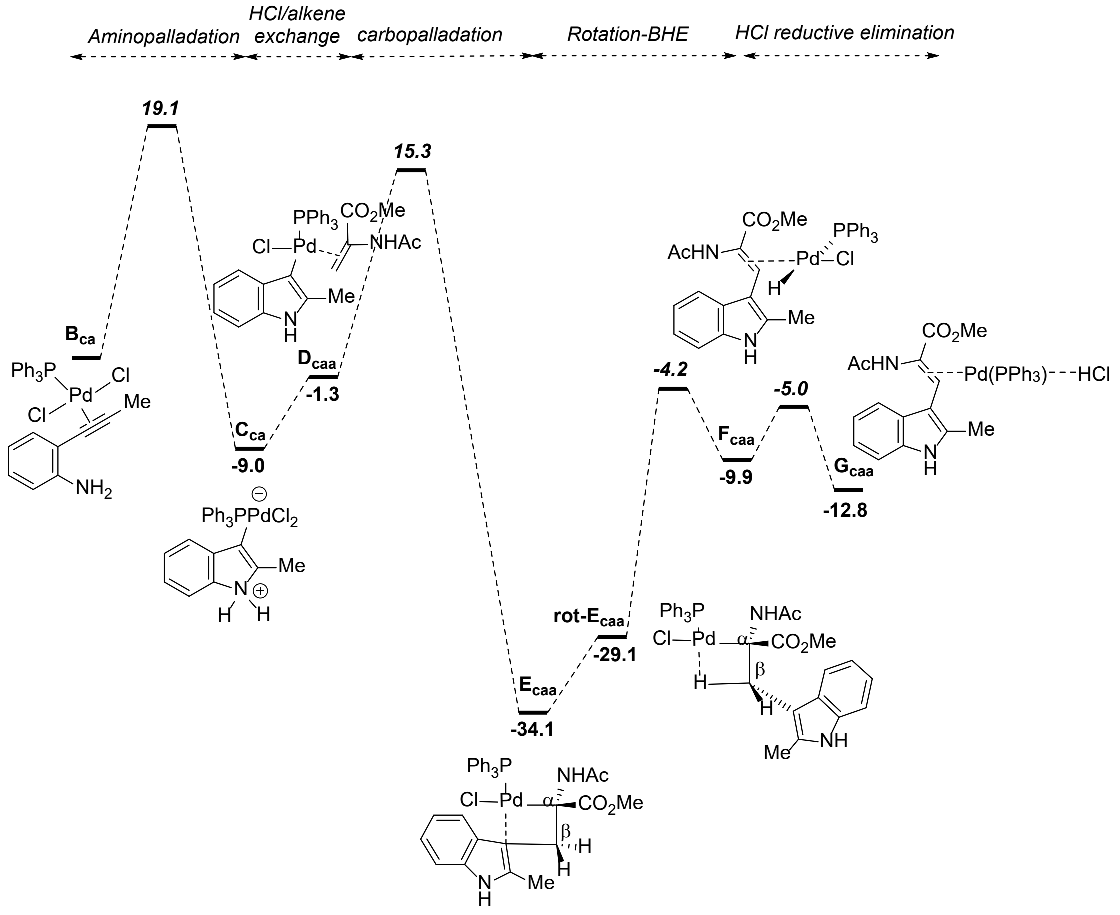

Aminopalladation
HCl/alkene exchange
carbopalladation
Rotation-BHE
HCl reductive elimination
19.1
15.3
B_ca
C_ca
-9.0
D_caa
-1.3
-4.2
F_caa
-9.9
-5.0
G_caa
-12.8
E_caa
-34.1
rot-E_caa
-29.1

D.

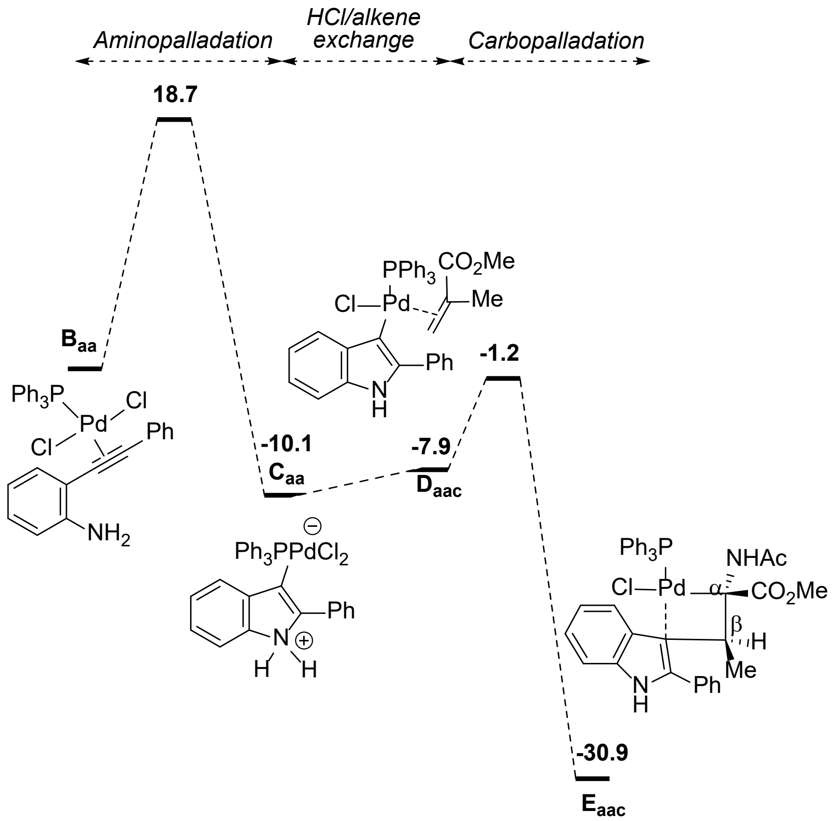

Aminopalladation
HCl/alkene exchange
Carbopalladation
18.7
B_aa
-10.1
C_aa
-7.9
D_aac
-1.2
-30.9
E_aac

Table S3. Energies [kcal/mol; WB97XD/def2SVPP-LANL2DZ (SMD, DMF)//B97XD/def2TZVP(SMD, DMF)] for structures involved in reaction profiles of Figure S3.

Profile A

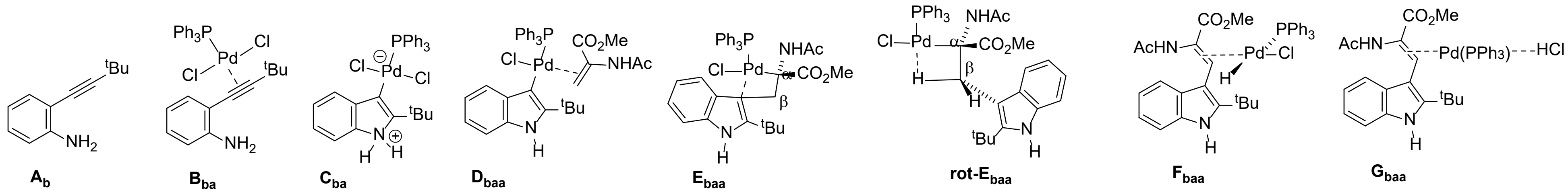

A_b B_{ba} C_{ba} D_{baa} E_{baa} rot-E_{baa} F_{baa} G_{baa}

wB97XD(SMD-DMF)/def2svpp-LANL2DZ							wB97XD(SMD-DMF)/def2TZVP	
	**SCF**	**SCF+zpve**	**Enthalpy**	**TS**	**Free**	**ΔG**	**Free (TZ)**	**ΔG (TZ)**
**A_b (1c)**	-326597.4	-326446.5	-326437.5	34.3	-326471.8		-326973.8	-15.6
**B_{ba}**	-1634109.4	-1633781.0	-1633757.9	67.9	-1633825.7	**ref**	-1635257.7	**ref**
**TS$B_{ba}C_{ba}$**	-1634090.7	-1633762.6	-1633739.9	68.2	-1633808.1	17.6	-1635239.9	17.8
**C_{ba}**	-1634118.9	-1633787.7	-1633765.6	65.6	-1633831.1	-5.4	-1635265.9	-8.2
**D_{baa}**	-1667553.3	-1667133.9	-1667105.9	76.8	-1667182.7	5.5	-1668969.4	1.8
**TS$D_{baa}E_{baa}$**	-1667538.2	-1667117.7	-1667090.6	74.1	-1667164.7	23.5	-1668954.5	16.7
**E_{baa}**	-1667588.0	-1667166.5	-1667139.0	75.5	-1667214.6	-26.3	-1669002.0	-30.8
**rot-E_{baa}**	-1667581.7	-1667160.3	-1667133.1	74.8	-1667207.9	-19.7	-1668997.1	-25.8
**TSrot-$E_{baa}F_{baa}$**	-1667556.3	-1667138.2	-1667110.6	76.8	-1667187.3	0.9	-1668972.9	-1.7
**F_{baa}**	-1667560.8	-1667142.6	-1667114.4	78.4	-1667192.8	-4.5	-1668977.5	-6.3
**TS$F_{baa}G_{baa}$**	-1667557.0	-1667140.9	-1667112.9	77.5	-1667190.3	-2.1	-1668974.0	-2.8
**G_{baa}**	-1667561.7	-1667145.5	-1667116.8	80.3	-1667197.1	-8.9	-1668979.3	-8.1

Table S3 (cont.). Energies [kcal/mol; WB97XD/def2SVPP-LANL2DZ (SMD, DMF)//B97XD/def2TZVP(SMD, DMF)] for structures involved in reaction profiles of Figure S3.

Profile B

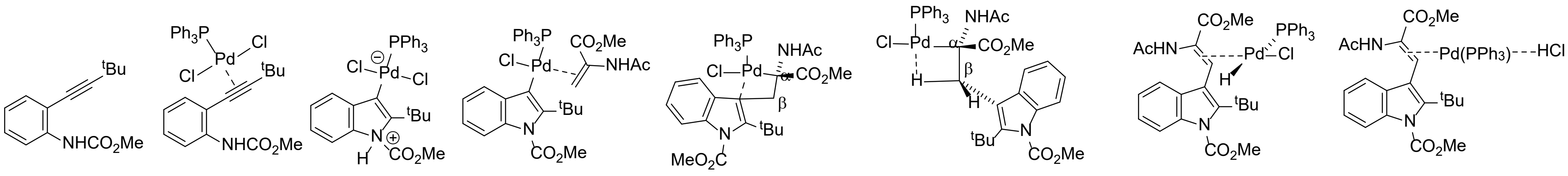

$\mathbf{A_{b\_carb}}$ $\mathbf{B_{ba\_carb}}$ $\mathbf{C_{ba\_carb}}$ $\mathbf{D_{baa\_carb}}$ $\mathbf{E_{baa\_carb}}$ **rot-**$\mathbf{E_{baa\_carb}}$ $\mathbf{F_{baa\_carb}}$ $\mathbf{G_{baa\_carb}}$

wB97XD(SMD-DMF)/def2svpp-LANL2DZ **wB97XD(SMD-DMF)/def2TZVP**

	SCF	SCF+zpve	Enthalpy	TS	Free	ΔG	Free (TZ)	ΔG (TZ)
**A_{b}_carb (2c)**	-469450.5	-469271.5	-469260.0	H	-469301.1		-469988.9	-16.3
**B_{ba_carb}**	-1776961.1	-1776605.3	-1776579.3	75.3	-1776654.6	**ref**	-1778272.1	**ref**
**TS$B_{ba_carb}C_{ba_carb}$**	-1776939.1	-1776582.7	-1776557.6	72.8	-1776630.4	24.2	-1778250.3	21.8
**C_{ba_carb}**	-1776949.8	-1776591.8	-1776566.9	72.8	-1776639.6	15.0	-1778259.7	12.5
**D_{baa_carb}**	-1810391.8	-1809944.7	-1809914.1	81.8	-1809995.9	21.2	-1811970.3	15.4
**TS$D_{baa_carb}E_{baa_carb}$**	-1810375.0	-1809928.0	-1809897.9	81.6	-1809979.5	37.6	-1811953.9	31.7
**E_{baa_carb}**	-1810422.7	-1809974.1	-1809943.8	81.9	-1810025.7	-8.6	-1811998.8	-13.2
**rot-E_{baa_carb}**	-1810417.2	-1809968.5	-1809938.6	81.2	-1810019.8	-2.6	-1811995.0	-9.3
**TSrot-$E_{baa\_carb}F_{baa\_carb}$**	-1810389.8	-1809944.1	-1809914.0	82.1	-1809996.1	21.0	-1811968.7	17.0
**F_{baa_carb}**	-1810393.4	-1809947.5	-1809916.7	84.1	-1810000.8	16.3	-1811972.7	13.0
**TS$F_{baa_carb}G_{baa_carb}$**	-1810390.9	-1809946.8	-1809916.3	83.4	-1809999.7	17.4	-1811970.6	15.1
**G_{baa_carb}**	-1810397.3	-1809953.5	-1809922.1	86.1	-1810008.2	8.9	-1811977.5	8.1

Table S3 (cont.). Energies [kcal/mol; WB97XD/def2SVPP-LANL2DZ (SMD, DMF)//B97XD/def2TZVP(SMD, DMF)] for structures involved in reaction profiles of Figure S3.

Profile C

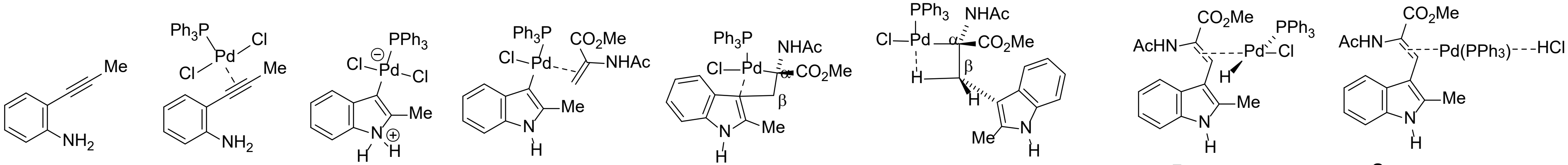

$\mathbf{A_c}$ $\mathbf{B_{ca}}$ $\mathbf{C_{ca}}$ $\mathbf{D_{caa}}$ $\mathbf{E_{caa}}$ **rot-**$\mathbf{E_{caa}}$ $\mathbf{F_{caa}}$ $\mathbf{G_{caa}}$

wB97XD(SMD-DMF)/def2svpp-LANL2DZ | **wB97XD(SMD-DMF)/def2TZVP**

	**SCF**	**SCF+zpve**	**Enthalpy**	**TS**	**Free**	**ΔG**	**Free (TZ)**	**ΔG (TZ)**
$\mathbf{A_c}$	-252666.3	-252568.7	-252562.1	28.6	-252590.7		-252955.6	-16.3
$\mathbf{B_{ca}}$	-1560176.2	-1559901.1	-1559880.4	62.5	-1559942.9	**ref**	-1561238.7	**ref**
$\mathbf{TSB_{ca}C_{ca}}$	-1560156.2	-1559881.4	-1559861.1	62.1	-1559923.3	19.7	-1561219.6	19.1
$\mathbf{C_{ca}}$	-1560186.6	-1559909.0	-1559889.1	61.9	-1559951.0	-8.1	-1561247.7	-9.0
$\mathbf{D_{caa}}$	-1593623.7	-1593258.4	-1593232.6	73.6	-1593306.2	-0.7	-1594953.6	-1.3
$\mathbf{TSD_{caa}E_{caa}}$	-1593606.5	-1593240.5	-1593215.3	71.0	-1593286.3	19.1	-1594936.9	15.3
$\mathbf{E_{caa}}$	-1593658.2	-1593290.9	-1593265.5	72.3	-1593337.8	-32.3	-1594986.3	-34.1
**rot-**$\mathbf{E_{caa}}$	-1593651.9	-1593284.8	-1593259.6	71.7	-1593331.2	-25.8	-1594981.4	-29.1
$\mathbf{TSrotE_{caa}F_{caa}}$	-1593625.9	-1593261.7	-1593236.2	72.6	-1593308.8	-3.4	-1594956.5	-4.2
$\mathbf{F_{caa}}$	-1593631.3	-1593266.0	-1593240.4	73.1	-1593313.5	-8.0	-1594962.1	-9.9
$\mathbf{TSF_{caa}G_{caa}}$	-1593627.0	-1593264.0	-1593238.4	72.9	-1593311.3	-5.9	-1594957.3	-5.0
$\mathbf{G_{caa}}$	-1593633.0	-1593270.3	-1593243.9	77.8	-1593321.7	-16.3	-1594965.0	-12.8

Table S3 (cont.). Energies [kcal/mol; WB97XD/def2SVPP-LANL2DZ (SMD, DMF)//B97XD/def2TZVP(SMD, DMF)] for structures involved in reaction profiles of Figure S3.

Profile D

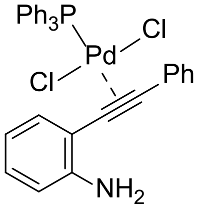

B_{aa}

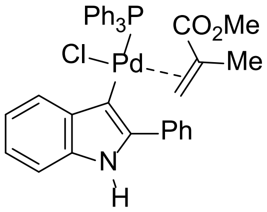

D_{aac}

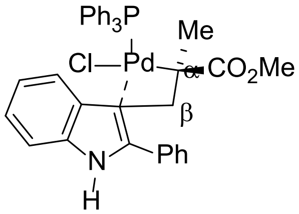

E_{aac}

wB97XD(SMD-DMF)/def2svpp-LANL2DZ | **wB97XD(SMD-DMF)/def2TZVP**

	SCF	SCF+zpve	Enthalpy	TS	Free	ΔG	Free (TZ)	ΔG (TZ)
methyl methacrylate	-216751.4	-216673.7	-216668.0	26.3	-216694.4		-217008.1	
**B_{aa}**	-1680370.2	-1680061.2	-1680038.6	68.4	-1680107.0	**ref**	**-1681559.8**	**ref**
**D_{aac}**	-1608068.7	-1607685.9	-1607660.3	72.6	-1607732.9	-3.5	-1609403.6	-7.9
**$TSD_{aac}E_{aac}$**	-1608062.0	-1607679.5	-1607654.2	71.5	-1607725.6	3.7	-1609396.9	-1.2
**E_{aac}**	-1608094.8	-1607710.3	-1607685.3	70.6	-1607755.9	-26.5	-1609426.6	-30.9

Table S4. Energies [kcal/mol; WB97XD/def2SVPP-LANL2DZ (SMD, DMF)//B97XD/def2TZVP(SMD, DMF)] for structures involved in the reaction profile of Figure 2.

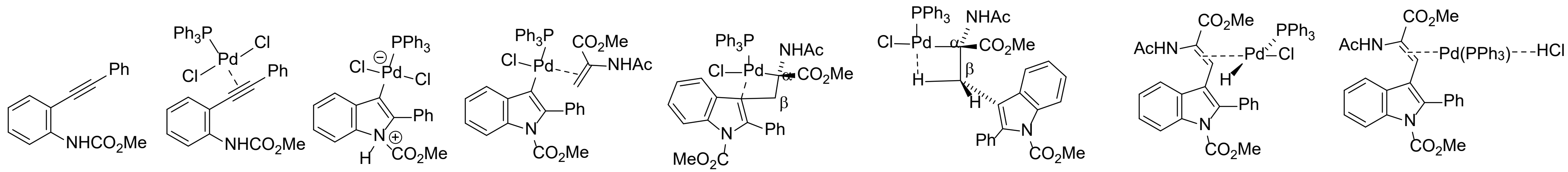

A_{a_carb} B_{aa_carb} C_{aa_carb} D_{aaa_carb} E_{aaa_carb} rot-E_{aaa_carb} F_{aaa_carb} G_{aaa_carb}

	wB97XD(SMD-DMF)/def2svpp-LANL2DZ						wB97XD(SMD-DMF)/def2TZVP	
	**SCF**	**SCF+zpve**	**Enthalpy**	**TS**	**Free**	**ΔG**	**Free (TZ)**	**ΔG (TZ)**
**A_{a_carb}**	-515712.9	-515553.2	-515542.2	41.6	-515583.8		-516291.6	-16.8
**B_{aa_carb}**	-1823222.1	-1822885.3	-1822860.0	75.4	-1822935.4	**ref**	-1824574.3	**ref**
**TS$B_{aa_carb}C_{aa_carb}$**	-1823202.0	-1822865.5	-1822840.8	73.7	-1822914.4	20.9	-1824553.8	20.4
**C_{aa_carb}**	-1823214.6	-1822876.2	-1822851.6	72.5	-1822924.1	11.2	-1824565.1	9.2
**D_{aaa_carb}**	-1856665.5	-1856238.2	-1856208.0	82.1	-1822924.1	7.8	-1858284.9	2.9
**TS$D_{aaa_carb}E_{aaa_carb}$**	-1856647.2	-1856220.2	-1856190.3	81.8	-1856272.1	25.8	-1858266.8	21.0
**E_{aaa_carb}**	-1856695.1	-1856266.7	-1856236.7	81.9	-1856318.6	-20.7	-1858312.0	-24.2
**rot-E_{aaa_carb}**	-1856691.6	-1856263.3	-1856233.4	82.2	-1856315.6	-17.7	-1858284.9	2.9
**TSrot-$E_{aaa\_carb}F_{aaa\_carb}$**	-1856660.3	-1856235.0	-1856204.8	83.9	-1856288.7	9.2	-1858280.9	6.9
**F_{aaa_carb}**	-1856667.3	-1856241.5	-1856211.0	83.7	-1856295.0	2.9	-1858287.9	-0.1
**TS$F_{aaa_carb}G_{aaa_carb}$**	-1856665.5	-1856241.5	-1856211.1	84.3	-1856295.4	2.5	-1858285.2	2.6
**G_{aaa_carb}**	-1856669.1	-1856245.0	-1856214.0	86.7	-1856300.7	-2.8	-1858289.8	-2.0

Table S5. Energies [kcal/mol; WB97XD/def2SVPP-LANL2DZ (SMD, DMF)//B97XD/def2TZVP(SMD, DMF)] for rotation-β-elimination to obtain *Z* and *E*-isomers.

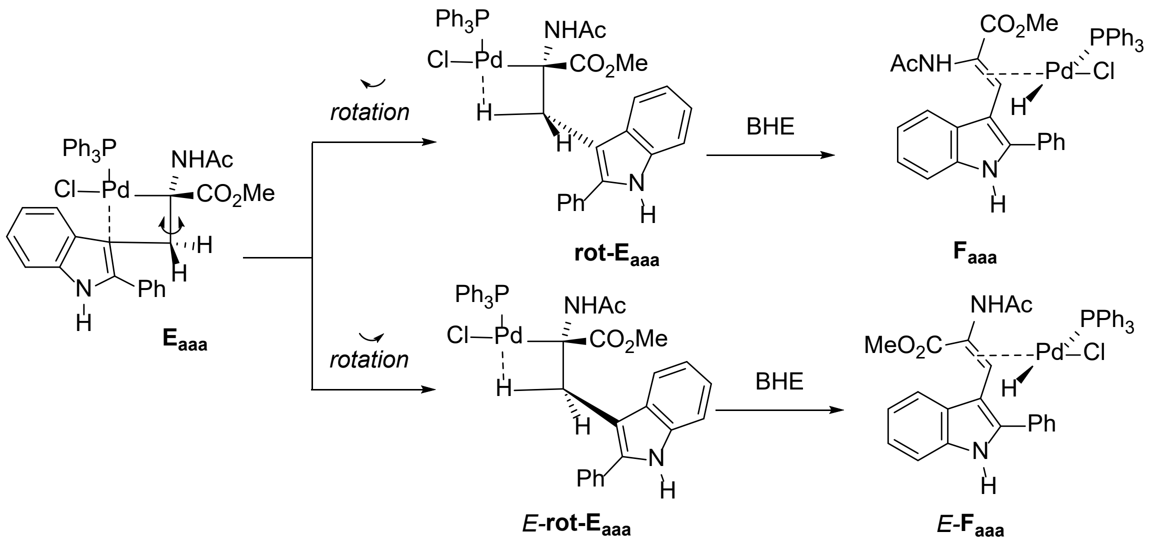

wB97XD(SMD-DMF)/def2svpp-LANL2DZ							wB97XD(SMD-DMF)/def2TZVP	
	**SCF**	**SCF+zpve**	**Enthalpy**	**TS**	**Free**	**ΔG**	**Free (TZ)**	**ΔG (TZ)**
**B$_{aa}$**	-1680370.2	-1680061.2	-1680038.6	68.4	-1680107.0	**ref**	-1681559.8	**ref**
**E$_{aaa}$**	-1713848.7	-1713447.0	-1713419.9	75.3	-1713495.3	-25.7	-1715303.5	-30.1
**rot-E$_{aaa}$**	-1713848.6	-1713447.3	-1713420.3	75.7	-1713496.0	-26.5	-1715303.9	-30.6
**TSrot-E$_{aaa}$F$_{aaa}$**	-1713819.2	-1713421.5	-1713394.0	78.0	-1713472.0	-2.5	-1715276.9	-3.6
**F$_{aaa}$**	-1713827.2	-1713428.3	-1713400.7	77.3	-1713478.0	-8.4	-1715285.2	-11.8
***E*-rot-E$_{aaa}$**	-1713838.2	-1713437.2	-1713410.2	75.6	-1713485.8	-16.3	-1715295.3	-21.9
**TS*E*-rot-E$_{aaa}$ *E*-F$_{aaa}$**	-1713813.0	-1713414.2	-1713387.3	75.7	-1713463.0	6.5	-1715270.0	3.4
***E*-F$_{aaa}$**	-1713817.4	-1713418.2	-1713391.0	75.6	-1713466.5	3.0	-1715273.1	0.3

Figure S4. Profile [kcal/mol; WB97XD/def2SVPP-LANL2DZ (SMD, DMF)//B97XD/def2TZVP(SMD, DMF)] for alternative cycloisomerization pathways based on palladium hydride complexes.

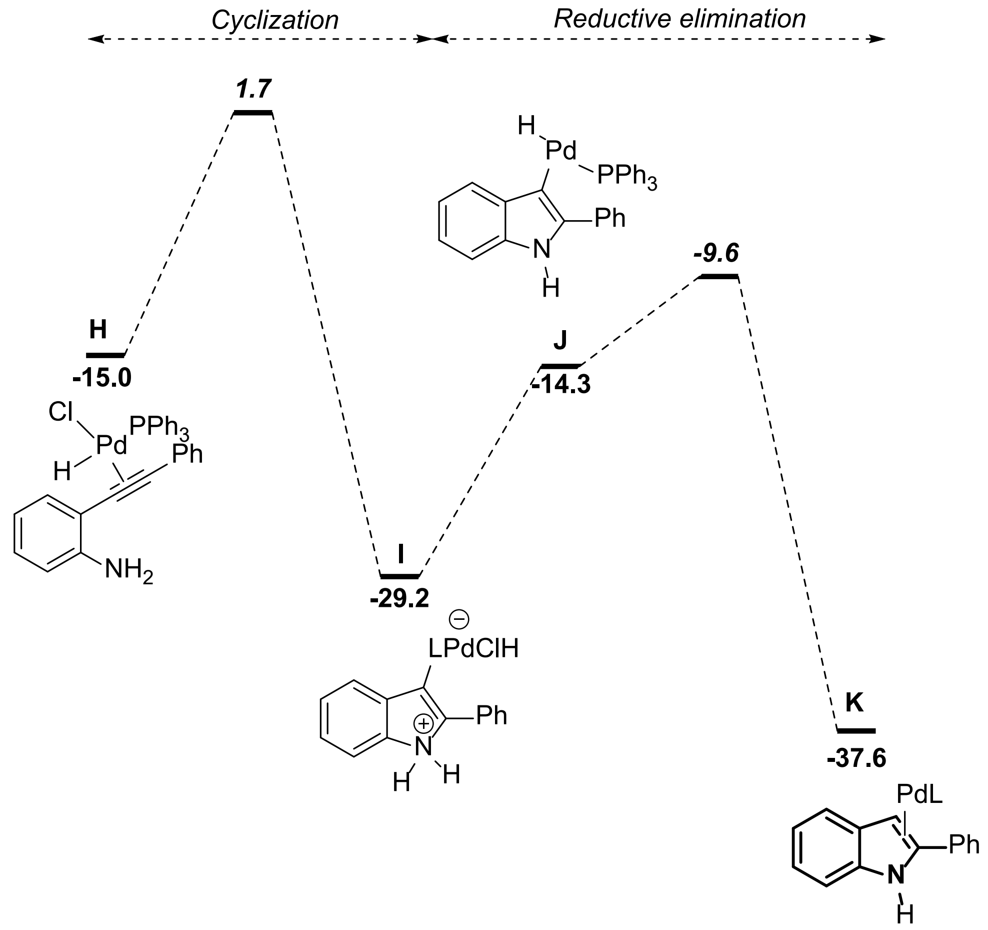

wB97XD(SMD-DMF)/def2svpp-LANL2DZ							wB97XD(SMD-DMF)/def2TZVP	
	**SCF**	**SCF+zpve**	**Enthalpy**	**TS**	**Free**	**DG**	**Free (TZ)**	**DG (TZ)**
**H**	-1392020.6	-1391707.8	-1391686.1	65.9	-1391752.0	-14.8	-1393112.9	-15.0
**TSHI**	-1392003.6	-1391690.5	-1391669.5	62.9	-1391732.4	4.8	-1393096.1	1.7
**I**	-1392036.8	-1391720.8	-1391700.3	61.5	-1391761.8	-24.6	-1393127.0	-29.2
**J**	-1102953.4	-1102647.5	-1102627.9	60.4	-1102688.3	-23.1	-1103939.9	-14.3
**TSJK**	-1102948.5	-1102643.7	-1102624.5	60.1	-1102684.6	-19.4	-1103935.2	-9.6
**K**	-1102978.2	-1102669.8	-1102650.2	62.0	-1102712.2	-47.0	-1103963.3	-37.6
